# Maintaining Bone Health in the Lumbar Spine: Routine Activities Alone Are Not Enough

**DOI:** 10.3389/fbioe.2021.661837

**Published:** 2021-05-19

**Authors:** Clément D. Favier, Alison H. McGregor, Andrew T. M. Phillips

**Affiliations:** ^1^Structural Biomechanics, Department of Civil and Environmental Engineering, Imperial College London, London, United Kingdom; ^2^Musculoskeletal Lab, Department of Surgery and Cancer, Imperial College London, London, United Kingdom

**Keywords:** lumbar vertebra, bone adaptation, structural finite element analysis, predictive modelling, strain-driven optimisation, sedentary behaviour

## Abstract

Public health organisations typically recommend a minimum amount of moderate intensity activities such as walking or cycling for two and a half hours a week, combined with some more demanding physical activity on at least 2 days a week to maintain a healthy musculoskeletal condition. For populations at risk of bone loss in the lumbar spine, these guidelines are particularly relevant. However, an understanding of how these different activities are influential in maintaining vertebral bone health is lacking. A predictive structural finite element modelling approach using a strain-driven algorithm was developed to study mechanical stimulus and bone adaptation in the lumbar spine under various physiological loading conditions. These loading conditions were obtained with a previously developed full-body musculoskeletal model for a range of daily living activities representative of a healthy lifestyle. Activities of interest for the simulations include moderate intensity activities involving limited spine movements in all directions such as, walking, stair ascent and descent, sitting down and standing up, and more demanding activities with large spine movements during reaching and lifting tasks. For a combination of moderate and more demanding activities, the finite element model predicted a trabecular and cortical bone architecture representative of a healthy vertebra. When more demanding activities were removed from the simulations, areas at risk of bone degradation were observed at all lumbar levels in the anterior part of the vertebral body, the transverse processes and the spinous process. Moderate intensity activities alone were found to be insufficient in providing a mechanical stimulus to prevent bone degradation. More demanding physical activities are essential to maintain bone health in the lumbar spine.

## 1. Introduction

Bone health relates to its capacity to resist the loads applied to it. It is widely accepted that bone adapts its structure, effectively the thickness of the cortex and the orientation and size of the trabeculae, to withstand the mechanical loads it is subjected to. Bone apposition occurs when the structure is over stimulated, while bone resorption is observed when bone is under stimulated. This process is called bone remodelling, and was theorised by Frost (1987, 2003) as the Mechanostat principle. Following this principle, sedentary behaviours and low physical activity levels may be considered as a cause of osteoporosis (Lau and Guo, 2011), and exercise is usually recommended for the management of this condition (Nelson et al., 2007; Sinaki et al., 2010; Rossini et al., 2016; Benedetti et al., 2018). Public health organisations typically recommend a combination of daily moderate intensity activities and regular more demanding physical activities to maintain musculoskeletal health (Davies et al., 2019). While these guidelines are generally promoted for an aging population, they are also pertinent for a younger population who may be sedentary due to home confinement as a consequence of the current COVID-19 pandemic (Narici et al., 2020). Moderate intensity activities usually refer to walking or cycling for a minimum of 20 min everyday. More demanding physical activities such as heavy gardening, carrying heavy shopping or resistance exercise, involving the major muscle groups, should be practised at least twice a week. To maintain a healthy musculoskeletal system, lifting, and carrying activities recruiting the erector spinae and the abdominal muscles are deemed to be of importance. It is intuitive to understand how activities recruiting specific muscle groups will help maintaining muscular health. However, this is less obvious with skeletal health and an understanding of how these activities can be influential in maintaining lumbar spine bone health is lacking.

Finite element modelling is a computational method that can be used to predict bone architecture under particular loading conditions if coupled with an optimisation algorithm. Applied to the lumbar spine, this modelling approach can provide the necessary information to understand which activities stimulate particular regions of lumbar vertebrae, essential for maintaining lumbar spine bone health. Detailed models of the complete lumbar spine have been developed with accurate geometry derived from CT images (Little et al., 2008; Ayturk and Puttlitz, 2011; Park et al., 2013), although these do not propose any prediction of bone remodelling to its mechanical loading environment. Many finite element models with varying levels of complexity have been developed to study bone remodelling. A common phenomenological approach consists in adapting bone toward a homeostatic state of strain, strain energy, or stress (Tsubota et al., 2003; Adachi et al., 2010; Homminga et al., 2012; Badilatti et al., 2015; van Rijsbergen et al., 2018). Other models use a mechanistic approach combining mechanical and metabolic factors in the adaptation algorithm (Huiskes et al., 2000; Taylor et al., 2004). Modelling of interstitial fluid flow has also been investigated (Tsubota et al., 2009; Hambli and Kourta, 2015; Tiwari et al., 2018). However, few of these predictive models consider a representation of the lumbar vertebrae using a realistic geometry. Macroscale continuum models developed by Homminga et al. (2012) and van Rijsbergen et al. (2018) both predict bone remodelling of the lumbar spine in an altered mechanical environment using isotropic bone material properties and a strain energy density driven optimisation. Although this approach allows for the study of bone stiffness adaptation, isotropic material properties in a continuum model cannot capture the directionality of trabeculae. Microscale continuum models developed by Tsubota et al. (2003) and Badilatti et al. (2015) are able to capture the remodelling of individual trabeculae in an entire vertebra under a particular loading condition. However, the model developed by Badilatti et al. (2015) is based on high resolution μCT images which are ethically complicated to obtain on healthy volunteers due to radiation exposure and long acquisition times. Tsubota et al. (2003) limited their study to a simplified geometry by creating an axisymmetric model based on a cross-sectional photograph of a vertebral body available in the literature (Mosekilde, 1990). Both studies applied simplified loading on the vertebral bodies of their models, which is not representative of the range of recommended physical activities. Despite the simplified loading conditions these models were still computationally demanding due to the number of continuum elements needed to represent bone at microscale.

To avoid these limitations and understand the influence of mechanical loading from a range of physical activities on the vertebral architecture, a modelling framework originally developed for the femur (Phillips, 2012; Phillips et al., 2015) and pelvis (Zaharie and Phillips, 2018, 2019) has been adapted to study the five lumbar vertebrae. It combines a subject-specific musculoskeletal model which provides realistic loading conditions with predictive structural finite element modelling based on the same subject for increased consistency. The structural finite element approach is a computationally efficient alternative to microscale continuum modelling of bones (Pothuaud et al., 2004; van Lenthe et al., 2006; Phillips, 2012; Zaharie and Phillips, 2019). It uses idealised elements (shells and trusses) to model the structure of the bone, allowing modelling of the vertebrae at mesoscale, where structural finite elements can be larger than the individual trabeculae but still capture the trajectories of the trabeculae and the overall bone architecture.

## 2. Materials and Methods

**Figure 1:**
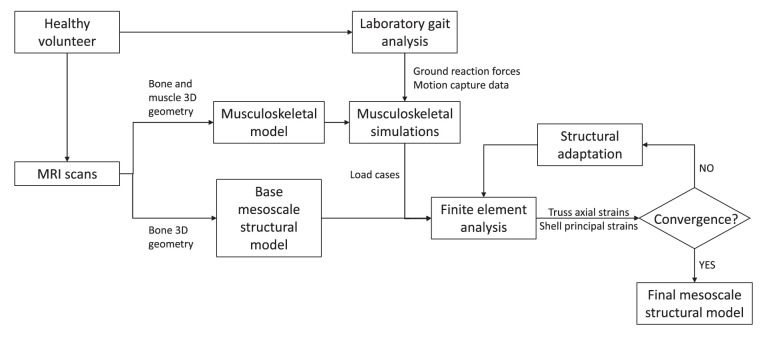
Modelling framework.

For each of the five lumbar vertebrae, the mesoscale structural model is obtained through iterative adaptation of a base finite element model subject to a loading envelope derived from musculoskeletal simulations of a range of physical activities representative of a healthy lifestyle. Figure 1 illustrates the modelling framework.

### 2.1. Musculoskeletal Modelling

The load cases applied to the finite element model were obtained with a previously validated subject-specific musculoskeletal model of the lumbar spine and lower limbs (Favier et al., 2021). This musculoskeletal model is based on full-body high-resolution MRI scans of a healthy volunteer (26 yo, 175 *cm*, 67.8 *kg*) with no history of spine pathologies. It was developed in OpenSim 3.3 (Delp et al., 2007) and is available to download at https://simtk.org/projects/llsm/. Full-body motion capture data were collected with the same healthy volunteer for eighteen activities following a previously developed protocol (Favier et al., 2021). The study was granted ethical approval by the NHS Health Research Authority (REC reference: 17/HRA/0465) and the Imperial College Research Ethics Committee (ICREC reference: 17IC3811), and the volunteer gave written informed consent. Recorded activities include six static positions of the spine (flexion at 20°, extension at 15°, lateral bending at 20° on both sides and axial rotation at 15° on both sides), five activities related to locomotion (level walking, stair ascent, stair descent, sit-to-stand, and stand-to-sit) and seven more demanding activities involving spine movements while sitting (maximum flexion, twisting, and lifting a box from floor to table (from both sides)) and standing (maximum flexion, lifting a box from floor to chest, twisting, and lifting a box from floor to floor (on both sides)). Musculoskeletal simulations were performed in OpenSim 3.3. An inverse kinematics approach was used to obtain joint angles for each of the recorded activities. Muscle forces were estimated using static optimisation where the sum of muscle activations squared was minimised for each frame of the kinematics. Joint reaction forces were also calculated at each lumbar joint using the JointReaction analysis tool available in OpenSim (Steele et al., 2012).

### 2.2. Finite Element Modelling

#### 2.2.1. Structural Finite Element Base Model

**Figure 2:**
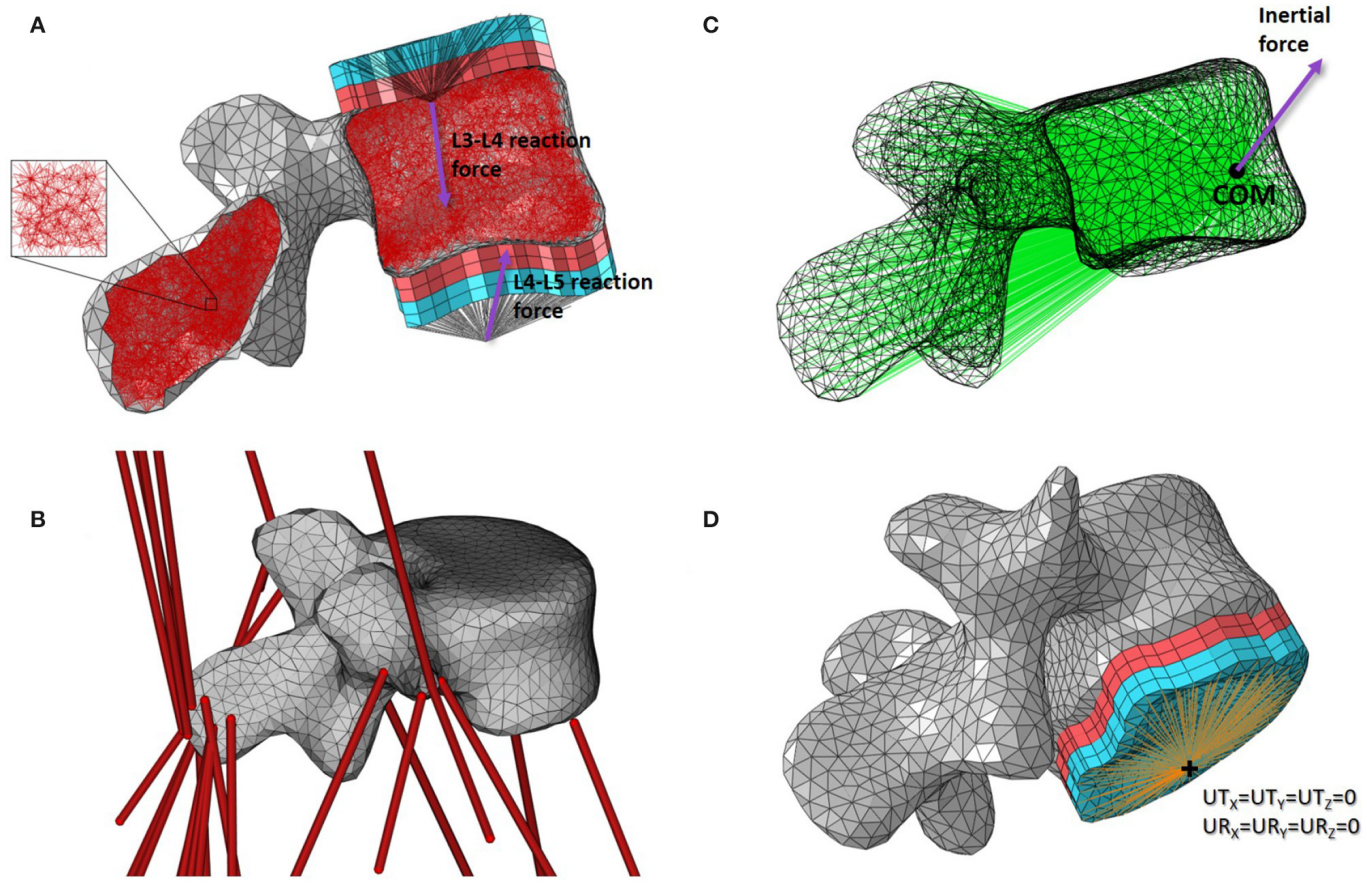
Base mesoscale structural model of L4. **(A)** Side cut with load applicators. **(B)** 3D view with musculotendon actuators from the musculoskeletal model. **(C)** Side view with inertial load applicator. **(D)** 3D view with boundary condition. Cortical shell elements are shown in grey and trabecular truss elements in dark red. Wedge elements of the softer layers of the load applicators are shown in light red while the stiffer layers elements are in light blue. Truss elements connecting the nodes on the external layers of the load applicators to the joint centres where the joint reaction force is applied are shown in black. Truss elements of the inertia applicator connecting the nodes of the cortical elements to the centre of mass (COM) of the lumbar segment where the inertial force is applied are shown in green. Beam elements connecting the nodes on the external layer of the load applicator to the duplicate node at the joint centre for the boundary condition are shown in orange.

Base models of the five lumbar vertebrae were created from the MRI scans of the same healthy volunteer recruited to develop the musculoskeletal model (Favier et al., 2021). These base structural models were generated using the same approach as described in Phillips (2012), Phillips et al. (2015), and Zaharie and Phillips (2018) and summarized here. The bone geometries were segmented in Mimics (Mimics Research 19.0, Materialise NV, Leuven, Belgium), reconstructed and exported as STL files following the protocol described in Favier et al. (2021). The STL files were then imported in 3-matic (3-matic Research 11.0, Materialise NV, Leuven, Belgium) where the coordinate systems of the vertebrae were adjusted to match the joint definitions used in the musculoskeletal model. The 3-matic meshing tools were used to create volumetric meshes of the vertebrae composed of four-noded tetrahedral elements with a 3 *mm* average edge length. These volumetric meshes were adapted to create structural meshes (Figure 2A) using MATLAB (The MathWorks, Inc., USA). Cortical bone was modelled with three-noded linear triangular shell elements defined by the nodes and element faces of the tetrahedral elements located on the external surface of the volumetric mesh. These shell elements were arbitrarily assigned an initial thickness of 0.1 *mm* in the base models. The internal nodes were used to create a network of two-noded truss elements representative of trabecular bone. Each node was linked to its closest sixteen neighbours. These truss elements were arbitrarily assigned an initial radius of 0.1 *mm* in the base models. The average element length of 3 *mm* and minimum nodal connectivity of 16 are considered to provide a sufficient mesh refinement and range of element directionalities to enable specific trabecular trajectories to develop during bone adaptation (Villette, 2016). All shell and truss elements were assigned linear isotropic material properties representative of bone material at the tissue level, with a Young's modulus of 18.0 *GPa* and a Poisson's ratio of 0.3 (Turner et al., 1999).

#### 2.2.2. Loading Conditions

Loading conditions include joint reaction forces and muscle forces estimated with the musculoskeletal model, as well as inertial loads experienced during movements.

##### 2.2.2.1. Joint Reaction Forces

For each lumbar vertebra, joint reaction forces calculated at the superior and inferior joint centres in OpenSim were transferred to the vertebral endplate areas with constructs called “load applicators” (Figure 2A). These applicators spread the point load calculated with the musculoskeletal model over the corresponding bone surface, performing a similar role to the intervertebral discs. The load applicators are composed of four layers of six-noded linear continuum wedge elements. To build each layer, surface nodes corresponding to the vertebral endplates were projected four times with a distance of 2 *mm* outward and orthogonally to the average endplate plane. Nodes of the vertebral endplate areas are shared between the load applicator and the cortical shell elements, reducing significantly the CPU time during the finite element analysis. Material properties of these applicators are adopted from the work of Phillips et al. (2015). The two layers closest to the bone were assigned linear elastic material properties of a soft material similar to cartilage (E = 10 *MPa*; ν = 0.49). The two external layers were assigned linear elastic material properties of a stiffer material similar to bone (E = 18 *GPa*; ν = 0.3). In the musculoskeletal model, intervertebral joints are modelled with three rotational degrees of freedom which only allow the transfer of forces. Since no moments are transferred through these idealised joints, truss elements were used to connect the joint centres as defined in the musculoskeletal model with the external nodes of the load applicators. These trusses were assigned a 2.5 *mm*^2^ circular cross sectional area and linear elastic material properties similar to bone (E = 18 *GPa*; ν = 0.3).

##### 2.2.2.2. Muscle Forces

The attachment site coordinates and fibre direction of the OpenSim musculotendon actuators acting on each lumbar vertebra (Figure 2B) were extracted from the musculoskeletal model at each time frame with a dedicated plug-in developed by Modenese (van Arkel et al., 2013). A MATLAB script was then used to locate the surface nodes closest to the attachment sites in the finite element model. Muscle forces were applied as point loads, with the magnitude and direction of the force vector corresponding to the muscle force estimated from the musculoskeletal simulations.

##### 2.2.2.3. Inertial Loads

To apply the inertial load of the lumbar segment to the vertebra, a construct called an “inertia applicator” based on the same concept as the load applicator was used. Spreading the inertial load over the volume of the vertebra is computationally demanding (Villette, 2016). Every cortical node of the vertebra was therefore connected to a node located at the centre of mass of the lumbar segment with soft truss elements (Figure 2C). These trusses have a circular cross sectional area of 2.5 *mm*^2^ and were assigned linear elastic material properties with a low stiffness (E = 5 *MPa*; ν = 0.3) to avoid stiffening of the model. The “body kinematics” tool available in OpenSim 3.3 was used to determine the position and velocity of the vertebra in the global coordinate system at each timeframe. The direction and magnitude of the inertial load were calculated based on these positions and velocities, and the mass of the lumbar segment defined in the musculoskeletal model. The inertial load was applied at the node located at the centre of mass of the lumbar segment.

#### 2.2.3. Boundary Conditions

The loading applied to the finite element model of the vertebra was obtained with the musculoskeletal model. At each time step, musculoskeletal simulations were solved for equilibrium of each segment. If all loads are applied, the vertebra should be at equilibrium in the finite element analysis, and no boundary condition should be needed. However, the musculoskeletal model represents bones as rigid bodies while the finite element model allows bones to deform, which compromises the equilibrium condition found in the musculoskeletal simulations. To ensure numerical stability of the finite element model, soft boundary conditions were applied using a similar approach as the load applicators. At the inferior joint, beam elements connecting the external nodes of the inferior endplate applicator with a coincident node at the joint centre were added (Figure 2D). This coincident node was constrained in all six degrees of freedom. The beam elements were assigned a circular cross section of 2.5 *mm*^2^, a Young's modulus of 1 *GPa* and a Poisson's ratio of 0.3. These relatively soft material properties compared to the load applicator properties prevent stiffening of the vertebra's structure induced by the boundary condition.

### 2.3. Bone Adaptation Algorithm

The bone adaptation algorithm used in this study was developed in the Structural Biomechanics Group at Imperial College London (Phillips, 2012; Phillips et al., 2015). With the structural mesoscale finite element approach, all truss and shell elements representing bone are assigned the same linear isotropic material properties. Shell thickness and truss cross-sectional area (arbitrarily assigned in the base models) are then optimised in the simulation of bone adaptation. The algorithm follows the Mechanostat hypothesis (Frost, 1987, 2003), optimising bone toward a target strain in an iterative process. This process is implemented with a combination of MATLAB and Python (Python Software Foundation, Beaverton, OR, USA) scripts, and successive finite element models are run using the Abaqus/Standard solver (Dassault Systèmes, Vélizy-Villacoublay, France).

At each iteration *i*, bone is subjected to a loading envelope of *n* load cases representing a combination of different activities. The maximum absolute strain for each element *j* is extracted and compared to the target strain. Equation (1) defines the maximum absolute strain in truss elements.

(1) $$\begin{array}{r} |\epsilon_{i,j}{|}_{max}=max\left(\left|\epsilon_{11,j,\lambda }\right|\right) \end{array}$$

where ϵ_11, *j*, λ_ is the axial strain in element *j* for the load case λ, with λ = 1, ..., *n*.

Equation (2) defines the maximum absolute strain in shell elements.

(2) $$\begin{array}{r} |\epsilon_{i,j}{|}_{max}=max\left(\left|\epsilon_{max,j,\lambda }^{b}|,|\epsilon_{min,j,\lambda }^{b}|,|\epsilon_{max,j,\lambda }^{t}|,|\epsilon_{min,j,\lambda }^{t}\right|\right) \end{array}$$

where $\epsilon_{max,j,\lambda }^{b}$, $\epsilon_{min,j,\lambda }^{b}$, $\epsilon_{max,j,\lambda }^{t}$, $\epsilon_{min,j,\lambda }^{t}$ are the maximum and minimum principal strains in the bottom and top surfaces of the shell element *j* for the load case λ, with λ = 1, ..., *n*.

The strain ranges associated with the Mechanostat (Frost, 1987, 2003) are given in Equation (3). The target strain was given a value of ϵ_*t*_ = 1250 μϵ (Aamodt et al., 1997; Phillips, 2012).

(3) $$\varphi_{i,j=}\left\{ \begin{array}{l} 1,\text{ for }0\mu \epsilon \leq {\left|\epsilon_{i,j}\right|}_{max}\leq 250\mu \epsilon \;(\text{Dead zone})\;\\ 1,\text{ for}250\mu \epsilon <{\left|\epsilon_{i,j}\right|}_{max}<1000\mu \epsilon \;(\text{Bone resorption})\;\\ 0,\text{ for }1000\mu \epsilon \leq {\left|\epsilon_{i,j}\right|}_{max}\leq 1500\mu \epsilon \;(\text{Lazy zone})\\ 1,\text{ for }{\left|\epsilon_{i,j}\right|}_{max}>1500\mu \epsilon \;(\text{Bone deposition})\; \end{array}\right.$$

One aspect of the adaptation algorithm that should be highlighted is the presence of a dead zone where bone is taken to resorp completely. In the base model, a randomised network of truss elements was created, resulting in a number of trusses in excess of that required. Trusses that are not needed to bear the load applied to the bone will fall in this dead zone.

For iteration *i*+1, the cross-sectional area of each truss element and the thickness of each shell element are adjusted using Equations (4) and (5), respectively. Adaptation of trabecular bone was given preference compared to adaptation of cortical bone at each iteration in order to avoid oscillation of the shell element thicknesses in the initial iterations.

(4) $$A_{i+1,j}\text{= }\left\{ \begin{array}{l} A_{i,j}\frac{{\left|\epsilon_{i,j}\right|}_{max}}{\epsilon_{t}}\text{ if }\phi_{i,j}=1\;\\ A_{i,j}\text{ if }\phi_{i,j}\neq 1 \end{array}\right.$$

where *A*_*i, j*_ is the cross section area of truss element *j*.

(5) $$T_{i+1,j}\text{= }\left\{ \begin{array}{l} \frac{T_{i,j}}{2}\left(1+\frac{{\left|\epsilon_{i,j}\right|}_{max}}{\epsilon_{t}}\right)\text{ if }\phi_{i,j}=1\;\\ T_{i,j}\text{ if }\phi_{i,j}\neq 1 \end{array}\right.$$

where *T*_*i, j*_ is the thickness of shell element *j*.

To increase computational efficiency, shell thicknesses were discretised linearly into 256 categories. The thickness of cortical bone varies between 0.2 and 0.9 *mm* in the vertebral body (Ritzel et al., 1997; Edwards et al., 2001). The thickness range of the shell elements was set between 0.1 and 2.0 *mm* to account for potential inter-subject variability. The same approach was used for the truss cross-sectional areas which were linearly discretised into 255 categories. The radius range of the truss elements was set between 0.1 and 2.0 *mm*, which characterises trabecular bone at a mesoscale level (Nagele et al., 2004; Phillips et al., 2015). An extra category with a radius of 1 μ*m* was added and allocated to elements in the dead zone. With such a small radius, the contribution of these elements is negligible while the numerical stability of the model is maintained. Elements in the dead zone were also allowed to regrow and be reassigned to one of the 255 categories if appropriate at a later iteration.

### 2.4. Loading Scenarios

Two loading scenarios were investigated in this study. A healthy scenario composed of all eighteen activities previously mentioned was investigated first. This scenario is representative of a healthy lifestyle. Adapting the base model of each lumbar vertebra to this set of load cases is expected to provide converged models with a trabecular and cortical architecture similar to that observed in healthy vertebrae. A second scenario representative of a more sedentary lifestyle was also investigated. For this scenario, the converged healthy models obtained previously were adapted to the same set of load cases where the seven more demanding activities involving lifting tasks and large combined movements of the spine toward the limits of the range of motion in the three anatomical planes were removed.

**Figure 3:**
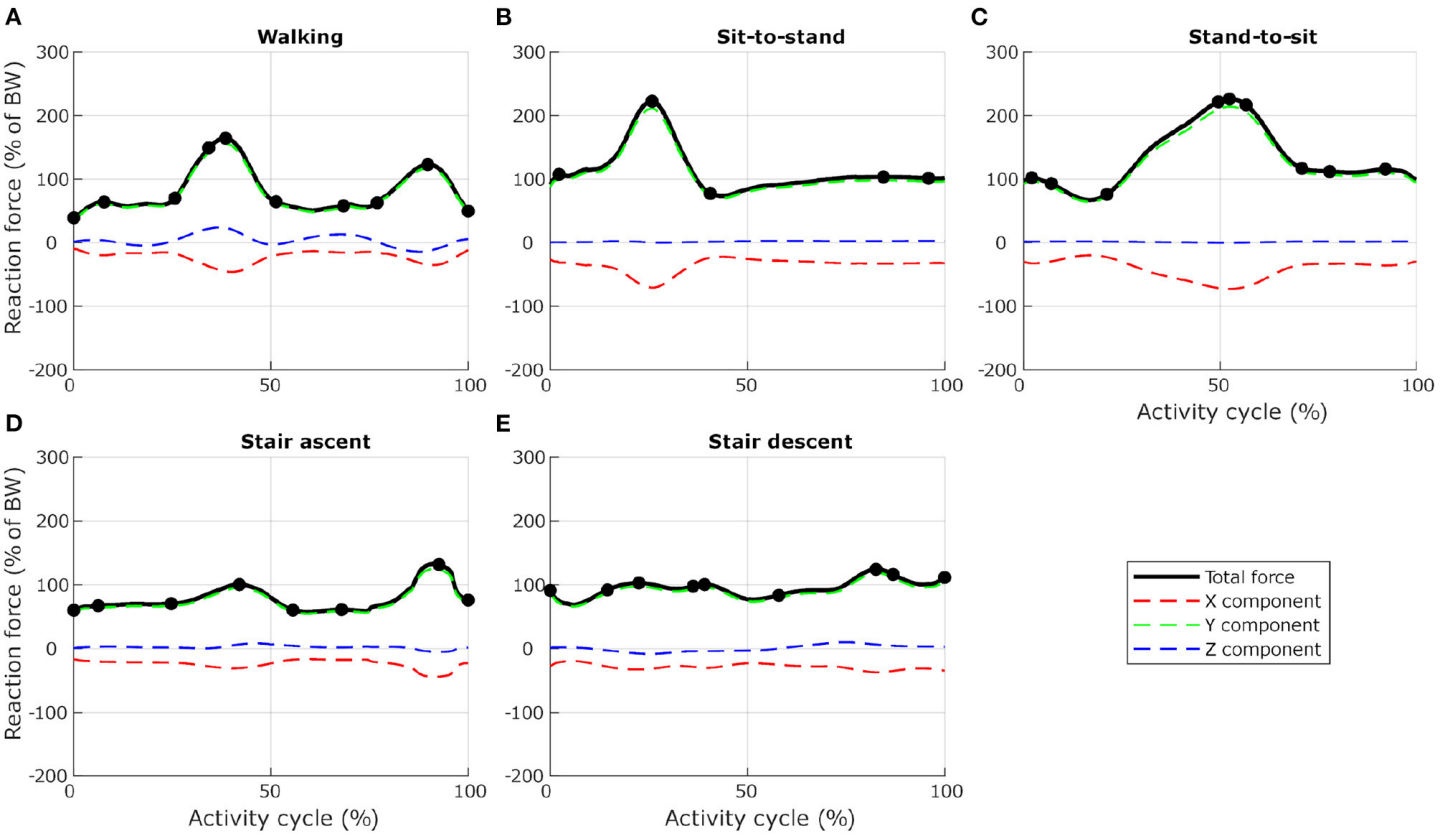
Selection of load cases for L4, for five activities related to locomotion (**(A)** level walking, **(B)** standing up from a chair, **(C)** sitting down on a chair, **(D)** walking up the stairs, and **(E)** walking down the stairs). Total reaction force at L4–L5 joint derived from the musculoskeletal model and normalised to body weight (BW) are shown as black lines. Dots indicate the frames selected for the finite element analysis. Red, green, and blue dashed lines show X, Y, and Z components of the reaction force expressed in the vertebra coordinate system.

**Figure 4:**
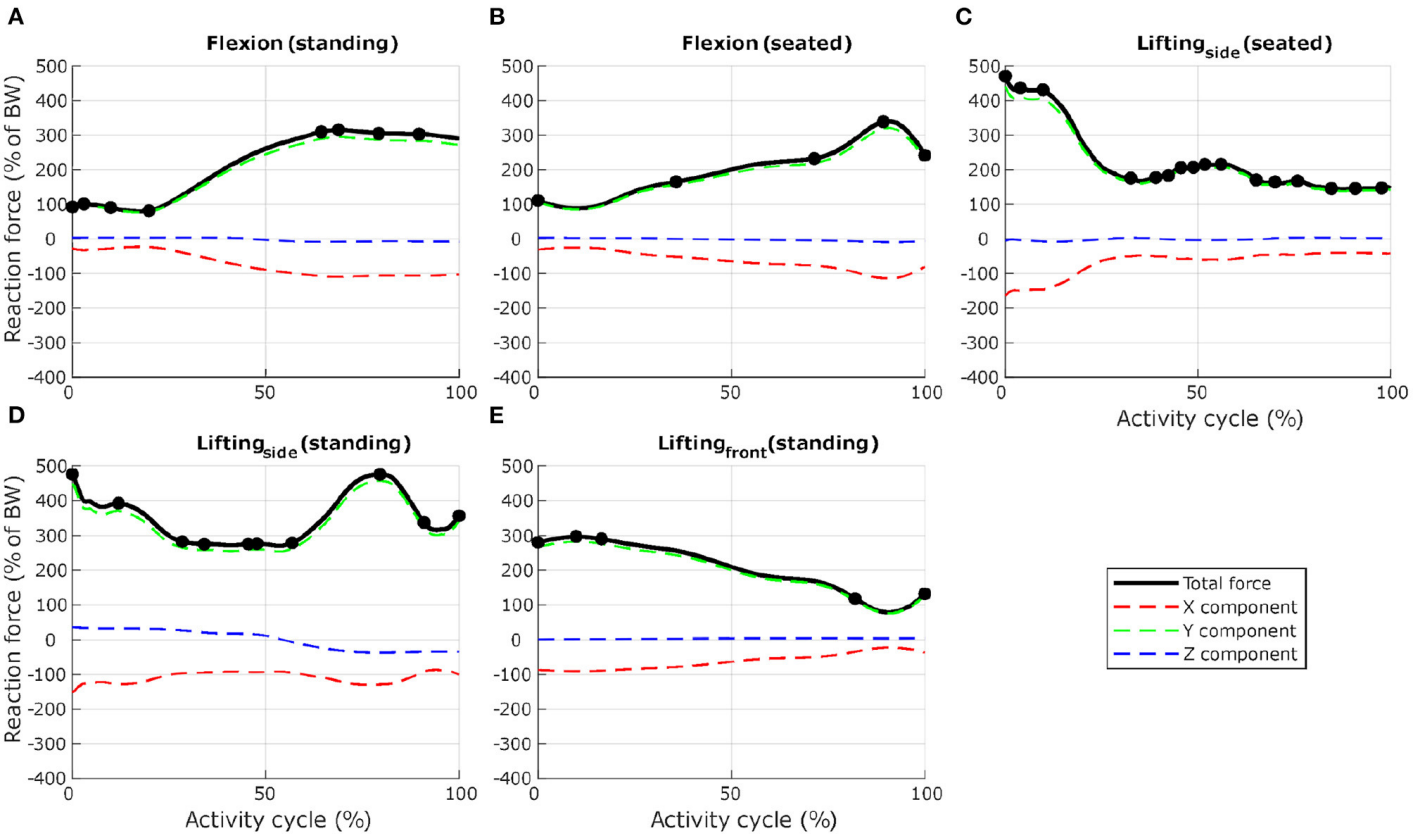
Selection of load cases for L4, for seven activities involving spine movements (**(A)** forward flexion from upright standing to maximum flexion, **(B)** forward flexion from upright sitting, **(C)** in a seated position, twisting and lifting a box from the floor, from the right side to a table in front, **(D)** in a standing position, twisting and lifting a box from the floor, from the left side to the right side, and **(E)** in a standing position, lifting a box from the floor in front to the chest). Total reaction force at L4–L5 joint derived from the musculoskeletal model and normalised to body weight (BW) are shown as black lines. Dots indicate the frames selected for the finite element analysis. Load cases obtained with activities **(C,D)** were mirrored through the sagittal plane to obtain loading on the other side. Red, green, and blue dashed lines show X, Y, and Z components of the reaction force expressed in the vertebra coordinate system.

To ensure computational efficiency for each lumbar vertebra model, subsets of load cases were selected from the 12 dynamic activities based on the reaction force calculated at the inferior idealised joint in the musculoskeletal model. For each activity, the full set of frames was first subsampled at 10 *Hz* (every 10 frames) to reduce the number of frames for the simulations. Any peak value was also added to the subset. This initial subset was then optimised by removing frames until a 1% difference between the integrated load for the initial subset of frames and the integrated load for the final subset of frames was reached. At each selected frame, the corresponding muscle forces, joint reaction forces and inertia forces were applied in consecutive steps in the finite element model. Figures 3, 4 show the selected load cases from the dynamic activities for L4 with this method. For the static activities, a single frame was selected in the middle of each activity to obtain six additional load cases. The reaction force at L4–L5 joint for these load cases is shown in Table 1 for reference. Load cases for the other vertebrae are available in the Supplementary Material.

**Table 1:** Reaction force at L4–L5 joint derived from the musculoskeletal model and used for the finite element analysis for six static positions of the spine.

	**X component (% of BW)**	**Y component (% of BW)**	**Z component (% of BW)**	**Total reaction force (% of BW)**
Flexion 20°	−60.5	174.8	3.5	185.0
Extension 15°	−74.5	190.1	4.2	204.2
Lateral bending 20° (right)	−45.8	127.3	13.2	135.9
Lateral bending 20° (left)	−45.4	129.0	−3.9	136.8
Axial rotation 15° (right)	−43.2	118.4	−2.2	126.0
Axial rotation 15° (left)	−29.1	90.2	5.3	94.9

*Forces are normalised to body weight (BW)*.

### 2.5. Adapted Bone Architecture Analysis

The structural finite element approach used in this study allows a direct visual observation of the cortical and trabecular bone architecture. *In-vivo* observations of vertebral architecture are not abundant in the literature for healthy young subjects, as most studies focus on elderly and pathological populations. After adaptation to the healthy scenario, the structural architecture of the L4 model was compared to a description of the vertebra's internal architecture made by Gallois and Japiot (1925). Trabecular anisotropy in the lumbar vertebrae was characterised using coloured lines at each node. Every truss element was expressed as a normalised vector of X, Y, and Z components in the vertebra's coordinate system. These element vectors were then weighted based on the cross-sectional area of the elements. For each node, connected weighted element vectors were summed to create a node vector. The norm of this node vector was used to scale the length of the line at each node. Components of the normalised node vector were used as RGB values for the line's colour, with X, Y, and Z components corresponding to red, green, and blue, respectively. With this method, if elements linking to a node are oriented along the X axis (respectively Y or Z) only, a red (respectively green or blue) line will be produced at this node. Similarly, if elements linking to a node are oriented at ±45° in the XY plane (respectively XZ plane or YZ plane), a yellow (respectively magenta or cyan) line will be produced at this node as a combination of red and green light. A white dot indicates a node without elements in the size range being looked at connected to it. A difference is made between the trusses with a radius larger than 0.1 *mm* referred to as the “primary structure,” and the trusses with a radius of 0.1 *mm* referred to as the “secondary structure.” The trabecular trusses of the primary structure resist the major loads experienced by the vertebra while the secondary structure is believed to give a base stiffness to the bone.

Cortical thickness and trabecular architecture of the five lumbar vertebrae adapted were also analysed in the healthy and the sedentary scenarios. To understand the influence of the different activities, each finite element (cortical shell or trabecular truss) was colour-coded in the adapted models based on the load case which resulted in the absolute maximum strain value (as defined in Equations 1 and 2). This allows a direct visualisation of the activities most beneficial to maintaining bone health, and gives an understanding of which areas of the lumbar vertebrae are stimulated by a given activity.

## 3. Results

### 3.1. Healthy Scenario

On average, the structural finite element models converged in 25 iterations for the healthy scenario. The relative density, calculated as the ratio between the volume of all bone elements (cortical and trabecular) and the total volume of the vertebra, is 20.27% on average for the five adapted lumbar vertebrae. This value is within the range reported by Eriksen et al. (2002) (19.0%, SD 8.5%) and Muller (2004) (17.9%, SD 6.7%). 16.97% of the initial truss elements representing trabecular bone in the base models ended in the dead zone after adaptation to 115.2 load cases on average. The remaining truss elements have an average connectivity of 17.20 (SD 4.16). Characteristics of the converged models can be found in Table 2. Converged structural finite element models of the five lumbar vertebrae adapted to the healthy scenario are available in the Supplementary Material.

**Table 2:** Characteristics of the converged mesoscale structural finite element models after adaptation to the healthy and sedentary scenarios.

		**L1**	**L2**	**L3**	**L4**	**L5**	**Average**
	Cortical elements	2,964	3,390	3,620	3,600	3,712	3457.2
	Trabecular elements (initial mesh)	89,042	101,805	115,063	115,988	130,460	110471.6
	Vertebra volume (*mm*^3^)	54,400	59,320	69,630	70,094	78,820	66,453
Healthy	Load cases	114	115	118	116	113	115.2
scenario	Iterations to convergence	25	23	30	20	27	25
	Trabecular elements (converged model)	79,826	86,905	93,452	98,558	96,483	91044.8
	Trabecular connectivity						
	Mean (SD)	18.20 (3.96)	17.45 (4.23)	16.77 (4.30)	17.21 (3.96)	16.38 (4.36)	17.20 (4.16)
	Minimum	1	1	1	1	1	1
	Maximum	49	45	53	46	31	44.8
	Trabecular volume (*mm*^3^)	9,185	7,948	7,828	9,010	10,050	8,804
	Cortical volume (*mm*^3^)	6,026	4,608	3,875	4,877	2,249	4,327
	Relative density (% of bone volume over total volume)	27.96	21.17	16.81	19.81	15.60	20.27
	Dead elements (% of initial trabecular elements)	10.35	14.64	18.78	15.03	26.04	16.97
Sedentary	Load cases	51	48	51	51	52	50.6
scenario	Iterations to convergence	24	38	56	39	44	40.2
	Trabecular elements (converged model)	49,535	57,404	38,233	63,703	61,477	54070.4
	Trabecular connectivity						
	Mean (SD)	14.70 (4.64)	13.36 (4.53)	10.75 (4.39)	12.78 (4.36)	13.88 (4.67)	13.10 (4.52)
	Minimum	1	1	1	1	1	1
	Maximum	28	37	26	36	28	31
	Trabecular volume (*mm*^3^)	3,074	3,246	2,468	3,765	4,729	3,456
	Cortical volume (*mm*^3^)	1,549	1,628	1,666	3,476	1,719	2,008
	Relative density (% of bone volume over total volume)	8.50	8.22	5.94	10.33	8.18	8.23
	Dead elements (% of initial trabecular elements)	37.95	33.95	59.09	35.36	36.28	40.53

**Figure 5:**
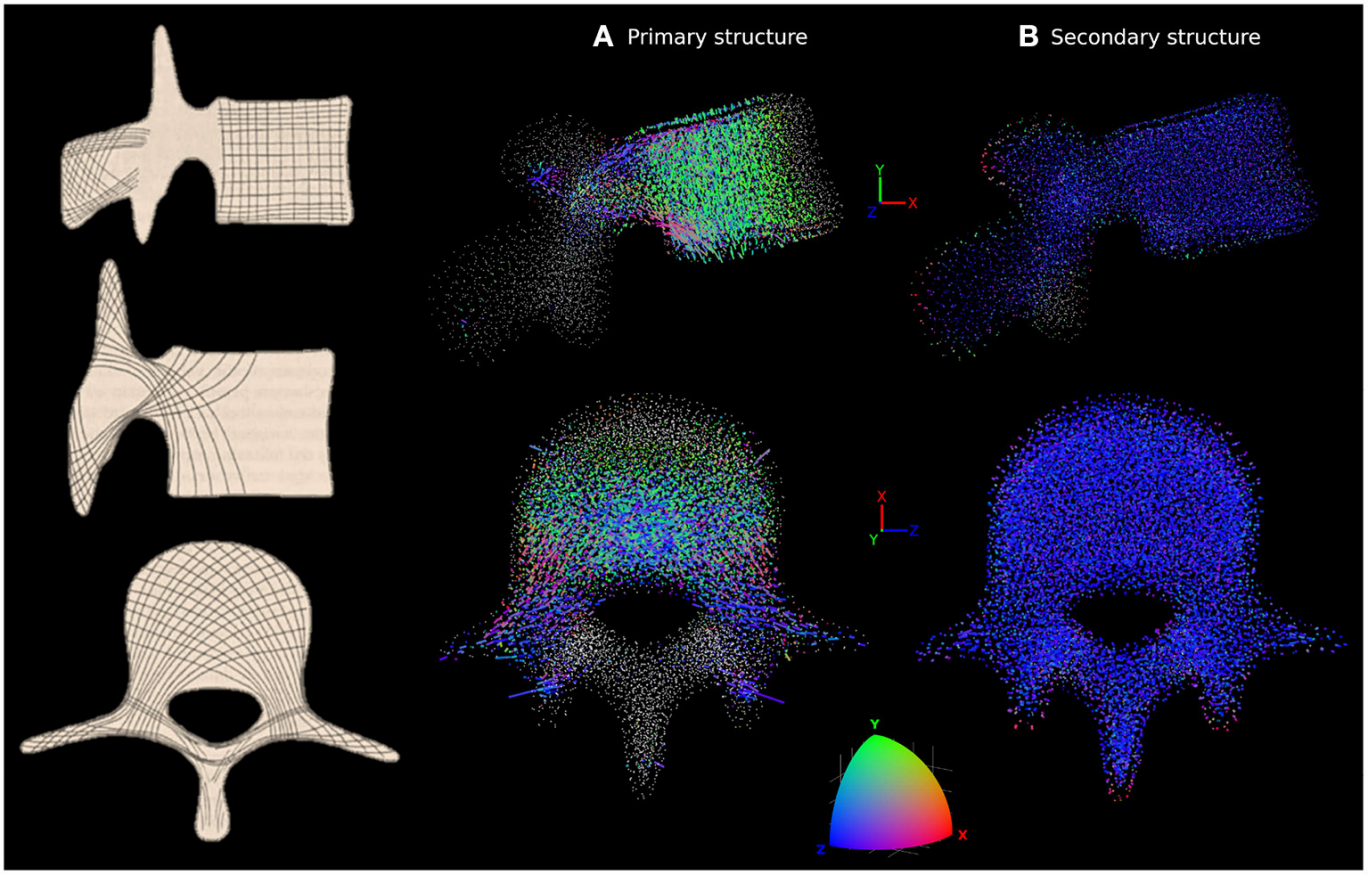
Characterisation of anisotropy in the trabecular bone of L4 adapted to the healthy scenario. *In-vivo* observations by Gallois and Japiot (1925) are shown on the left. **(A)** Shows the orientation of the trabecular trusses of the primary structure (with a radius larger than 0.1 *mm*). **(B)** Shows the orientation of the trabecular trusses of the secondary structure (with a radius of 0.1 *mm*). For **(A,B)**, side view (top row) and top view (bottom row) are shown. Lines are attached to each trabecular node, with colour and length varying respectively with the orientations and radii of the truss elements connected to that particular node. The colour scale at the bottom shows how the colour of the lines should be interpreted. Orientation along the X, Y, or Z axes are in red, green, or blue, respectively. Any orientation that is not colinear with these axes shows as a combination of red, green and blue. A white dot indicates a node without elements in the size range being looked at connected to it.

The adapted trabecular trajectories were studied with the coloured lines method for anisotropy characterisation. Figure 5 shows the line plot for L4 compared to *in-vivo* observations by Gallois and Japiot (1925) on the left. Figure 5A focuses on trabecular truss elements with a radius larger than 0.1 *mm* forming the primary structure, while Figure 5B shows only the secondary structure composed of elements with a radius of 0.1 *mm*. The primary structure compares favourably with observations made by Gallois and Japiot (1925). There is a clear orientation along the Y axis for the trabeculae in the vertebral body as green is the dominant colour. This group of trabeculae runs perpendicular to the endplates and resists vertical compression. Blue is the dominant colour in the transverse processes and the vertebral arch, indicating that most trabeculae run medio-laterally in these parts of the vertebra, resisting tension in the transverse processes. Elements running diagonally across the vertebral body and the pedicles, finishing in the transverse and superior articular processes can also be identified in pink, purple, and grey. For the elements of the secondary structure, the lines are predominantly blue, indicating a principal orientation of the smaller truss elements along the Z axis. Line plots and MATLAB figure files for the five lumbar vertebrae are available in the Supplementary Material.

### 3.2. Sedentary Scenario

The average number of load cases over the five lumbar vertebrae was 50.6 for the eleven activities of the sedentary scenario. The structural finite element models converged in 40.2 iterations on average with a relative bone density of 8.23%, a mean connectivity of 13.10 (SD 4.52) and 40.53% of the trabecular elements in the dead zone (Table 2). Relative density is 58.39% lower than in the healthy scenario. This shows that moderate intensity activities alone are insufficient in providing a mechanical stimulus to prevent a decrease of bone density in the lumbar spine.

**Figure 6:**
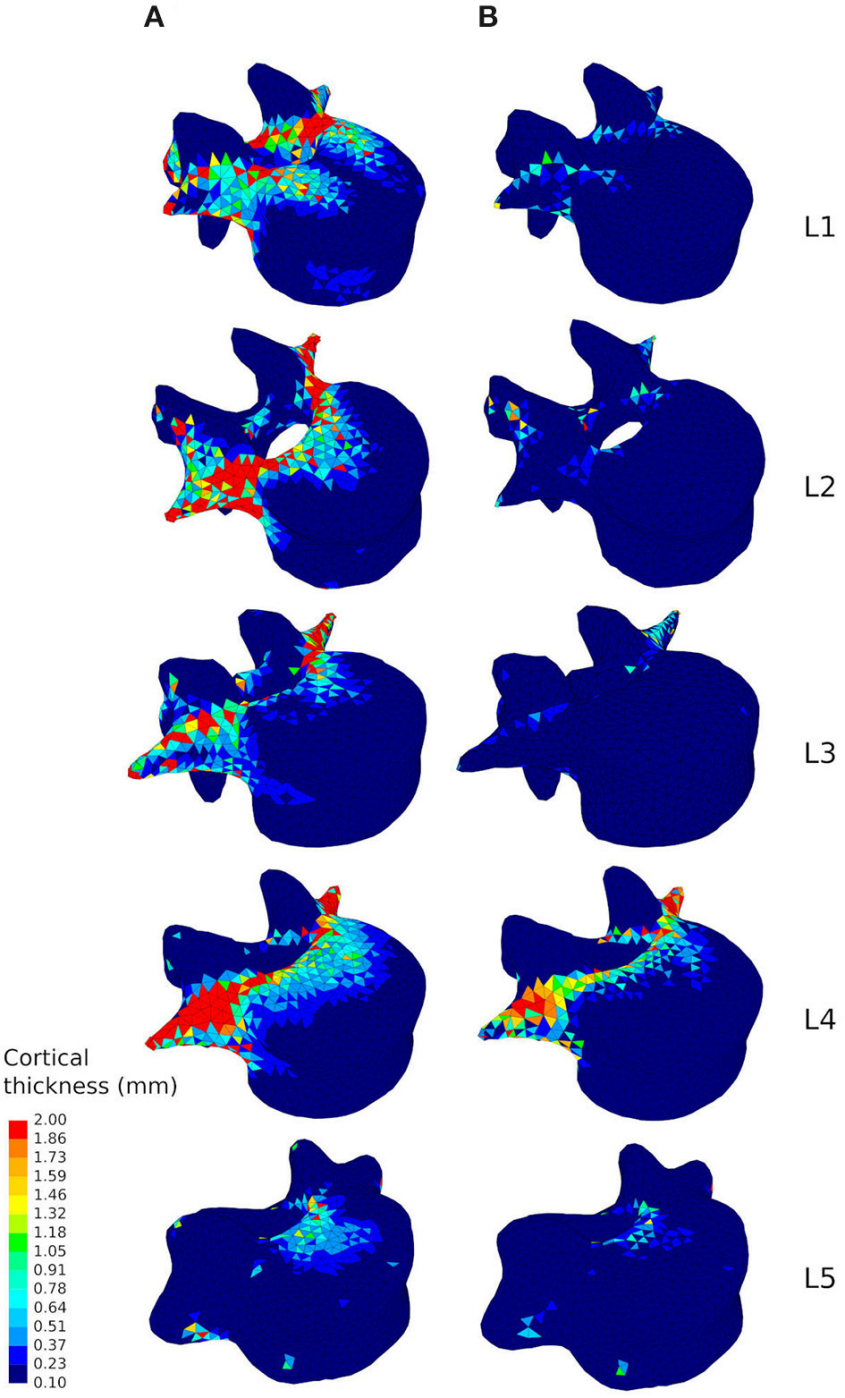
Cortical thickness of the converged mesoscale structural models of the lumbar vertebrae ranging from 0.1 to 2.0 *mm* adapted to the healthy scenario **(A)** and the sedentary scenario **(B)**.

Looking at the cortical thickness in the converged models, a reduced range of activity results in a thinner cortex (Figure 6). In the healthy scenario the thicker shell elements are found in the posterior part of the vertebral body, the pedicles, and the transverse processes (Figure 6A), as these structures have to resist increased muscle forces due to movement of the lumbar spine about the medio-lateral axis during flexion extension activities and about the antero-posterior axis during lateral bending activities. In the sedentary scenario, the thickness of the cortex in these parts of the vertebrae reduces considerably, especially in L1, L2, and L3 (Figure 6B). Detailed views of the cortical thickness after adaptation to the healthy and to the sedentary scenarios can be found in the Supplementary Material for the five lumbar vertebrae.

**Figure 7:**
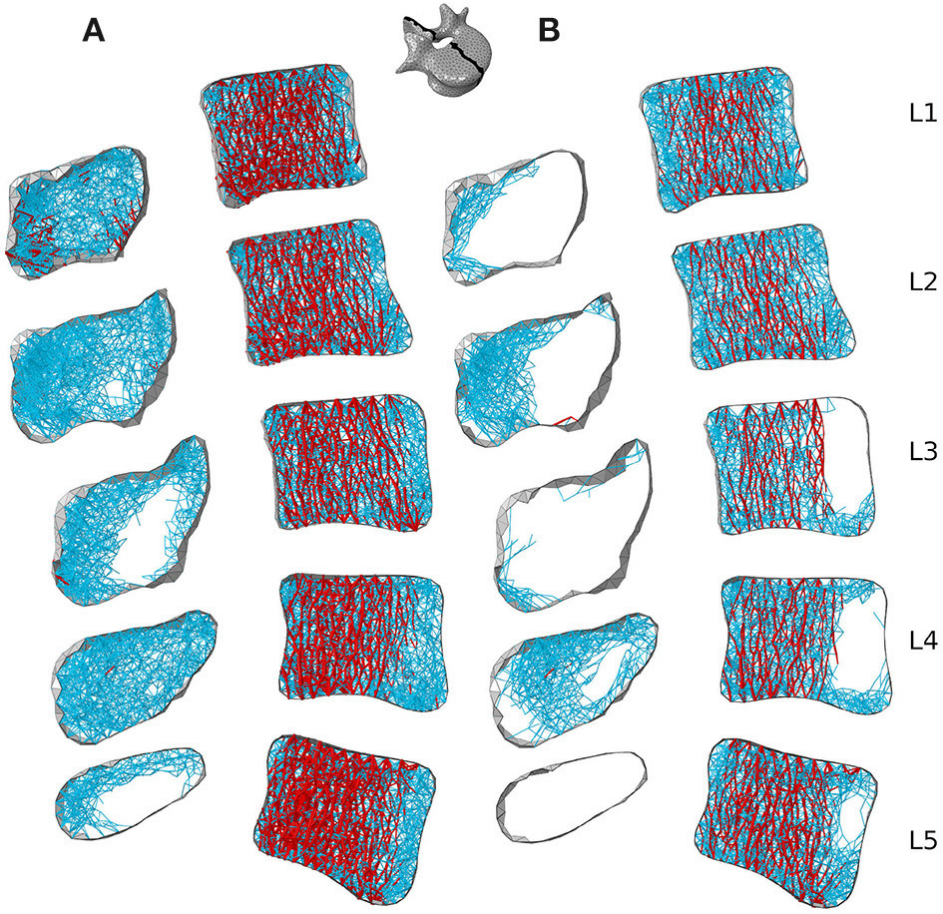
3 *mm* mid-sagittal (XY plane) slices for the converged models adapted to the healthy scenario **(A)** and the sedentary scenario **(B)**. Cortical shell elements are shown in grey. Thicker truss elements representing the primary structure are shown in red. Trabecular truss elements representing the secondary structure (with a radius of 0.1 *mm*) are shown in blue. Truss elements in the dead zone (with a radius of 1 μ*m*) are not shown for clarity.

**Figure 8:**
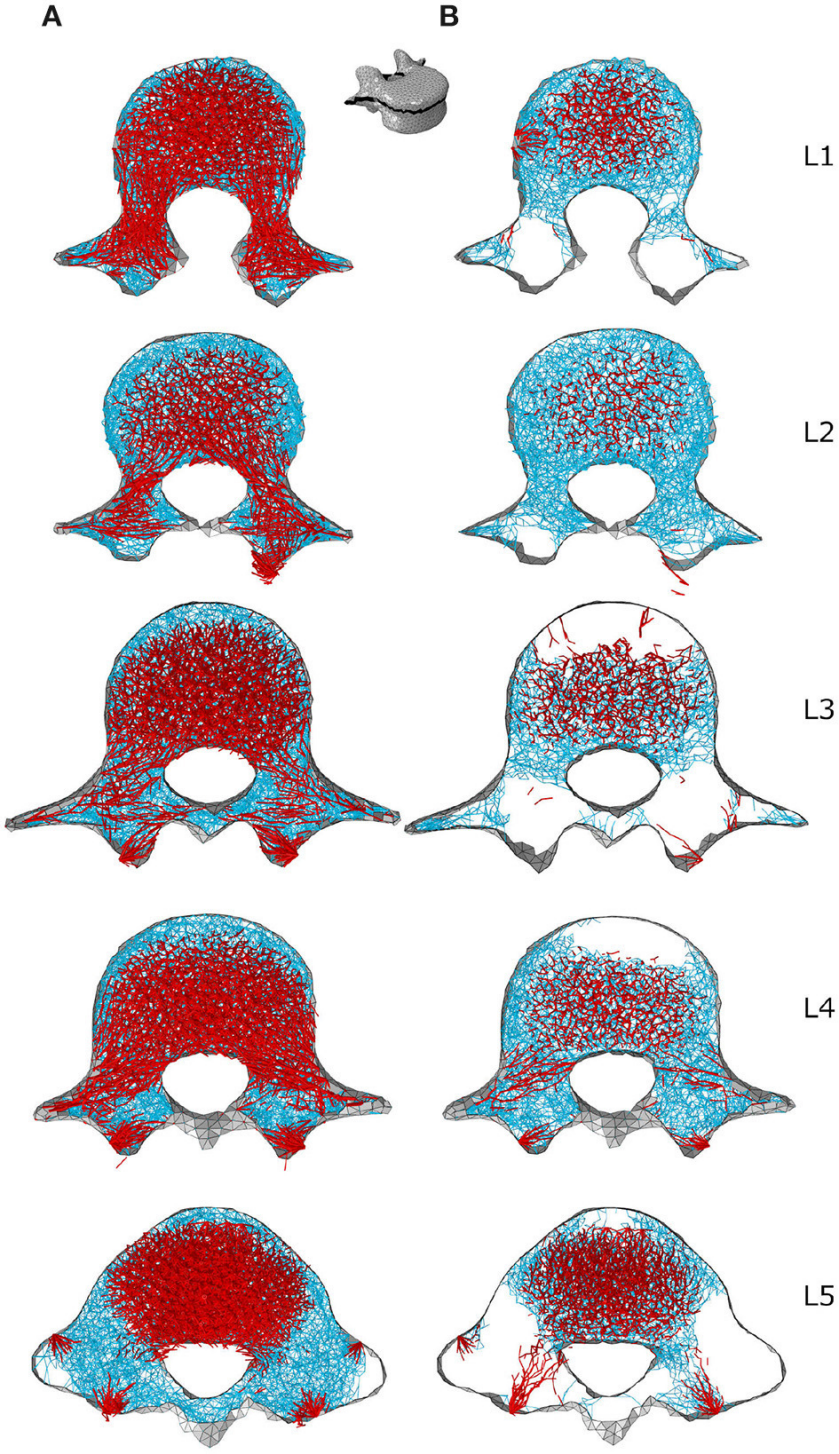
Through-processes transverse (XZ plane) slices for the converged models adapted to the healthy scenario **(A)** and the sedentary scenario **(B)**. In the background, cortical shell elements are shown in grey. Thicker truss elements representing the primary structure located between the superior endplate and the through-processes transverse slice are shown in red. Trabecular truss elements representing the secondary structure (with a radius of 0.1 *mm*) are shown in blue for a 3 *mm* slice. Truss elements in the dead zone (with a radius of 1 μ*m*) are not shown for clarity.

Figures 7B, 8B respectively show mid-sagittal and through-processes transverse slices of the five lumbar vertebrae adapted to the sedentary scenario, highlighting the areas at risk of trabecular bone resorption when demanding activities involving spine movements are not performed, in comparison to the healthy scenario (Figures 7A, 8A). The trabecular bone secondary structure present in the spinous and transverse processes tends to degrade when the vertebrae are subjected to a reduced range of activity (Figures 7B, 8B). L3, L4, and L5 also show this trend in the frontal part of the vertebral body (Figure 7B). For all lumbar vertebrae, the larger trabeculae of the primary structure resisting vertical compression are clustered in the center of the vertebral body (Figure 8B). Apart for L2 and L4 where some of the broader structure remains, the primary structure is missing in the transverse processes for the sedentary scenario (Figure 8B). Detailed slices in the three anatomical planes showing the structure of the five lumbar vertebrae after adaptation to the healthy and to the sedentary scenarios can be found in the Supplementary Material.

### 3.3. Influence of Activities

**Figure 9:**
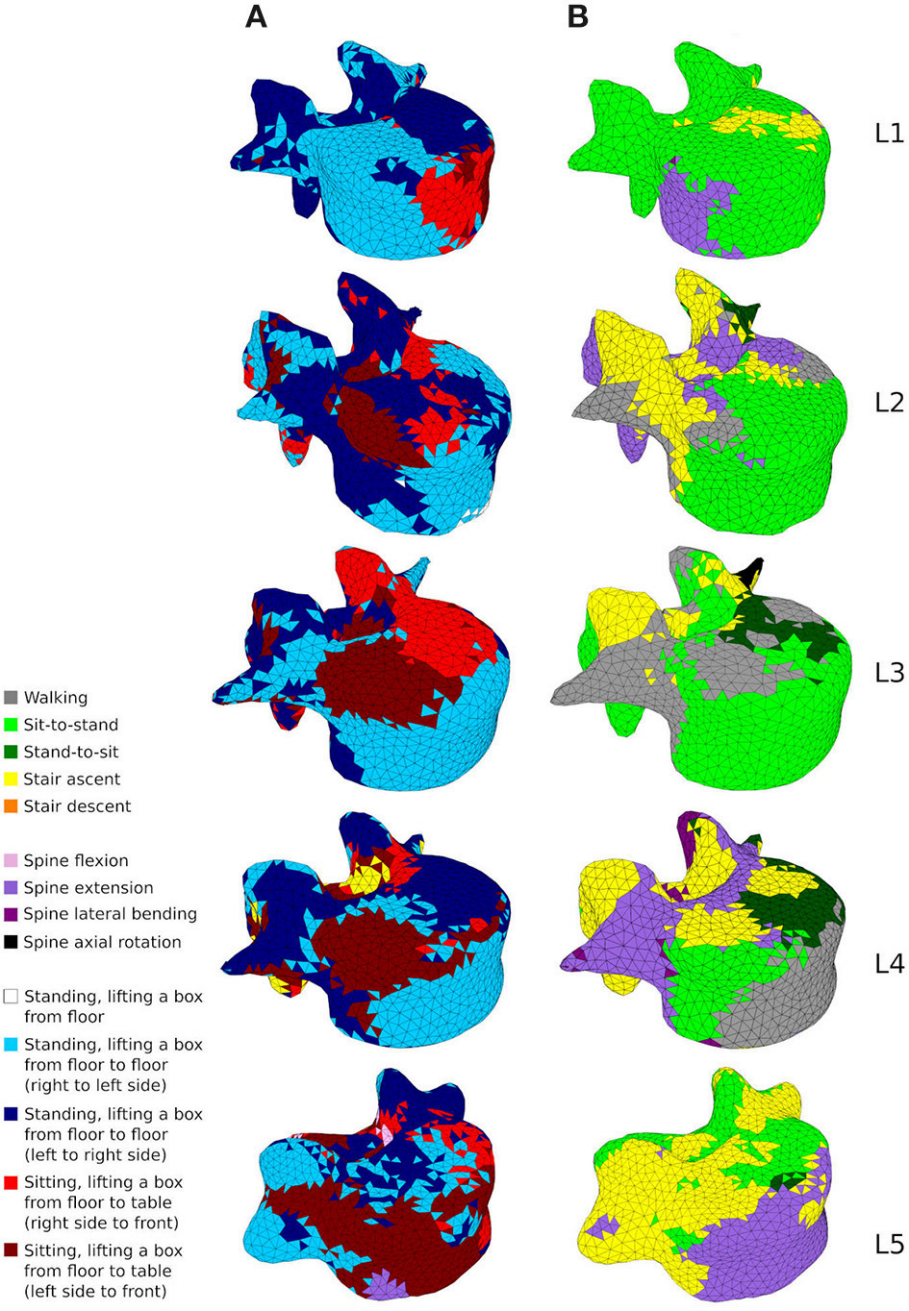
A map showing which activity gives rise to the highest absolute maximum principal strain for the adaptation of the cortical shell elements in the healthy scenario **(A)** and the sedentary scenario **(B)**.

Figure 9 shows how cortical regions of the lumbar vertebrae are influenced by the performed activities. In the healthy scenario (Figure 9A), the most influential activities for cortical adaptation are lifting tasks involving twisting movements of the spine. Lifting a box in the sagittal plane from the floor to the chest while standing has reduced influence on the cortical adaptation. In the sedentary scenario (Figure 9B), the most influential activities are walking, sit-to-stand, stair ascent and spine extension, while stair descent, spine flexion, lateral bending, and axial rotation have reduced influence.

**Figure 10:**
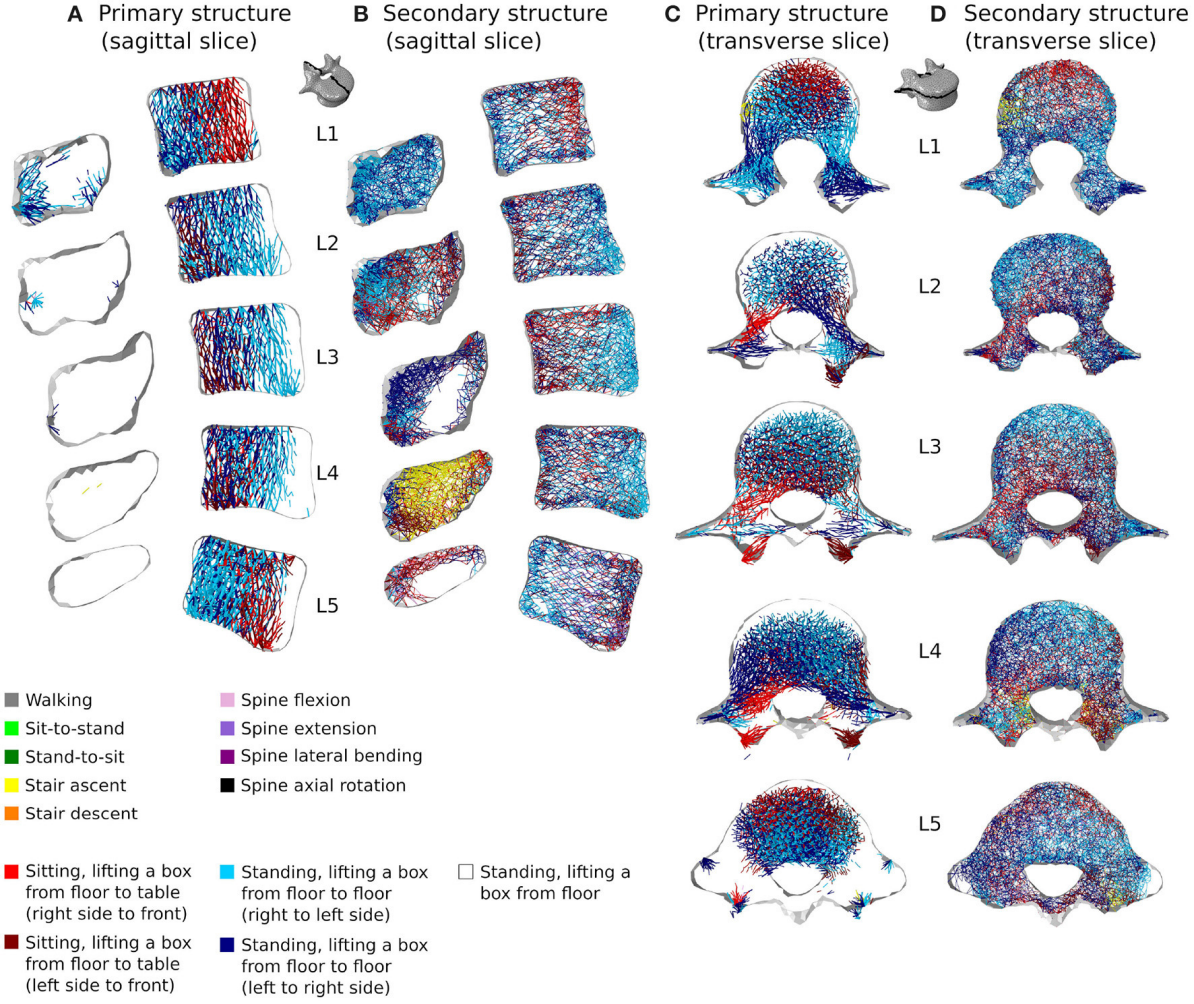
3 *mm* mid-sagittal (XY plane) and through-processes transverse (XZ plane) slices of the converged models in the healthy scenario, showing a map of which activity gives rise to the highest absolute maximum axial strain for the adaptation of the trabecular truss elements of the primary **(A,C)** and secondary **(B,D)** structures. Cortical shell elements are shown in light grey.

**Figure 11:**
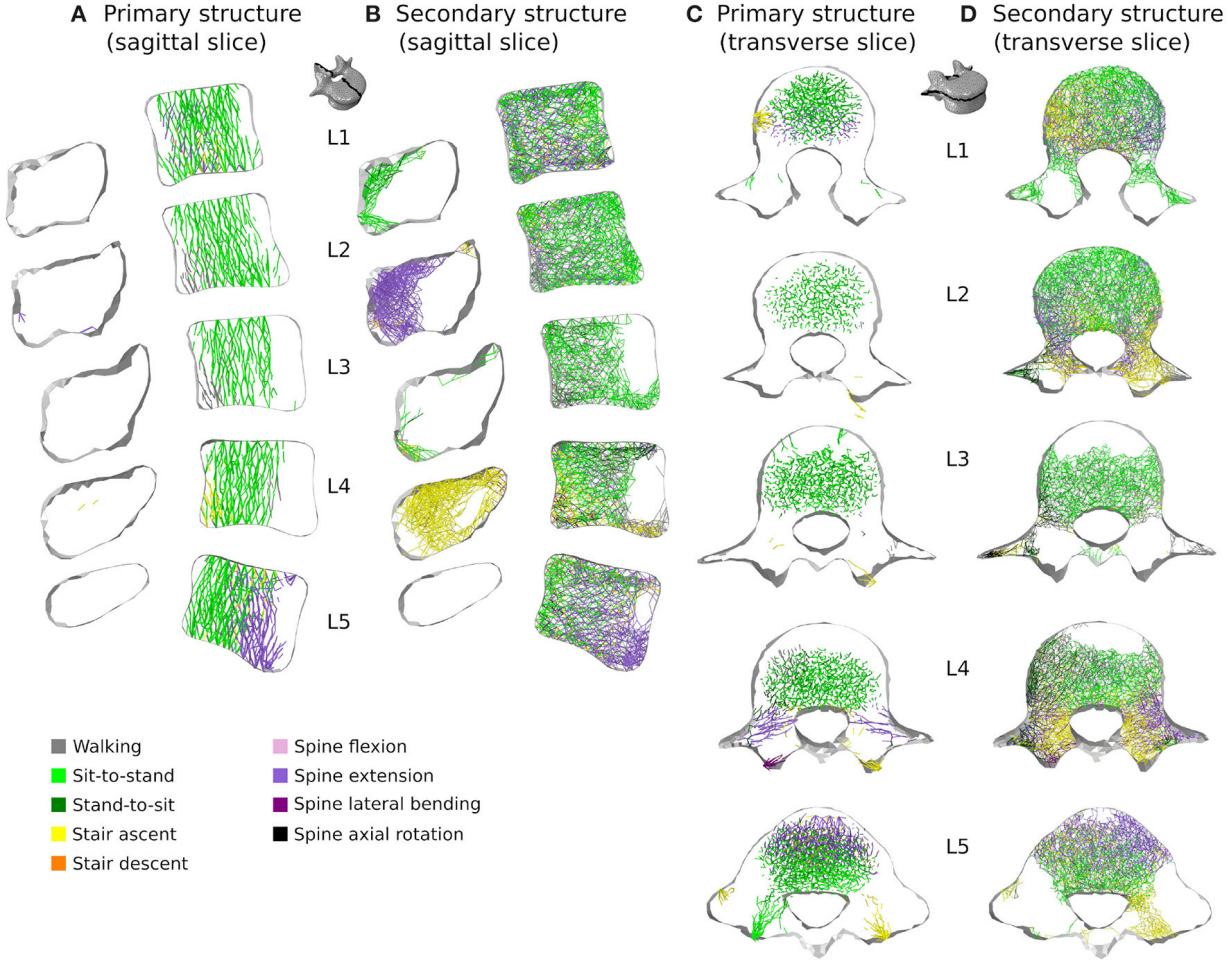
3 *mm* mid-sagittal (XY plane) and through-processes transverse (XZ plane) slices of the converged models in the sedentary scenario, showing a map of which activity gives rise to the highest absolute maximum axial strain for the adaptation of the trabecular truss elements of the primary **(A,C)** and secondary **(B,D)** structures. Cortical shell elements are shown in light grey.

Figures 10, 11 show how trabecular regions of the lumbar vertebrae are influenced by the performed activities in the healthy and sedentary scenarios, respectively. For both scenarios, the primary and secondary structures are influenced by the same activities. Similarly to cortical bone, lifting tasks involving twisting movements of the spine have the most influence on trabecular adaptation in the healthy scenario for truss elements of the primary (Figures 10A,C) and secondary (Figures 10B,D) structures. In the sedentary scenario, the most influential activities on the primary (Figures 11A,C) and secondary (Figures 11B,D) trabecular structures are sit-to-stand, stair ascent, and spine extension.

In the healthy scenario, sagittal slices of the primary (Figure 10A) and secondary (Figure 10B) trabecular structures also show that lifting activities performed in a standing position influence predominantly the posterior part of the vertebral body in L1 and L5 and the anterior part of the vertebral body in L2, L3, and L4, while lifting activities performed in a sitting position stimulate the other part of the vertebral bodies. It is also important to note that even in the sedentary scenario where walking might be expected to be one of the more onerous physical activities, it has reduced influence on the trabecular adaptation of the primary (Figures 11A,C) and secondary (Figures 11B,D) structures.

## 4. Discussion

Combining physiological loading representative of a healthy lifestyle with the bone structural adaptation algorithm produces cortical and trabecular finite element structures of the lumbar vertebrae which compare favourably with *in-vivo* observations. Bone relative density found in the models (Table 2) is within the range reported by Eriksen et al. (2002) and Muller (2004). Cortical thickness in the anterior and posterior walls of the vertebral body (Figure 6A) are in agreement with the range reported by Ritzel et al. (1997) (0.1–0.4 *mm*). In the pedicles, the models show a thicker cortex on the inferior and superior regions, similar to the observations made by Maillot and Wolfram-Gabel (1993). Trajectories of the trabecular elements observed in the models (Figure 5) compare favourably with the observations made by Gallois and Japiot (1925). These comparisons provide an initial positive assessment of the modelling framework.

In addition to producing models of the lumbar vertebrae adapted to a large number of load cases, the modelling approach allows for visualisation of the structural architecture of the vertebrae. The line plots in Figure 5 highlight the dominant trabecular trajectory at each node of the converged finite element model for the L4 vertebra. For the healthy scenario, it confirms that the predicted primary structure of the trabecular bone follows trajectories comparable to the observations of Gallois and Japiot (1925). It also shows that the secondary structure of the trabecular bone (trabecular trusses with a radius of 0.1 *mm*) are mostly aligned medio-laterally throughout the vertebra. The modelling framework also produces a mapping of the vertebrae (Figures 9–11) showing which of the performed activities are the most influential in the adaptation of the structural finite elements. This is a useful approach for understanding which activities are the most beneficial to bone formation in specific regions of the vertebrae. This could be used to inform physical training or rehabilitation treatment based on specific movements and activities. While this modelling framework uses hundreds of load cases to adaptatively produce mesoscale structural models of lumbar vertebrae, its computation cost remains low compared to other mechanical adaptation approaches used in the field. With a total of 119,588 elements, the L4 model converged in 20 iterations for the 116 load cases of the healthy scenario, with each iteration taking around 15 min (finite element analysis and structural adaptation) on a personal workstation (Intel Xeon E5-2630 v2 2.60 *GHz*, 12 CPUs, RAM 64 *GB*). This is computationally efficient compared to the classic microscale continuum modelling approach. For example, in their μCT derived model of L2, Badilatti et al. (2015) used 365 million elements and required 8 hours per iteration on a supercomputer with 1,024 CPUs to adapt the bone to three simplified load cases without any muscle forces. Despite the numerous advantages of the mesoscale structural models, some limitations inherent to the combined modelling approach and to the structural adaptation modelling choices remain and should be acknowledged.

Physiological loading and boundary conditions are essential to provide a realistic mechanical environment for finite element simulations (Bitsakos et al., 2005; Phillips et al., 2007; Speirs et al., 2007; Phillips, 2009). The combined multiscale modelling approach relies on a detailed musculoskeletal model with identical geometry to provide this mechanical environment (Favier et al., 2021). However, assumptions made for the musculoskeletal model will impact the finite element results (Wagner et al., 2010; Cronskaer et al., 2013; Zhu et al., 2013). The idealised representation of intervertebral joints in the musculoskeletal model requires the development of load applicators in the finite element model to spread the reaction force calculated at the joint centre. In particular, the three degree of freedom joint neglects translations permitted by the intervertebral disc, ligaments, and facet joints. While it is possible to improve musculoskeletal models with ligaments (Damm et al., 2019) and a better intervertebral disc representation (Wang et al., 2020), future work will implement the ligaments, discs, and facet joints in a finite element model of multiple spinal units. This is expected to reduce the impact of idealised musculoskeletal joints on the vertebra of interest.

The converged mesoscale structural models show discontinuities in the cortex, with shell thickness varying significantly from one element to the next in some locations. This phenomenon could be addressed in future work to provide a smoother and more realistic thickness variation across the vertebral cortex. The trabecular density in the model is also impacted by the choice of element for the trabeculae. While it has been shown that truss elements ensure a physiological macroscale behaviour of bone (Villette and Phillips, 2015, 2018), local architecture may be improved through using a beam element based bone adaptation with an alternative approach to generating the initial network (Phillips, 2019). Another limitation of the current method is that it does not allow nodes from the initial mesh to realign for better supporting the loading envelope, and future work will focus on allowing structural elements of the trabecular bone to reorient during adaptation. It is also important to note that given the mesoscale nature of the model with radii of up to 2 *mm* being allowed during adaptation, truss element radii in the converged models were expected to exceed the range reported by Rho et al. (1998) and Keaveny et al. (2002) (25–150 μ*m*). However, over 97% of the trabecular elements are within the reported physiological range, with maximum radii of 368 μ*m* found in L5. In all cases, secondary structure elements represent more than 80% of the total number of trabecular elements. Refining the trabecular size categories in the adaptation algorithm and reducing the average length of the structural elements may improve the match between converged models and *in-vivo* observations. An additional limitation, characteristic of the strain-driven adaptation approach, is the choice of values for the target strain, lazy zone, and dead zone in the optimisation algorithm. These values are in agreement with previous studies (Aamodt et al., 1997; Phillips, 2012; Zaharie and Phillips, 2018) and provide reasonable results, but are likely to change depending on age, sex, pathological conditions, and even regions of the skeletal system.

Despite the limitations associated with the current modelling approach, trends can be observed in bone adaptation to different scenarios. In a scenario of around a hundred load cases representing 18 activities typical of a healthy lifestyle, lifting activities involving bending and rotation of the spine were found to be the most influential in stimulating bone (Figures 9A, 10). In a sedentary scenario where the loading conditions were altered to remove any demanding activities involving large spine movements, the remaining trabecular structure is mainly stimulated by sit-to-stand, stair ascent and spine extension activities (Figure 11). The resulting bone architecture (Figures 7B, 8B) is similar to observations made on osteoporotic vertebrae (Jayasinghe et al., 1994). In this scenario, trabeculae tend to disappear completely in the anterior part of the vertebral body and the processes. This can be seen as an extreme degradation of bone and would imply that sedentary behaviours can rapidly lead to bone being unable to support occasional higher loads. However, it should be noted that the current study only predicts a final adapted state, with certain activities removed completely, as opposed to being reduced in daily frequency. For the sedentary scenario, a large amount of trabecular elements fall in the dead zone (Table 2). This is due to the adaptation not including a physiological bone remodelling rate and future developments should consider implementing a remodelling rate between 1,000 and 250 μϵ to obtain a more gradual bone resorption. The results obtained with the current approach should therefore be viewed as a prediction of the regions at risk of bone resorption with sedentary behaviour. Future work comparing the structural architecture obtained in this scenario to vertebra specimens of sedentary or osteoporotic patient populations would provide further validation of the modelling framework and assist in quantifying the extent of this overestimation.

Moderate intensity activities alone are insufficient in providing a mechanical stimulus to prevent bone degradation. This supports the recommendations from the clinical field that an active lifestyle incorporating a wide range of activities is essential to maintain bone health in the lumbar spine. While other physiological factors may influence bone remodelling, activities involving large spine movements in the three anatomical planes and lifting tasks should be performed when possible to maintain lumbar vertebrae bone health. This is particularly relevant for populations subject to physical deconditioning and osteoporosis associated with a sedentary lifestyle (Lau and Guo, 2011), ageing (Guadalupe-Grau et al., 2009; Gomez-Cabello et al., 2012), or chronic low back pain (Weiner et al., 2003; Bjoernsdottir et al., 2012), who carry out these more onerous activities with reduced frequency and may be at risk of bone structural degradation.

## Data Availability Statement

The original contributions presented in the study are included in the article/Supplementary Material, further inquiries can be directed to the corresponding authors.

## Author Contributions

CF designed the study, developed the protocol for collecting motion capture and MRI data and acquired these data, carried out all modelling, and drafted the manuscript. AP and AM conceived of, designed and coordinated the study, and drafted the manuscript. All authors contributed to the article and approved the submitted version.

## Conflict of Interest

The authors declare that the research was conducted in the absence of any commercial or financial relationships that could be construed as a potential conflict of interest.
