# Supplementary material for: Maintaining Bone Health in the Lumbar Spine: Routine Activities Alone Are Not Enough
Source: Front Bioeng Biotechnol. 2021 May 19;9:661837. doi: 10.3389/fbioe.2021.661837 (PMC8170092; doi:10.3389/fbioe.2021.661837)
Supplement: Supplementary file 1 [file Data_Sheet_1.pdf]

Supplementary Material

Maintaining bone health in the lumbar spine: routine activities alone are not enough.

Clément D. Favier[1], Alison H. McGregor[2] and Andrew T. M. Phillips[1]

[1]Structural Biomechanics, Department of Civil and Environmental Engineering, Imperial College London, London, United Kingdom
[2]Musculoskeletal Lab, Department of Surgery and Cancer, Imperial College London, London, United Kingdom

DOI: 10.3389/fbioe.2021.661837

Correspondence:

Clément D. Favier
clement.favier13@imperial.ac.uk

Andrew T. M. Phillips
andrew.phillips@imperial.ac.uk

1 DIGITAL FILES

1.1 Converged models

Converged structural finite element models of the five lumbar vertebrae adapted to the healthy scenario are available as Abaqus input files at http://dx.doi.org/10.6084/m9.figshare.14355794.

1.2 Anisotropy plots

3D plots characterising anisotropy in the five lumbar vertebrae adapted to the healthy scenario are available as MATLAB figure files at http://dx.doi.org/10.6084/m9.figshare.14356001.

2 FIRST LUMBAR VERTEBRA

2.1 Load cases

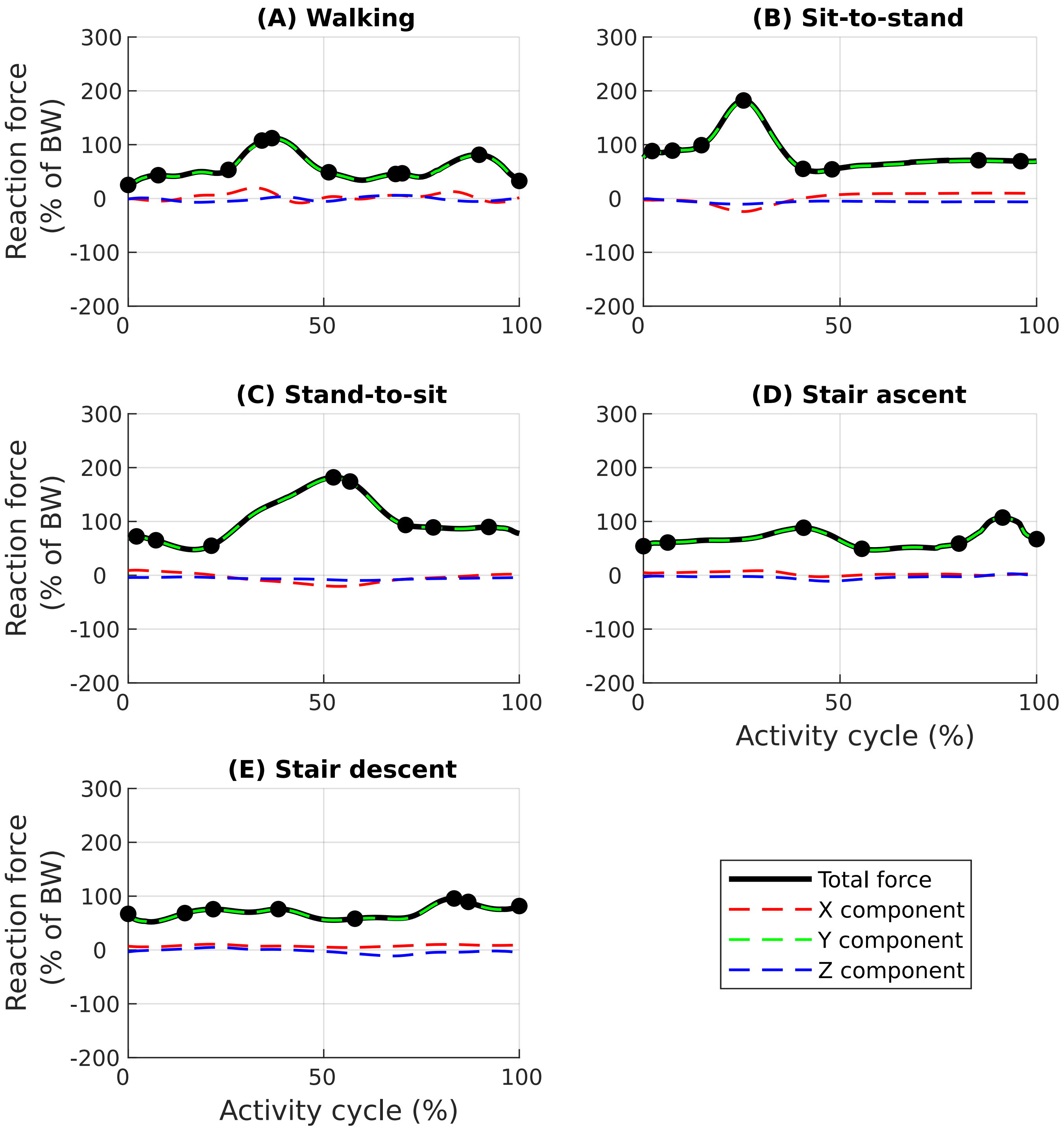

Figure S1: Selection of load cases for L1, for five activities related to locomotion ((A) level walking, (B) standing up from a chair, (C) sitting down on a chair, (D) walking up the stairs, and (E) walking down the stairs). Total reaction force at L1-L2 joint derived from the musculoskeletal model and normalised to body weight (BW) are shown as black lines. Dots indicate the frames selected for the finite element analysis. Red, green and blue dashed lines show X, Y and Z components of the reaction force expressed in the vertebra coordinate system.

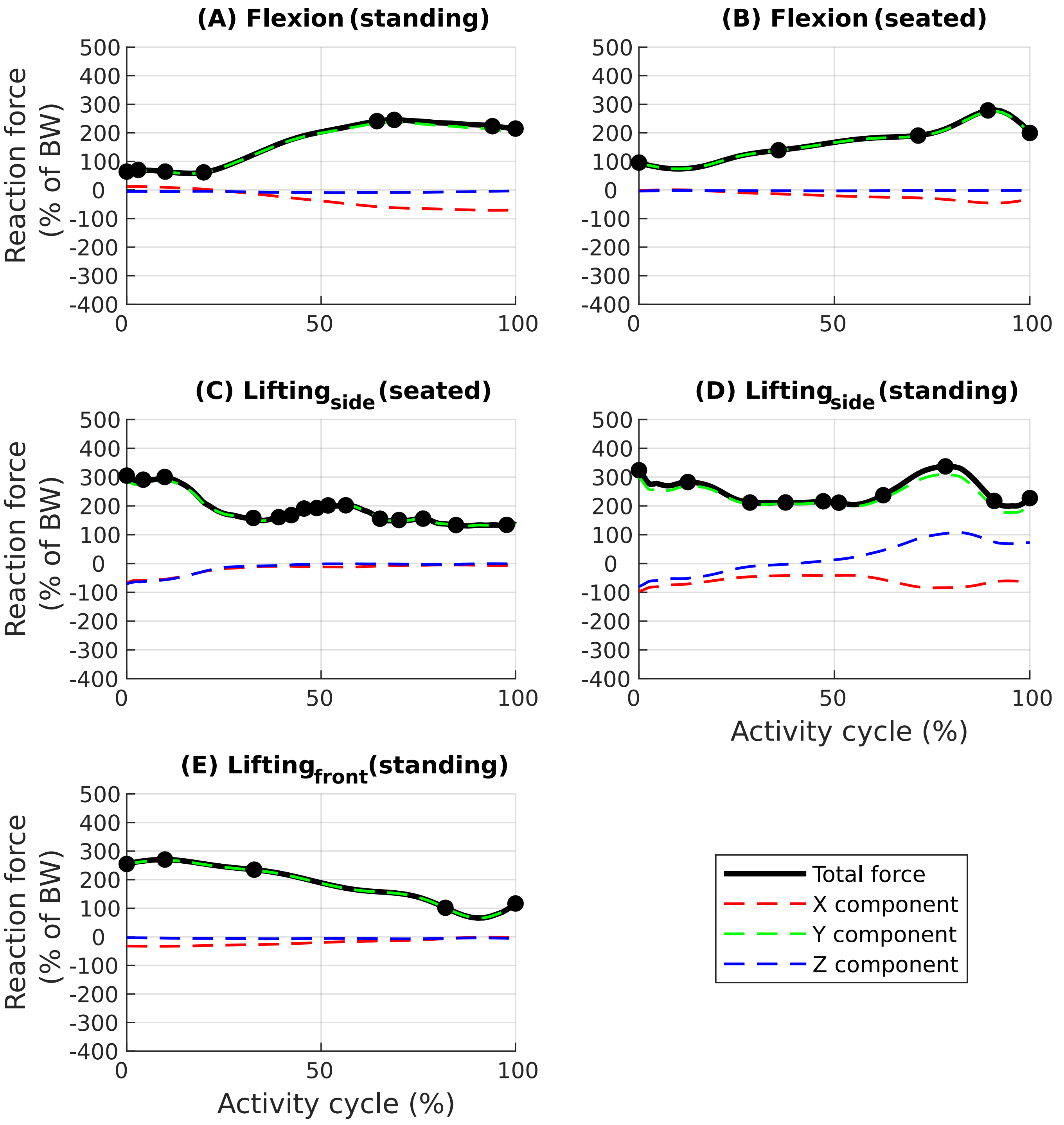

Figure S2: Selection of load cases for L1, for seven activities involving spine movements ((A) forward flexion from upright standing to maximum flexion, (B) forward flexion from upright sitting, (C) in a seated position, twisting and lifting a box from the floor, from the right side to a table in front, (D) in a standing position, twisting and lifting a box from the floor, from the left side to the right side, and (E) in a standing position, lifting a box from the floor in front to the chest). Total reaction force at L1-L2 joint derived from the musculoskeletal model and normalised to body weight (BW) are shown as black lines. Dots indicate the frames selected for the finite element analysis. Load cases obtained with activities (C) and (D) were mirrored through the sagittal plane to obtain loading on the other side. Red, green and blue dashed lines show X, Y and Z components of the reaction force expressed in the vertebra coordinate system.

2.2 Healthy scenario

2.2.1 Cortical thickness

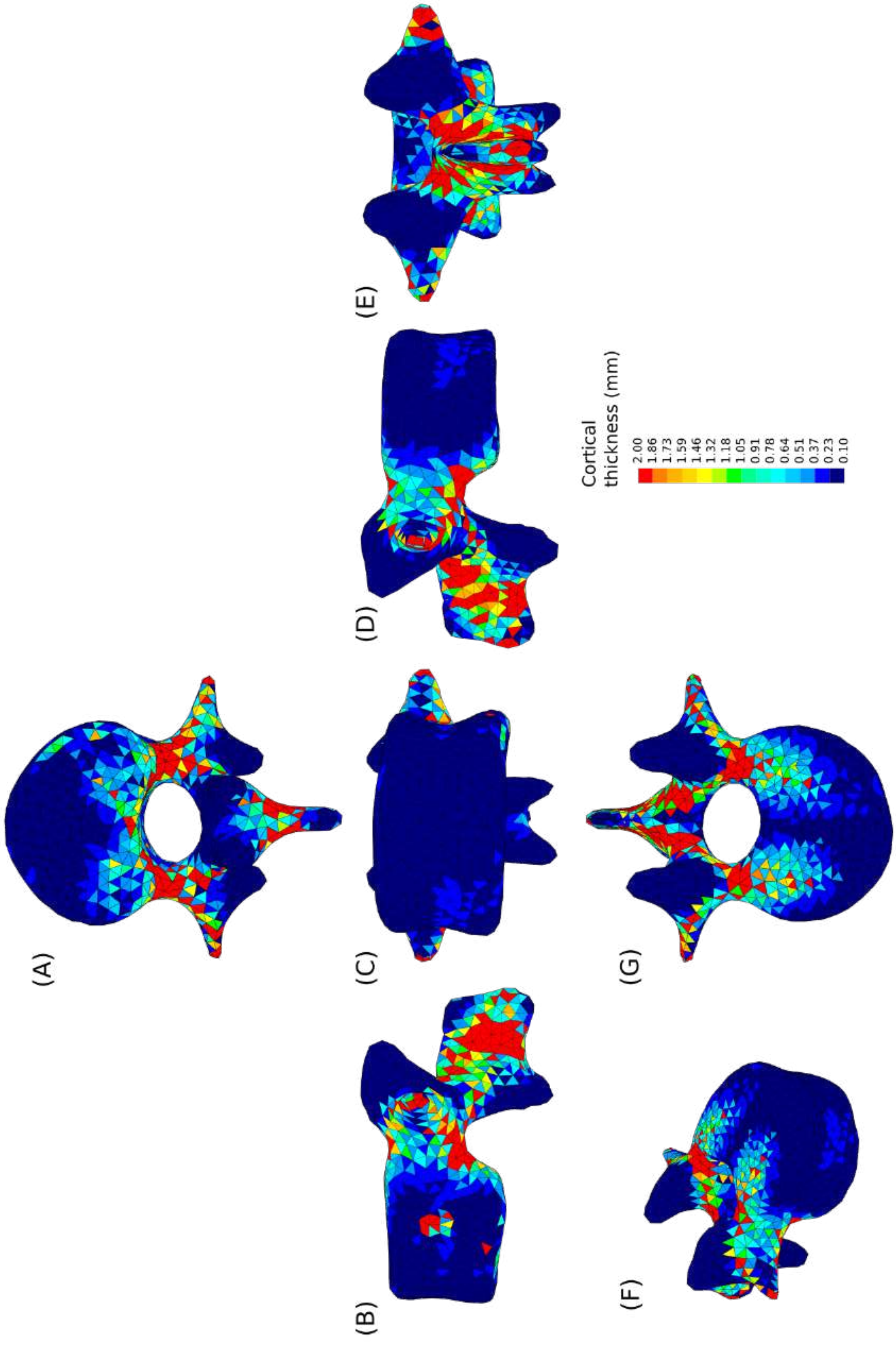

Figure S3: Cortical thickness ranging from 0.1 to 2 *mm* for the converged L1 model adapted to the healthy scenario. (A) caudal view, (B) left lateral view, (C) frontal view, (D) right lateral view, (E) dorsal view, (F) isometric view, (G) cranial view.

2.2.2 Trabecular architecture

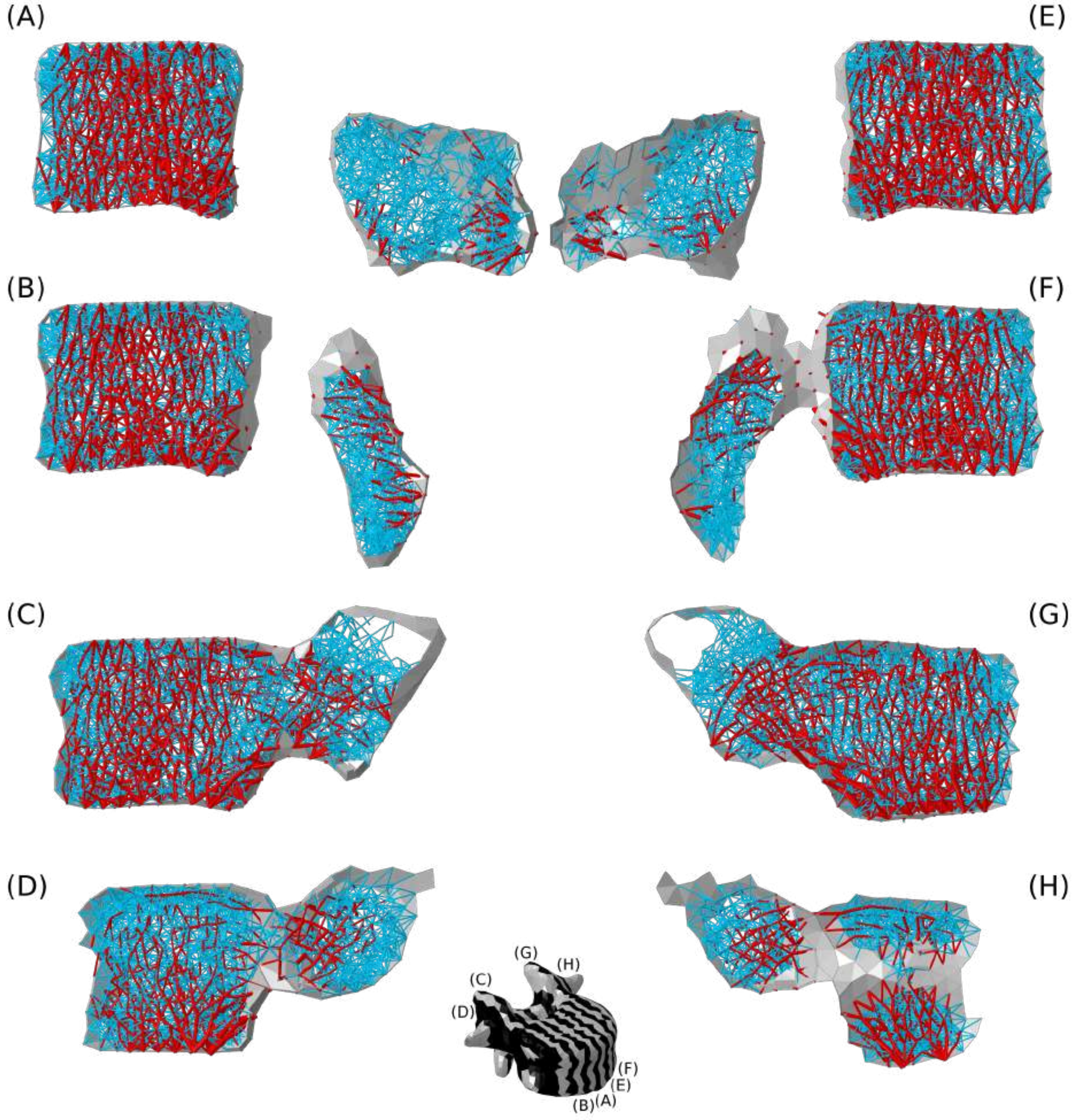

Figure S4: Selected sagittal 3 mm slices for the converged L1 model adapted to the healthy scenario. Cortical shell elements are shown in grey. Trabecular truss elements representing the secondary structure (with a radius of 0.1 mm) are shown in blue. Thicker truss elements representing the primary structure are shown in red. Truss elements in the dead zone (with a radius of 1 μm) are not shown for clarity.

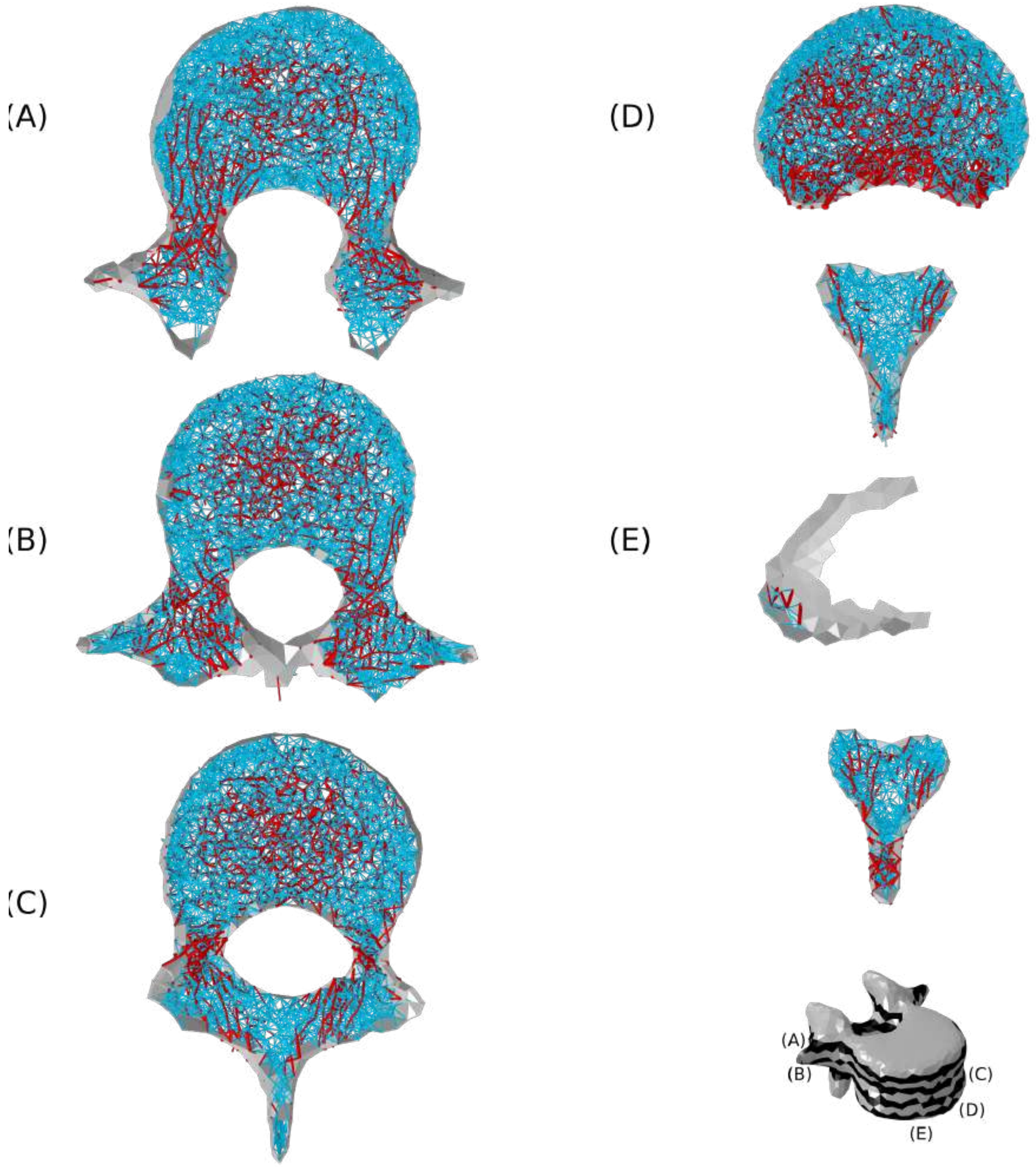

Figure S5: Selected transverse 3 mm slices for the converged L1 model adapted to the healthy scenario. Cortical shell elements are shown in grey. Trabecular truss elements representing the secondary structure (with a radius of 0.1 mm) are shown in blue. Thicker truss elements representing the primary structure are shown in red. Truss elements in the dead zone (with a radius of 1 μm) are not shown for clarity.

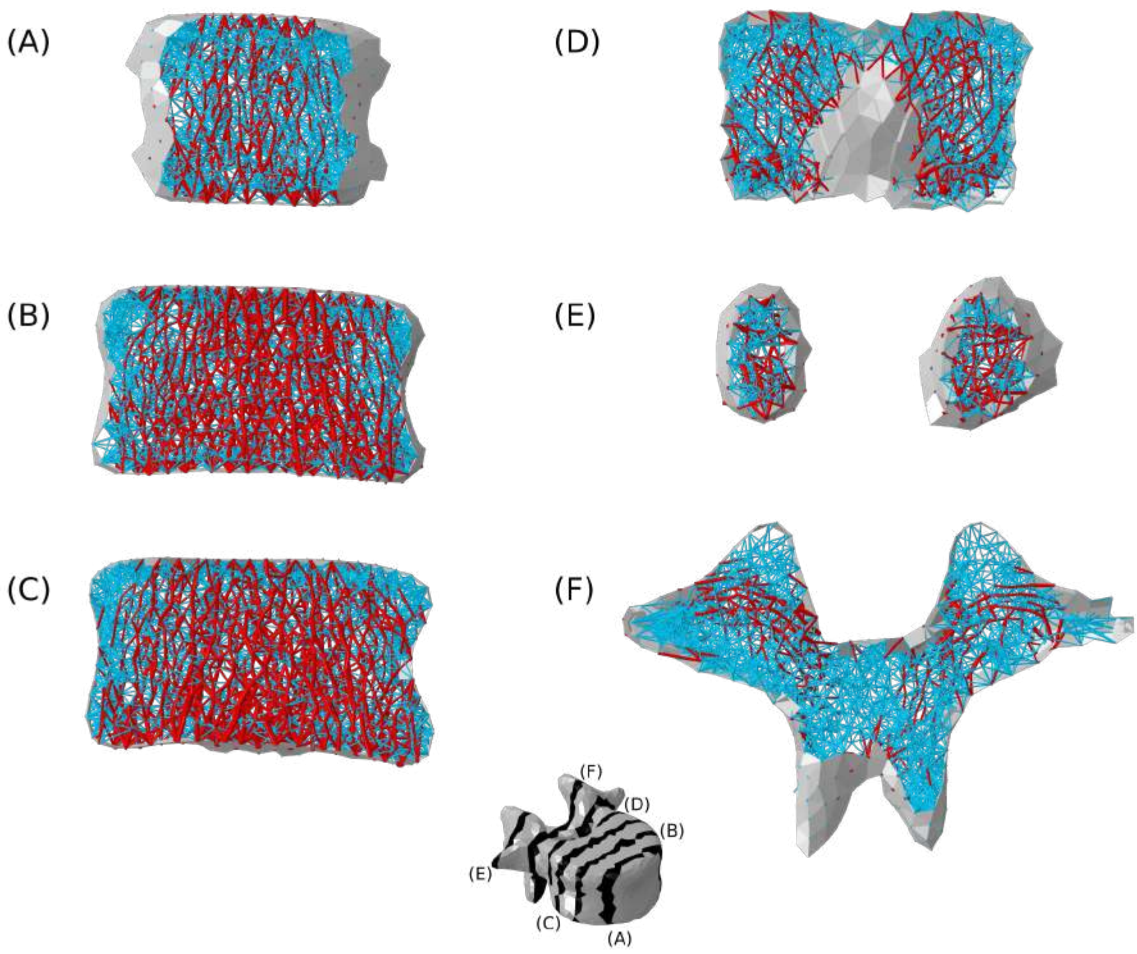

Figure S6: Selected coronal 3 mm slices for the converged L1 model adapted to the healthy scenario. Cortical shell elements are shown in grey. Trabecular truss elements representing the secondary structure (with a radius of 0.1 mm) are shown in blue. Thicker truss elements representing the primary structure are shown in red. Truss elements in the dead zone (with a radius of 1 μm) are not shown for clarity.

2.2.3 Contribution of each activities

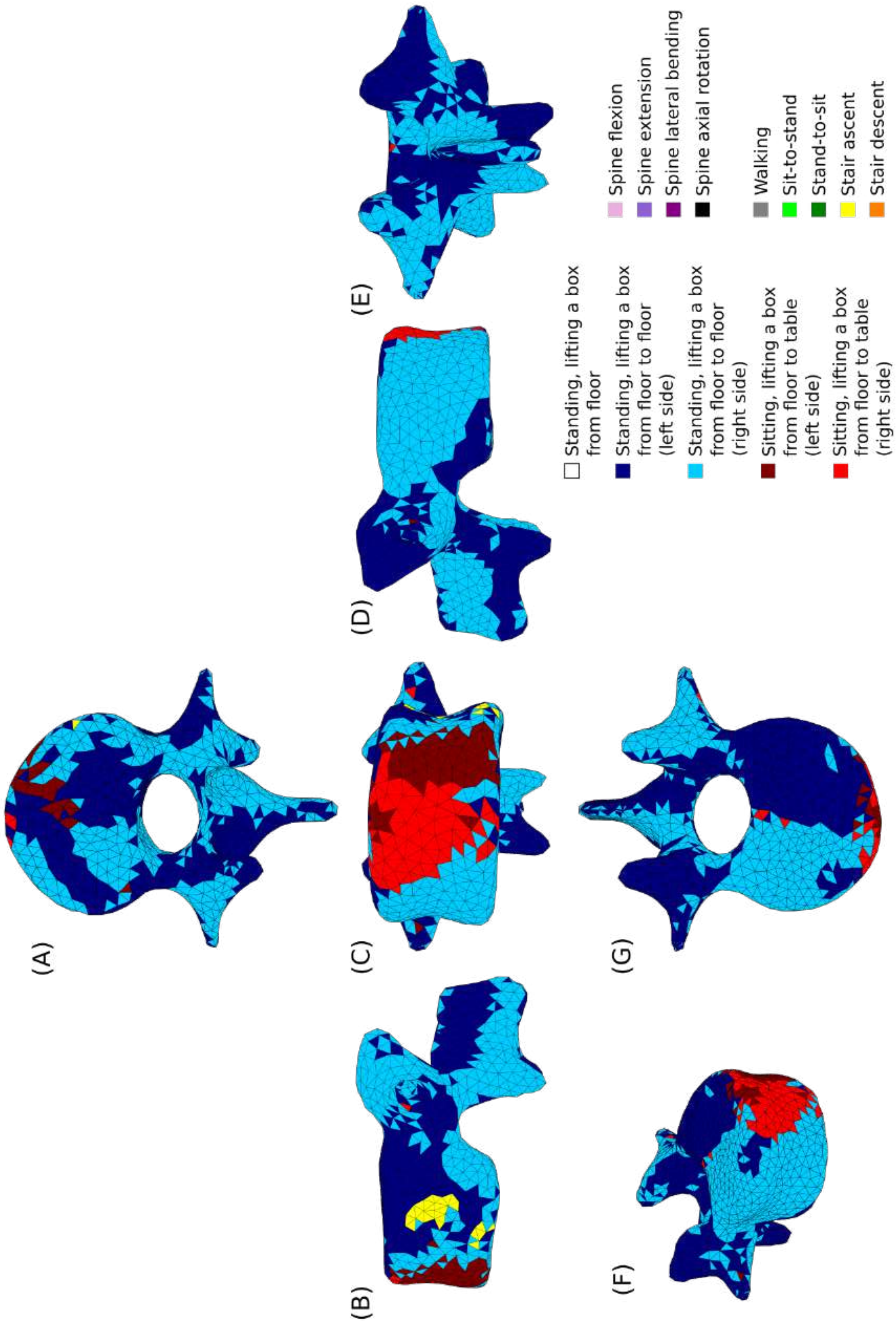

Figure S7: Contribution of each activity to the adaptation of the L1 cortical shells for the healthy scenario. (A) caudal view, (B) left lateral view, (C) frontal view, (D) right lateral view, (E) dorsal view, (F) isometric view, (G) cranial view.

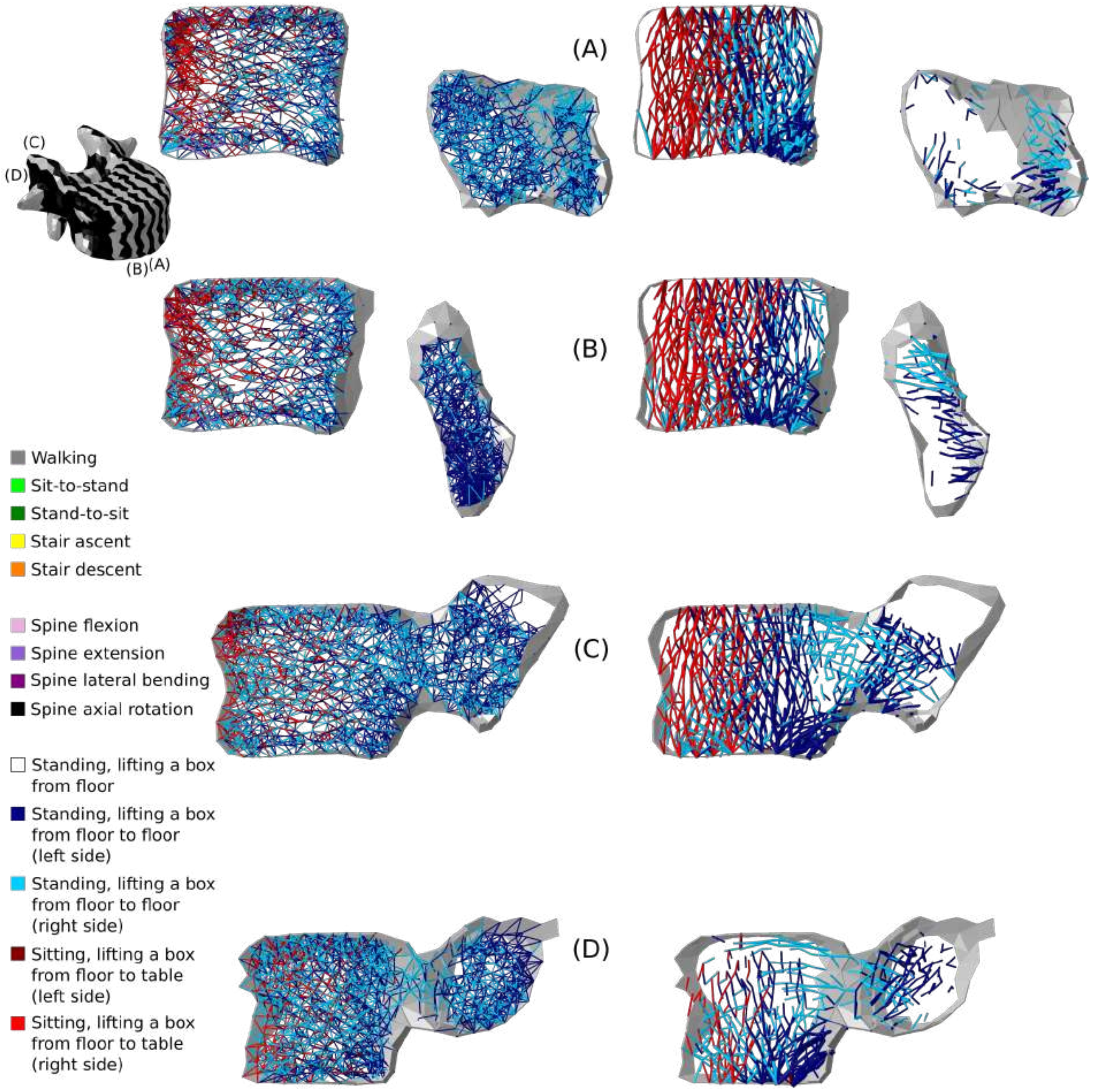

Figure S8: Selection of 3 *mm* sagittal slices of the right side of the converged L1 model adapted to the healthy scenario. Trabecular truss elements are colour mapped based on the activity most influential to their final geometry. On the left, only trabecular truss elements representing the secondary structure (with a radius of 0.1 *mm*) are shown. On the right, only trabecular truss elements representing the primary structure (with a radius superior to 0.1 *mm*) are shown. Cortical shell elements are sown in light grey.

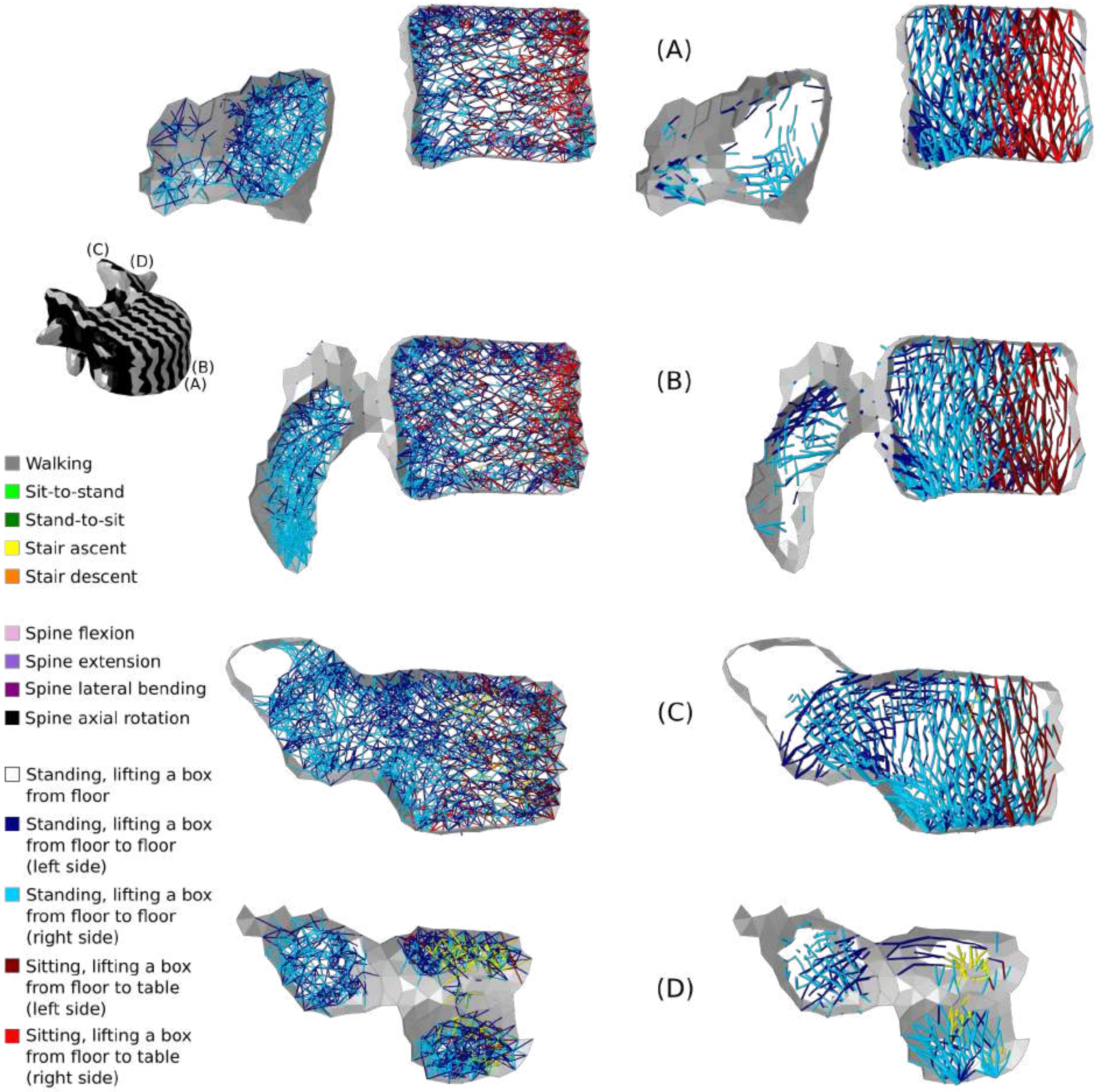

Figure S9: Selection of 3 mm sagittal slices of the left side of the converged L1 model adapted to the healthy scenario. Trabecular truss elements are colour mapped based on the activity most influential to their final geometry. On the left, only trabecular truss elements representing the secondary structure (with a radius of 0.1 mm) are shown. On the right, only trabecular truss elements representing the primary structure (with a radius superior to 0.1 mm) are shown. Cortical shell elements are sown in light grey.

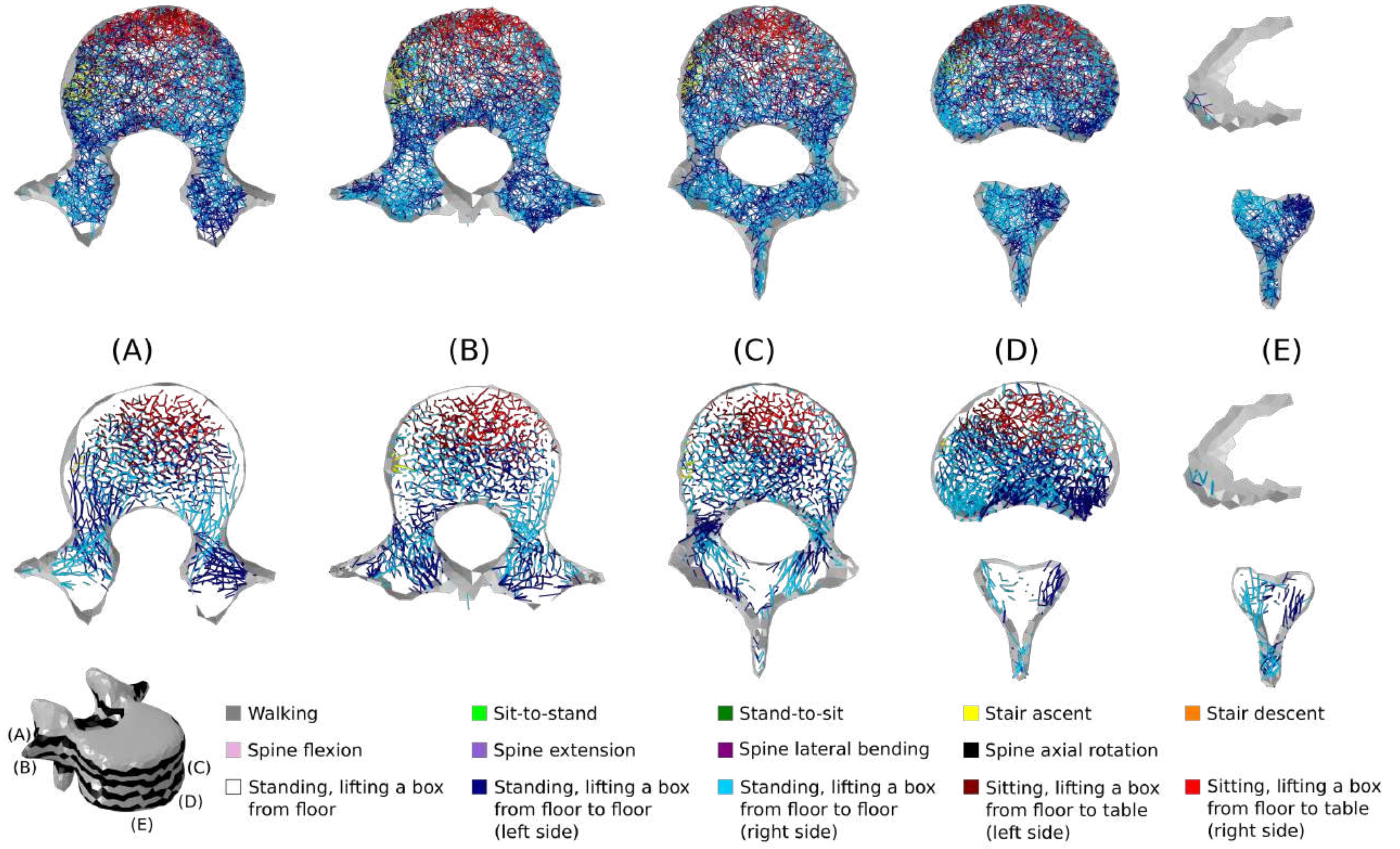

Figure S10: Selection of 3 mm transverse slices of the converged L1 model adapted to the healthy scenario. Trabecular truss elements are colour mapped based on the activity most influential to their final geometry. On the top row, only trabecular truss elements representing the secondary structure (with a radius of 0.1 mm) are shown. On the bottom row, only trabecular truss elements with a radius superior to 0.1 mm are shown. Cortical shell elements are sown in light grey.

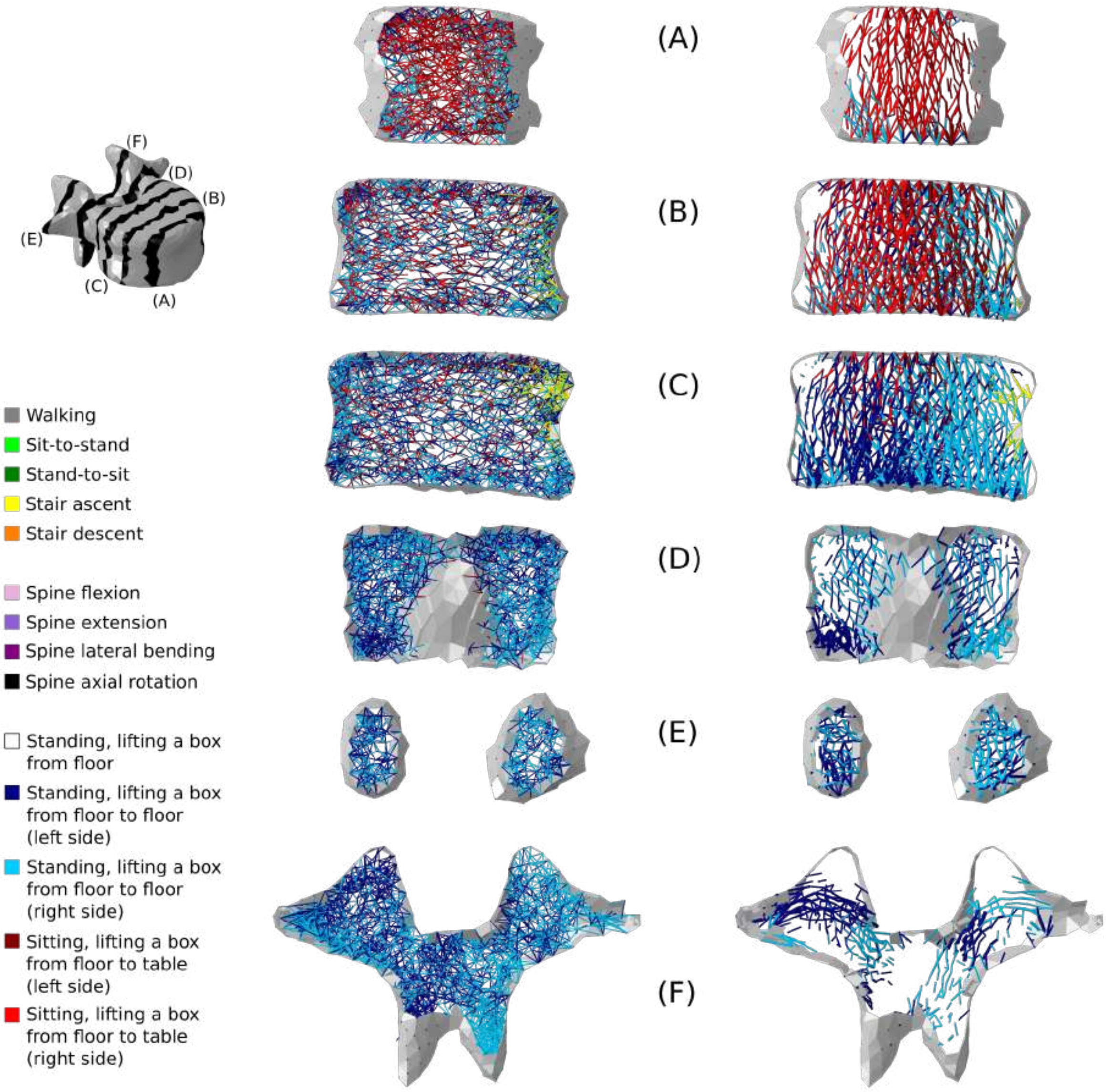

Figure S11: Selection of 3 mm coronal slices of the converged L1 model adapted to the healthy scenario. Trabecular truss elements are colour mapped based on the activity most influential to their final geometry. On the left, only trabecular truss elements representing the secondary structure (with a radius of 0.1 mm) are shown. On the right, only trabecular truss elements representing the primary structure (with a radius superior to 0.1 mm) are shown. Cortical shell elements are sown in light grey.

2.2.4 Characterisation of trabecular anisotropy

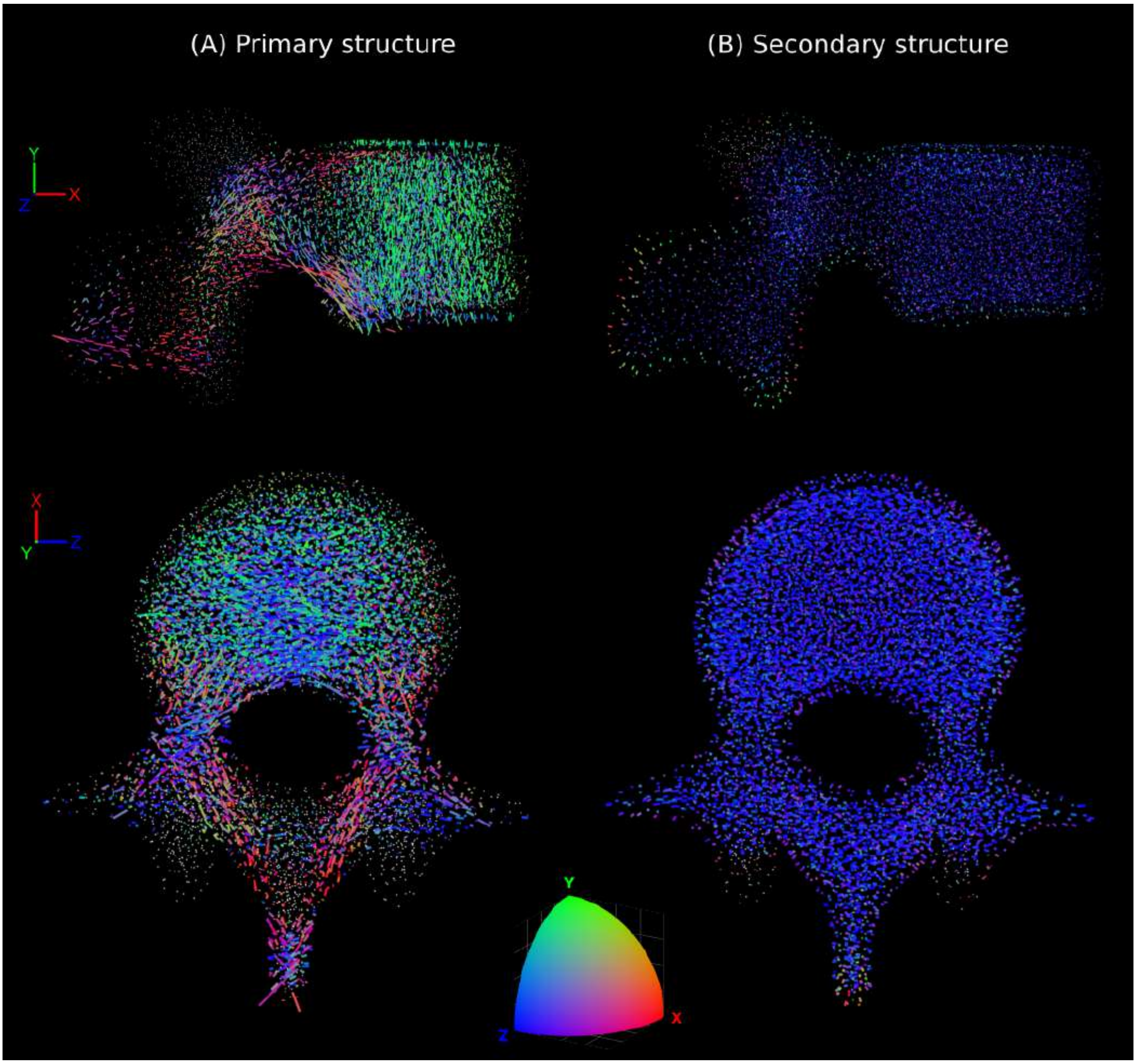

Figure S12: Characterisation of anisotropy in the trabecular bone of L1 adapted to the healthy scenario. (A) shows the orientation of the trabecular trusses of the primary structure (with a radius larger than 0.1 mm). (B) shows the orientation of the trabecular trusses of the secondary structure (with a radius of 0.1 mm). For (A) and (B), side view (top row) and top view (bottom row) are shown. Lines are attached to each trabecular node, with colour and length varying respectively with the orientations and radii of the truss elements connected to that particular node. The colour scale at the bottom shows how the colour of the lines should be interpreted. Orientation along the X, Y or Z axes are in red, green or blue respectively. Any orientation that is not colinear with these axes shows as a combination of red, green and blue. A white dot indicates a node without elements in the size range being looked at connected to it.

2.3 Sedentary scenario

2.3.1 Cortical thickness

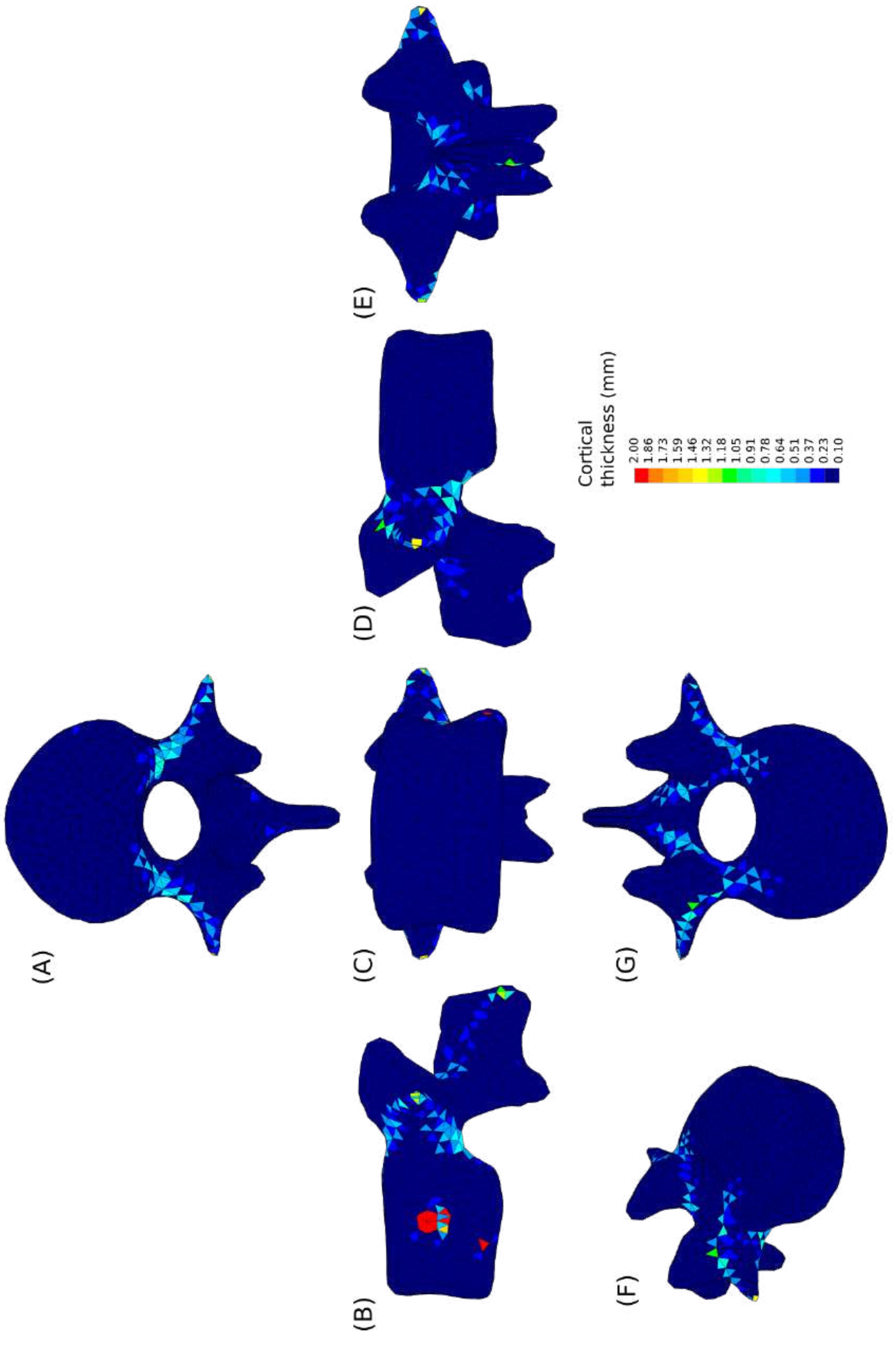

Figure S13: Cortical thickness ranging from 0.1 to 2 mm for the converged L1 model adapted to the sedentary scenario. (A) caudal view, (B) left lateral view, (C) frontal view, (D) right lateral view, (E) dorsal view, (F) isometric view, (G) cranial view.

2.3.2 Trabecular architecture

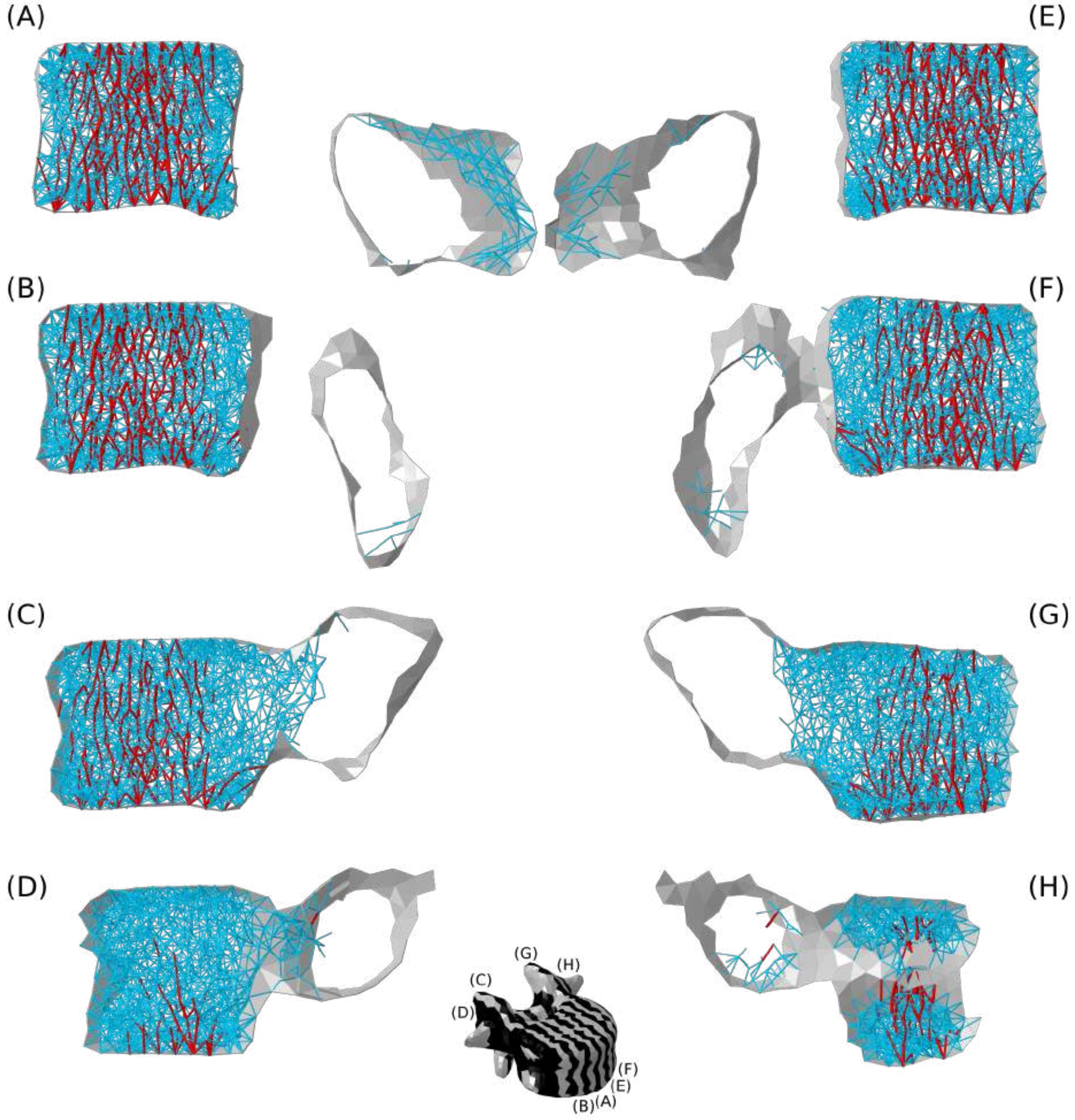

Figure S14: Selected sagittal 3 mm slices for the converged L1 model adapted to the sedentary scenario. Cortical shell elements are shown in grey. Trabecular truss elements representing the secondary structure (with a radius of 0.1 mm) are shown in blue. Thicker truss elements representing the primary structure are shown in red. Truss elements in the dead zone (with a radius of 1 μm) are not shown for clarity.

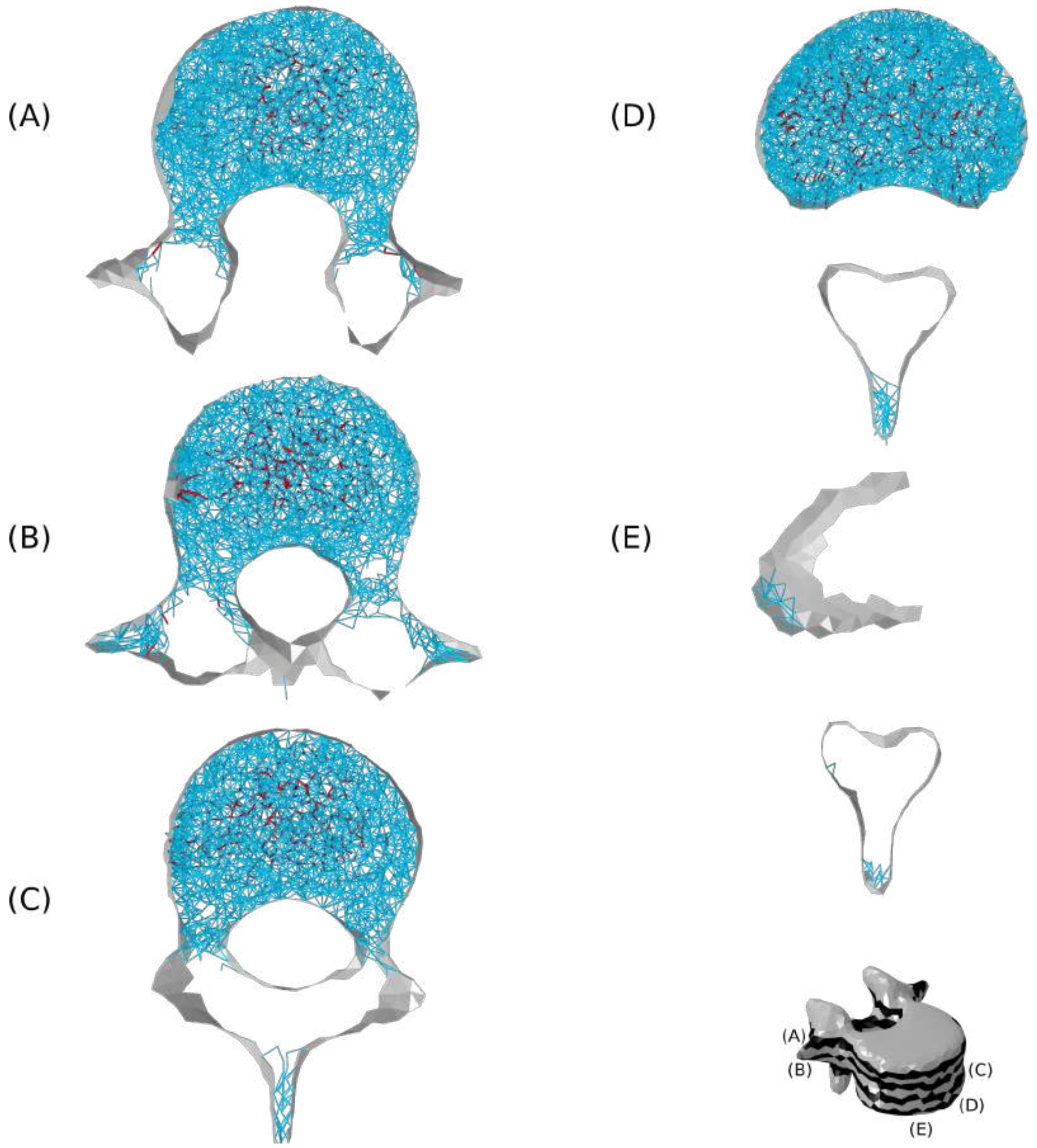

Figure S15: Selected transverse 3 mm slices for the converged L1 model adapted to the sedentary scenario. Cortical shell elements are shown in grey. Trabecular truss elements representing the secondary structure (with a radius of 0.1 mm) are shown in blue. Thicker truss elements representing the primary structure are shown in red. Truss elements in the dead zone (with a radius of 1 μm) are not shown for clarity.

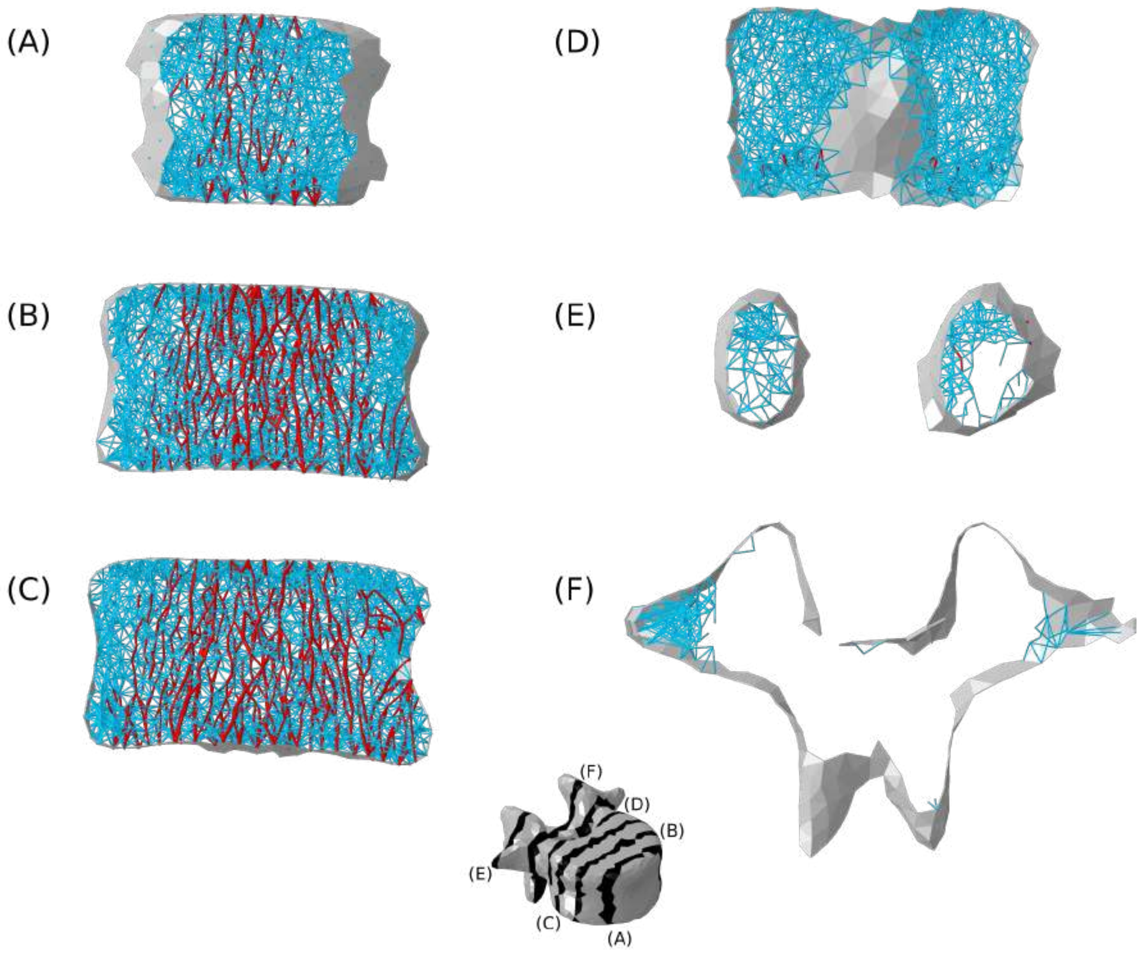

Figure S16: Selected coronal 3 *mm* slices for the converged L1 model adapted to the sedentary scenario. Cortical shell elements are shown in grey. Trabecular truss elements representing the secondary structure (with a radius of 0.1 *mm*) are shown in blue. Thicker truss elements representing the primary structure are shown in red. Truss elements in the dead zone (with a radius of 1 μm) are not shown for clarity.

2.3.3 Contribution of each activities

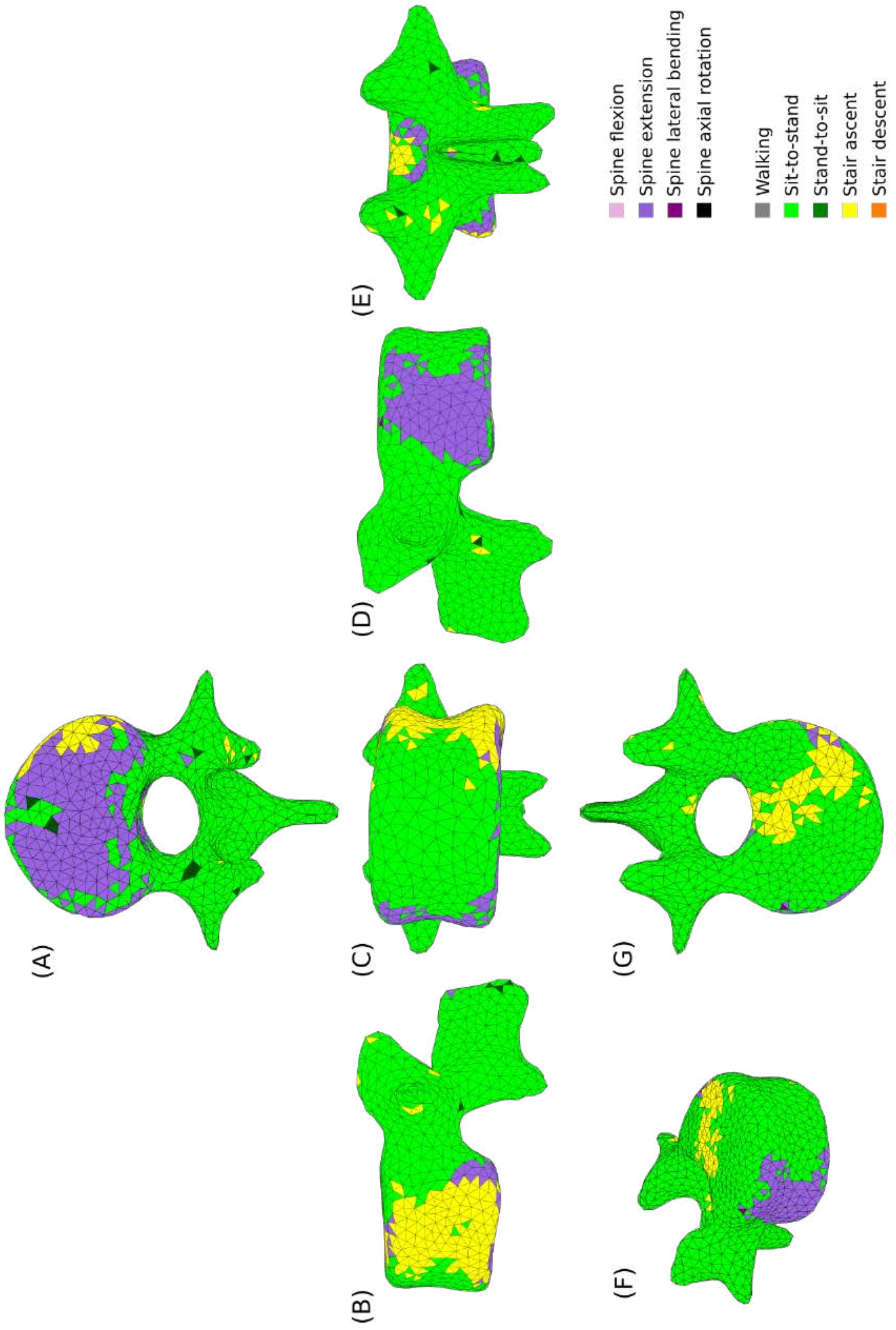

Figure S17: Contribution of each activity to the adaptation of the L1 cortical shells for the sedentary scenario. (A) caudal view, (B) left lateral view, (C) frontal view, (D) right lateral view, (E) dorsal view, (F) isometric view, (G) cranial view.

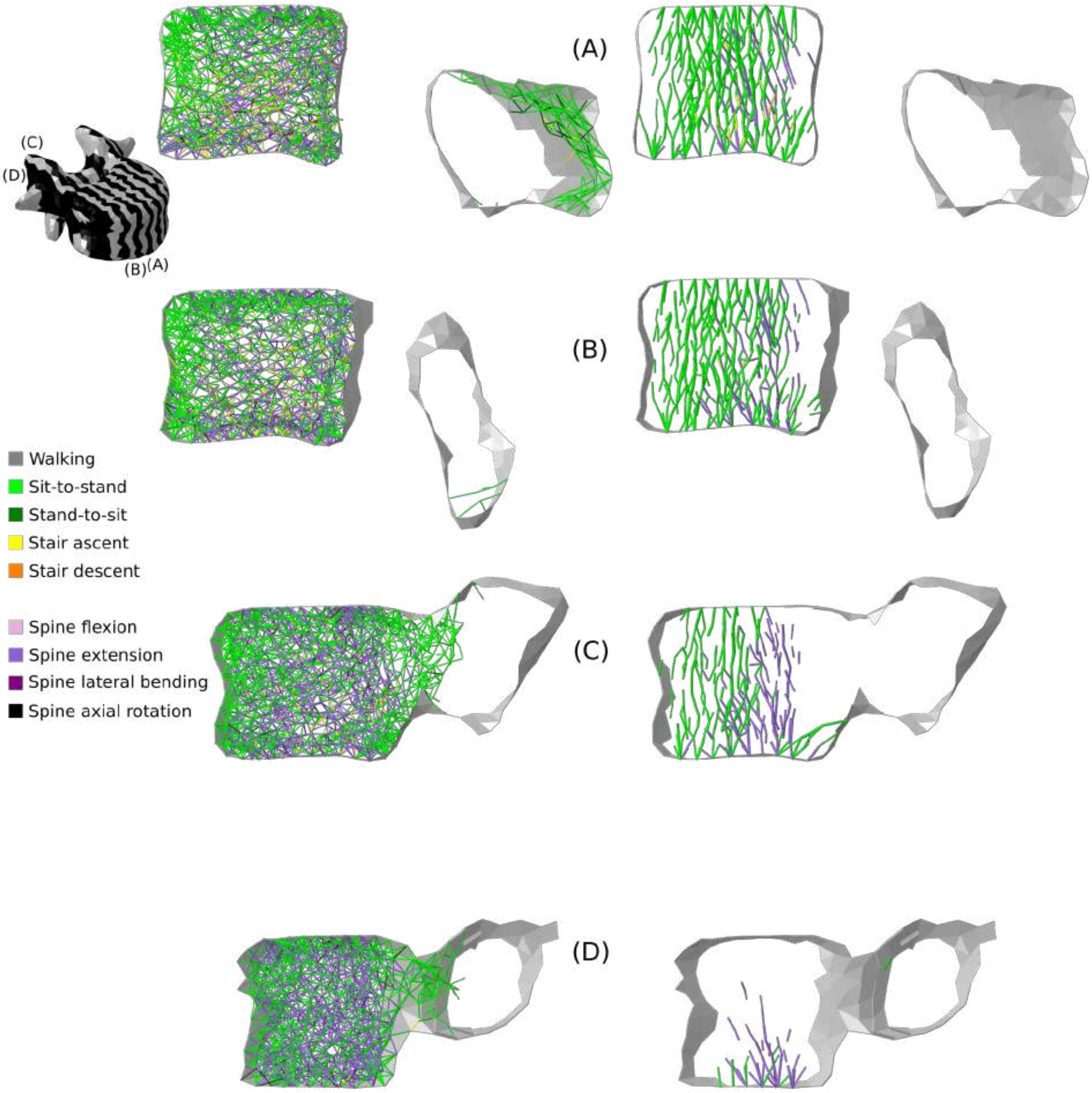

Figure S18: Selection of 3 mm sagittal slices of the right side of the converged L1 model adapted to the sedentary scenario. Trabecular truss elements are colour mapped based on the activity most influential to their final geometry. On the left, only trabecular truss elements representing the secondary structure (with a radius of 0.1 mm) are shown. On the right, only trabecular truss elements representing the primary structure (with a radius superior to 0.1 mm) are shown. Cortical shell elements are sown in light grey.

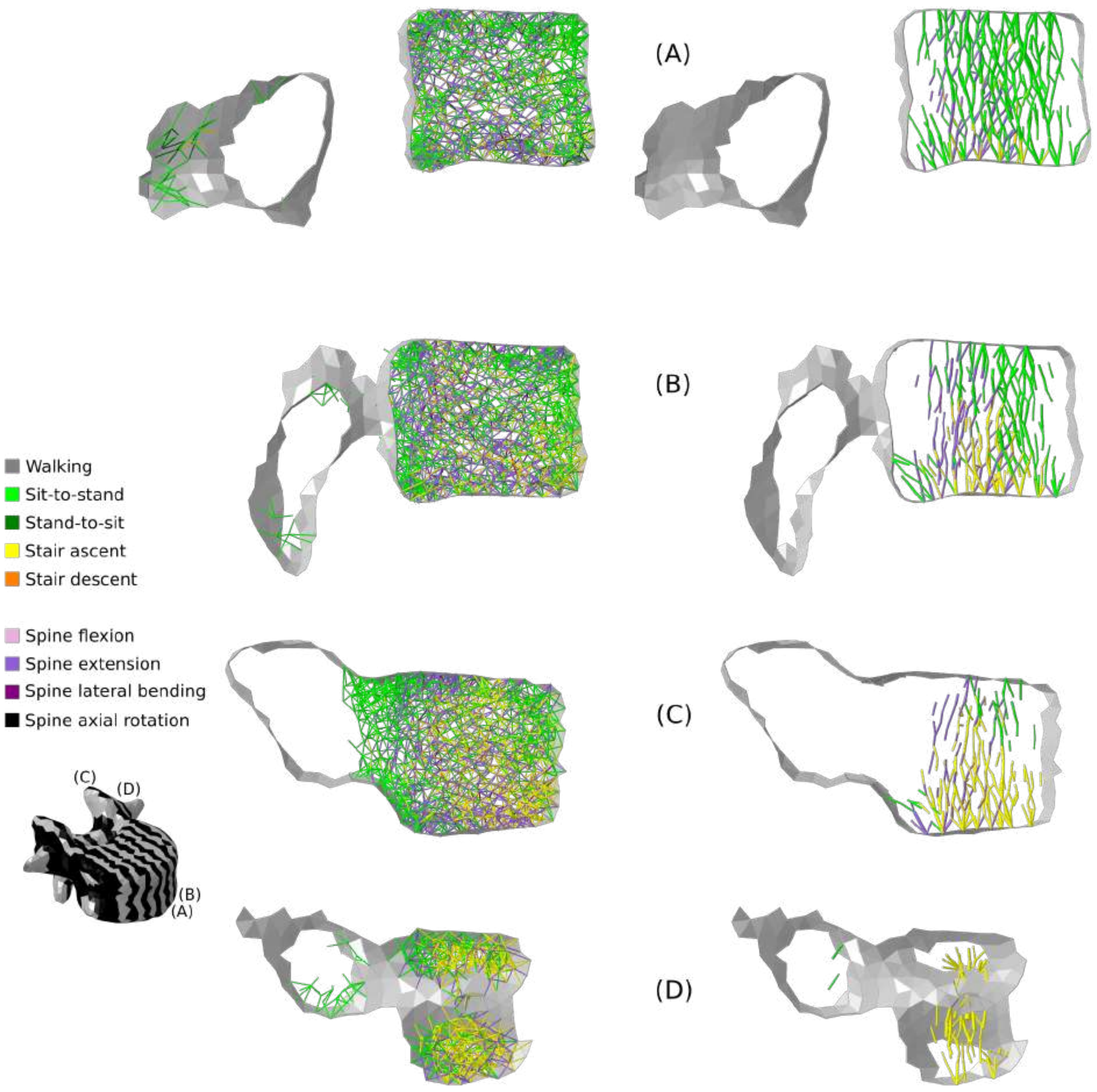

Figure S19: Selection of 3 mm sagittal slices of the left side of the converged L1 model adapted to the sedentary scenario. Trabecular truss elements are colour mapped based on the activity most influential to their final geometry. On the left, only trabecular truss elements representing the secondary structure (with a radius of 0.1 mm) are shown. On the right, only trabecular truss elements representing the primary structure (with a radius superior to 0.1 mm) are shown. Cortical shell elements are sown in light grey.

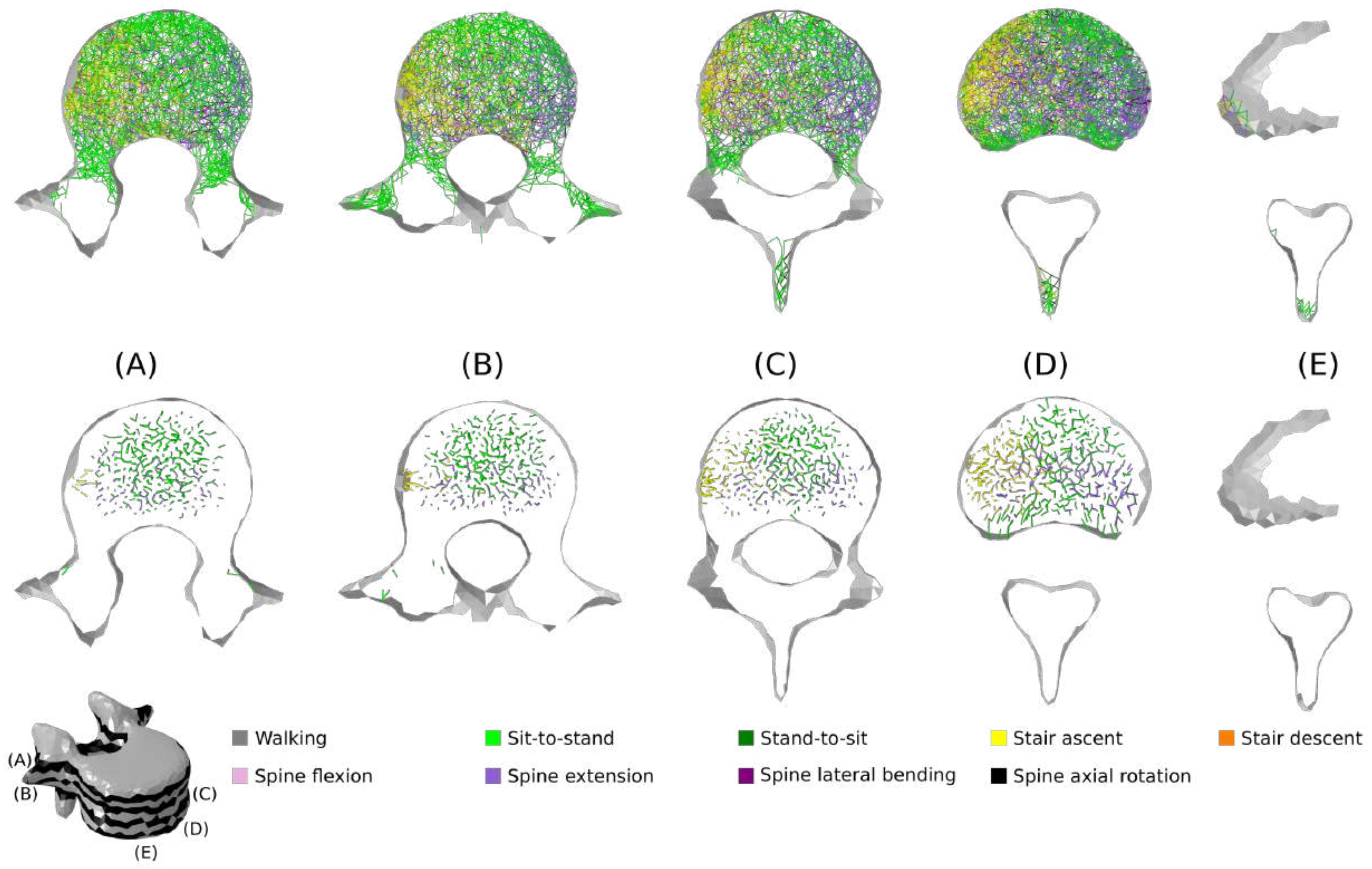

Figure S20: Selection of 3 mm transverse slices of the converged L1 model adapted to the sedentary scenario. Trabecular truss elements are colour mapped based on the activity most influential to their final geometry. On the top row, only trabecular truss elements representing the secondary structure (with a radius of 0.1 mm) are shown. On the bottom row, only trabecular truss elements with a radius superior to 0.1 mm are shown. Cortical shell elements are sown in light grey.

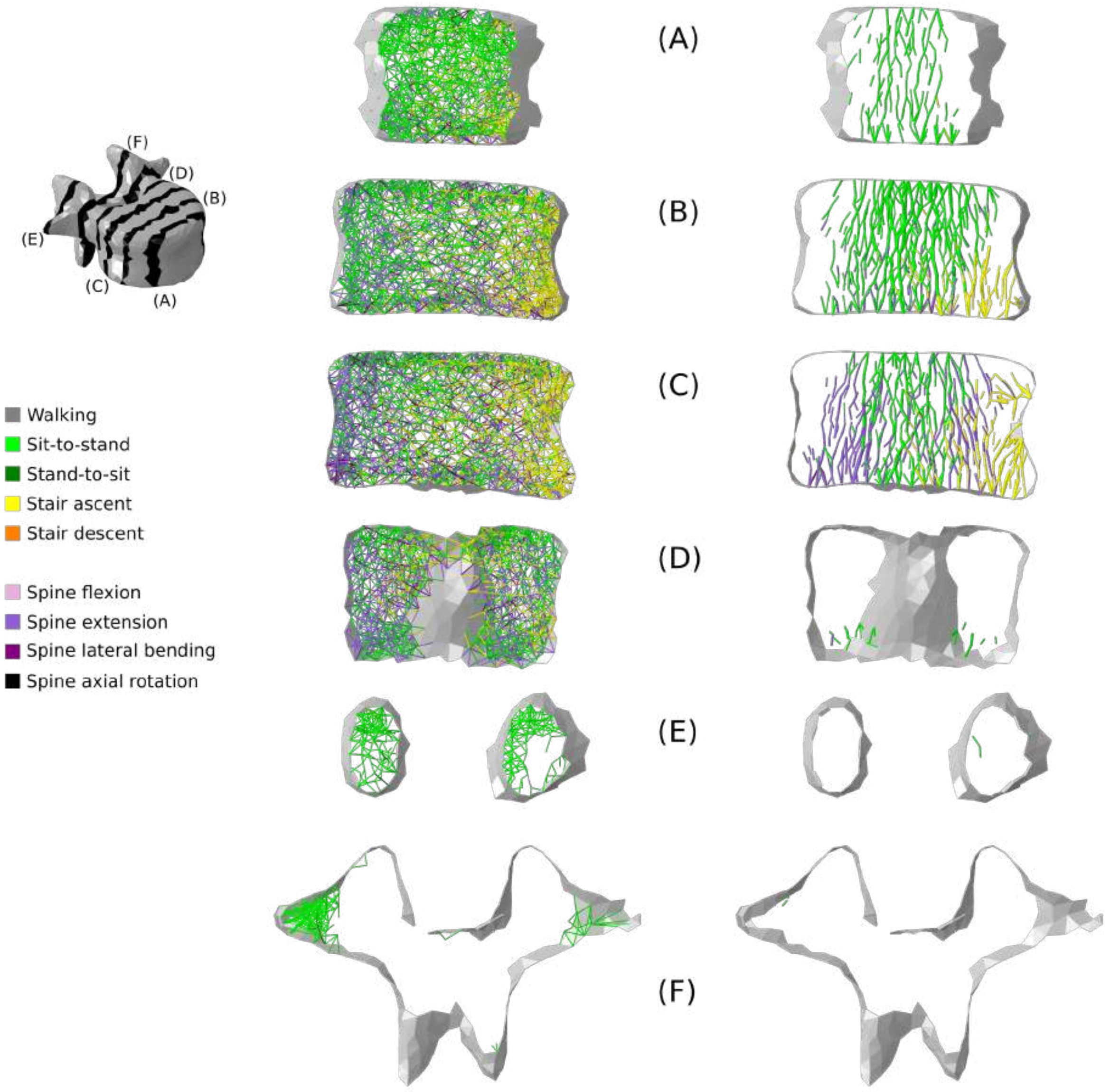

Figure S21: Selection of 3 *mm* coronal slices of the converged L1 model adapted to the sedentary scenario. Trabecular truss elements are colour mapped based on the activity most influential to their final geometry. On the left, only trabecular truss elements representing the secondary structure (with a radius of 0.1 *mm*) are shown. On the right, only trabecular truss elements representing the primary structure (with a radius superior to 0.1 *mm*) are shown. Cortical shell elements are sown in light grey.

3 SECOND LUMBAR VERTEBRA

3.1 Load cases

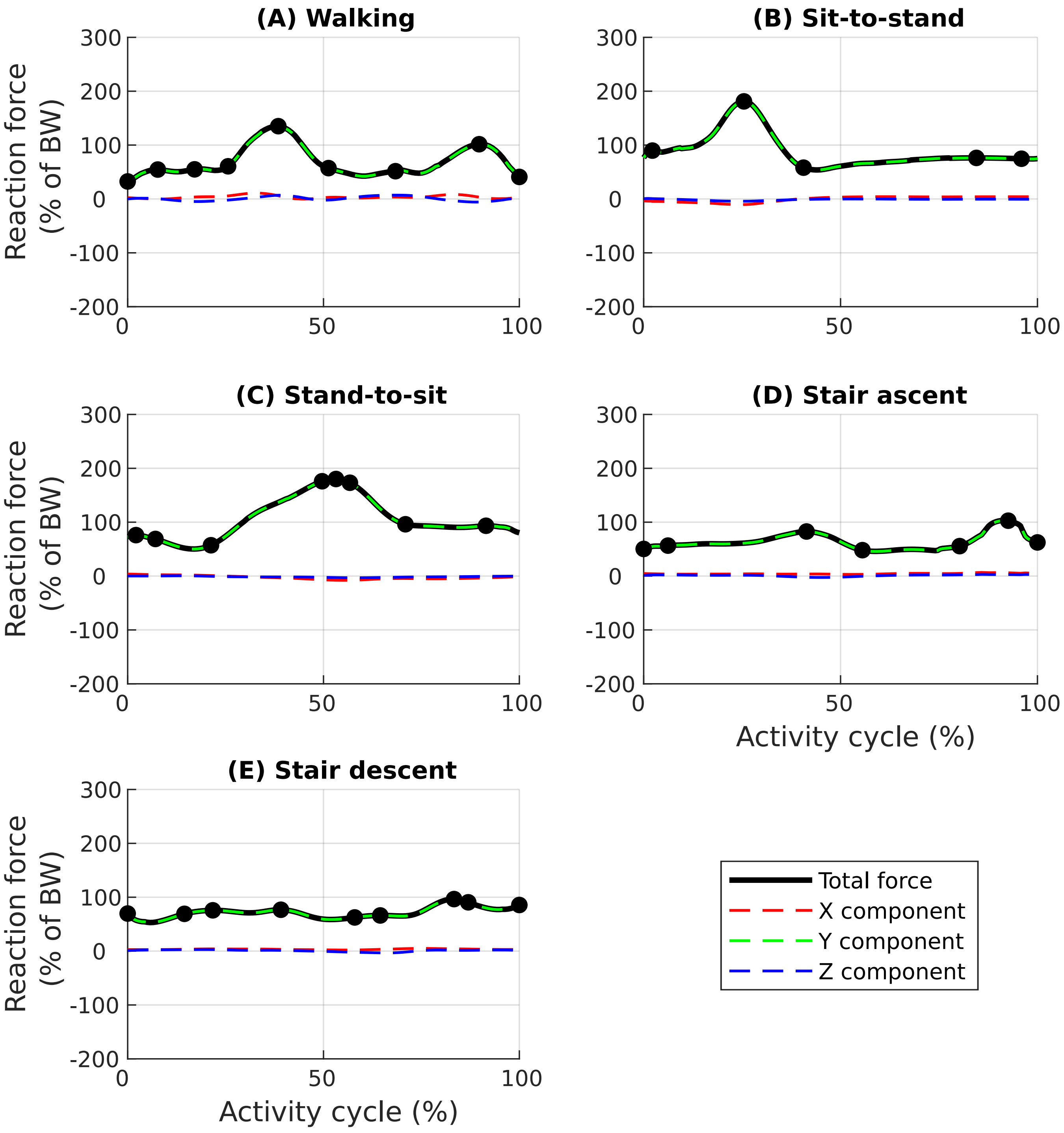

Figure S22: Selection of load cases for L2, for five activities related to locomotion ((A) level walking, (B) standing up from a chair, (C) sitting down on a chair, (D) walking up the stairs, and (E) walking down the stairs). Total reaction force at L2-L3 joint derived from the musculoskeletal model and normalised to body weight (BW) are shown as black lines. Dots indicate the frames selected for the finite element analysis. Red, green and blue dashed lines show X, Y and Z components of the reaction force expressed in the vertebra coordinate system.

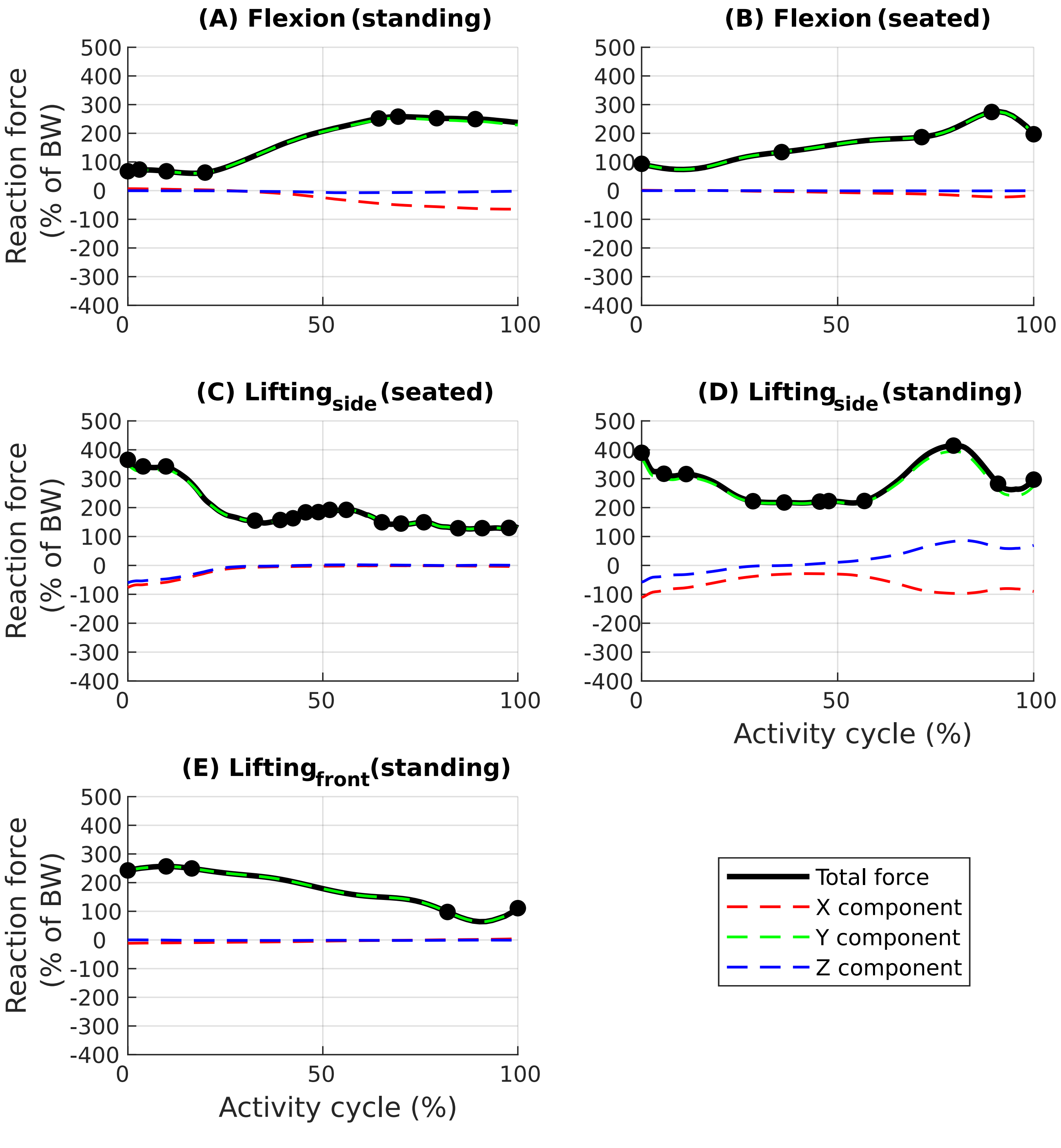

Figure S23: Selection of load cases for L2, for seven activities involving spine movements ((A) forward flexion from upright standing to maximum flexion, (B) forward flexion from upright sitting, (C) in a seated position, twisting and lifting a box from the floor, from the right side to a table in front, (D) in a standing position, twisting and lifting a box from the floor, from the left side to the right side, and (E) in a standing position, lifting a box from the floor in front to the chest). Total reaction force at L2-L3 joint derived from the musculoskeletal model and normalised to body weight (BW) are shown as black lines. Dots indicate the frames selected for the finite element analysis. Load cases obtained with activities (C) and (D) were mirrored through the sagittal plane to obtain loading on the other side. Red, green and blue dashed lines show X, Y and Z components of the reaction force expressed in the vertebra coordinate system.

3.2 Healthy scenario

3.2.1 Cortical thickness

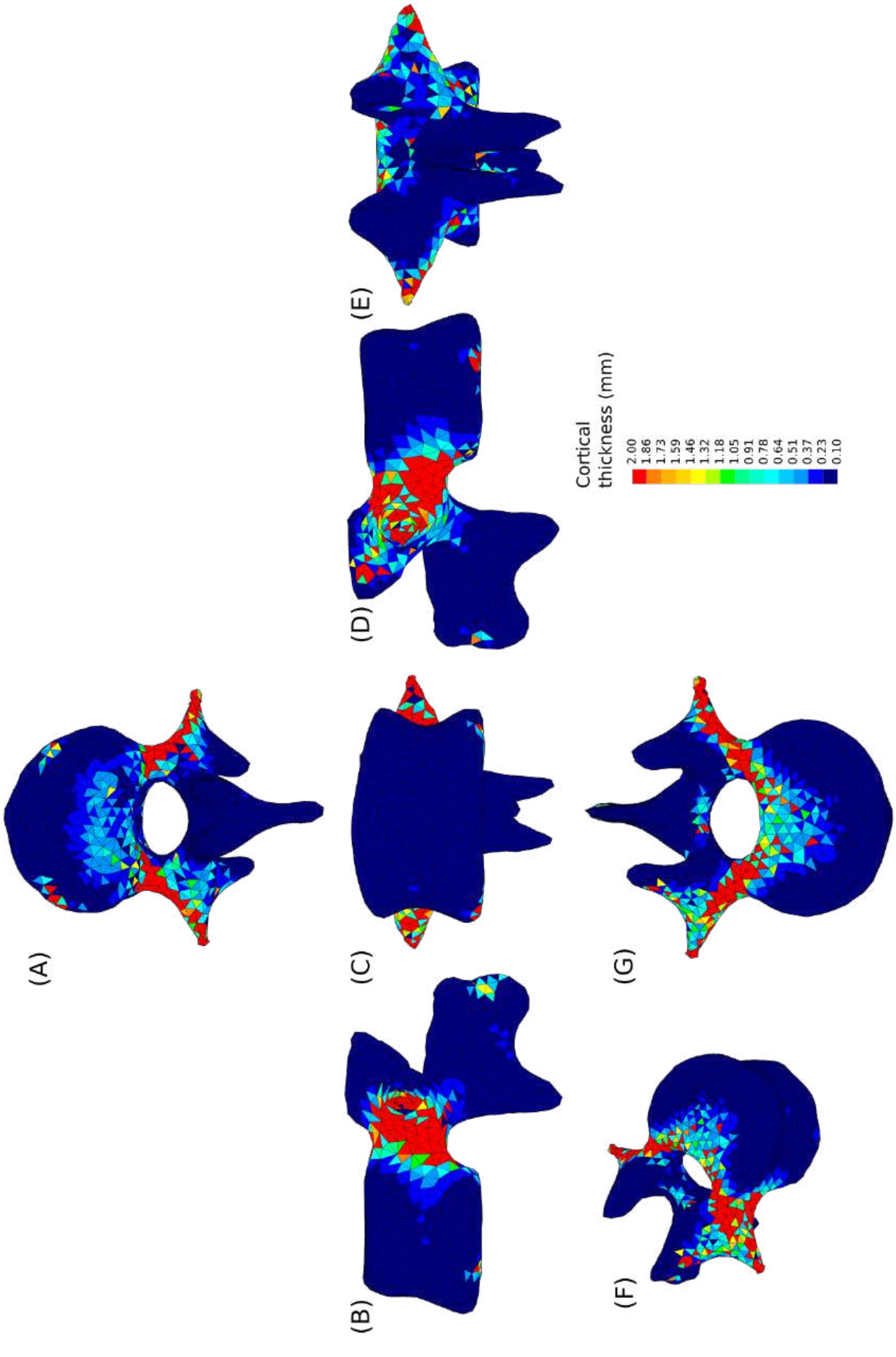

Figure S24: Cortical thickness ranging from 0.1 to 2 *mm* for the converged L2 model adapted to the healthy scenario. (A) caudal view, (B) left lateral view, (C) frontal view, (D) right lateral view, (E) dorsal view, (F) isometric view, (G) cranial view.

3.2.2 Trabecular architecture

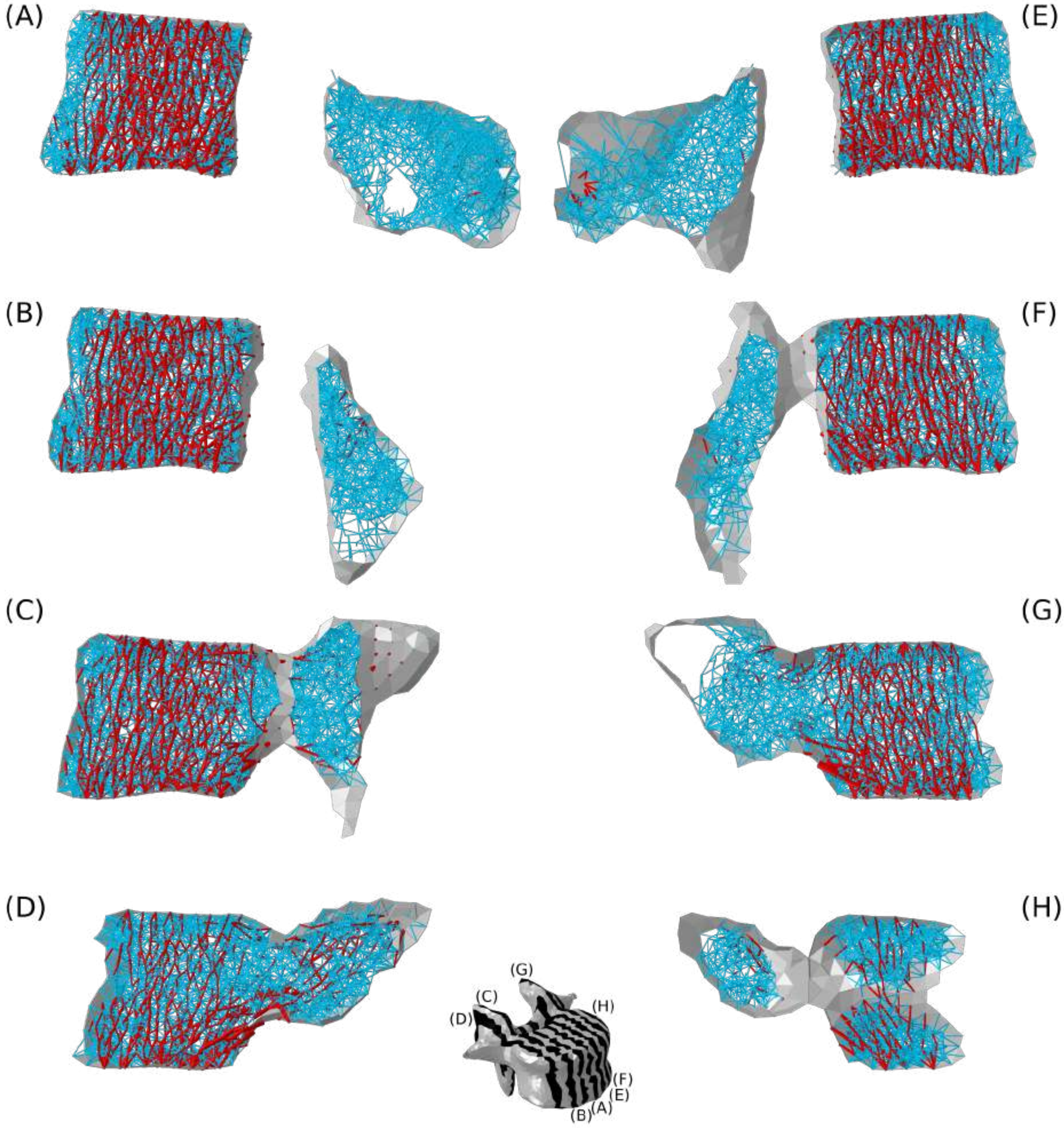

Figure S25: Selected sagittal 3 *mm* slices for the converged L2 model adapted to the healthy scenario. Cortical shell elements are shown in grey. Trabecular truss elements representing the secondary structure (with a radius of 0.1 *mm*) are shown in blue. Thicker truss elements representing the primary structure are shown in red. Truss elements in the dead zone (with a radius of 1 μm) are not shown for clarity.

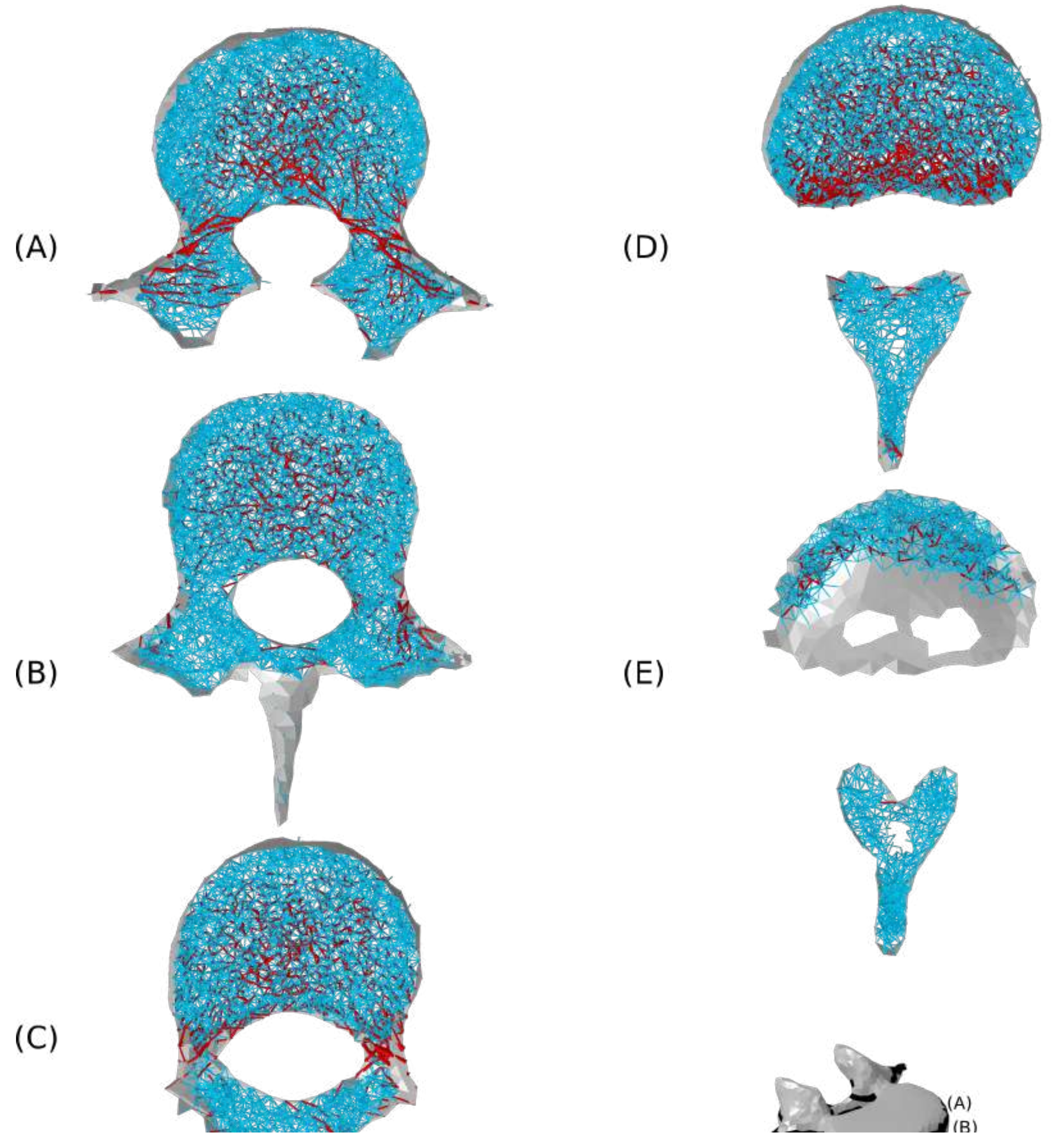

Figure S26: Selected transverse 3 mm slices for the converged L2 model adapted to the healthy scenario. Cortical shell elements are shown in grey. Trabecular truss elements representing the secondary structure (with a radius of 0.1 mm) are shown in blue. Thicker truss elements representing the primary structure are shown in red. Truss elements in the dead zone (with a radius of 1 μm) are not shown for clarity.

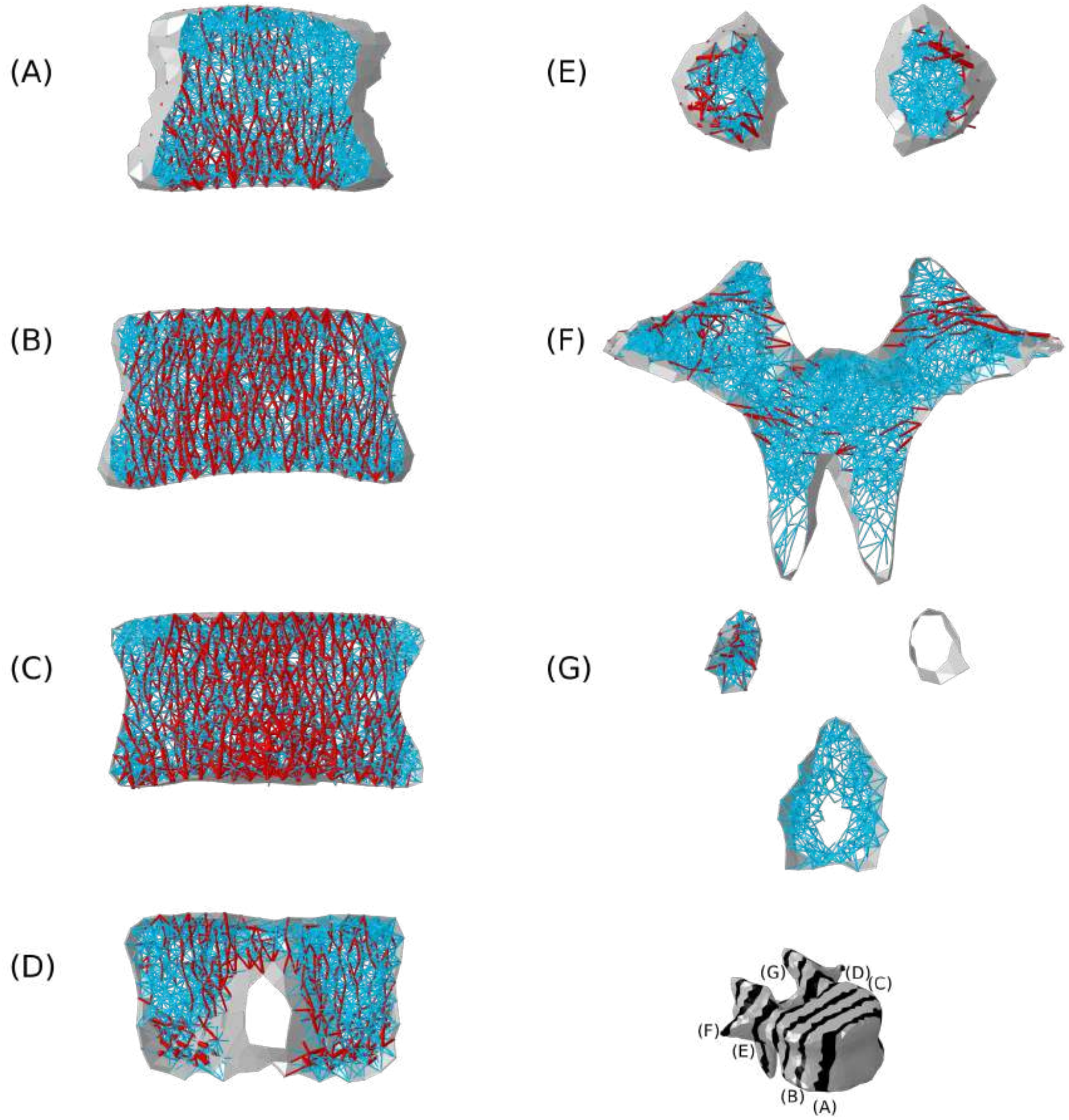

Figure S27: Selected coronal 3 mm slices for the converged L2 model adapted to the healthy scenario. Cortical shell elements are shown in grey. Trabecular truss elements representing the secondary structure (with a radius of 0.1 mm) are shown in blue. Thicker truss elements representing the primary structure are shown in red. Truss elements in the dead zone (with a radius of 1 μm) are not shown for clarity.

3.2.3 Contribution of each activities

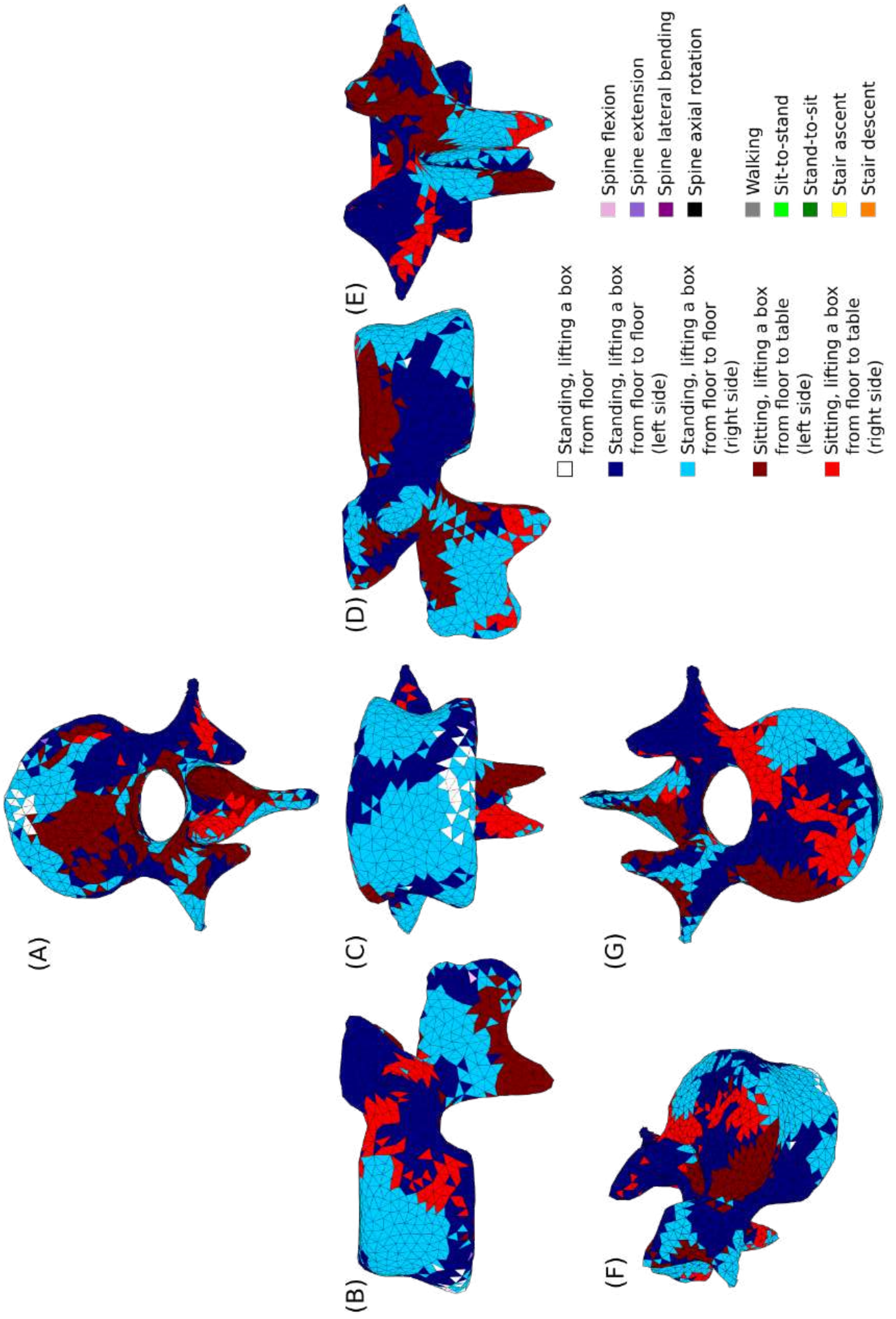

Figure S28: Contribution of each activity to the adaptation of the L2 cortical shells for the healthy scenario. (A) caudal view, (B) left lateral view, (C) frontal view, (D) right lateral view, (E) dorsal view, (F) isometric view, (G) cranial view.

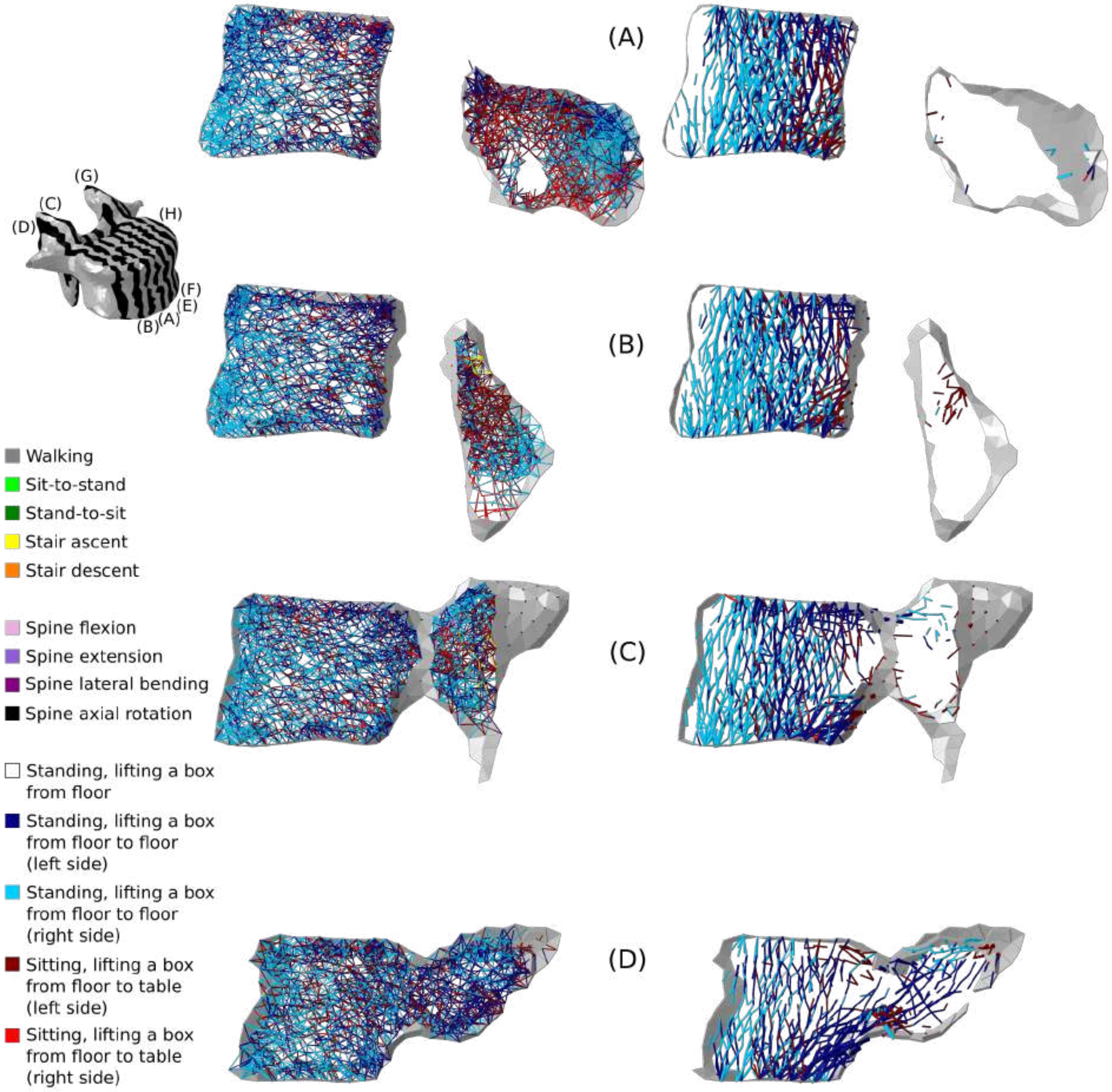

Figure S29: Selection of 3 mm sagittal slices of the right side of the converged L2 model adapted to the healthy scenario. Trabecular truss elements are colour mapped based on the activity most influential to their final geometry. On the left, only trabecular truss elements representing the secondary structure (with a radius of 0.1 mm) are shown. On the right, only trabecular truss elements representing the primary structure (with a radius superior to 0.1 mm) are shown. Cortical shell elements are sown in light grey.

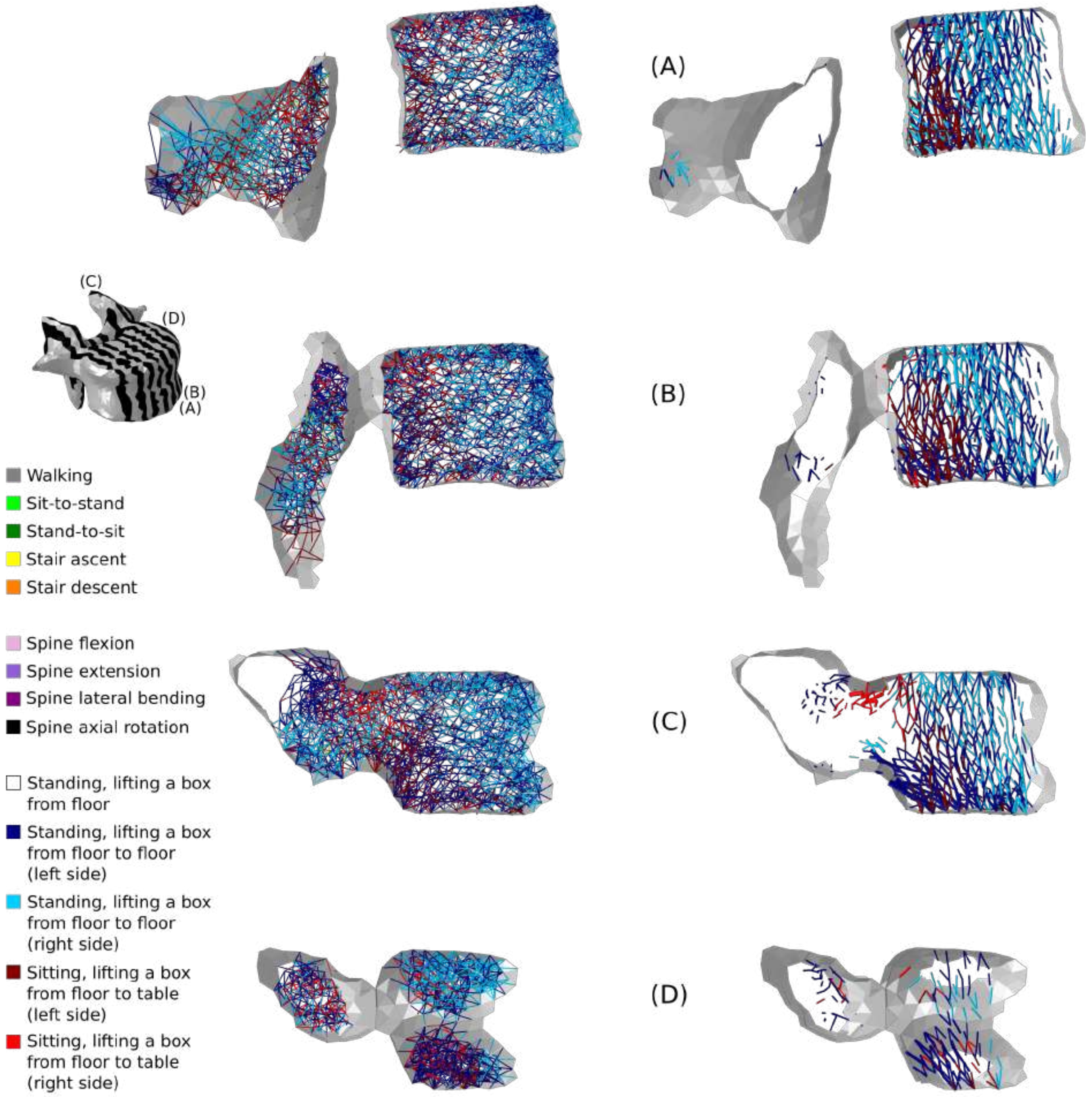

Figure S30: Selection of 3 mm sagittal slices of the left side of the converged L2 model adapted to the healthy scenario. Trabecular truss elements are colour mapped based on the activity most influential to their final geometry. On the left, only trabecular truss elements representing the secondary structure (with a radius of 0.1 mm) are shown. On the right, only trabecular truss elements representing the primary structure (with a radius superior to 0.1 mm) are shown. Cortical shell elements are sown in light grey.

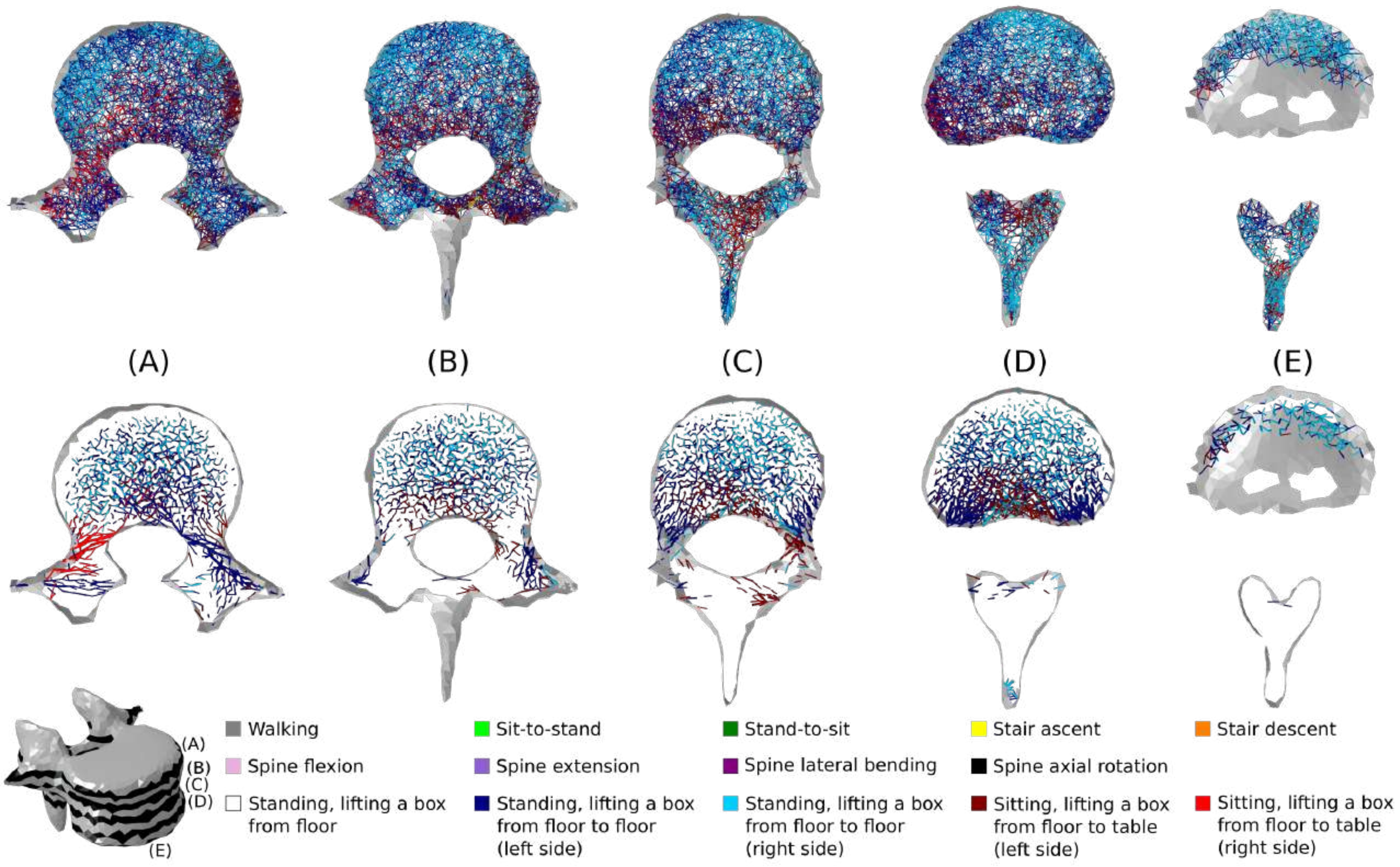

Figure S31: Selection of 3 *mm* transverse slices of the converged L2 model adapted to the healthy scenario. Trabecular truss elements are colour mapped based on the activity most influential to their final geometry. On the top row, only trabecular truss elements representing the secondary structure (with a radius of 0.1 *mm*) are shown. On the bottom row, only trabecular truss elements with a radius superior to 0.1 *mm* are shown. Cortical shell elements are sown in light grey.

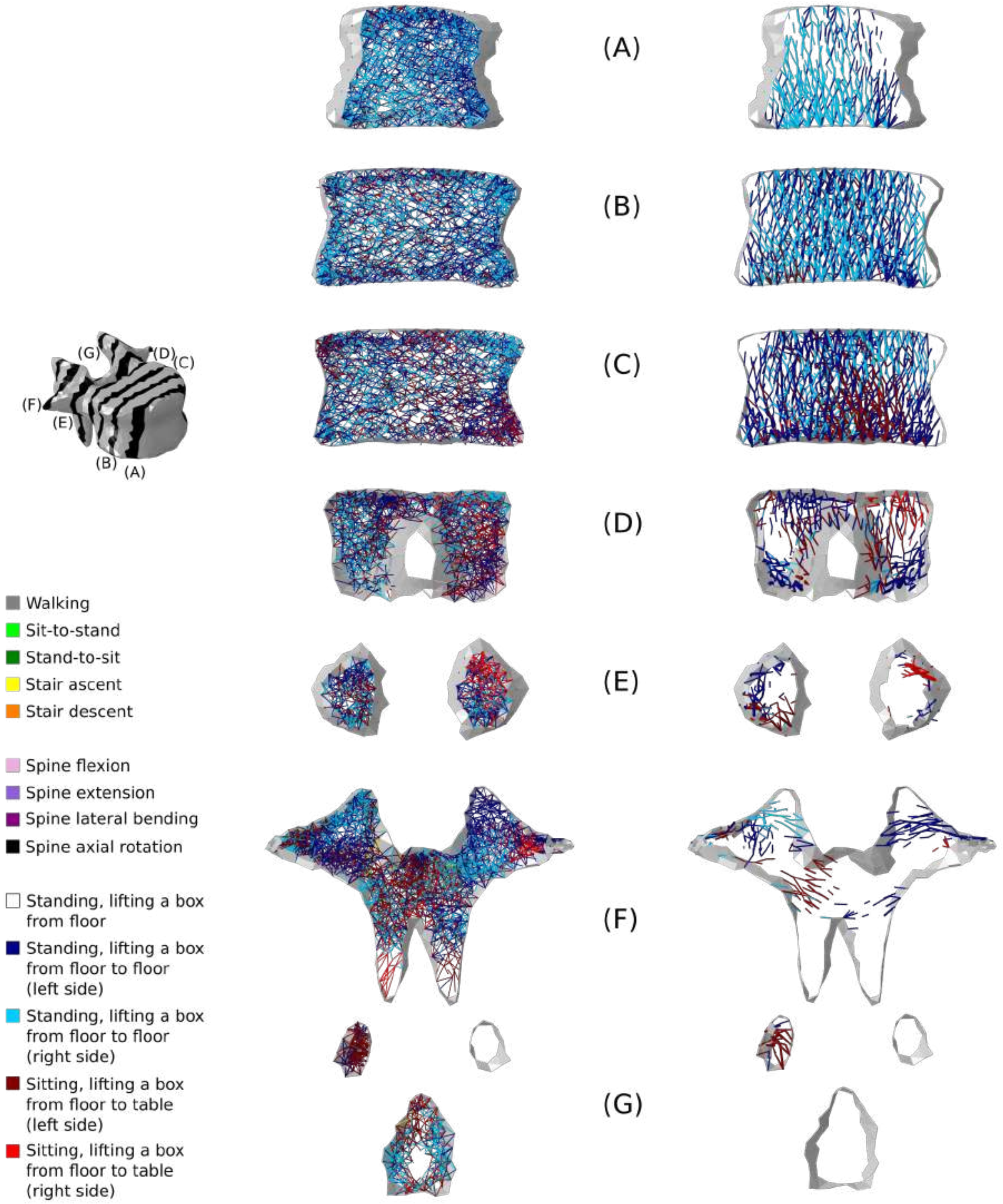

Figure S32: Selection of 3 mm coronal slices of the converged L2 model adapted to the healthy scenario. Trabecular truss elements are colour mapped based on the activity most influential to their final geometry. On the left, only trabecular truss elements representing the secondary structure (with a radius of 0.1 mm) are shown. On the right, only trabecular truss elements representing the primary structure (with a radius superior to 0.1 mm) are shown. Cortical shell elements are sown in light grey.

3.2.4 Characterisation of trabecular anisotropy

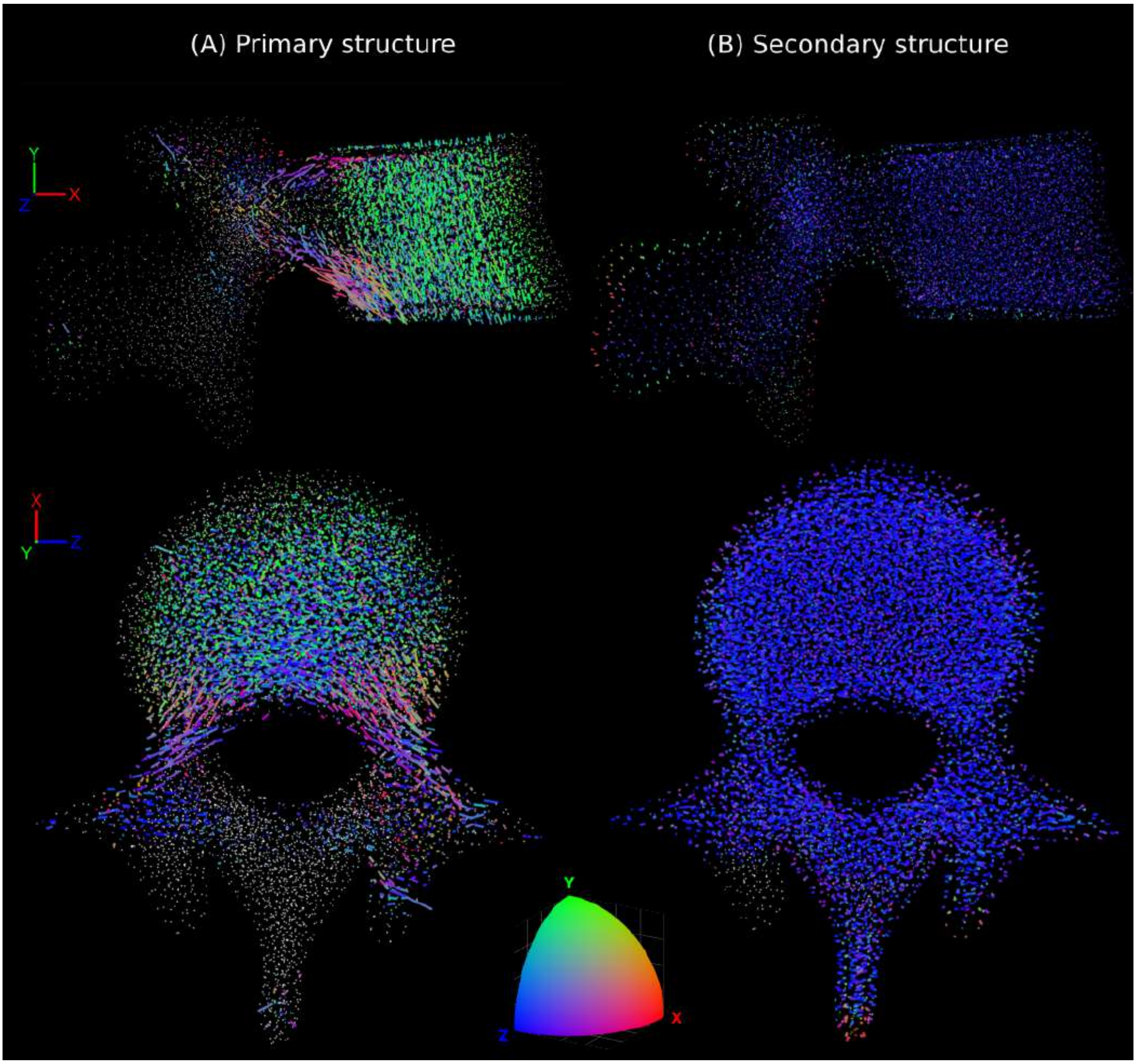

Figure S33: Characterisation of anisotropy in the trabecular bone of L2 adapted to the healthy scenario. (A) shows the orientation of the trabecular trusses of the primary structure (with a radius larger than 0.1 *mm*). (B) shows the orientation of the trabecular trusses of the secondary structure (with a radius of 0.1 *mm*). For (A) and (B), side view (top row) and top view (bottom row) are shown. Lines are attached to each trabecular node, with colour and length varying respectively with the orientations and radii of the truss elements connected to that particular node. The colour scale at the bottom shows how the colour of the lines should be interpreted. Orientation along the X, Y or Z axes are in red, green or blue respectively. Any orientation that is not colinear with these axes shows as a combination of red, green and blue. A white dot indicates a node without elements in the size range being looked at connected to it.

3.3 Sedentary scenario

3.3.1 Cortical thickness

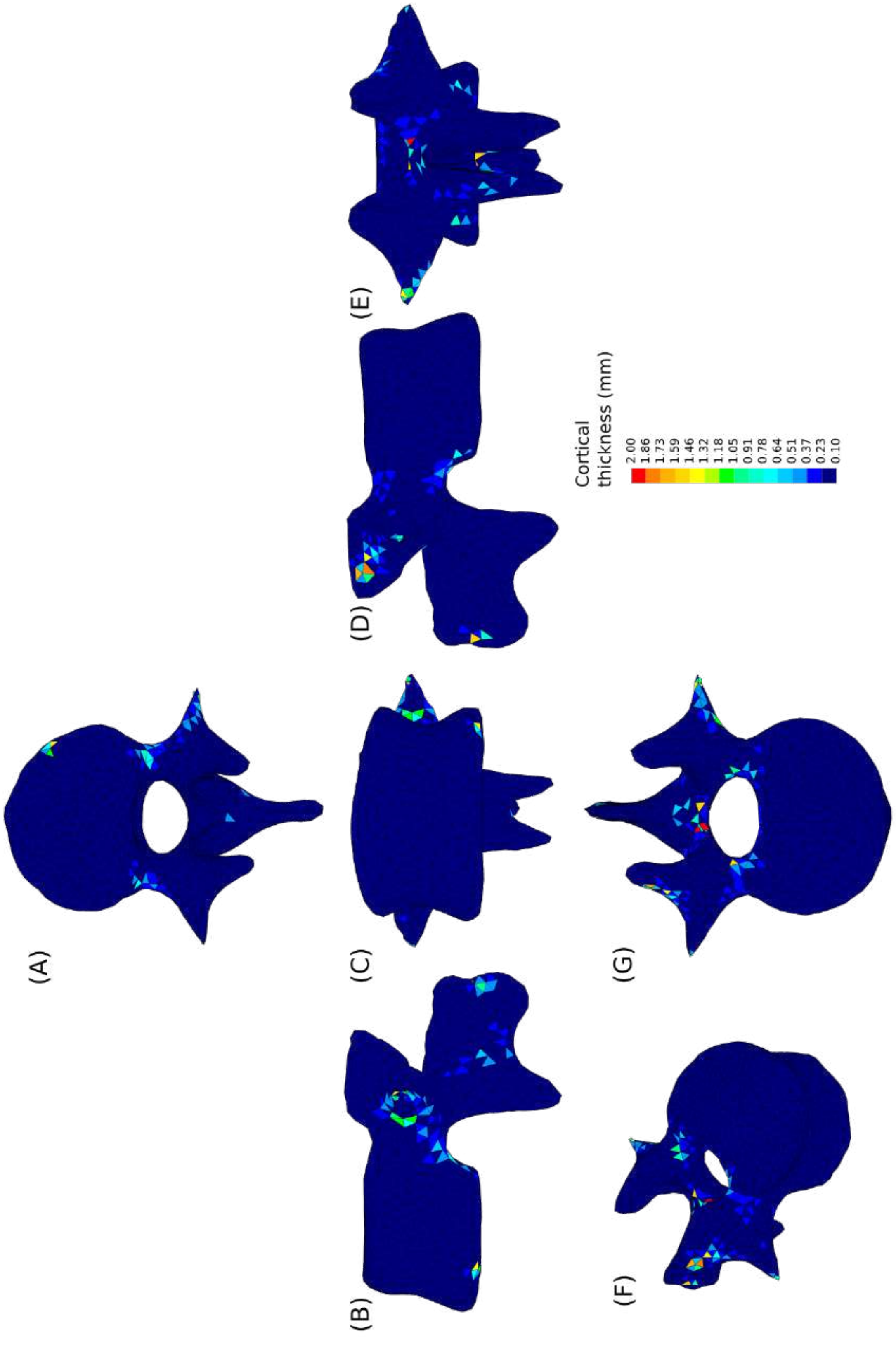

Figure S34: Cortical thickness ranging from 0.1 to 2 *mm* for the converged L2 model adapted to the sedentary scenario. (A) caudal view, (B) left lateral view, (C) frontal view, (D) right lateral view, (E) dorsal view, (F) isometric view, (G) cranial view.

3.3.2 Trabecular architecture

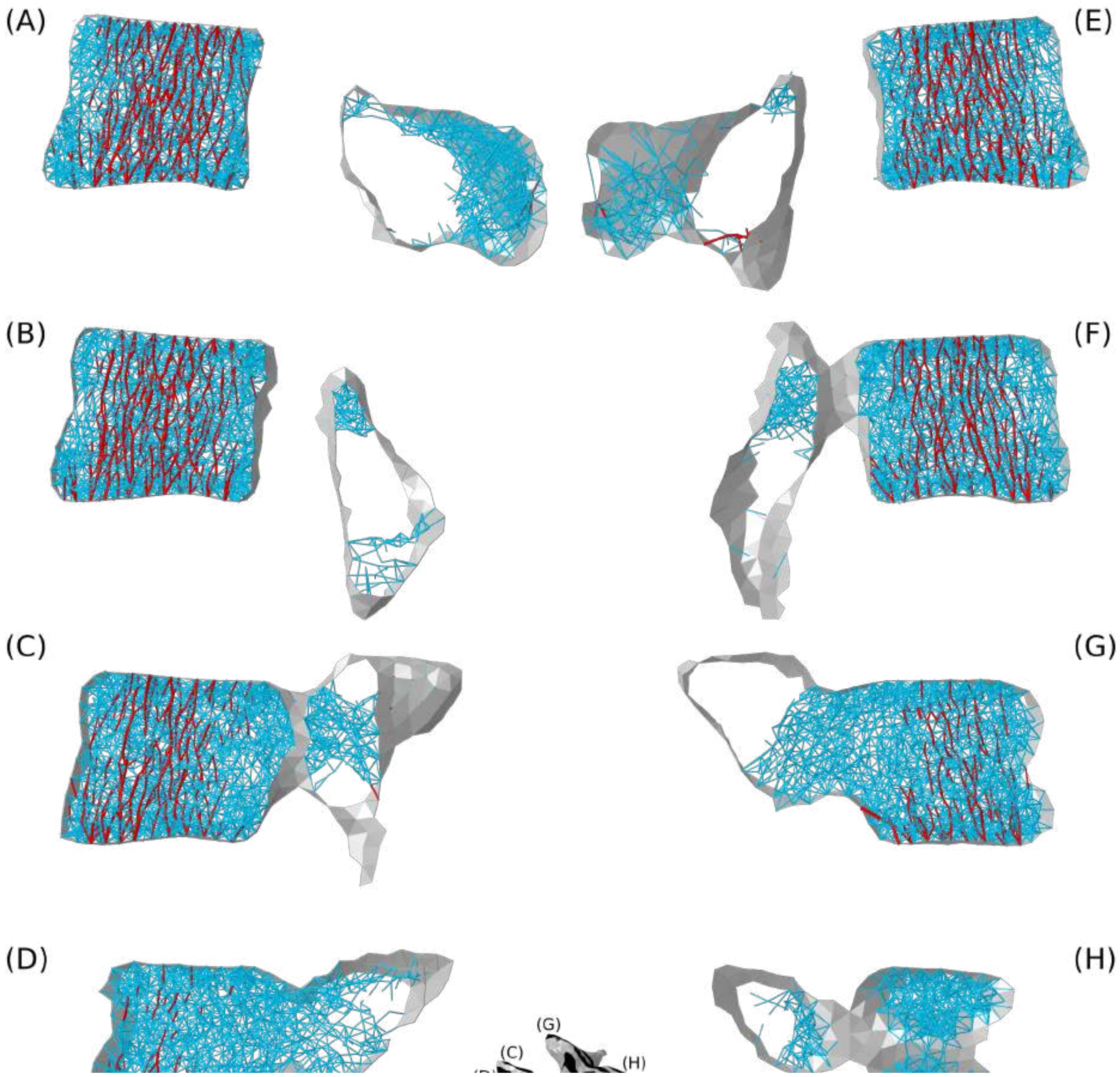

Figure S35: Selected sagittal 3 mm slices for the converged L2 model adapted to the sedentary scenario. Cortical shell elements are shown in grey. Trabecular truss elements representing the secondary structure (with a radius of 0.1 mm) are shown in blue. Thicker truss elements representing the primary structure are shown in red. Truss elements in the dead zone (with a radius of 1 μm) are not shown for clarity.

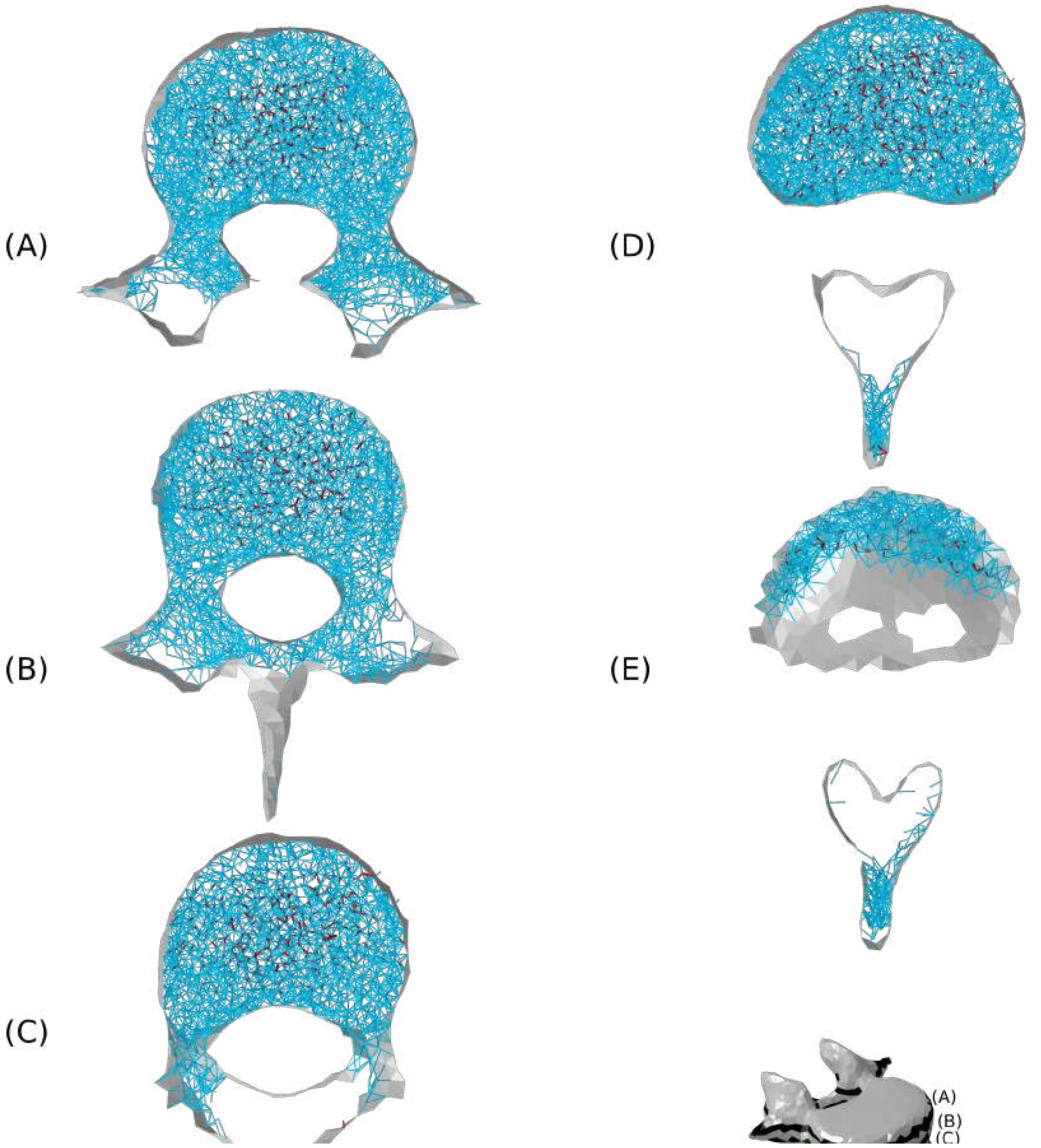

Figure S36: Selected transverse 3 mm slices for the converged L2 model adapted to the sedentary scenario. Cortical shell elements are shown in grey. Trabecular truss elements representing the secondary structure (with a radius of 0.1 mm) are shown in blue. Thicker truss elements representing the primary structure are shown in red. Truss elements in the dead zone (with a radius of 1 μm) are not shown for clarity.

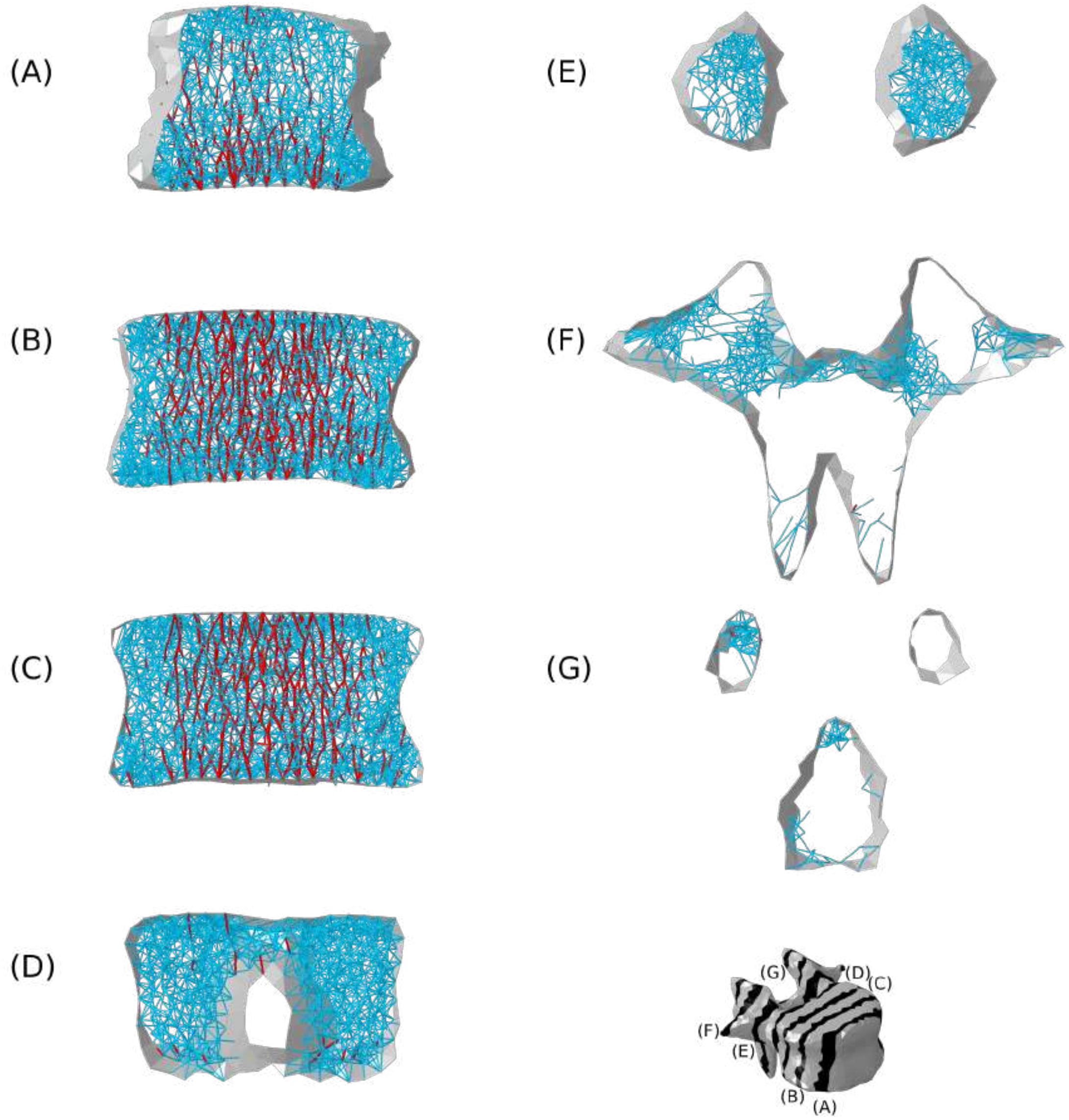

Figure S37: Selected coronal 3 mm slices for the converged L2 model adapted to the sedentary scenario. Cortical shell elements are shown in grey. Trabecular truss elements representing the secondary structure (with a radius of 0.1 mm) are shown in blue. Thicker truss elements representing the primary structure are shown in red. Truss elements in the dead zone (with a radius of 1 μm) are not shown for clarity.

3.3.3 Contribution of each activities

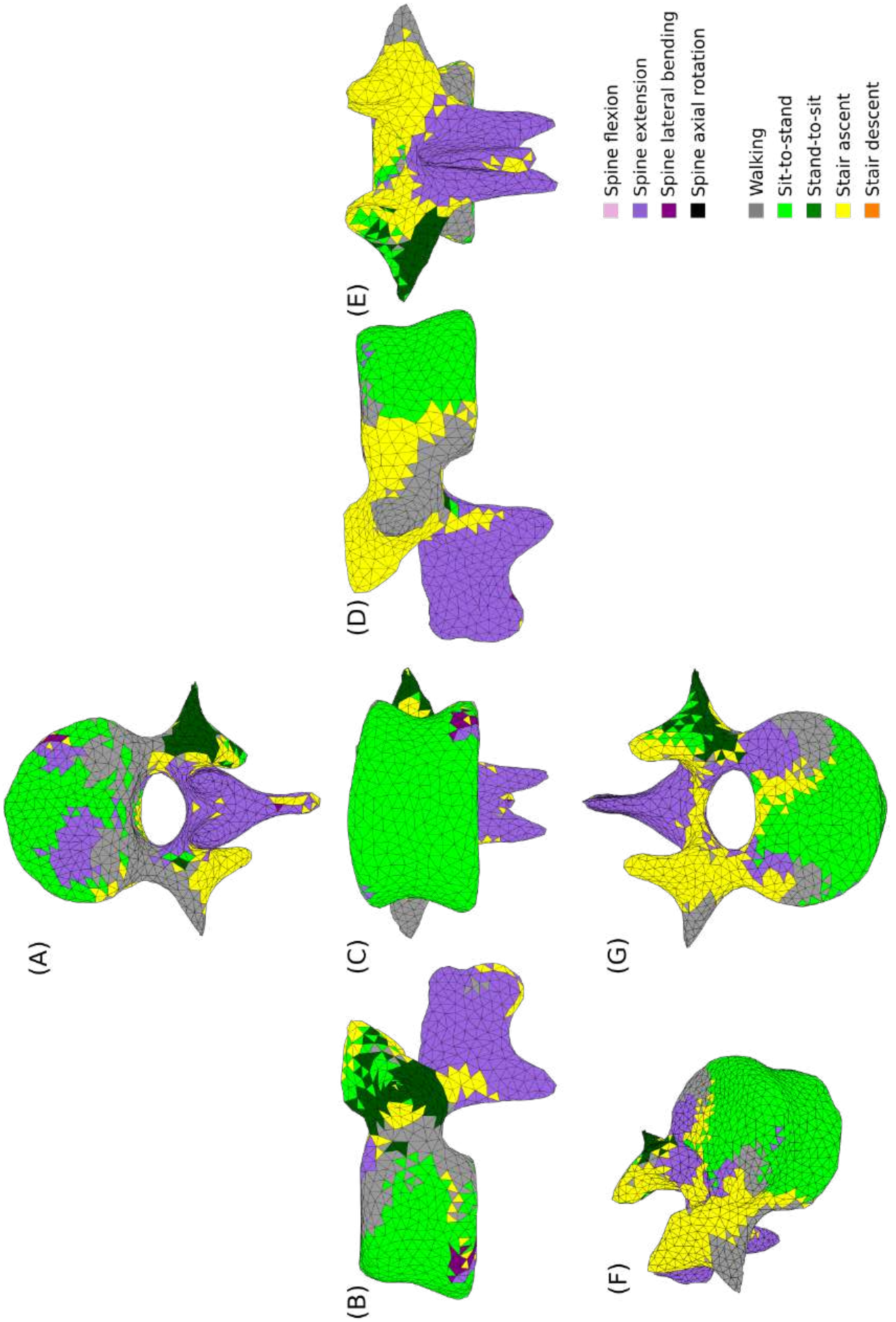

Figure S38: Contribution of each activity to the adaptation of the L2 cortical shells for the sedentary scenario. (A) caudal view, (B) left lateral view, (C) frontal view, (D) right lateral view, (E) dorsal view, (F) isometric view, (G) cranial view.

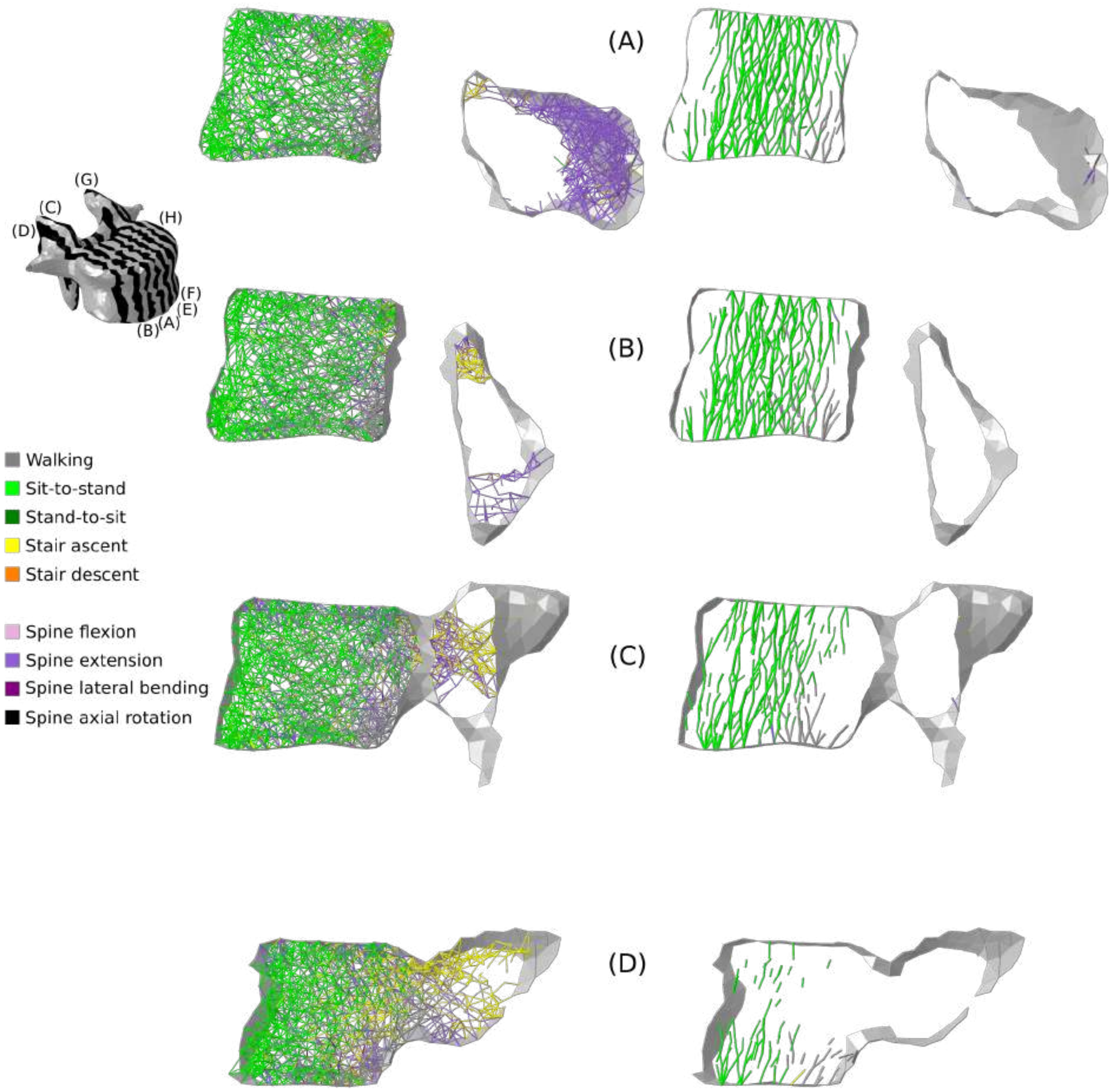

Figure S39: Selection of 3 *mm* sagittal slices of the right side of the converged L2 model adapted to the sedentary scenario. Trabecular truss elements are colour mapped based on the activity most influential to their final geometry. On the left, only trabecular truss elements representing the secondary structure (with a radius of 0.1 *mm*) are shown. On the right, only trabecular truss elements representing the primary structure (with a radius superior to 0.1 *mm*) are shown. Cortical shell elements are sown in light grey.

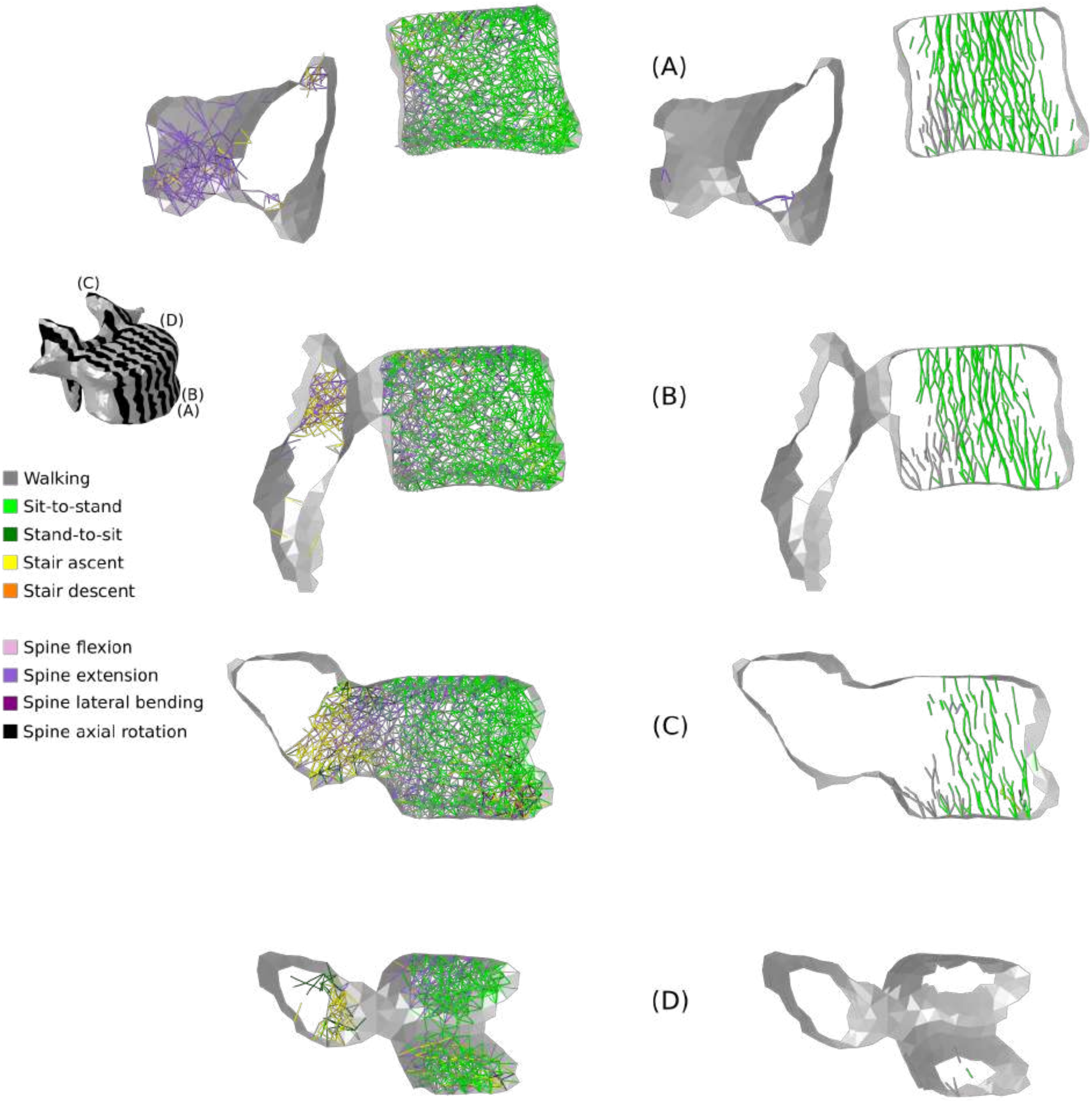

Figure S40: Selection of 3 *mm* sagittal slices of the left side of the converged L2 model adapted to the sedentary scenario. Trabecular truss elements are colour mapped based on the activity most influential to their final geometry. On the left, only trabecular truss elements representing the secondary structure (with a radius of 0.1 *mm*) are shown. On the right, only trabecular truss elements representing the primary structure (with a radius superior to 0.1 *mm*) are shown. Cortical shell elements are sown in light grey.

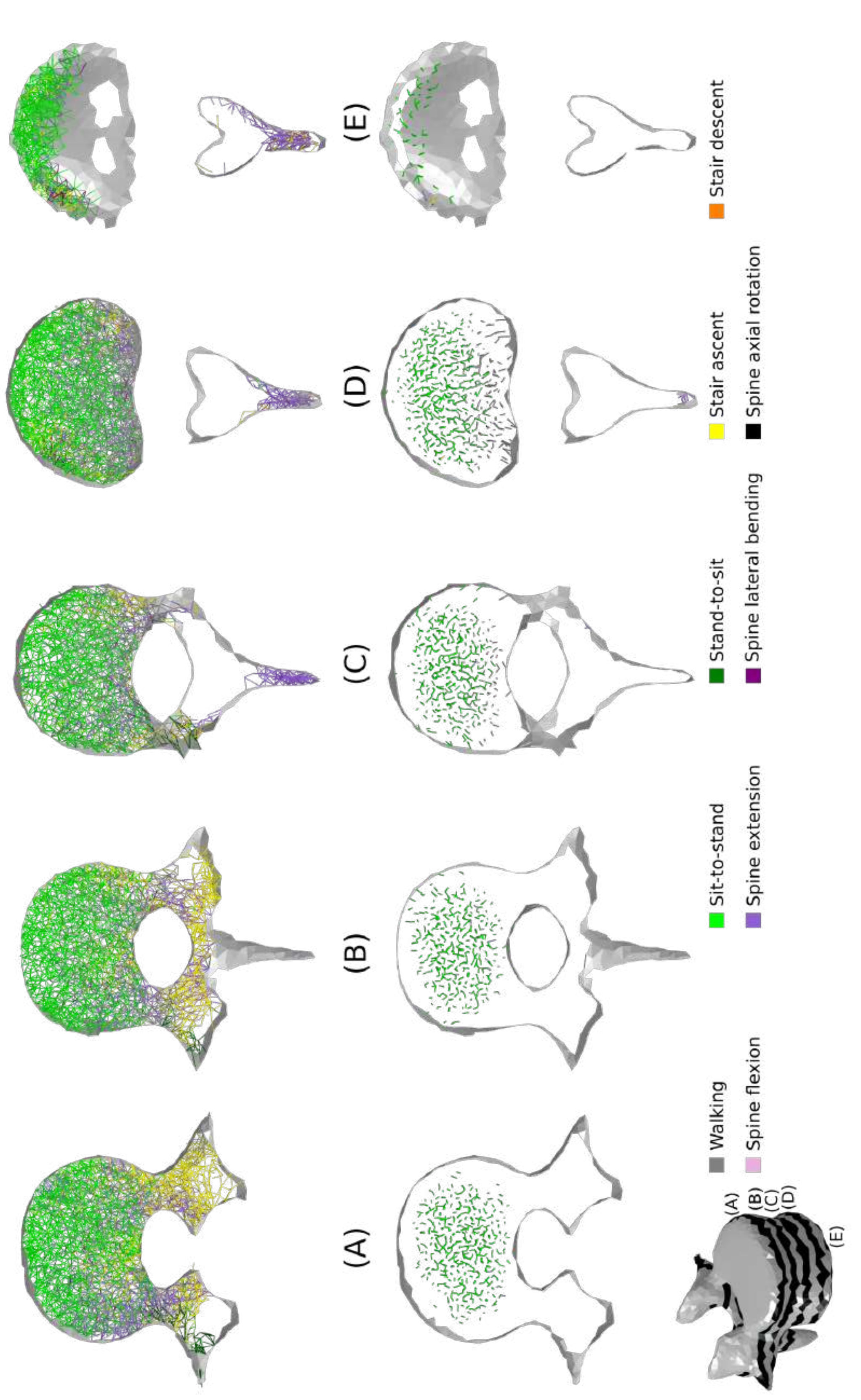

Figure S41: Selection of 3 *mm* transverse slices of the converged L2 model adapted to the sedentary scenario. Trabecular truss elements are colour mapped based on the activity most influential to their final geometry. On the top row, only trabecular truss elements representing the secondary structure (with a radius of 0.1 *mm*) are shown. On the bottom row, only trabecular truss elements with a radius superior to 0.1 *mm* are shown. Cortical shell elements are sown in light grey.

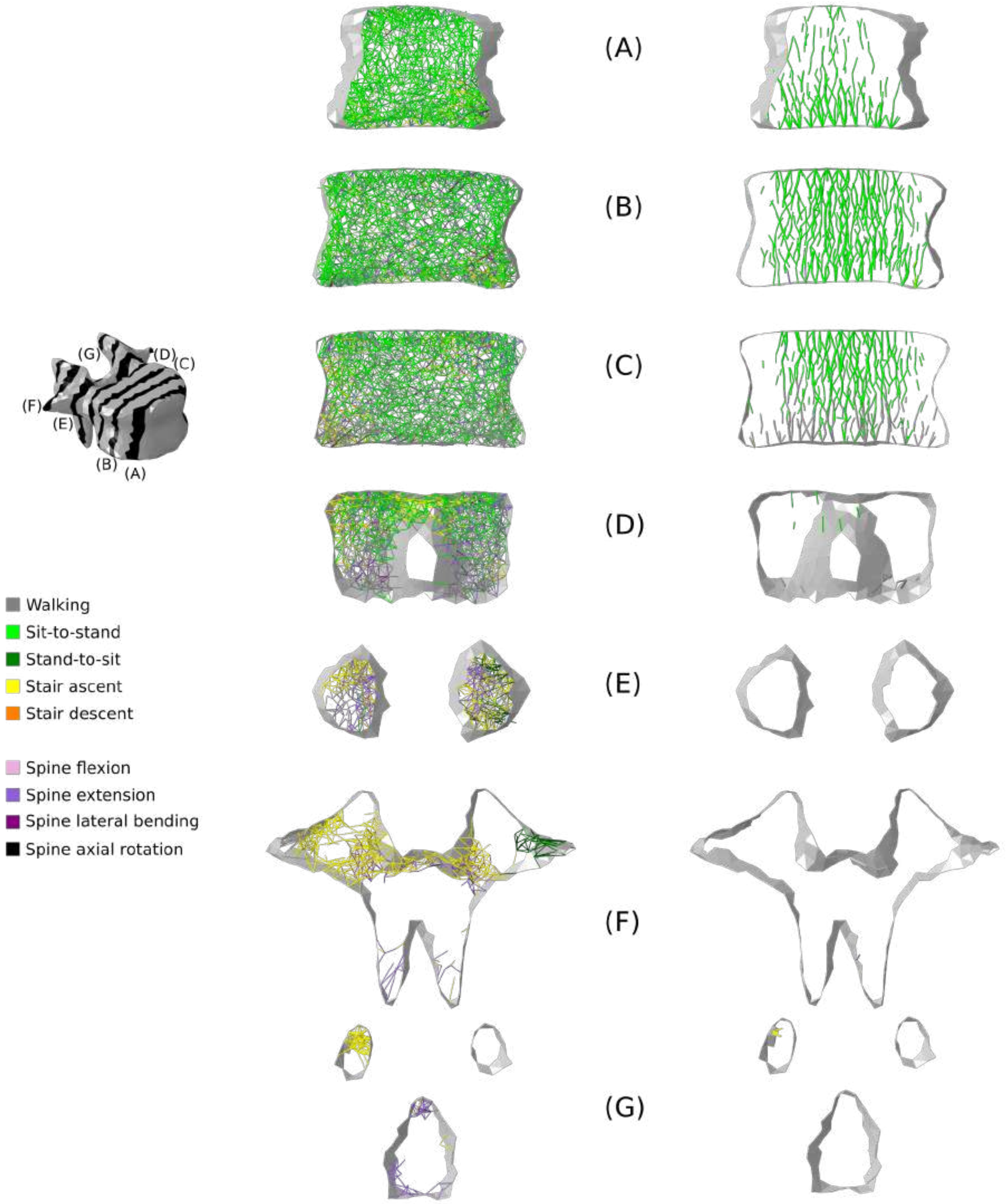

Figure S42: Selection of 3 mm coronal slices of the converged L2 model adapted to the sedentary scenario. Trabecular truss elements are colour mapped based on the activity most influential to their final geometry. On the left, only trabecular truss elements representing the secondary structure (with a radius of 0.1 mm) are shown. On the right, only trabecular truss elements representing the primary structure (with a radius superior to 0.1 mm) are shown. Cortical shell elements are sown in light grey.

4 THIRD LUMBAR VERTEBRA

4.1 Load cases

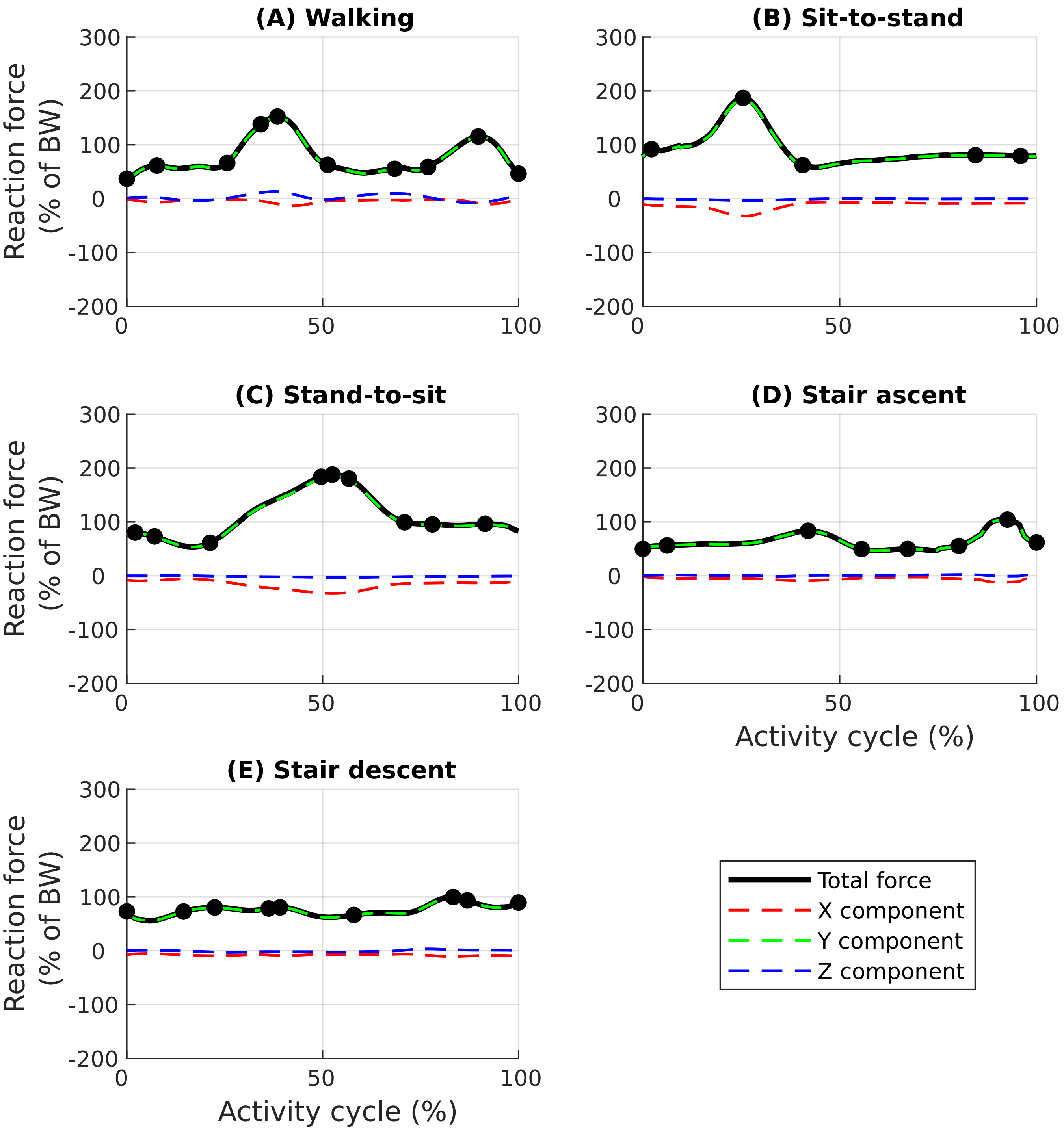

Figure S43: Selection of load cases for L3, for five activities related to locomotion ((A) level walking, (B) standing up from a chair, (C) sitting down on a chair, (D) walking up the stairs, and (E) walking down the stairs). Total reaction force at L3-L4 joint derived from the musculoskeletal model and normalised to body weight (BW) are shown as black lines. Dots indicate the frames selected for the finite element analysis. Red, green and blue dashed lines show X, Y and Z components of the reaction force expressed in the vertebra coordinate system.

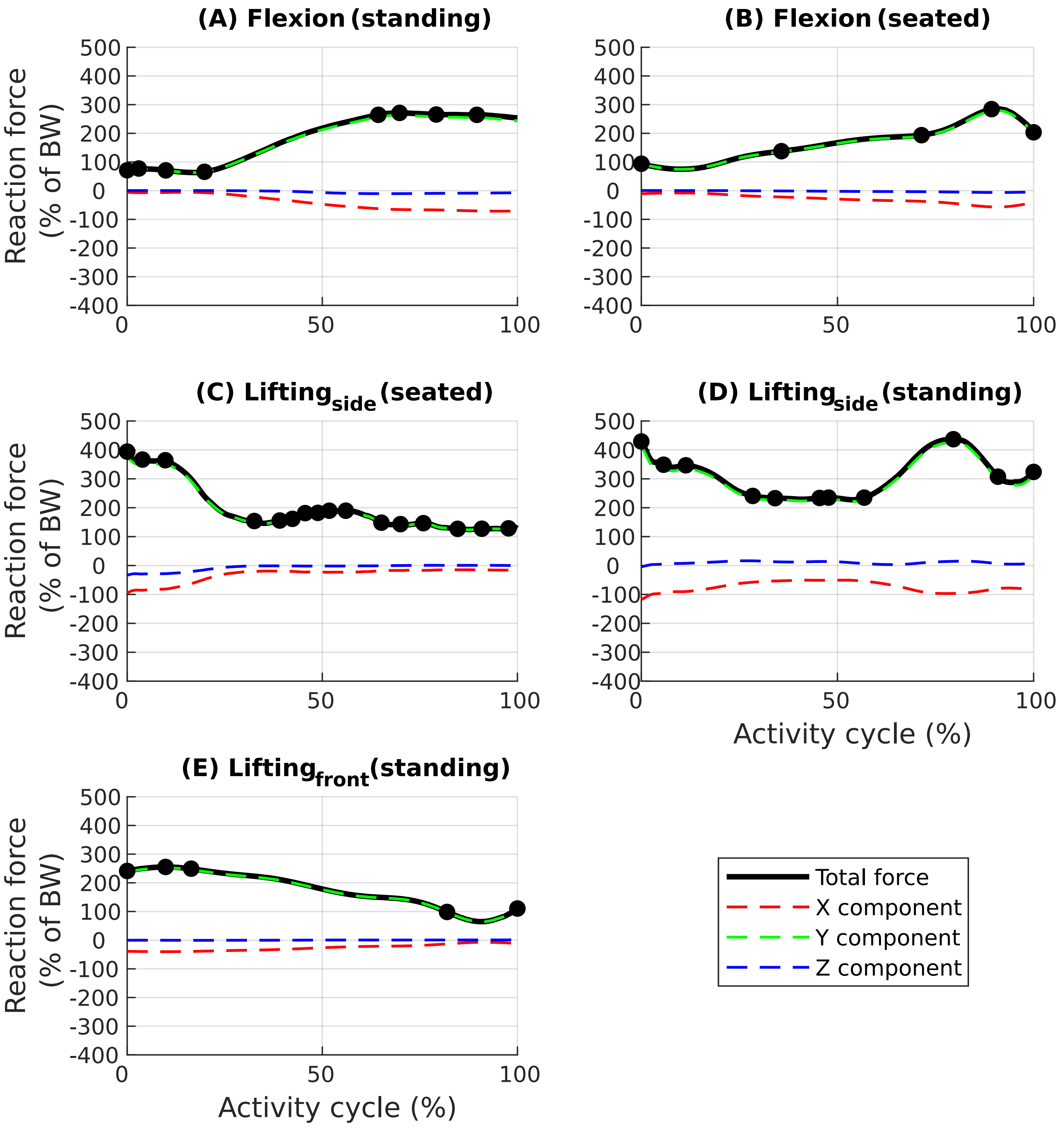

Figure S44: Selection of load cases for L3, for seven activities involving spine movements ((A) forward flexion from upright standing to maximum flexion, (B) forward flexion from upright sitting, (C) in a seated position, twisting and lifting a box from the floor, from the right side to a table in front, (D) in a standing position, twisting and lifting a box from the floor, from the left side to the right side, and (E) in a standing position, lifting a box from the floor in front to the chest). Total reaction force at L3-L4 joint derived from the musculoskeletal model and normalised to body weight (BW) are shown as black lines. Dots indicate the frames selected for the finite element analysis. Load cases obtained with activities (C) and (D) were mirrored through the sagittal plane to obtain loading on the other side. Red, green and blue dashed lines show X, Y and Z components of the reaction force expressed in the vertebra coordinate system.

4.2 Healthy scenario

4.2.1 Cortical thickness

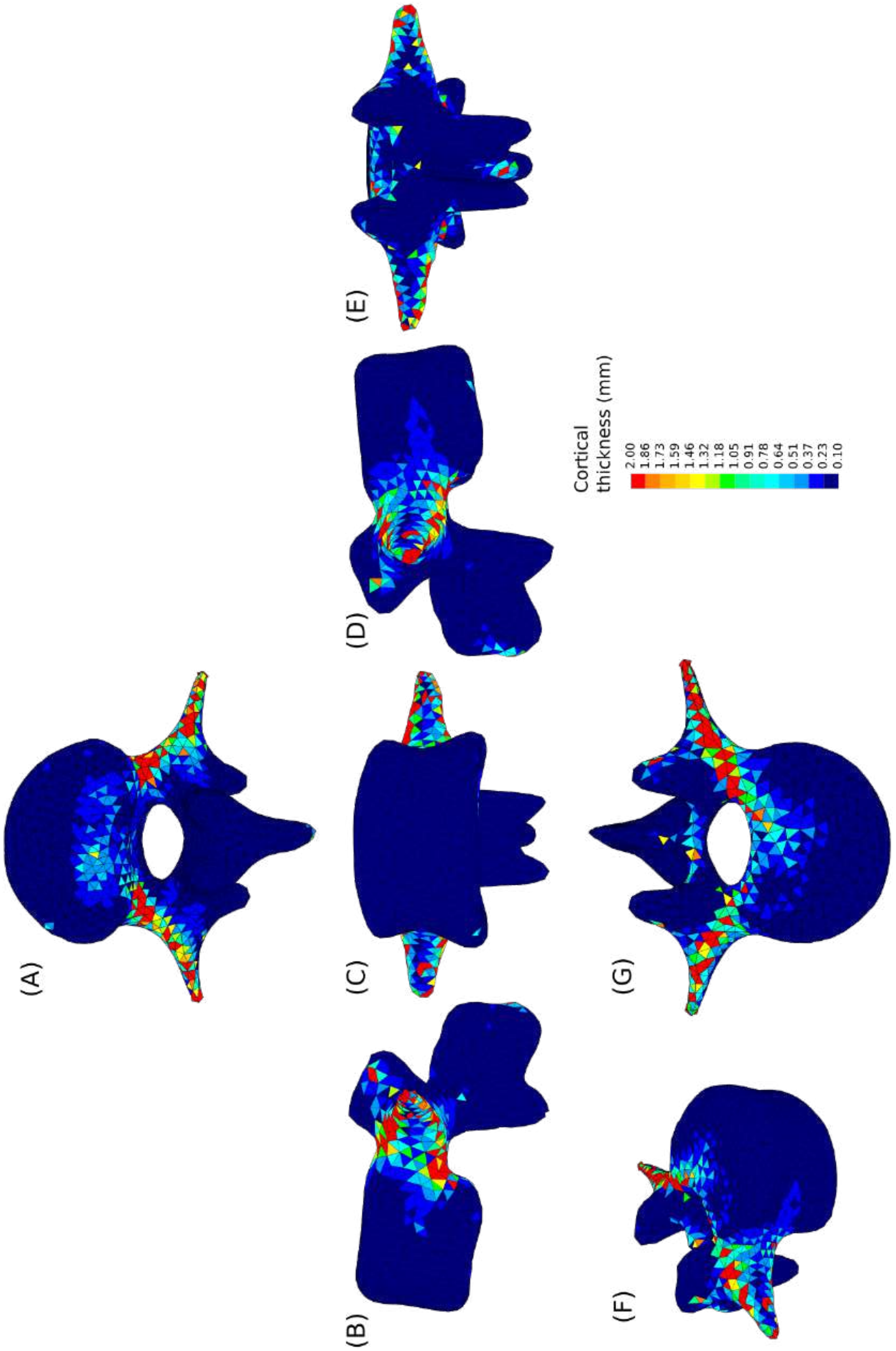

Figure S45: Cortical thickness ranging from 0.1 to 2 *mm* for the converged L3 model adapted to the healthy scenario. (A) caudal view, (B) left lateral view, (C) frontal view, (D) right lateral view, (E) dorsal view, (F) isometric view, (G) cranial view.

4.2.2 Trabecular architecture

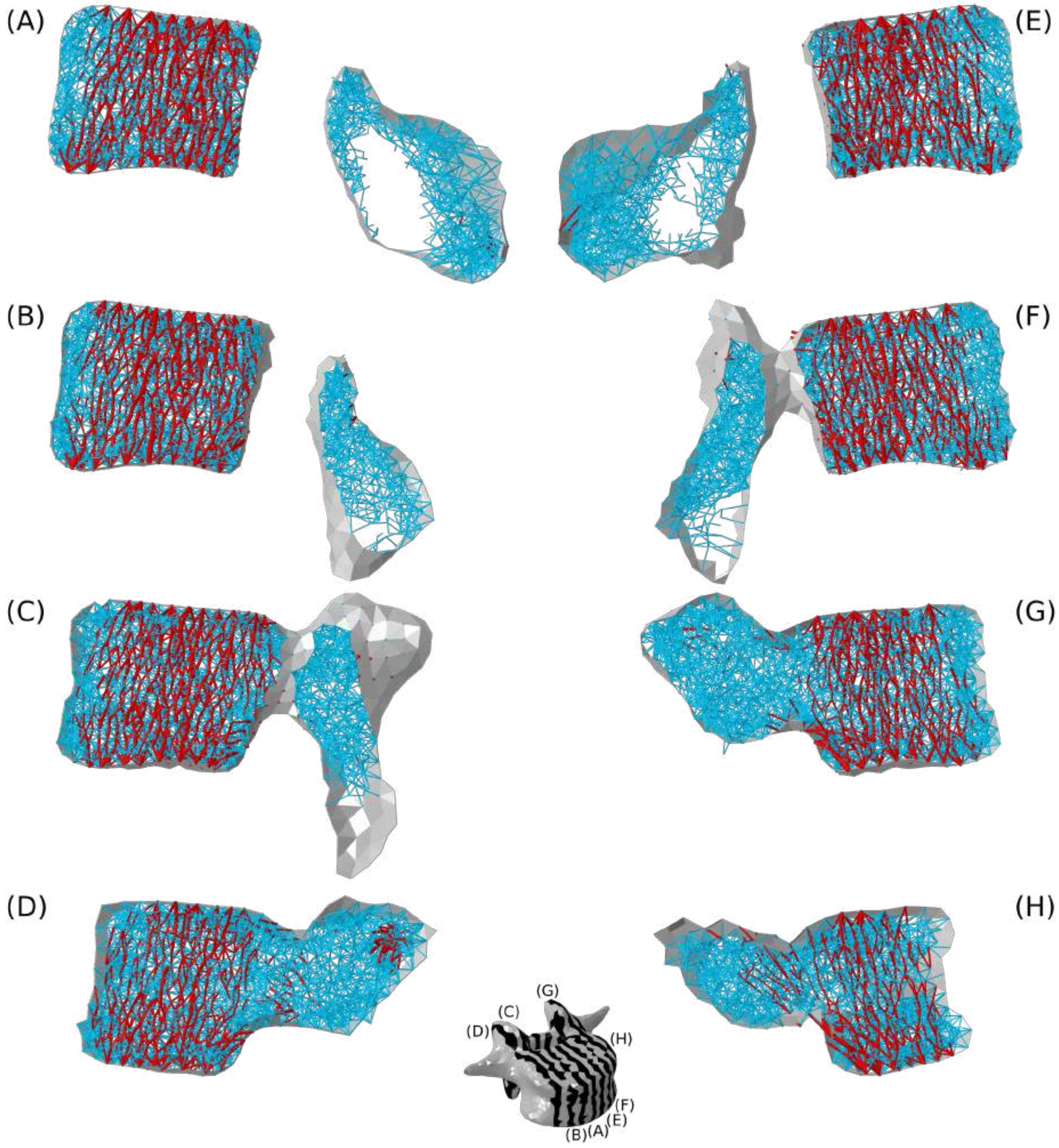

Figure S46: Selected sagittal 3 mm slices for the converged L3 model adapted to the healthy scenario. Cortical shell elements are shown in grey. Trabecular truss elements representing the secondary structure (with a radius of 0.1 mm) are shown in blue. Thicker truss elements representing the primary structure are shown in red. Truss elements in the dead zone (with a radius of 1 μm) are not shown for clarity.

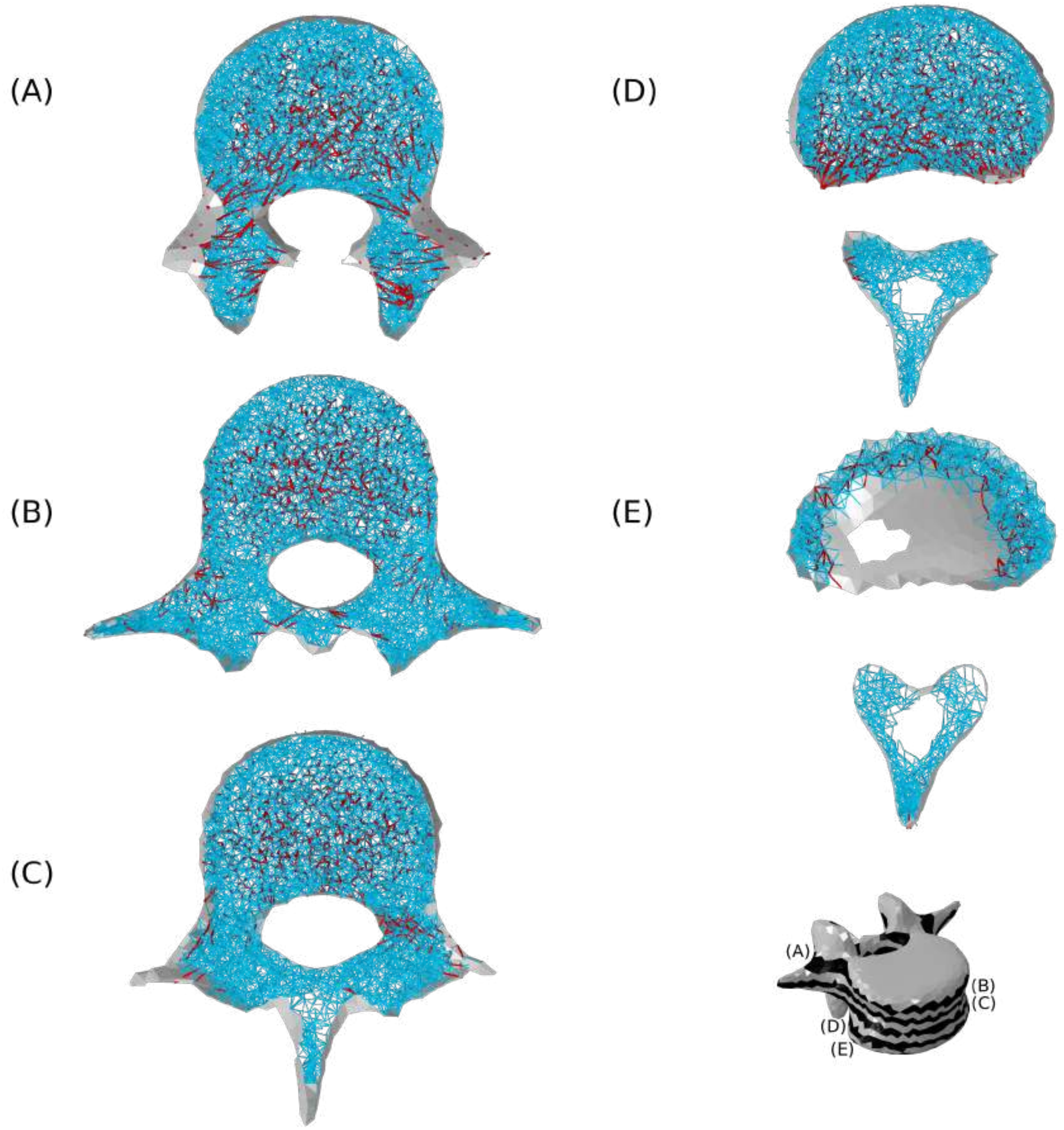

Figure S47: Selected transverse 3 mm slices for the converged L3 model adapted to the healthy scenario. Cortical shell elements are shown in grey. Trabecular truss elements representing the secondary structure (with a radius of 0.1 mm) are shown in blue. Thicker truss elements representing the primary structure are shown in red. Truss elements in the dead zone (with a radius of 1 μm) are not shown for clarity.

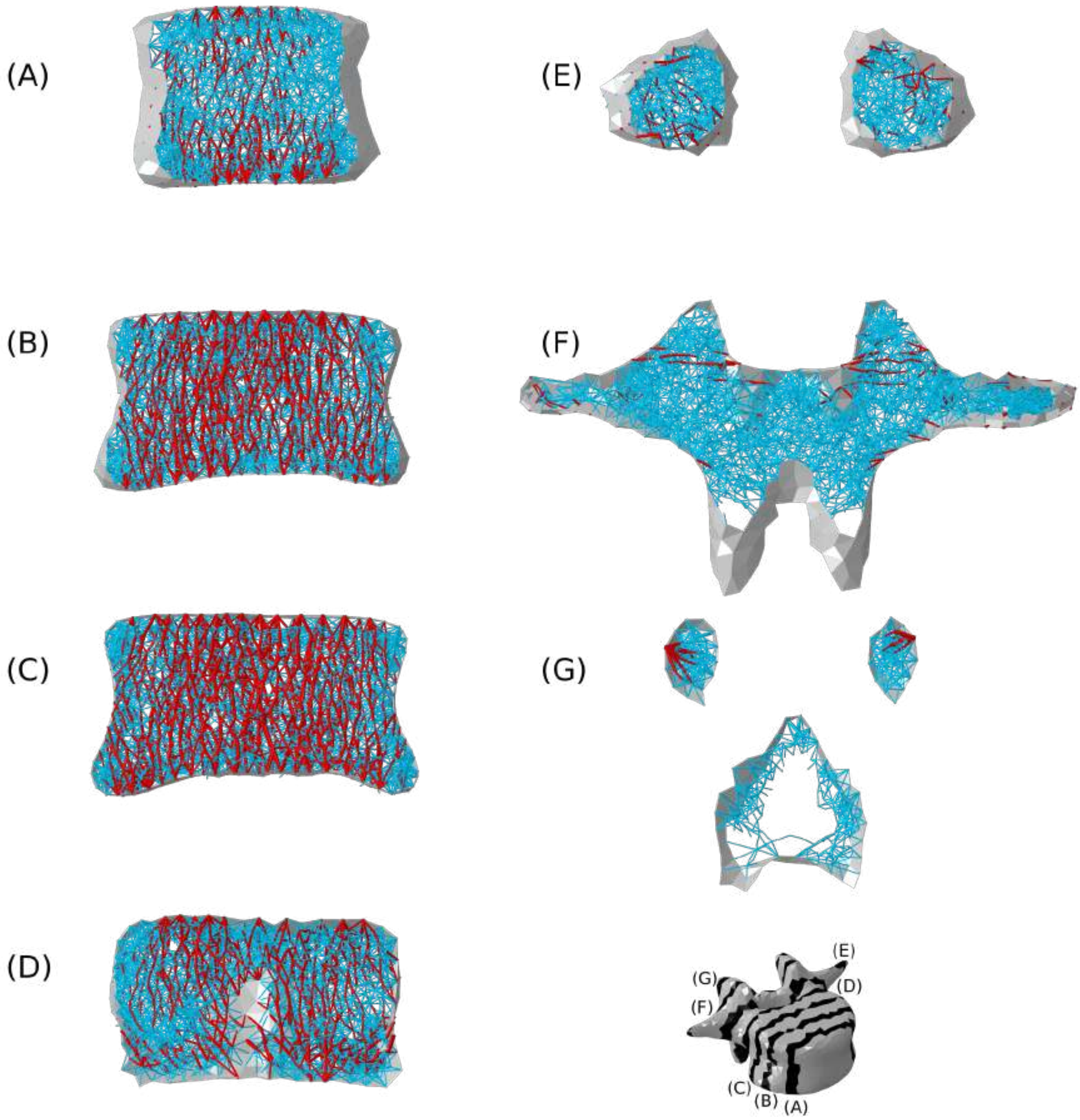

Figure S48: Selected coronal 3 mm slices for the converged L3 model adapted to the healthy scenario. Cortical shell elements are shown in grey. Trabecular truss elements representing the secondary structure (with a radius of 0.1 mm) are shown in blue. Thicker truss elements representing the primary structure are shown in red. Truss elements in the dead zone (with a radius of 1 μm) are not shown for clarity.

4.2.3 Contribution of each activities

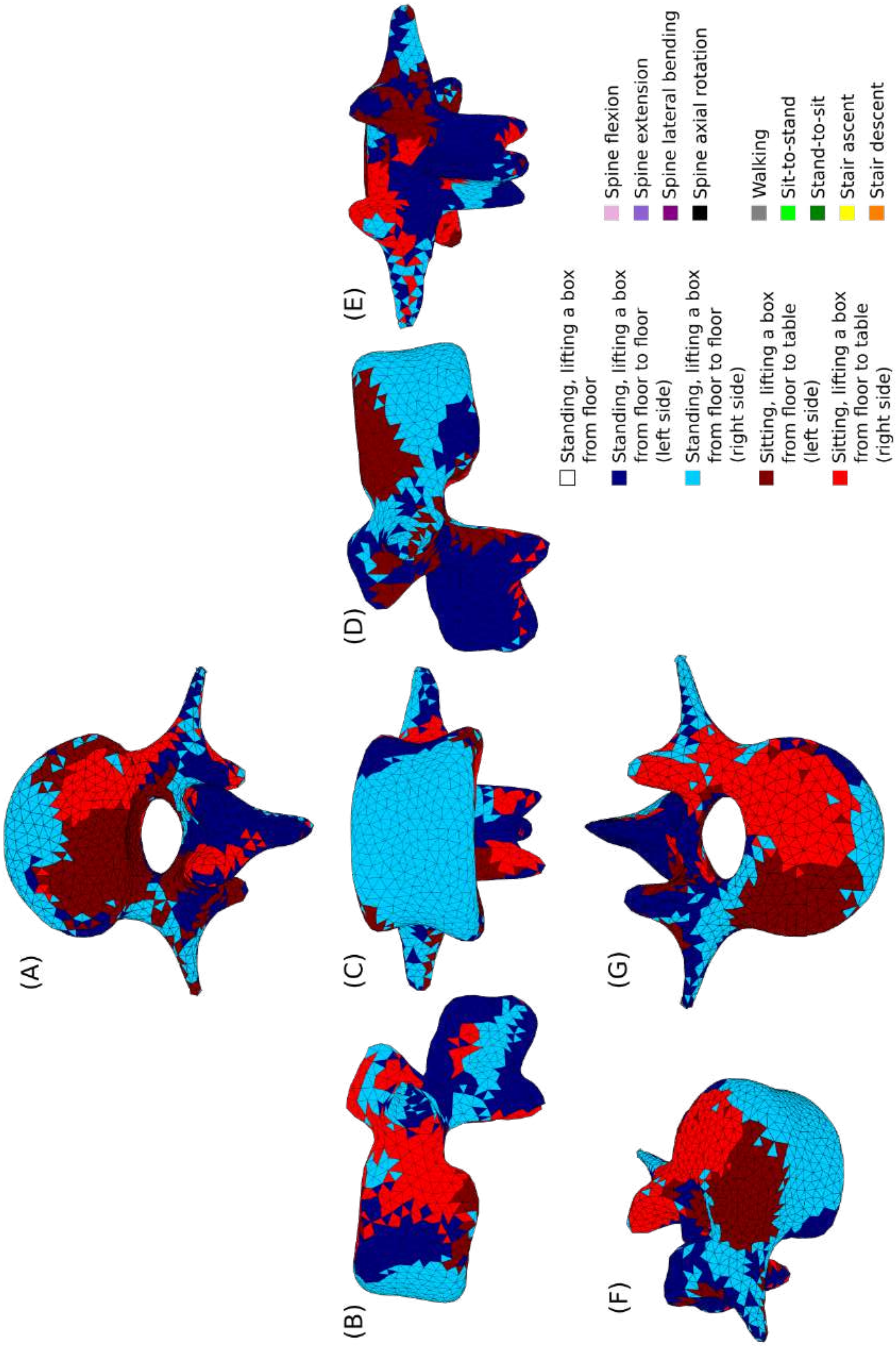

Figure S49: Contribution of each activity to the adaptation of the L3 cortical shells for the healthy scenario. (A) caudal view, (B) left lateral view, (C) frontal view, (D) right lateral view, (E) dorsal view, (F) isometric view, (G) cranial view.

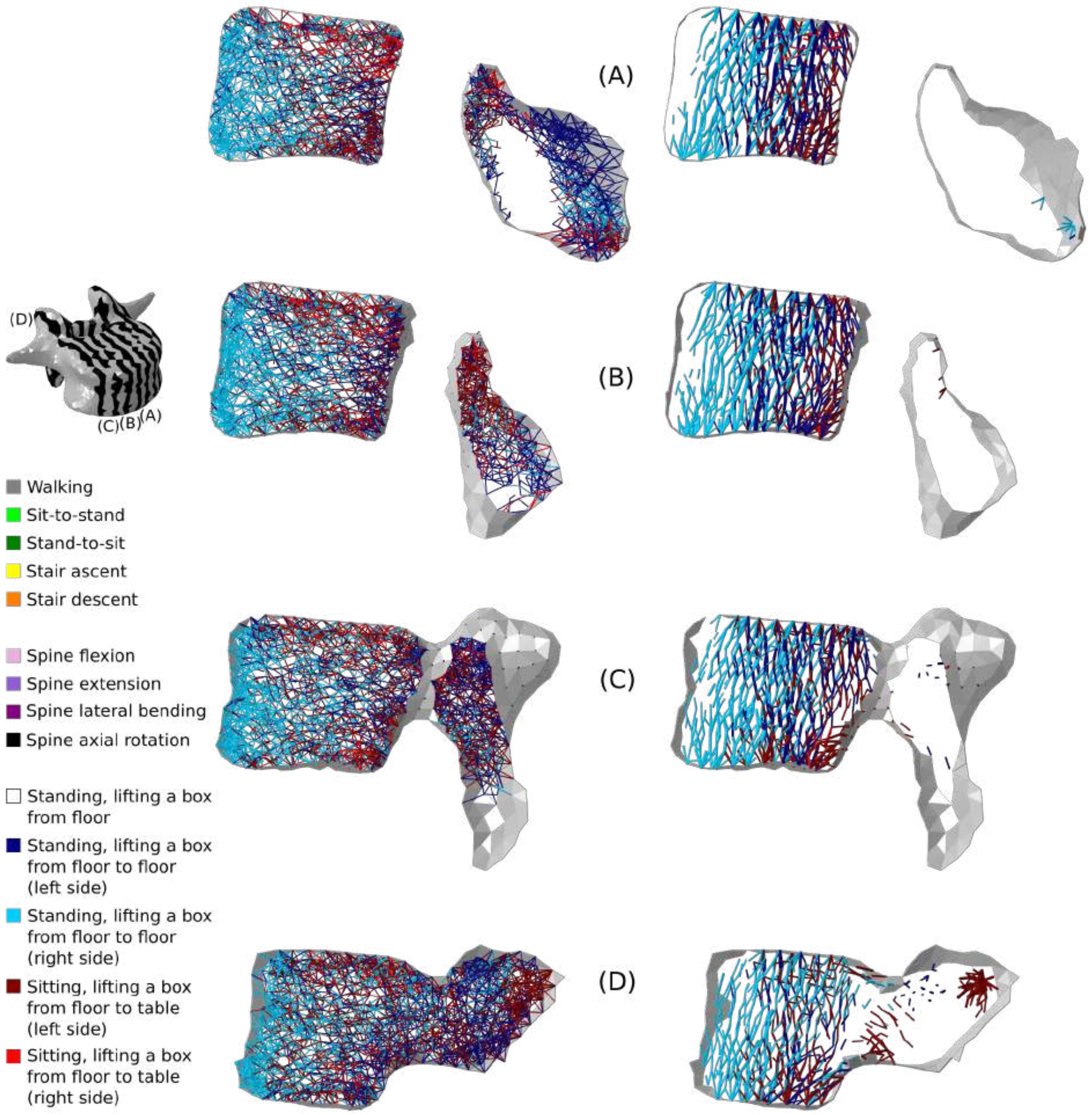

Figure S50: Selection of 3 *mm* sagittal slices of the right side of the converged L3 model adapted to the healthy scenario. Trabecular truss elements are colour mapped based on the activity most influential to their final geometry. On the left, only trabecular truss elements representing the secondary structure (with a radius of 0.1 *mm*) are shown. On the right, only trabecular truss elements representing the primary structure (with a radius superior to 0.1 *mm*) are shown. Cortical shell elements are sown in light grey.

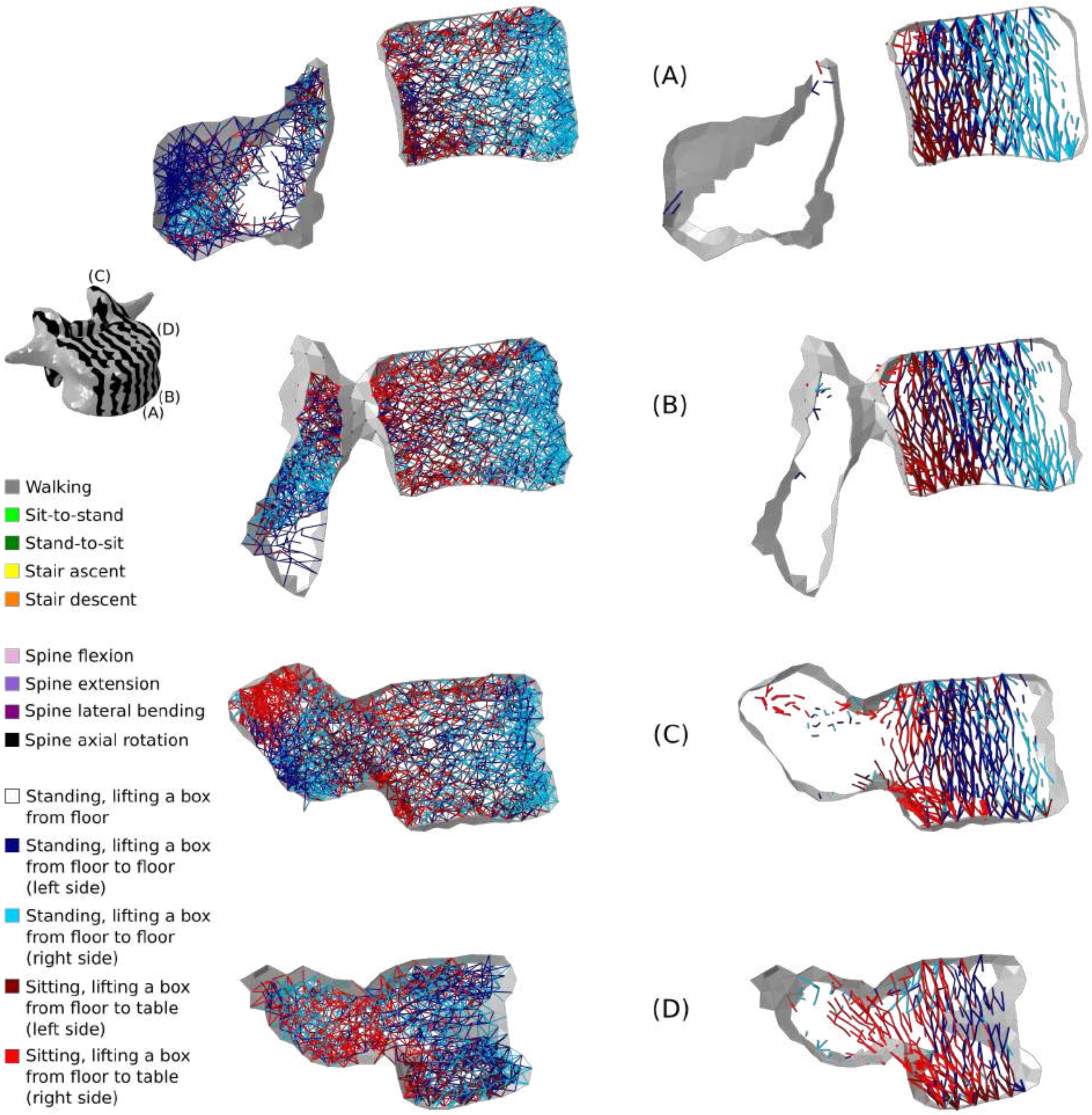

Figure S51: Selection of 3 *mm* sagittal slices of the left side of the converged L3 model adapted to the healthy scenario. Trabecular truss elements are colour mapped based on the activity most influential to their final geometry. On the left, only trabecular truss elements representing the secondary structure (with a radius of 0.1 *mm*) are shown. On the right, only trabecular truss elements representing the primary structure (with a radius superior to 0.1 *mm*) are shown. Cortical shell elements are sown in light grey.

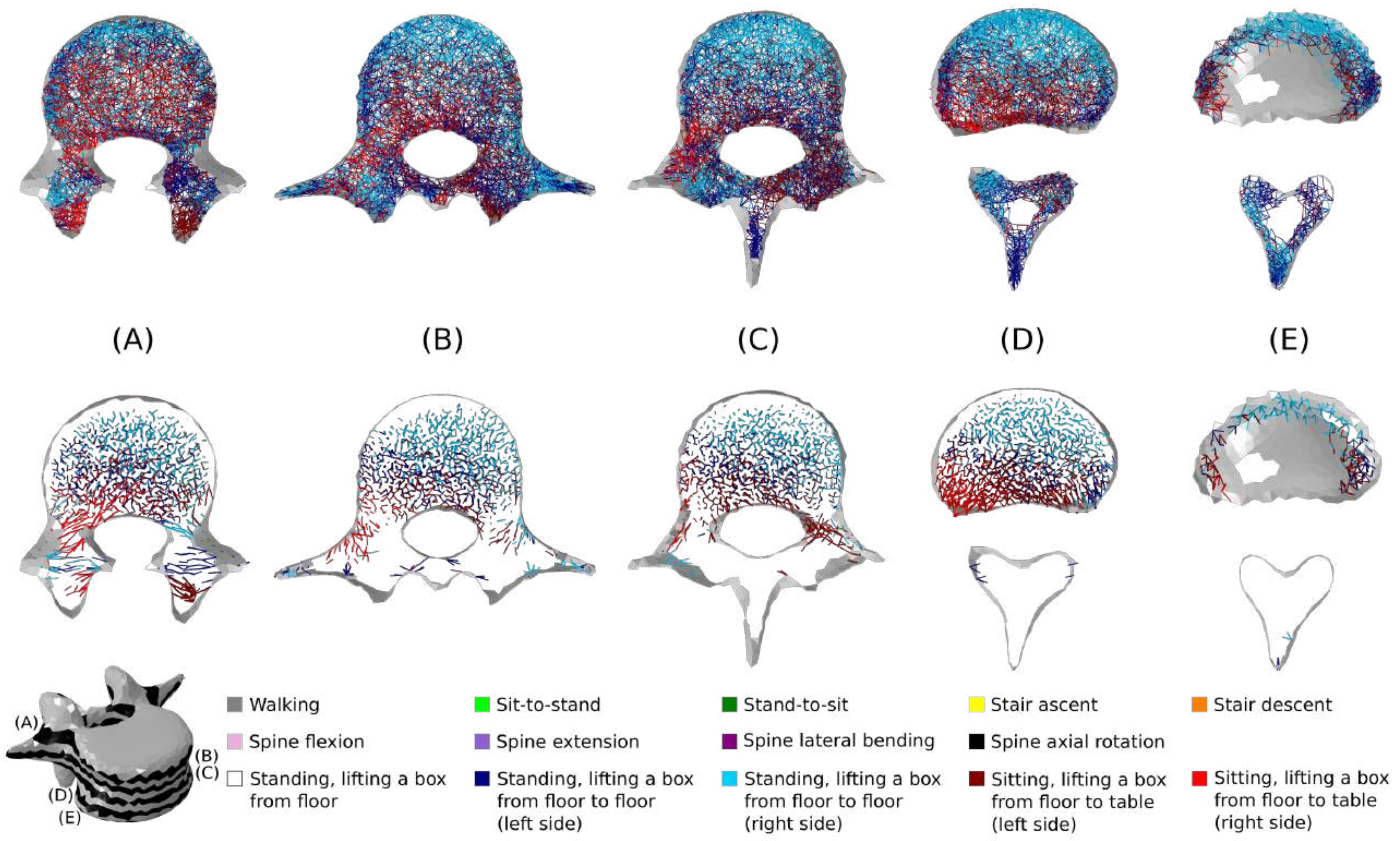

Figure S52: Selection of 3 mm transverse slices of the converged L3 model adapted to the healthy scenario. Trabecular truss elements are colour mapped based on the activity most influential to their final geometry. On the top row, only trabecular truss elements representing the secondary structure (with a radius of 0.1 mm) are shown. On the bottom row, only trabecular truss elements with a radius superior to 0.1 mm are shown. Cortical shell elements are sown in light grey.

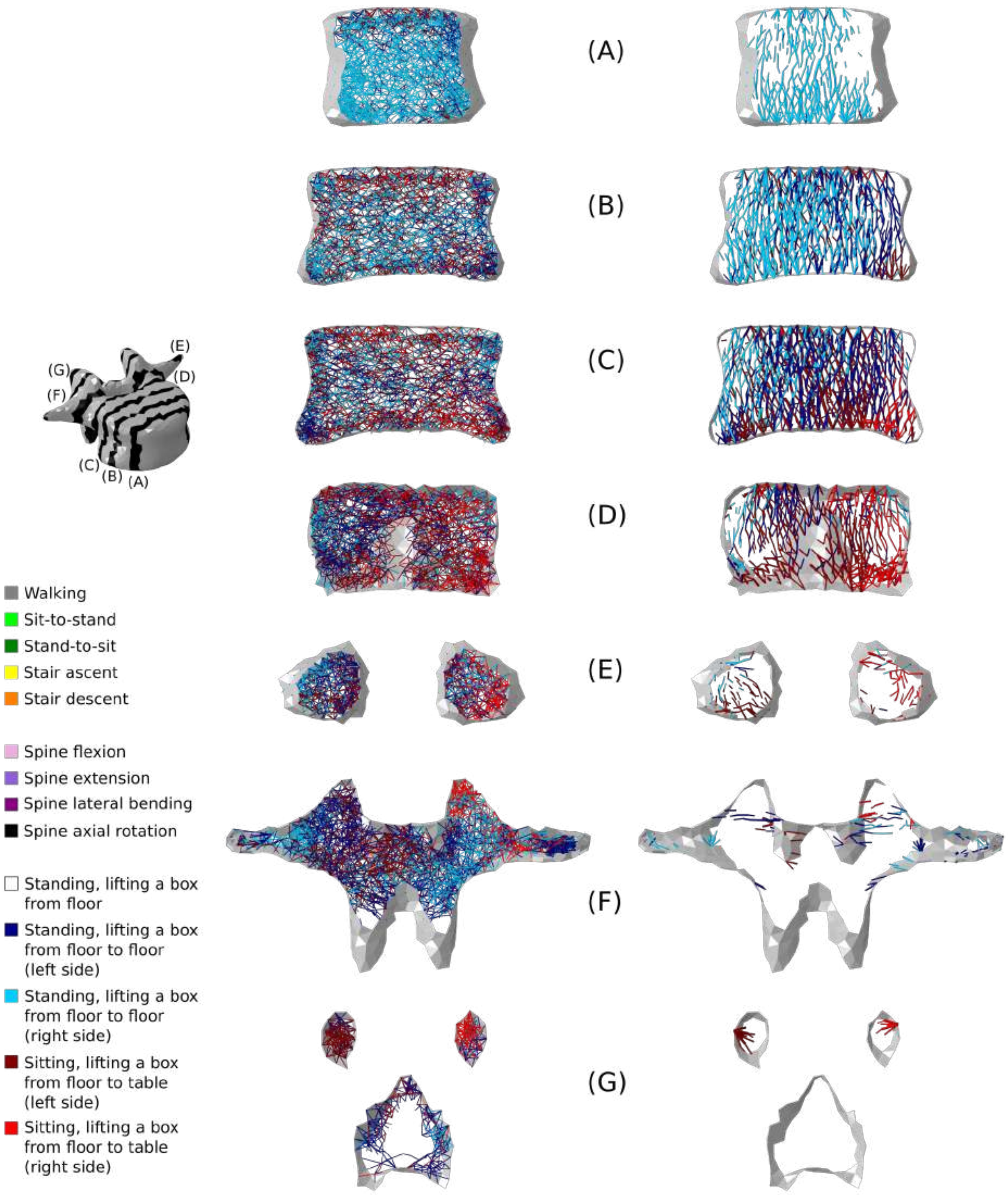

Figure S53: Selection of 3 *mm* coronal slices of the converged L3 model adapted to the healthy scenario. Trabecular truss elements are colour mapped based on the activity most influential to their final geometry. On the left, only trabecular truss elements representing the secondary structure (with a radius of 0.1 *mm*) are shown. On the right, only trabecular truss elements representing the primary structure (with a radius superior to 0.1 *mm*) are shown. Cortical shell elements are sown in light grey.

4.2.4 Characterisation of trabecular anisotropy

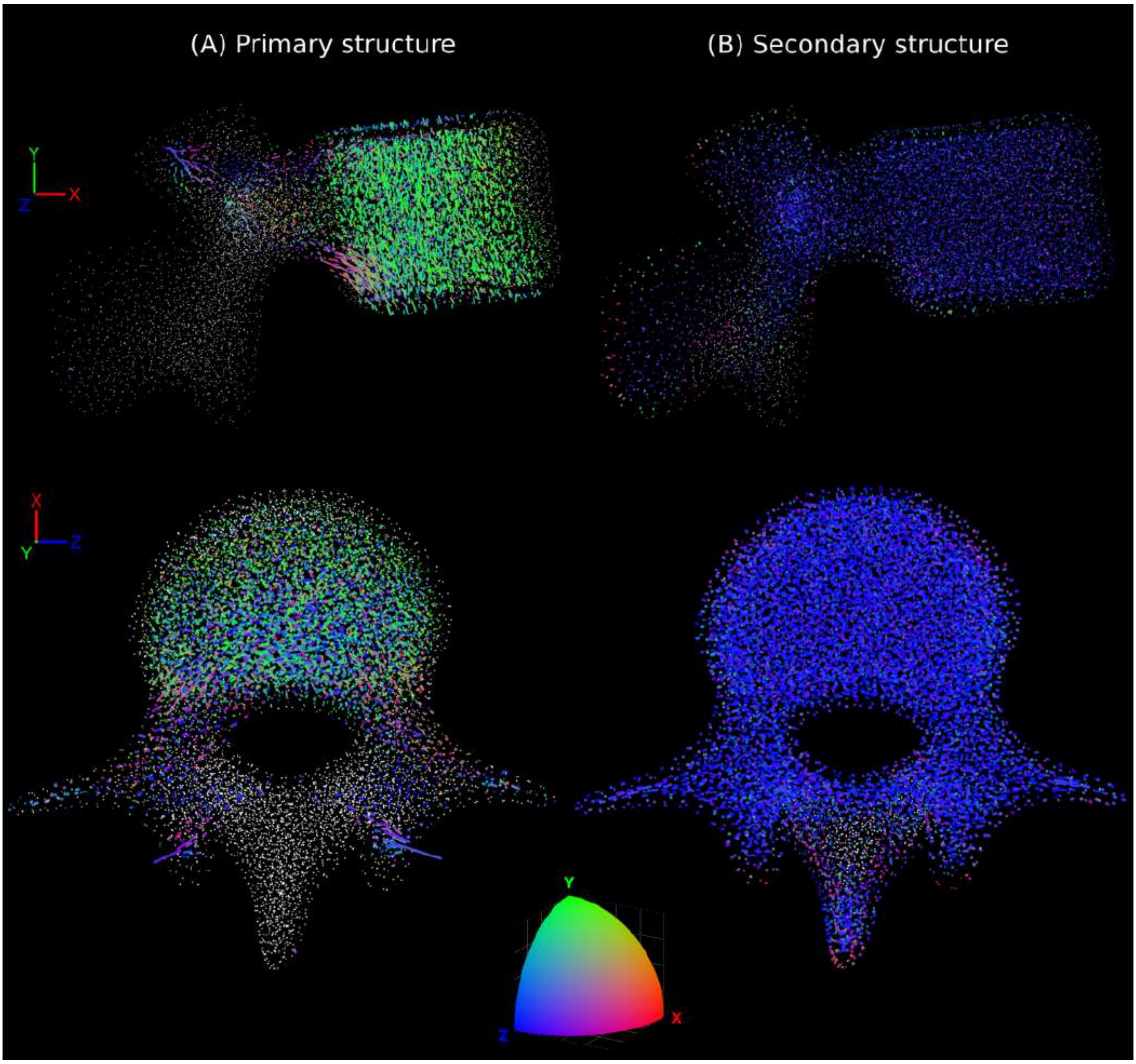

Figure S54: Characterisation of anisotropy in the trabecular bone of L3 adapted to the healthy scenario. (A) shows the orientation of the trabecular trusses of the primary structure (with a radius larger than 0.1 mm). (B) shows the orientation of the trabecular trusses of the secondary structure (with a radius of 0.1 mm). For (A) and (B), side view (top row) and top view (bottom row) are shown. Lines are attached to each trabecular node, with colour and length varying respectively with the orientations and radii of the truss elements connected to that particular node. The colour scale at the bottom shows how the colour of the lines should be interpreted. Orientation along the X, Y or Z axes are in red, green or blue respectively. Any orientation that is not colinear with these axes shows as a combination of red, green and blue. A white dot indicates a node without elements in the size range being looked at connected to it.

4.3 Sedentary scenario

4.3.1 Cortical thickness

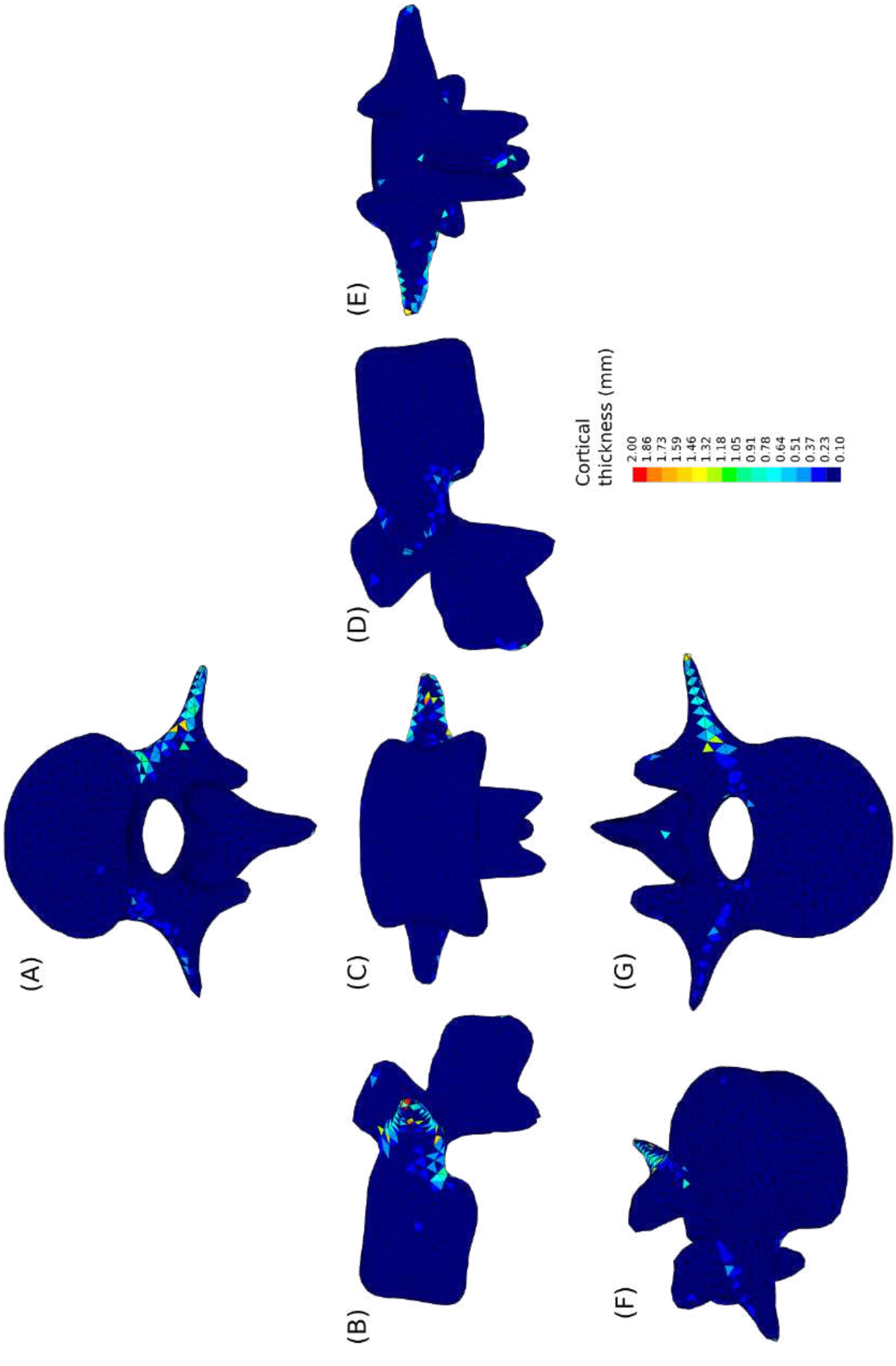

Figure S55: Cortical thickness ranging from 0.1 to 2 *mm* for the converged L3 model adapted to the sedentary scenario. (A) caudal view, (B) left lateral view, (C) frontal view, (D) right lateral view, (E) dorsal view, (F) isometric view, (G) cranial view.

4.3.2 Trabecular architecture

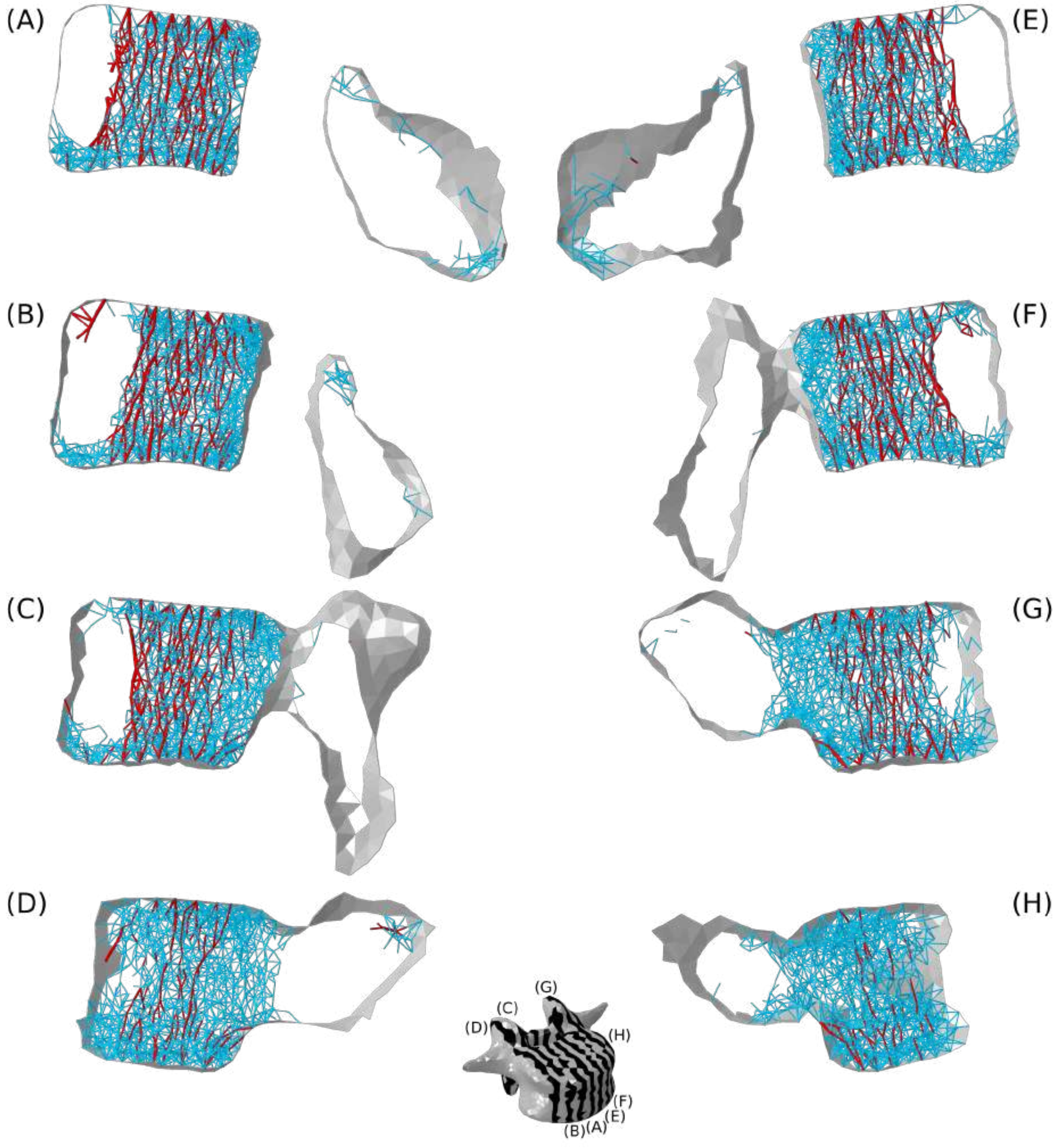

Figure S56: Selected sagittal 3 mm slices for the converged L3 model adapted to the sedentary scenario. Cortical shell elements are shown in grey. Trabecular truss elements representing the secondary structure (with a radius of 0.1 mm) are shown in blue. Thicker truss elements representing the primary structure are shown in red. Truss elements in the dead zone (with a radius of 1 μm) are not shown for clarity.

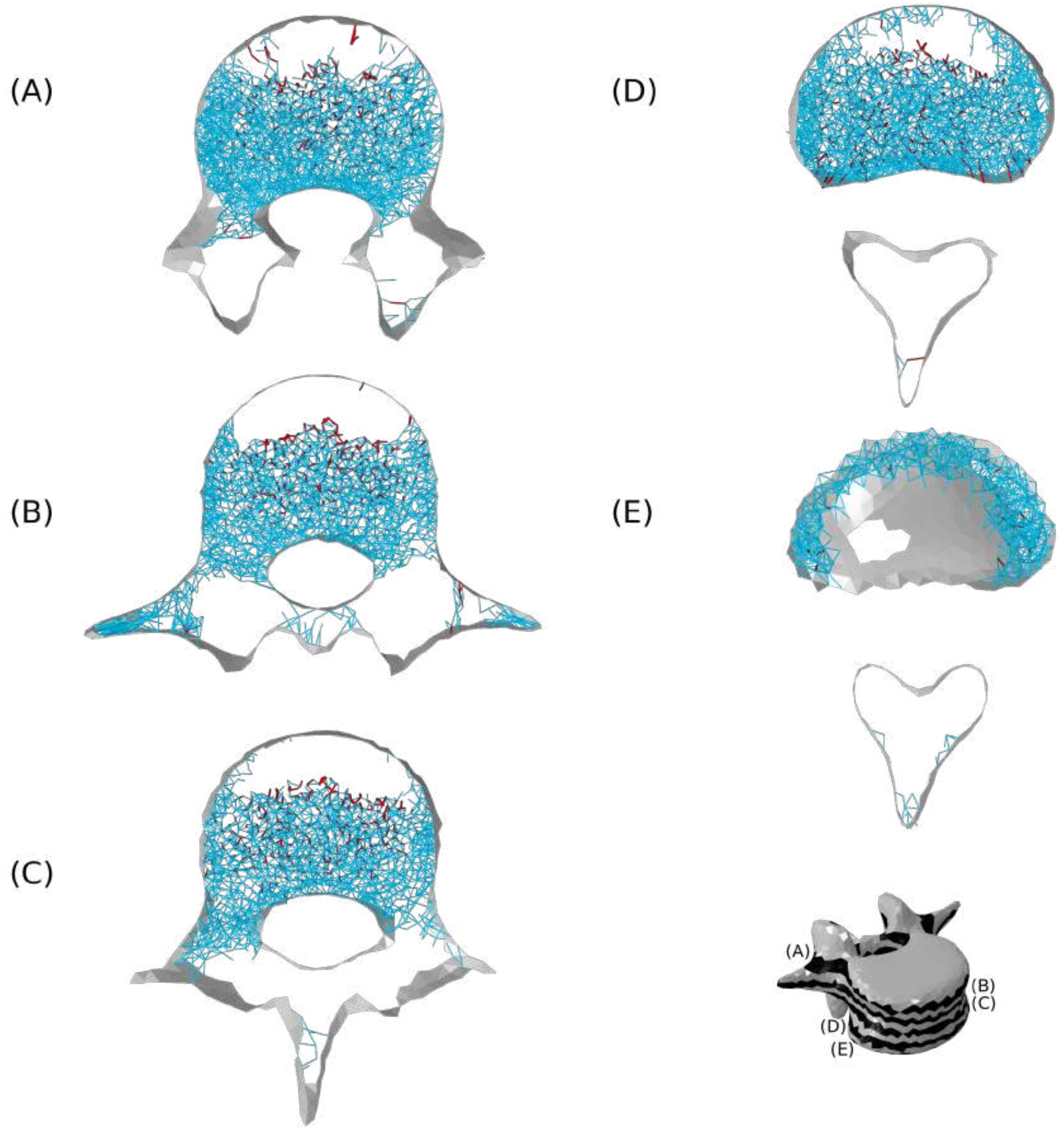

Figure S57: Selected transverse 3 mm slices for the converged L3 model adapted to the sedentary scenario. Cortical shell elements are shown in grey. Trabecular truss elements representing the secondary structure (with a radius of 0.1 mm) are shown in blue. Thicker truss elements representing the primary structure are shown in red. Truss elements in the dead zone (with a radius of 1 μm) are not shown for clarity.

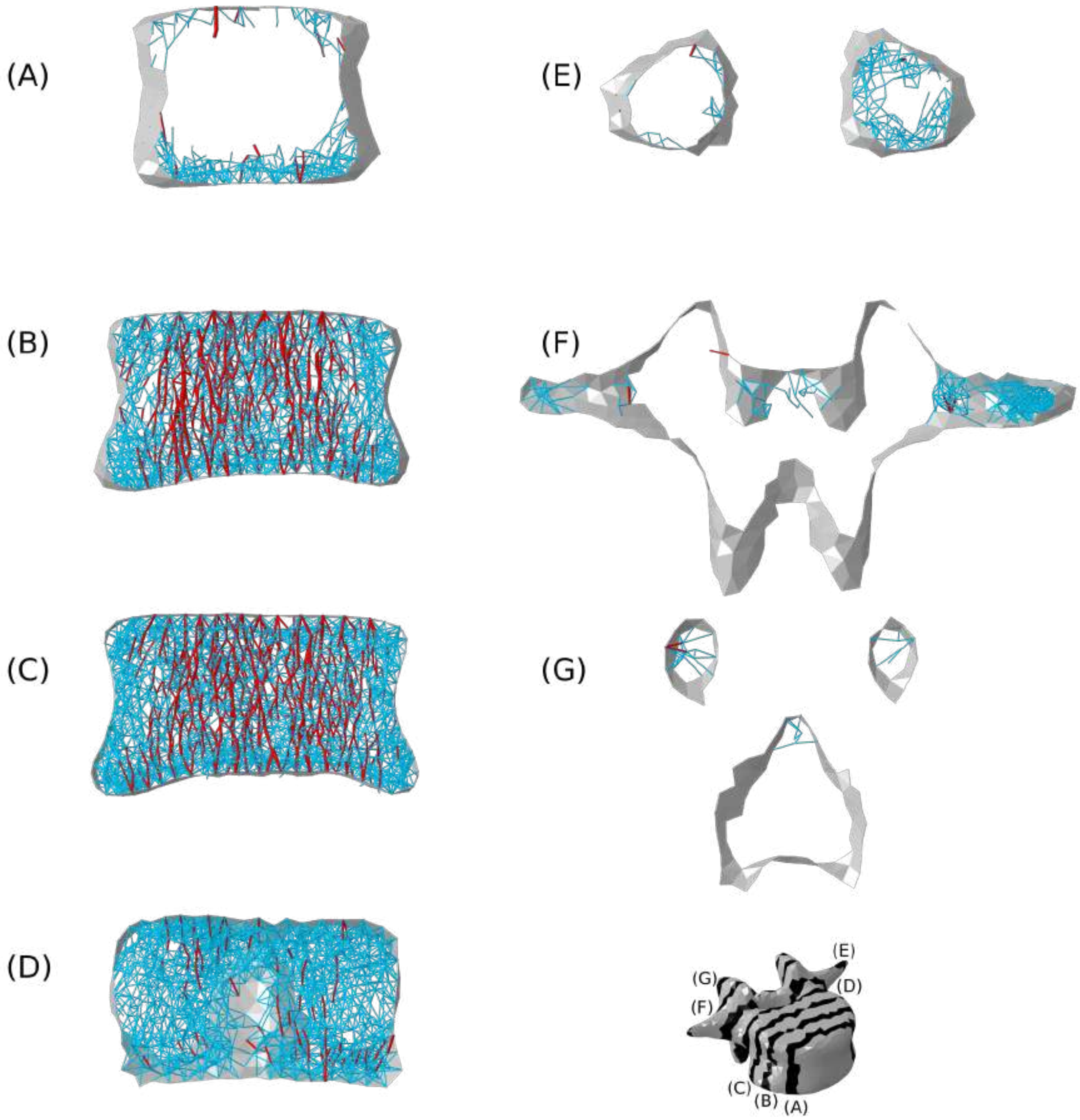

Figure S58: Selected coronal 3 *mm* slices for the converged L3 model adapted to the sedentary scenario. Cortical shell elements are shown in grey. Trabecular truss elements representing the secondary structure (with a radius of 0.1 *mm*) are shown in blue. Thicker truss elements representing the primary structure are shown in red. Truss elements in the dead zone (with a radius of 1 μm) are not shown for clarity.

4.3.3 Contribution of each activities

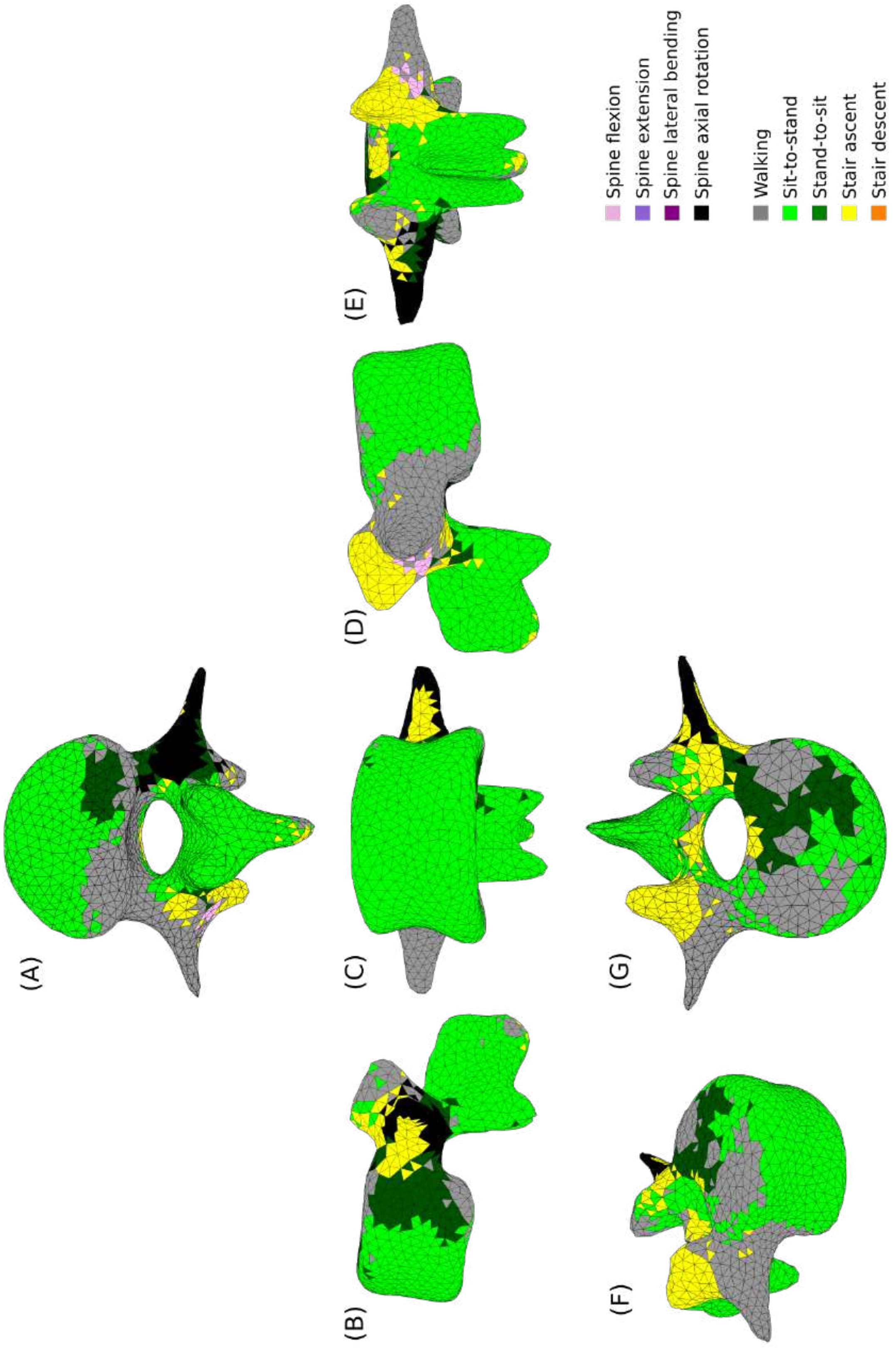

Figure S59: Contribution of each activity to the adaptation of the L3 cortical shells for the sedentary scenario. (A) caudal view, (B) left lateral view, (C) frontal view, (D) right lateral view, (E) dorsal view, (F) isometric view, (G) cranial view.

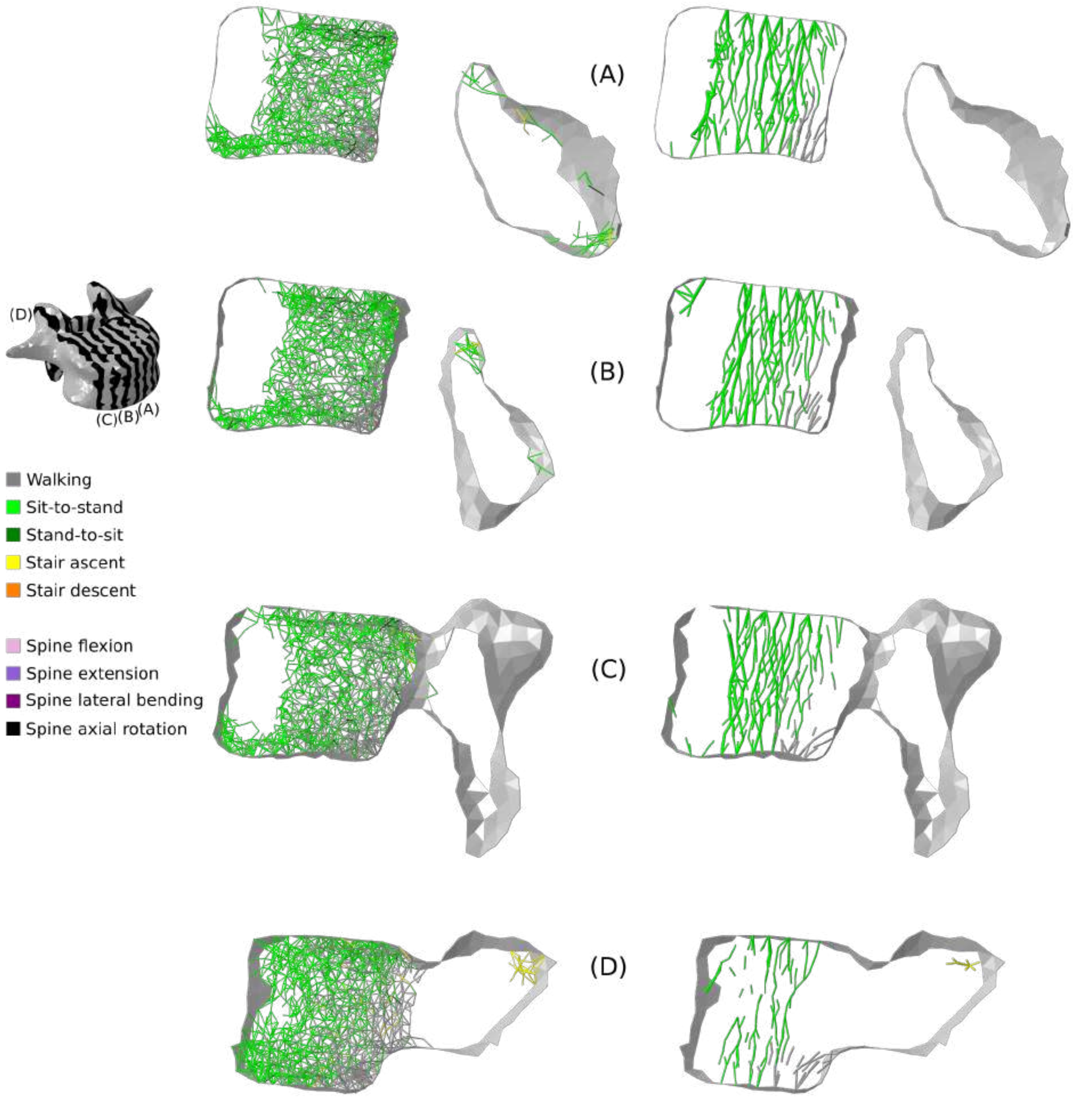

Figure S60: Selection of 3 mm sagittal slices of the right side of the converged L3 model adapted to the sedentary scenario. Trabecular truss elements are colour mapped based on the activity most influential to their final geometry. On the left, only trabecular truss elements representing the secondary structure (with a radius of 0.1 mm) are shown. On the right, only trabecular truss elements representing the primary structure (with a radius superior to 0.1 mm) are shown. Cortical shell elements are sown in light grey.

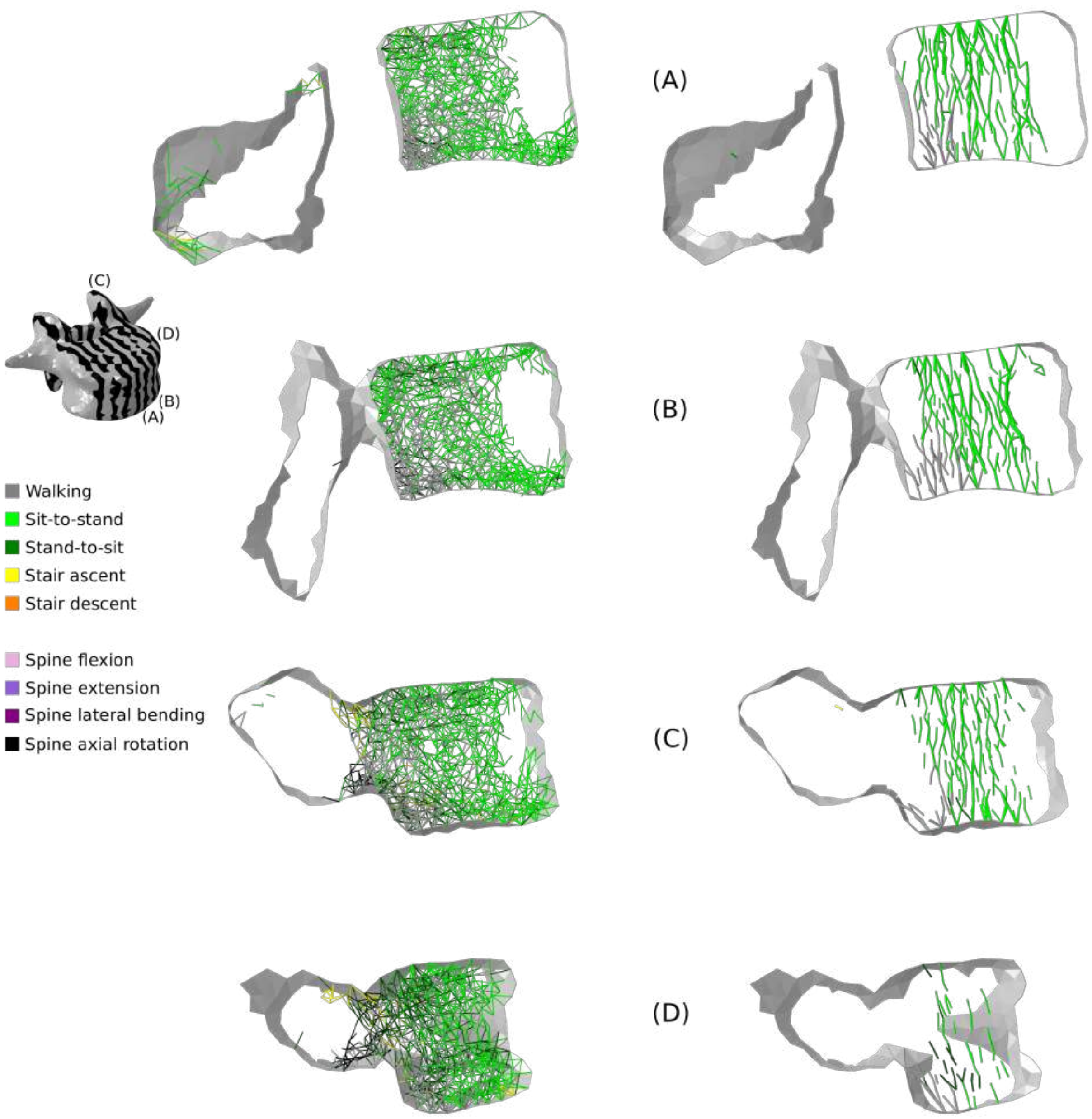

Figure S61: Selection of 3 *mm* sagittal slices of the left side of the converged L3 model adapted to the sedentary scenario. Trabecular truss elements are colour mapped based on the activity most influential to their final geometry. On the left, only trabecular truss elements representing the secondary structure (with a radius of 0.1 *mm*) are shown. On the right, only trabecular truss elements representing the primary structure (with a radius superior to 0.1 *mm*) are shown. Cortical shell elements are sown in light grey.

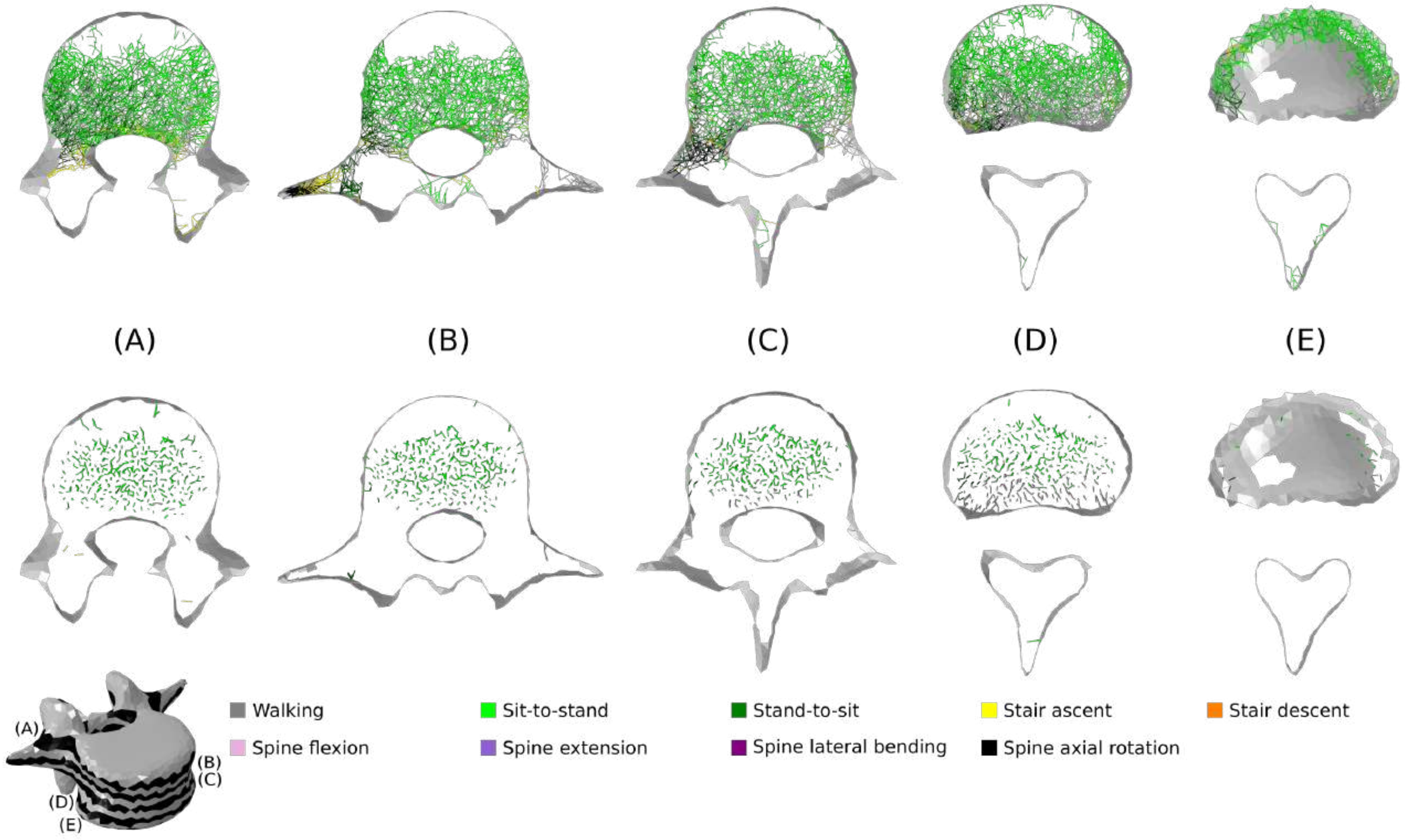

Figure S62: Selection of 3 mm transverse slices of the converged L3 model adapted to the sedentary scenario. Trabecular truss elements are colour mapped based on the activity most influential to their final geometry. On the top row, only trabecular truss elements representing the secondary structure (with a radius of 0.1 mm) are shown. On the bottom row, only trabecular truss elements with a radius superior to 0.1 mm are shown. Cortical shell elements are sown in light grey.

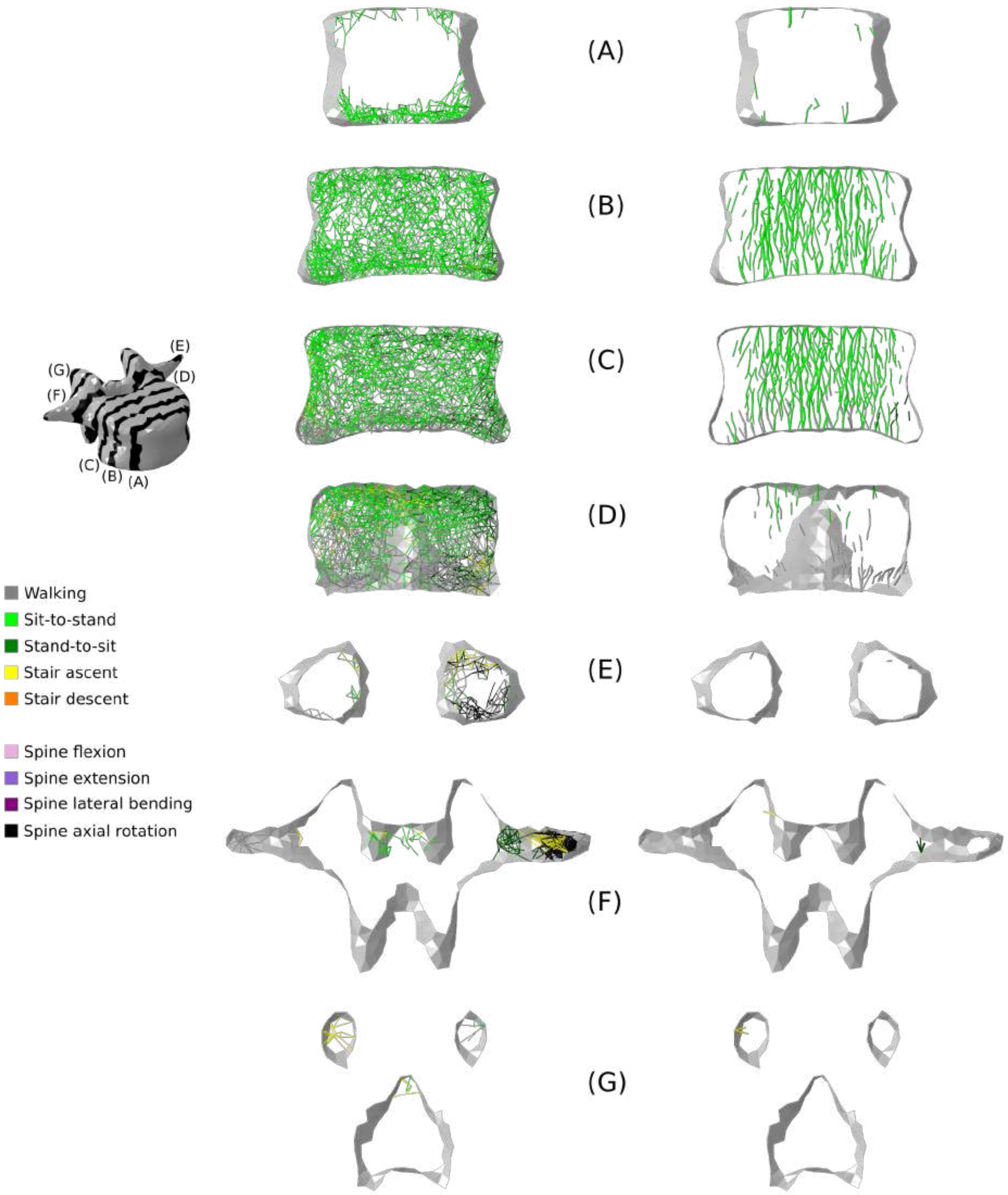

Figure S63: Selection of 3 mm coronal slices of the converged L3 model adapted to the sedentary scenario. Trabecular truss elements are colour mapped based on the activity most influential to their final geometry. On the left, only trabecular truss elements representing the secondary structure (with a radius of 0.1 mm) are shown. On the right, only trabecular truss elements representing the primary structure (with a radius superior to 0.1 mm) are shown. Cortical shell elements are sown in light grey.

5 FOURTH LUMBAR VERTEBRA

5.1 Load cases

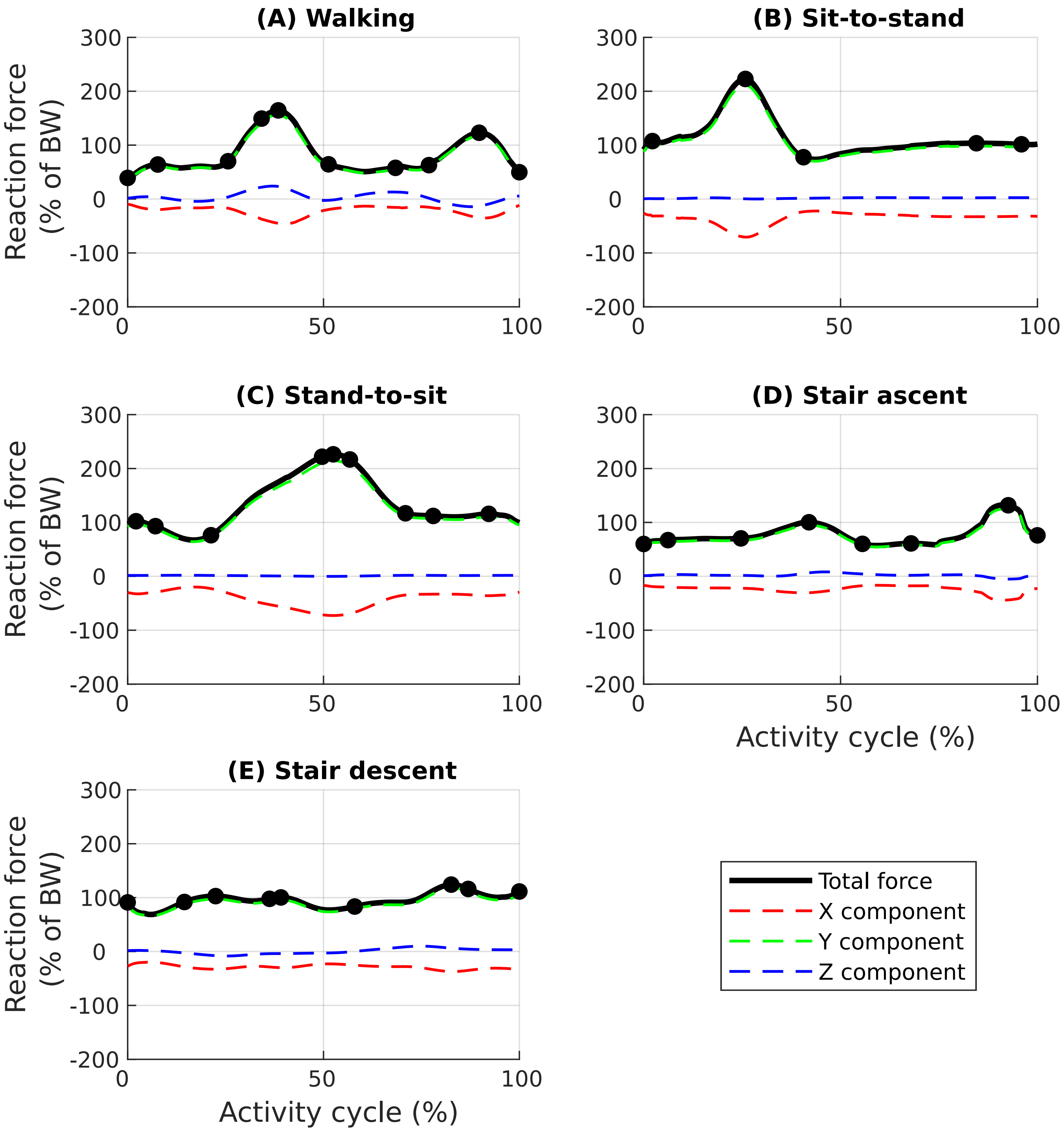

Figure S64: Selection of load cases for L4, for five activities related to locomotion ((A) level walking, (B) standing up from a chair, (C) sitting down on a chair, (D) walking up the stairs, and (E) walking down the stairs). Total reaction force at L4-L5 joint derived from the musculoskeletal model and normalised to body weight (BW) are shown as black lines. Dots indicate the frames selected for the finite element analysis. Red, green and blue dashed lines show X, Y and Z components of the reaction force expressed in the vertebra coordinate system.

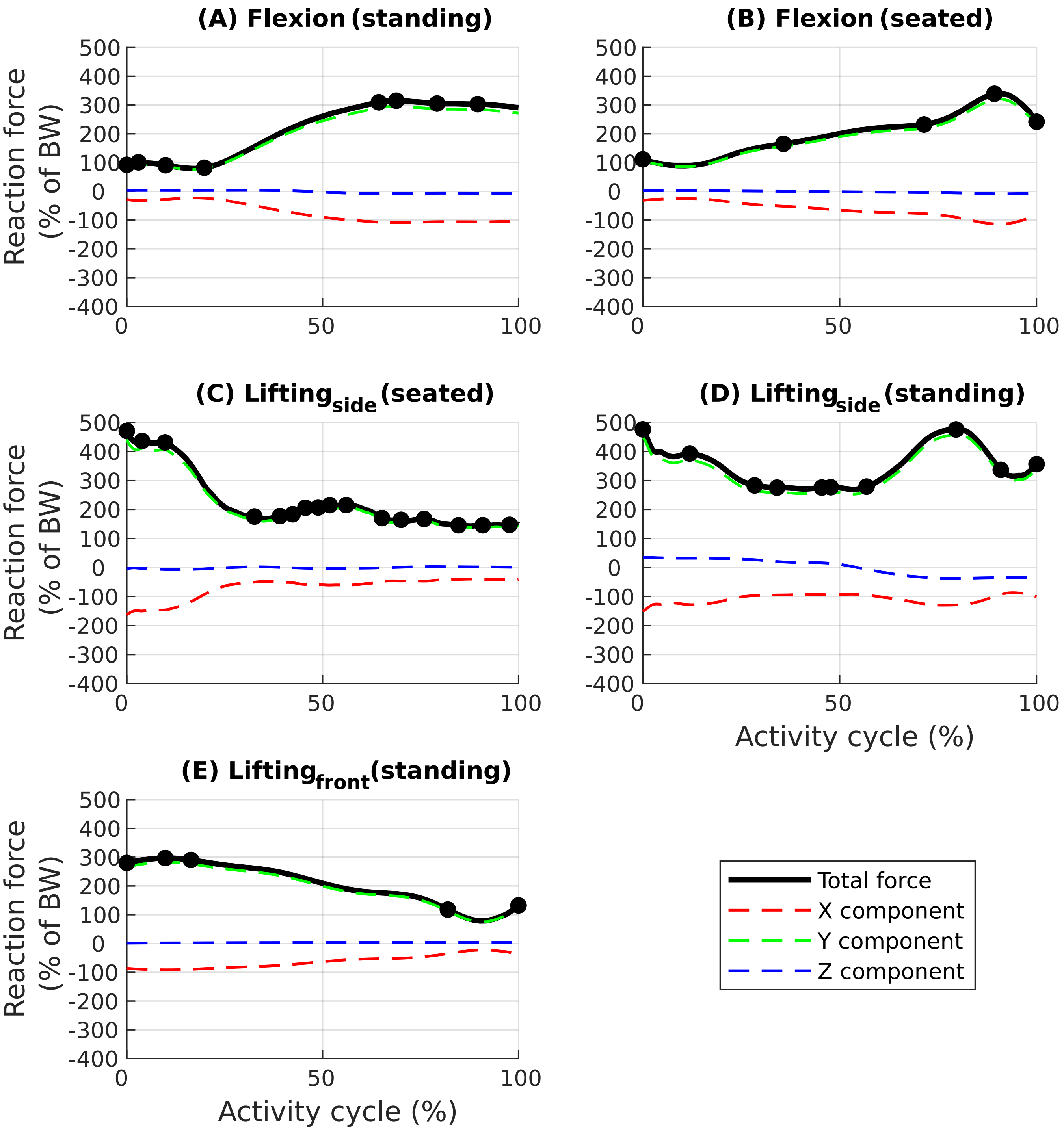

Figure S65: Selection of load cases for L4, for seven activities involving spine movements ((A) forward flexion from upright standing to maximum flexion, (B) forward flexion from upright sitting, (C) in a seated position, twisting and lifting a box from the floor, from the right side to a table in front, (D) in a standing position, twisting and lifting a box from the floor, from the left side to the right side, and (E) in a standing position, lifting a box from the floor in front to the chest). Total reaction force at L4-L5 joint derived from the musculoskeletal model and normalised to body weight (BW) are shown as black lines. Dots indicate the frames selected for the finite element analysis. Load cases obtained with activities (C) and (D) were mirrored through the sagittal plane to obtain loading on the other side. Red, green and blue dashed lines show X, Y and Z components of the reaction force expressed in the vertebra coordinate system.

5.2 Healthy scenario

5.2.1 Cortical thickness

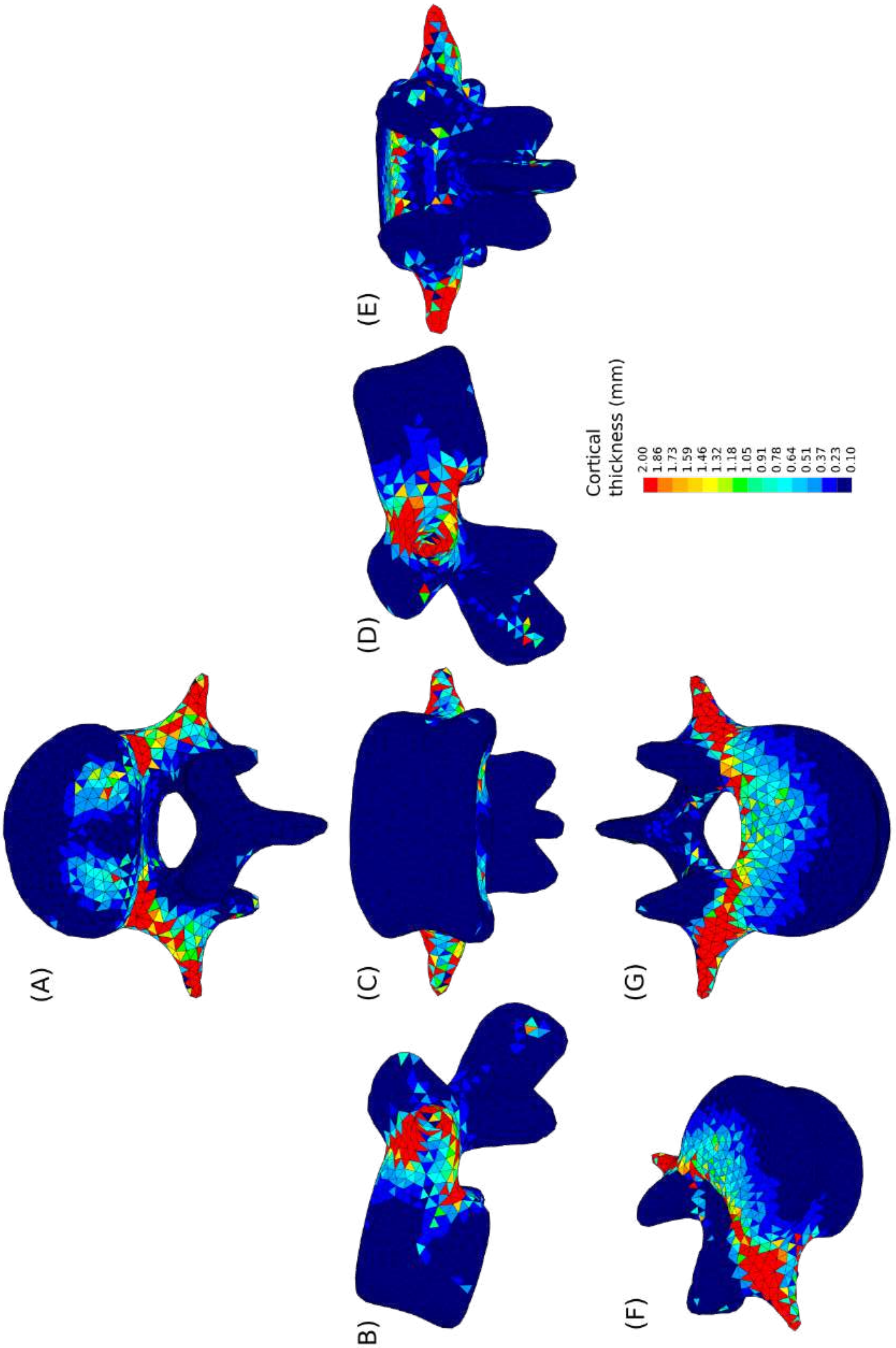

Figure S66: Cortical thickness ranging from 0.1 to 2 *mm* for the converged L4 model adapted to the healthy scenario. (A) caudal view, (B) left lateral view, (C) frontal view, (D) right lateral view, (E) dorsal view, (F) isometric view, (G) cranial view.

5.2.2 Trabecular architecture

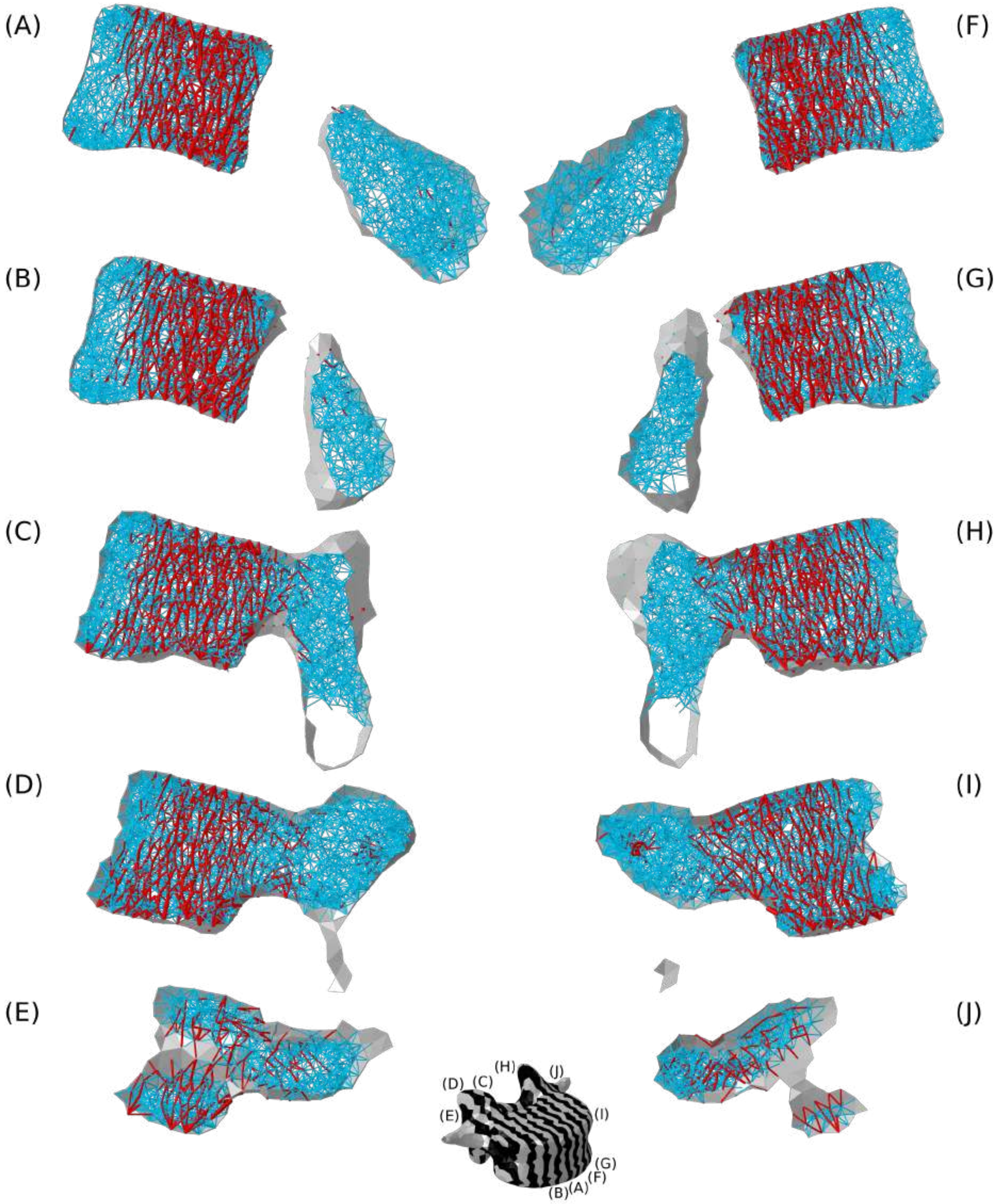

Figure S67: Selected sagittal 3 mm slices for the converged L4 model adapted to the healthy scenario. Cortical shell elements are shown in grey. Trabecular truss elements representing the secondary structure (with a radius of 0.1 mm) are shown in blue. Thicker truss elements representing the primary structure are shown in red. Truss elements in the dead zone (with a radius of 1 μm) are not shown for clarity.

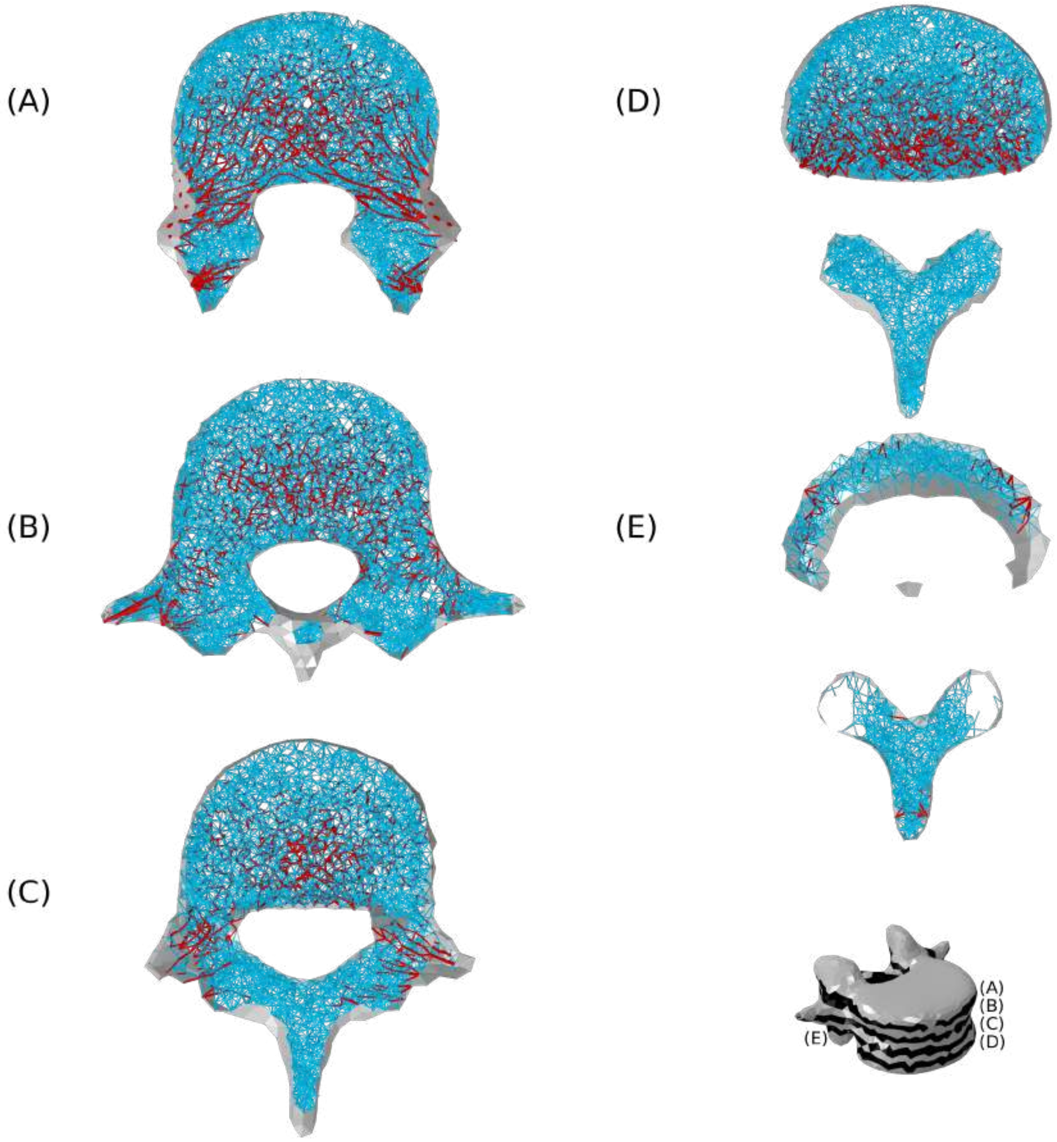

Figure S68: Selected transverse 3 mm slices for the converged L4 model adapted to the healthy scenario. Cortical shell elements are shown in grey. Trabecular truss elements representing the secondary structure (with a radius of 0.1 mm) are shown in blue. Thicker truss elements representing the primary structure are shown in red. Truss elements in the dead zone (with a radius of 1 μm) are not shown for clarity.

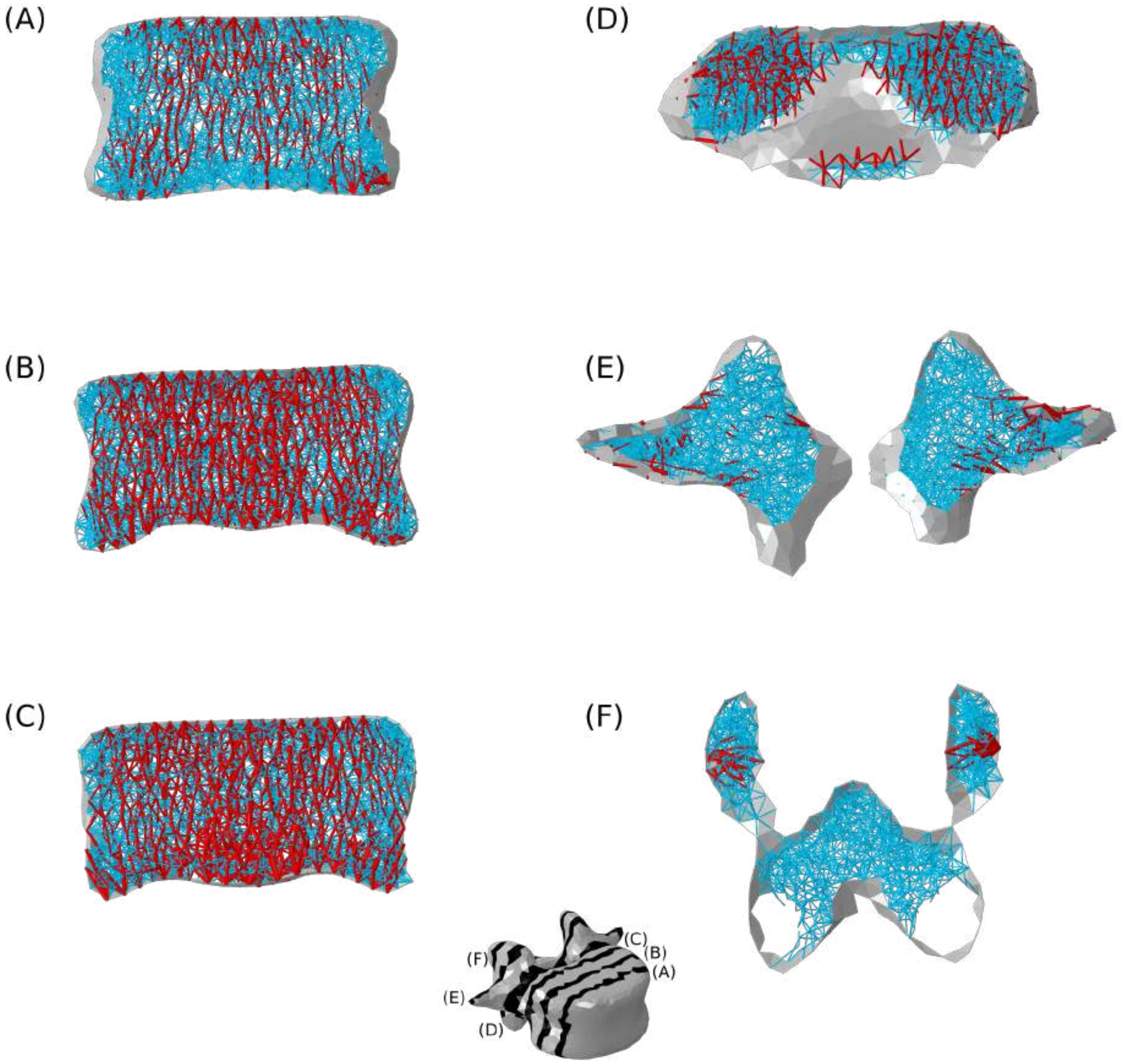

Figure S69: Selected coronal 3 mm slices for the converged L4 model adapted to the healthy scenario. Cortical shell elements are shown in grey. Trabecular truss elements representing the secondary structure (with a radius of 0.1 mm) are shown in blue. Thicker truss elements representing the primary structure are shown in red. Truss elements in the dead zone (with a radius of 1 μm) are not shown for clarity.

5.2.3 Contribution of each activities

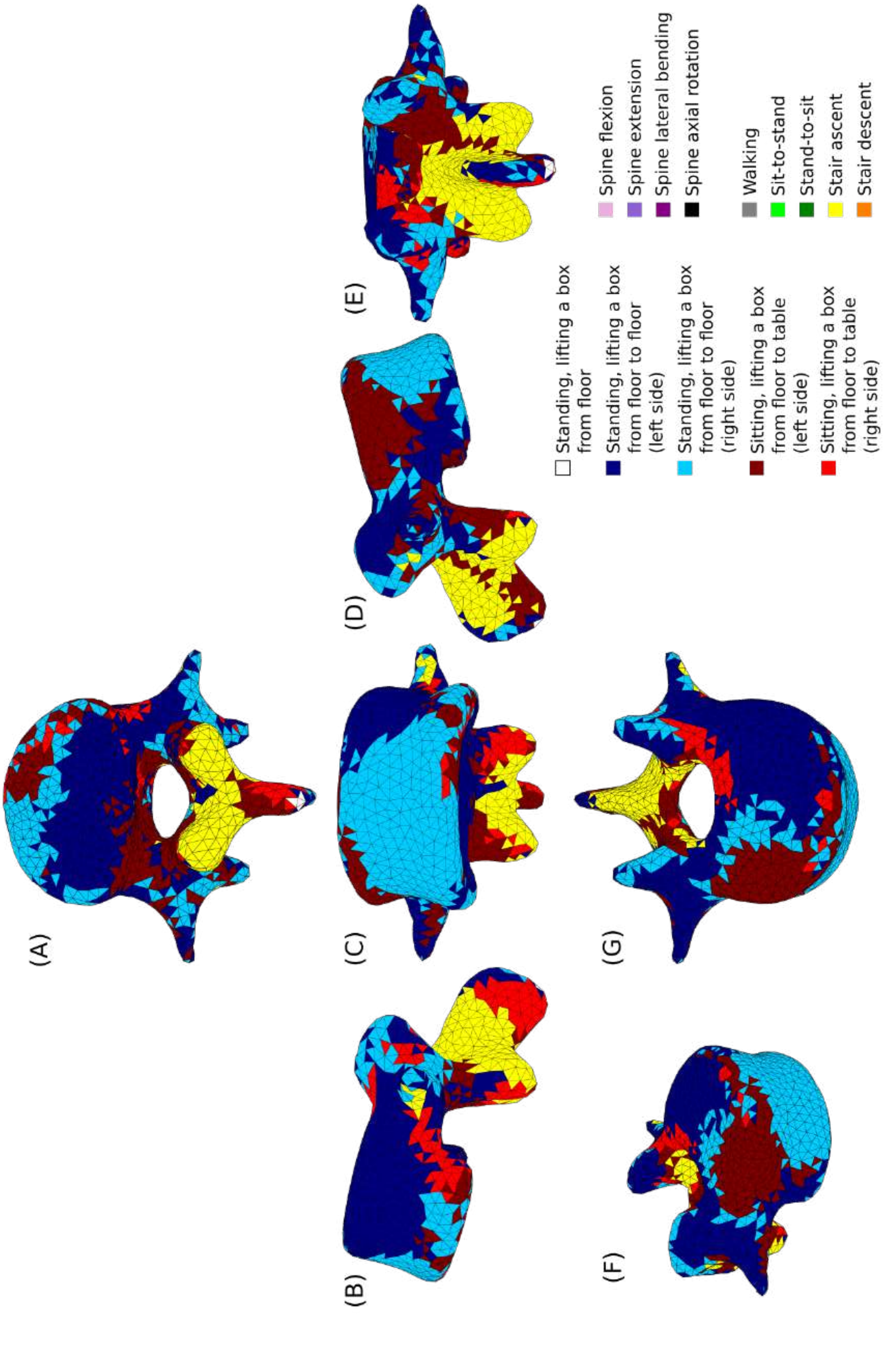

Figure S70: Contribution of each activity to the adaptation of the L4 cortical shells for the healthy scenario. (A) caudal view, (B) left lateral view, (C) frontal view, (D) right lateral view, (E) dorsal view, (F) isometric view, (G) cranial view.

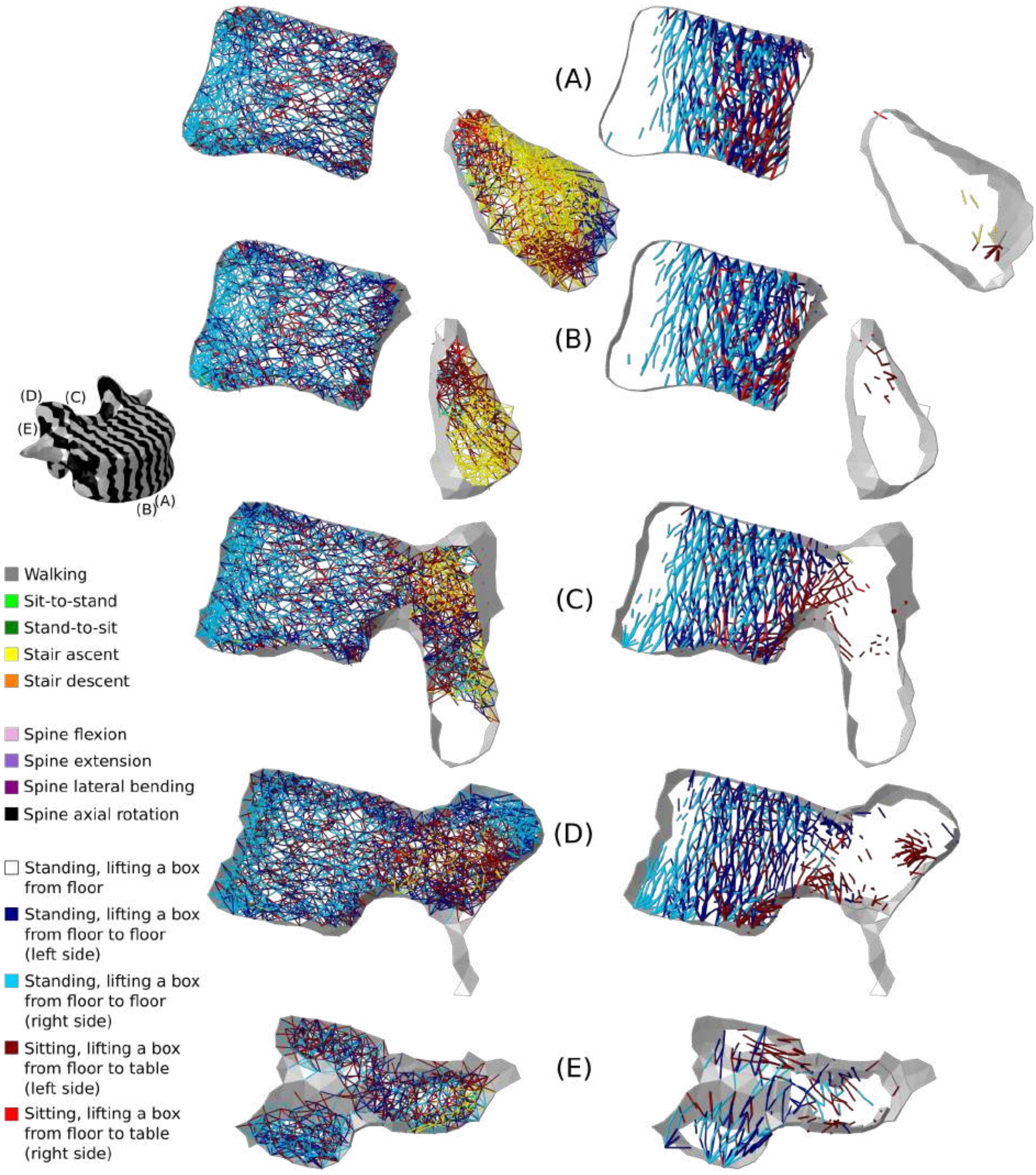

Figure S71: Selection of 3 mm sagittal slices of the right side of the converged L4 model adapted to the healthy scenario. Trabecular truss elements are colour mapped based on the activity most influential to their final geometry. On the left, only trabecular truss elements representing the secondary structure (with a radius of 0.1 mm) are shown. On the right, only trabecular truss elements representing the primary structure (with a radius superior to 0.1 mm) are shown. Cortical shell elements are sown in light grey.

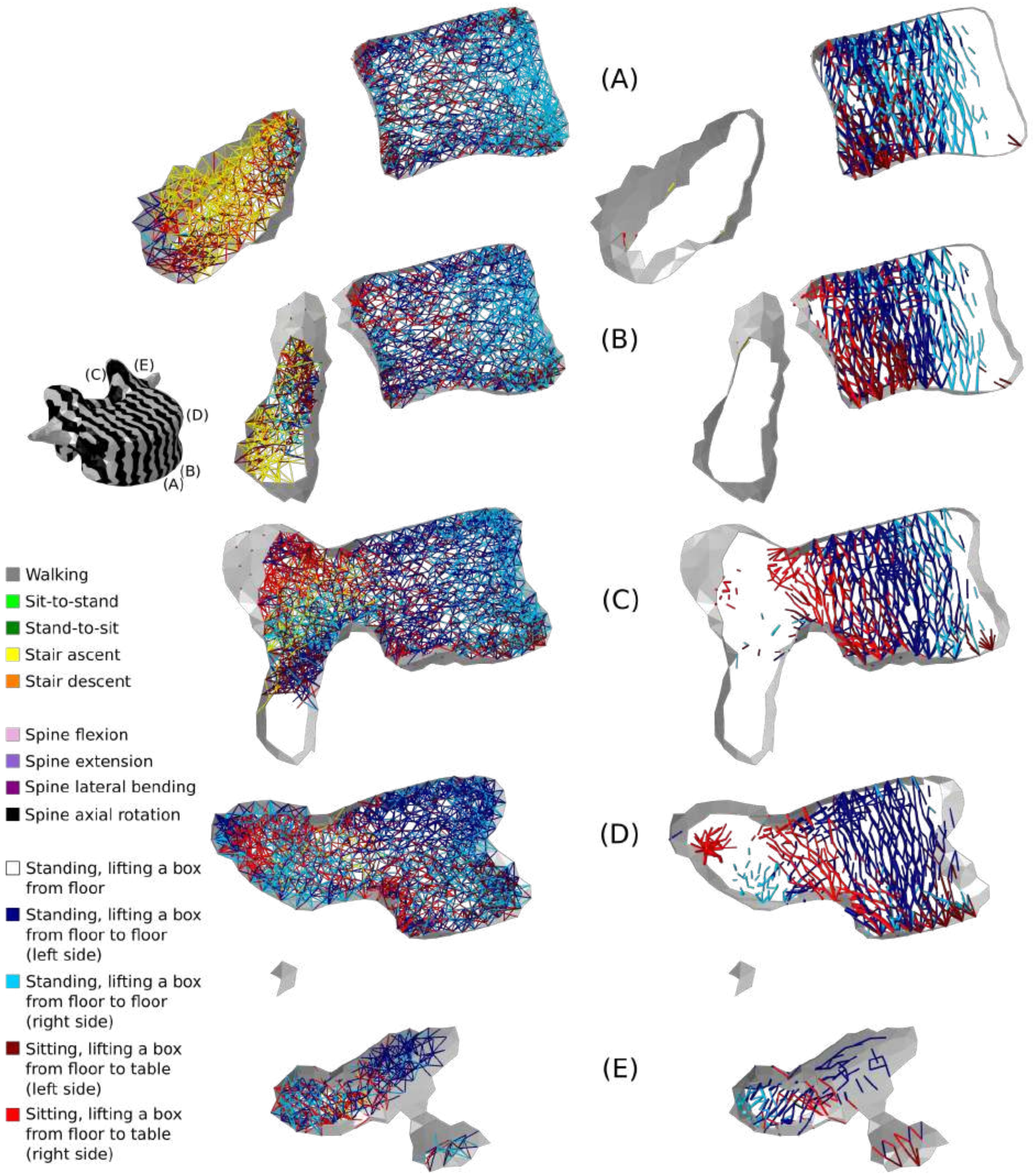

Figure S72: Selection of 3 *mm* sagittal slices of the left side of the converged L4 model adapted to the healthy scenario. Trabecular truss elements are colour mapped based on the activity most influential to their final geometry. On the left, only trabecular truss elements representing the secondary structure (with a radius of 0.1 *mm*) are shown. On the right, only trabecular truss elements representing the primary structure (with a radius superior to 0.1 *mm*) are shown. Cortical shell elements are sown in light grey.

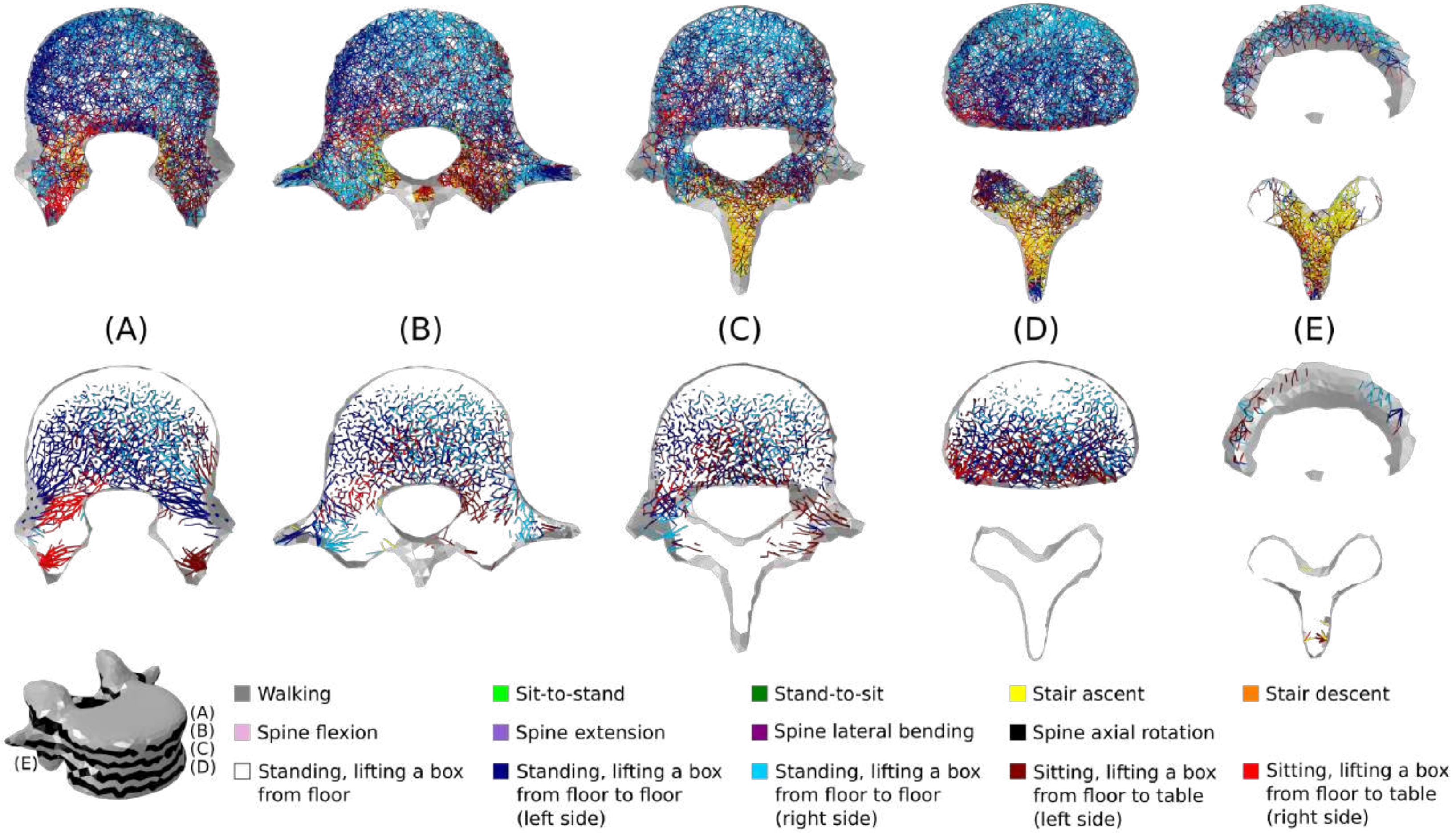

Figure S73: Selection of 3 mm transverse slices of the converged L4 model adapted to the healthy scenario. Trabecular truss elements are colour mapped based on the activity most influential to their final geometry. On the top row, only trabecular truss elements representing the secondary structure (with a radius of 0.1 mm) are shown. On the bottom row, only trabecular truss elements with a radius superior to 0.1 mm are shown. Cortical shell elements are sown in light grey.

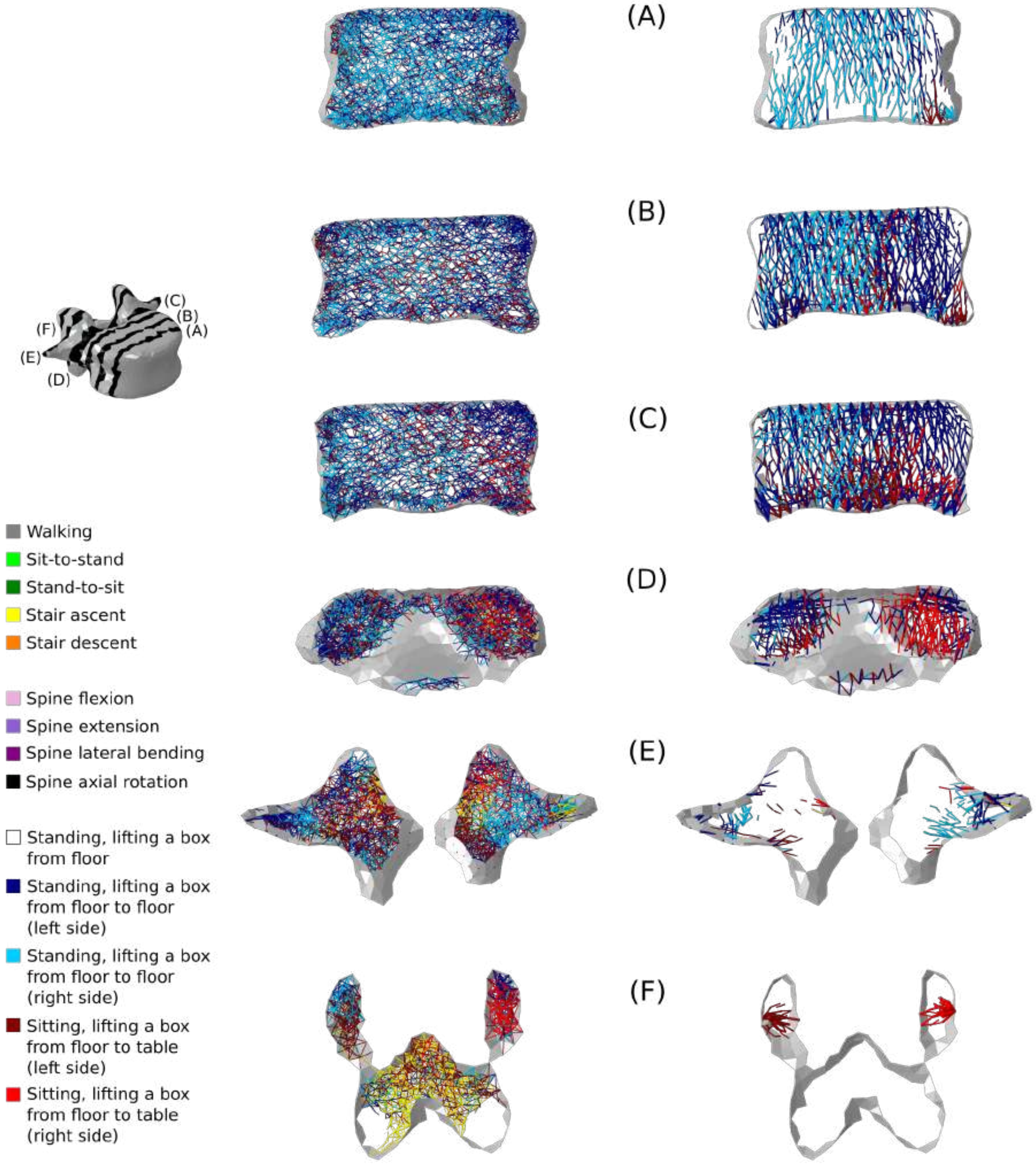

Figure S74: Selection of 3 mm coronal slices of the converged L4 model adapted to the healthy scenario. Trabecular truss elements are colour mapped based on the activity most influential to their final geometry. On the left, only trabecular truss elements representing the secondary structure (with a radius of 0.1 mm) are shown. On the right, only trabecular truss elements representing the primary structure (with a radius superior to 0.1 mm) are shown. Cortical shell elements are sown in light grey.

5.2.4 Characterisation of trabecular anisotropy

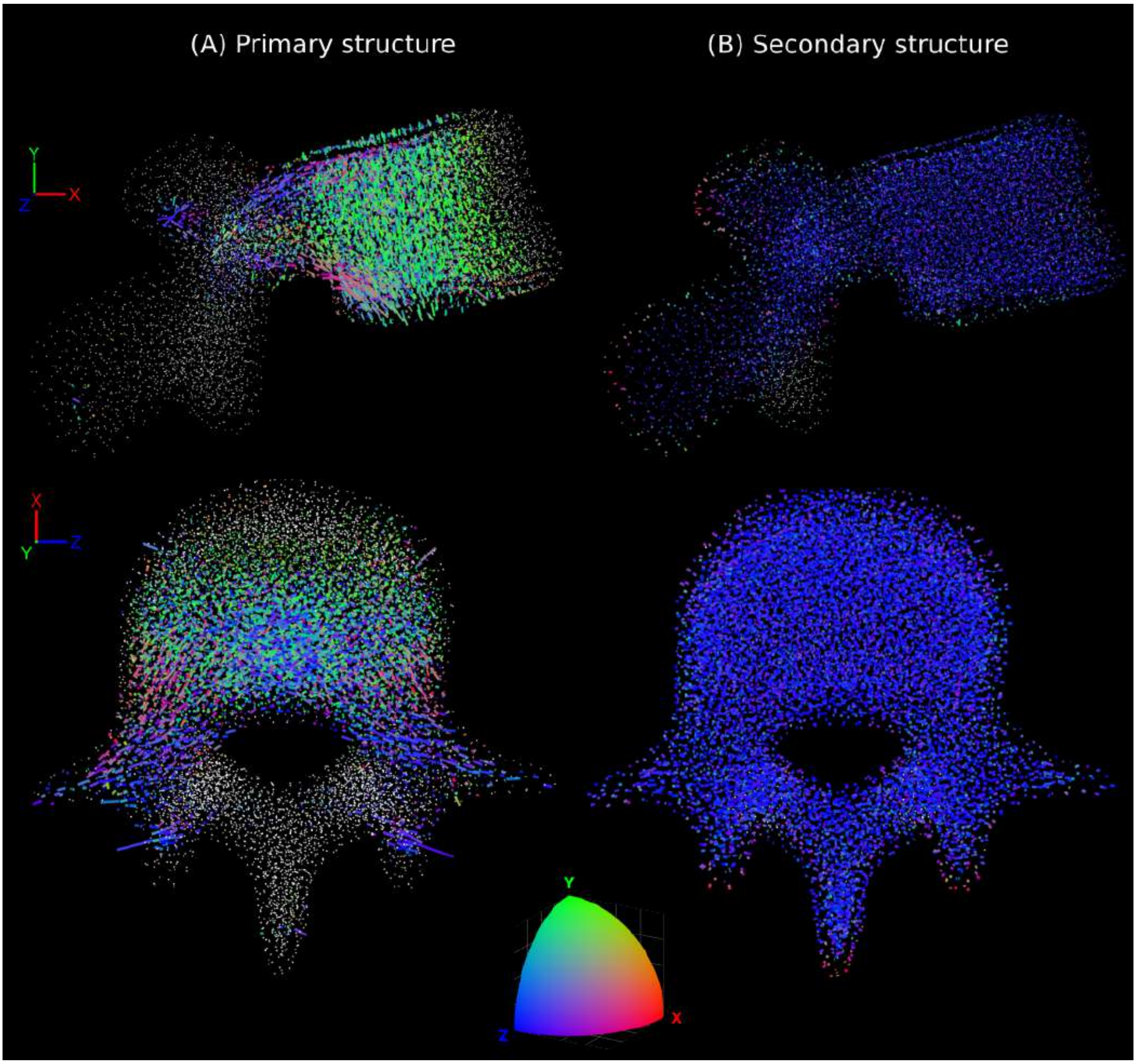

Figure S75: Characterisation of anisotropy in the trabecular bone of L4 adapted to the healthy scenario. (A) shows the orientation of the trabecular trusses of the primary structure (with a radius larger than 0.1 *mm*). (B) shows the orientation of the trabecular trusses of the secondary structure (with a radius of 0.1 *mm*). For (A) and (B), side view (top row) and top view (bottom row) are shown. Lines are attached to each trabecular node, with colour and length varying respectively with the orientations and radii of the truss elements connected to that particular node. The colour scale at the bottom shows how the colour of the lines should be interpreted. Orientation along the X, Y or Z axes are in red, green or blue respectively. Any orientation that is not colinear with these axes shows as a combination of red, green and blue. A white dot indicates a node without elements in the size range being looked at connected to it.

5.3 Sedentary scenario

5.3.1 Cortical thickness

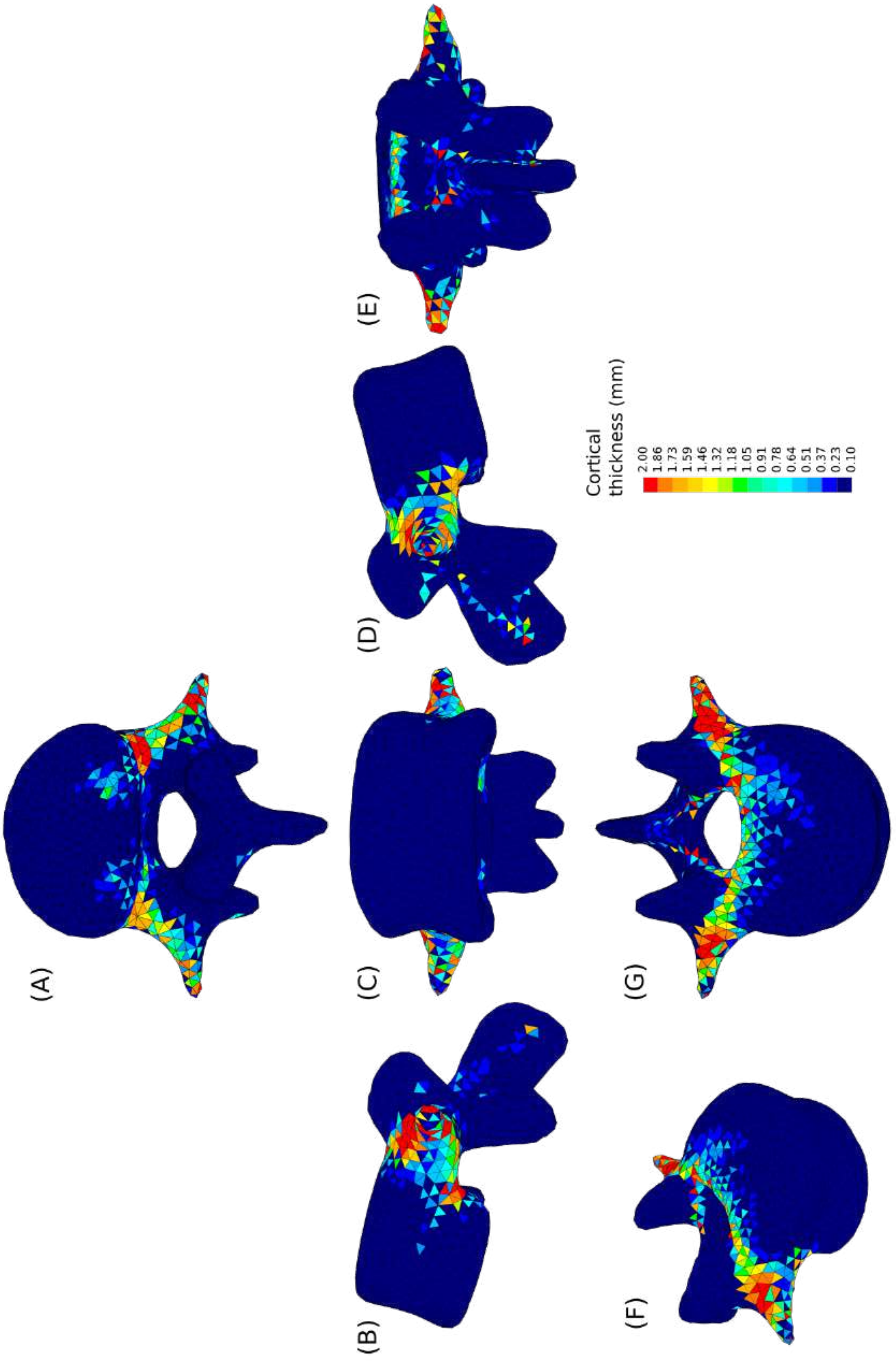

Figure S76: Cortical thickness ranging from 0.1 to 2 *mm* for the converged L4 model adapted to the sedentary scenario. (A) caudal view, (B) left lateral view, (C) frontal view, (D) right lateral view, (E) dorsal view, (F) isometric view, (G) cranial view.

5.3.2 Trabecular architecture

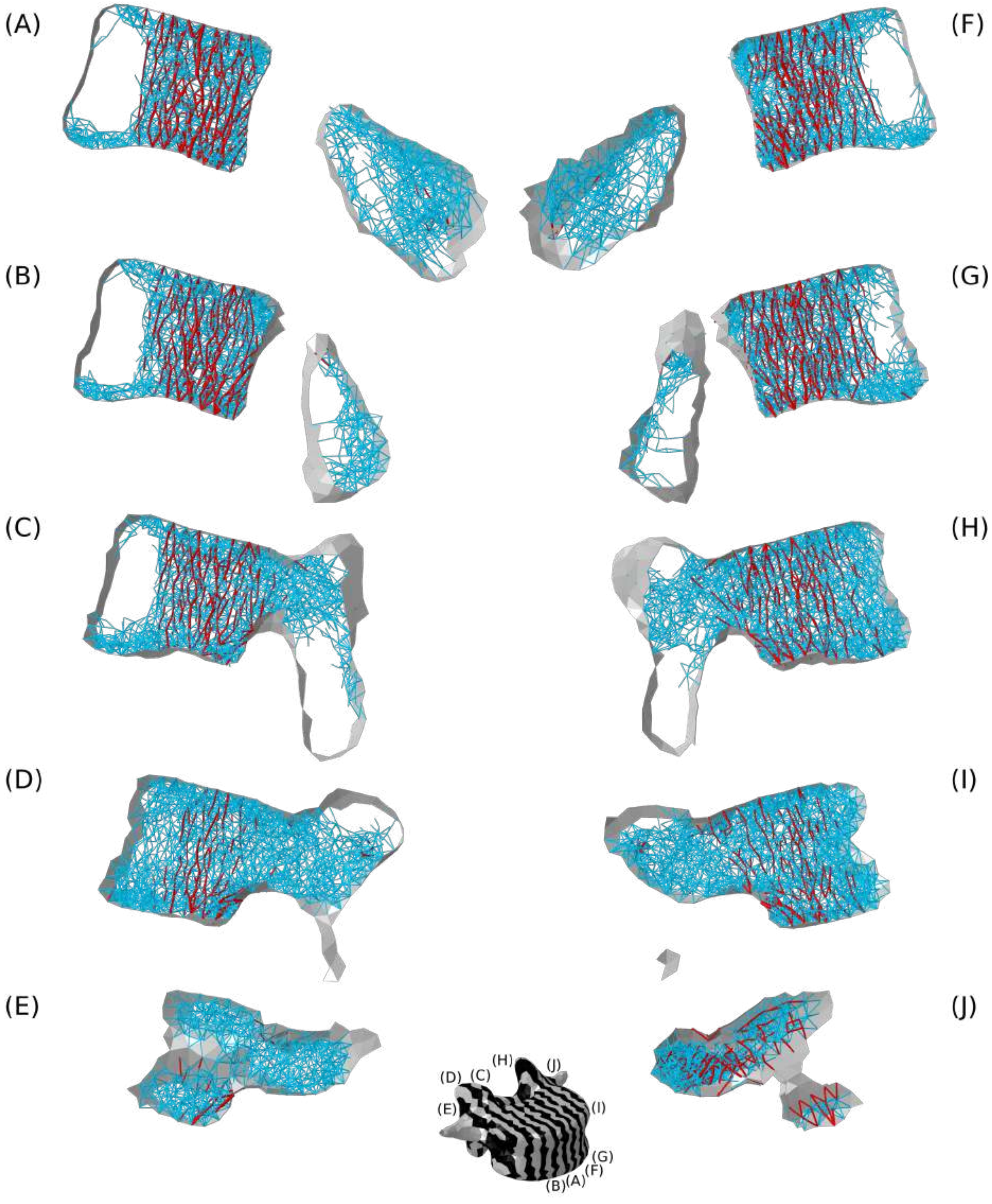

Figure S77: Selected sagittal 3 mm slices for the converged L4 model adapted to the sedentary scenario. Cortical shell elements are shown in grey. Trabecular truss elements representing the secondary structure (with a radius of 0.1 mm) are shown in blue. Thicker truss elements representing the primary structure are shown in red. Truss elements in the dead zone (with a radius of 1 μm) are not shown for clarity.

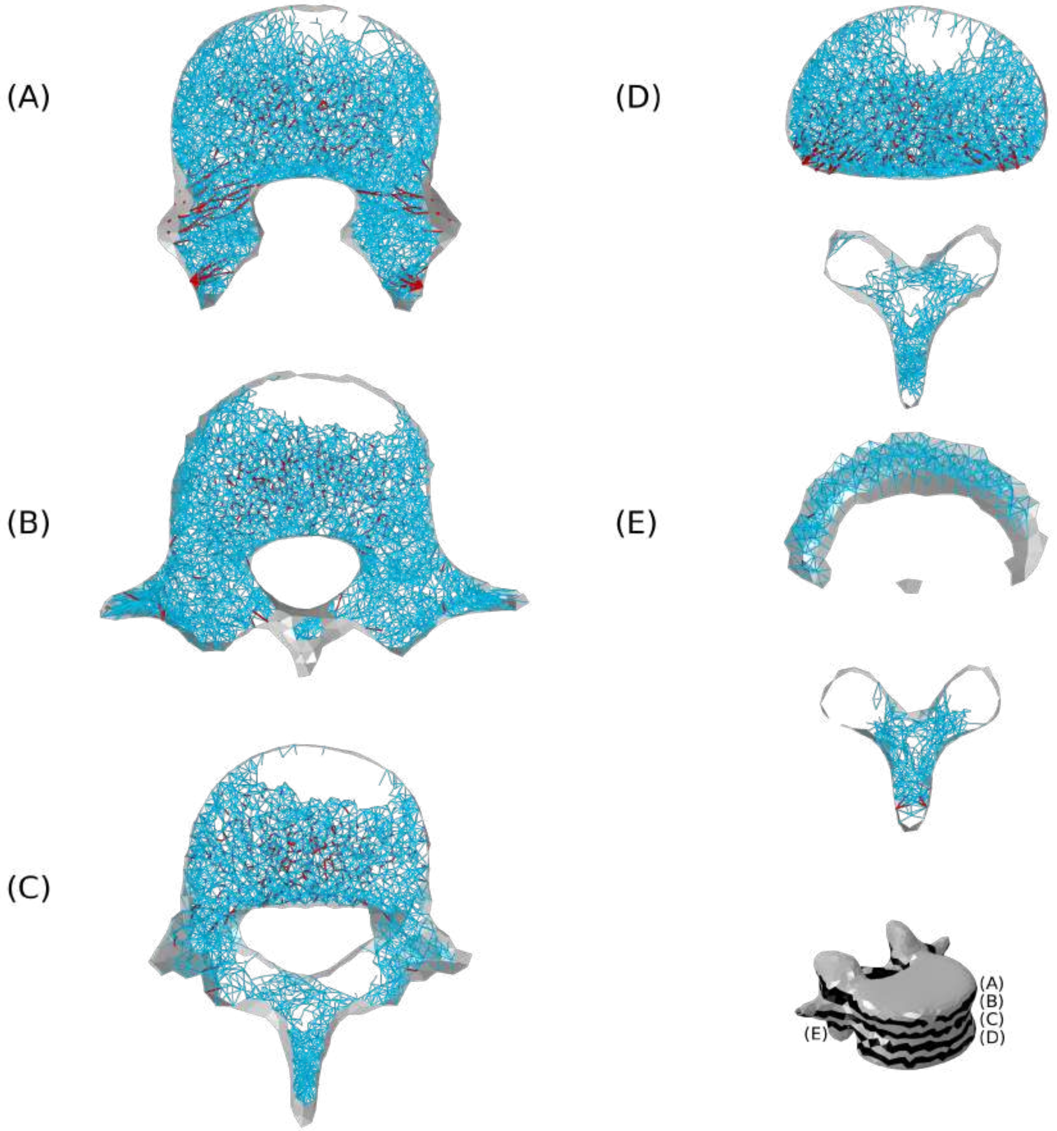

Figure S78: Selected transverse 3 *mm* slices for the converged L4 model adapted to the sedentary scenario. Cortical shell elements are shown in grey. Trabecular truss elements representing the secondary structure (with a radius of 0.1 *mm*) are shown in blue. Thicker truss elements representing the primary structure are shown in red. Truss elements in the dead zone (with a radius of 1 μm) are not shown for clarity.

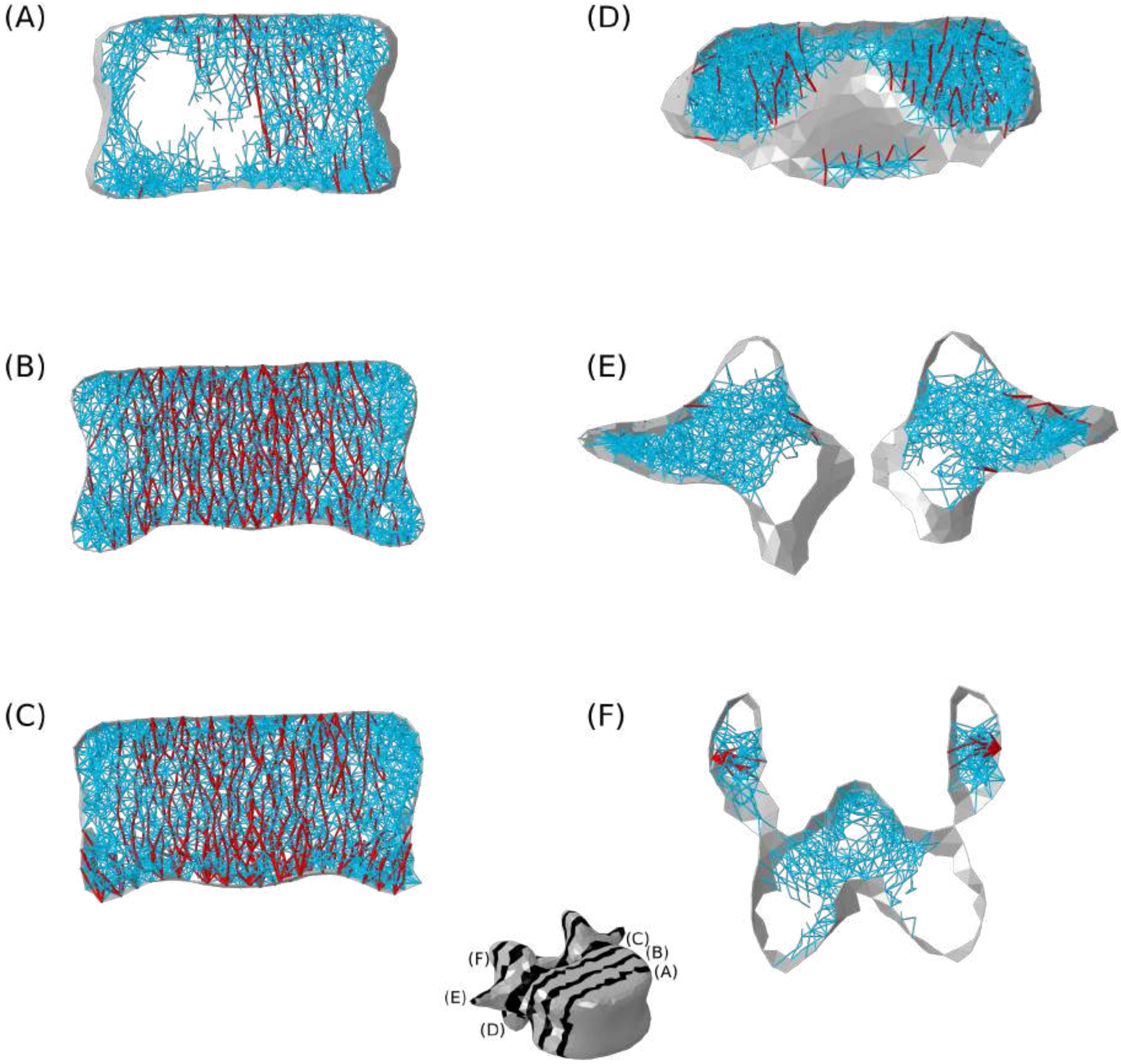

Figure S79: Selected coronal 3 mm slices for the converged L4 model adapted to the sedentary scenario. Cortical shell elements are shown in grey. Trabecular truss elements representing the secondary structure (with a radius of 0.1 mm) are shown in blue. Thicker truss elements representing the primary structure are shown in red. Truss elements in the dead zone (with a radius of 1 μm) are not shown for clarity.

5.3.3 Contribution of each activities

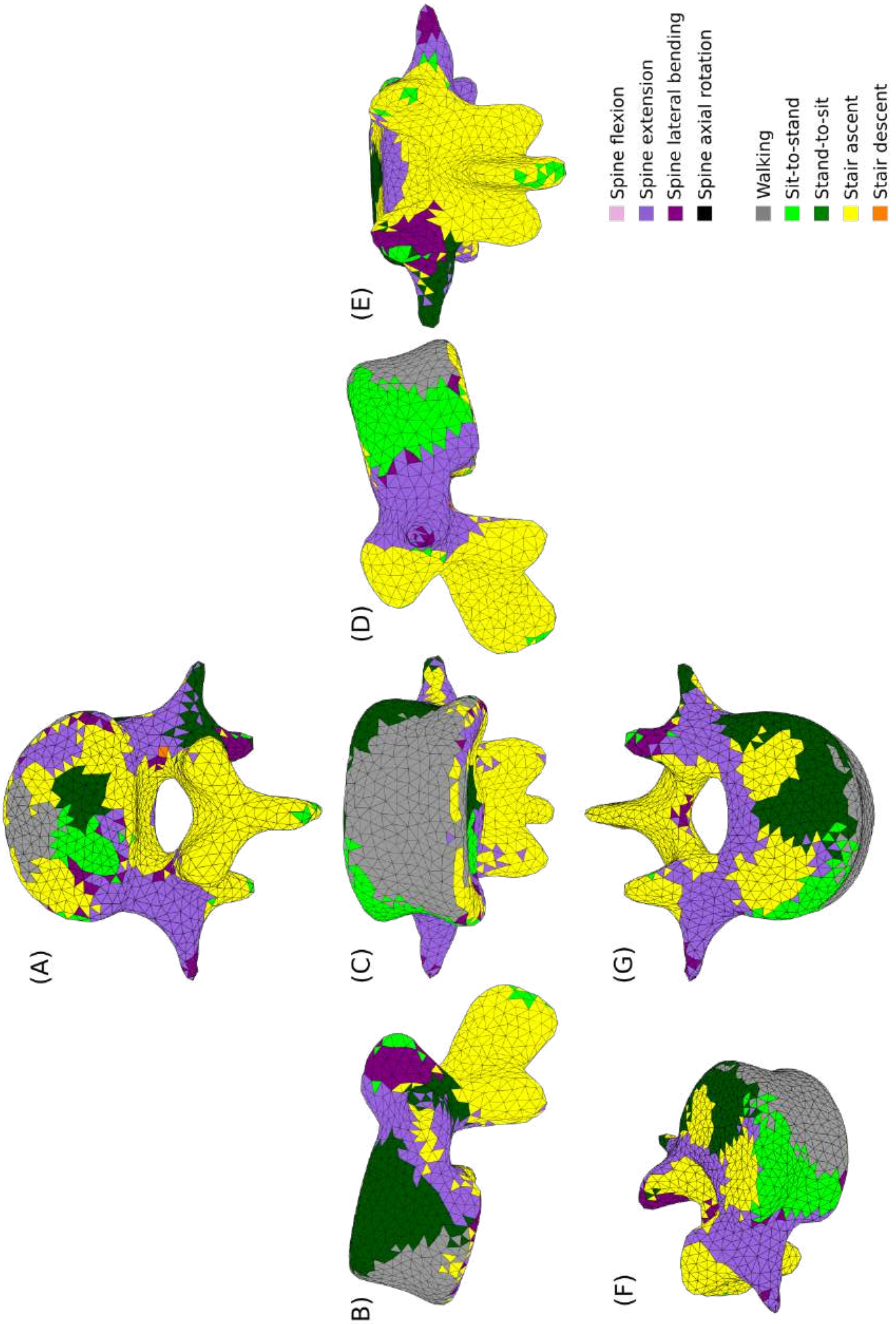

Figure S80: Contribution of each activity to the adaptation of the L4 cortical shells for the sedentary scenario. (A) caudal view, (B) left lateral view, (C) frontal view, (D) right lateral view, (E) dorsal view, (F) isometric view, (G) cranial view.

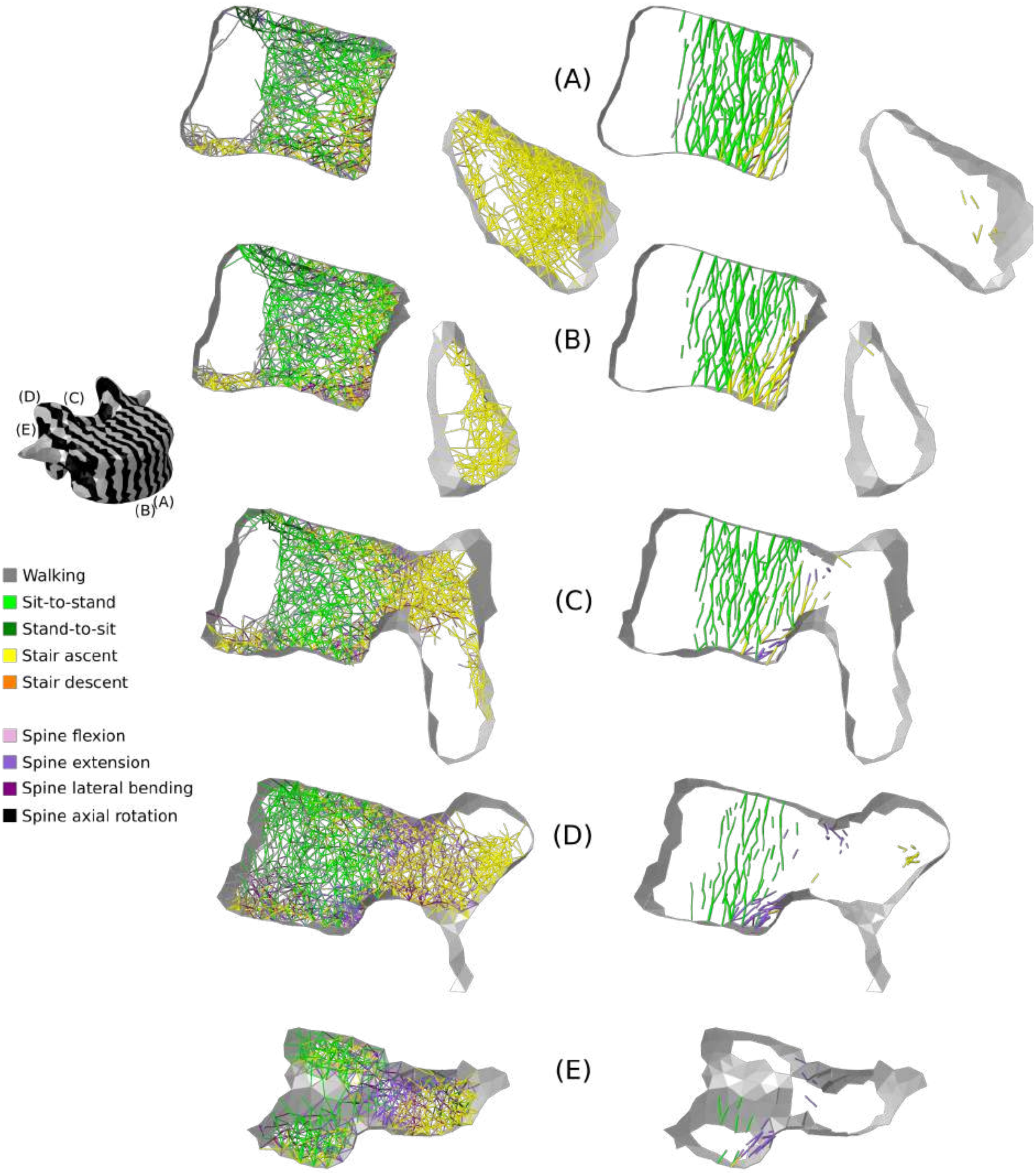

Figure S81: Selection of 3 mm sagittal slices of the right side of the converged L4 model adapted to the sedentary scenario. Trabecular truss elements are colour mapped based on the activity most influential to their final geometry. On the left, only trabecular truss elements representing the secondary structure (with a radius of 0.1 mm) are shown. On the right, only trabecular truss elements representing the primary structure (with a radius superior to 0.1 mm) are shown. Cortical shell elements are sown in light grey.

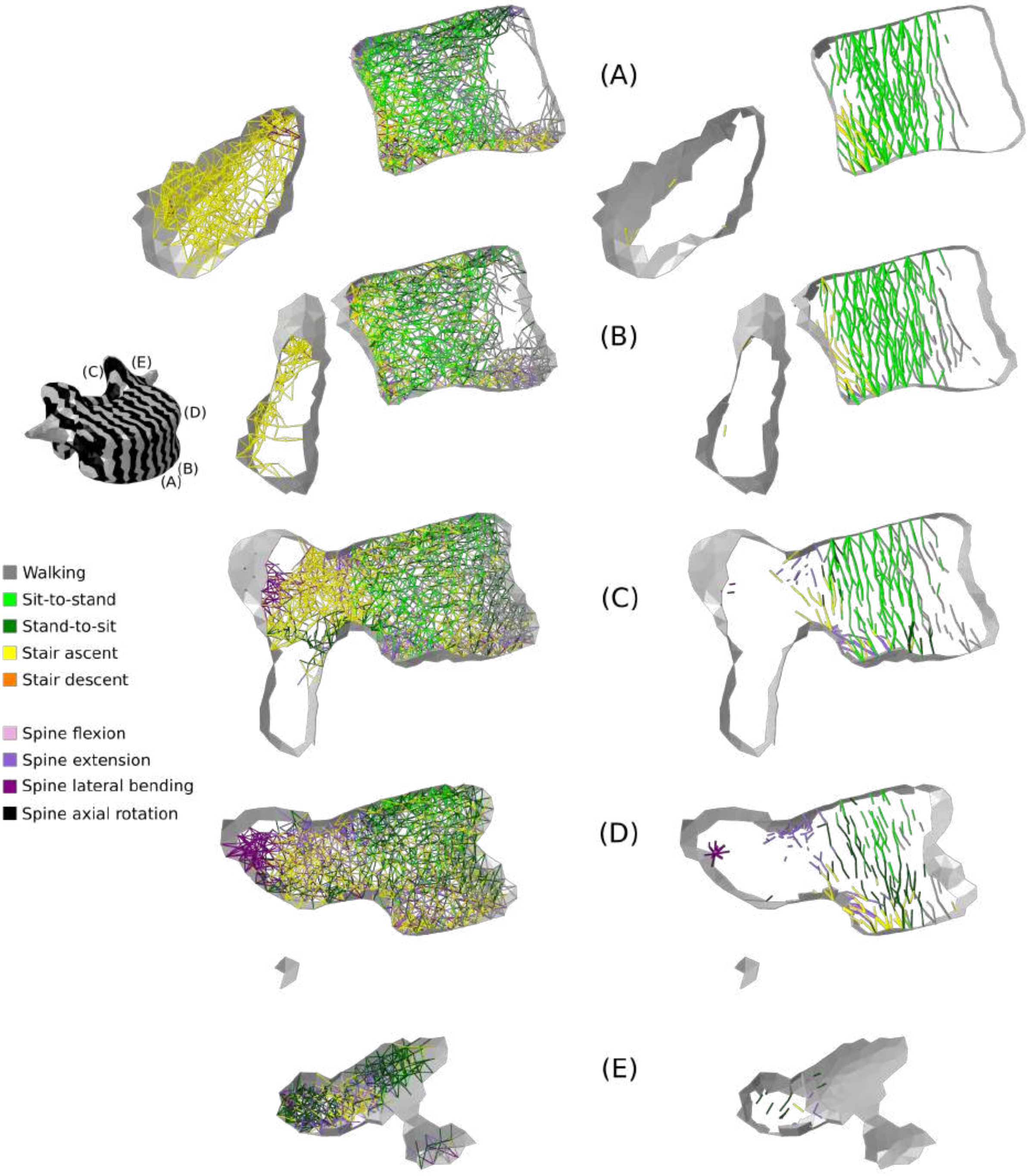

Figure S82: Selection of 3 mm sagittal slices of the left side of the converged L4 model adapted to the sedentary scenario. Trabecular truss elements are colour mapped based on the activity most influential to their final geometry. On the left, only trabecular truss elements representing the secondary structure (with a radius of 0.1 mm) are shown. On the right, only trabecular truss elements representing the primary structure (with a radius superior to 0.1 mm) are shown. Cortical shell elements are sown in light grey.

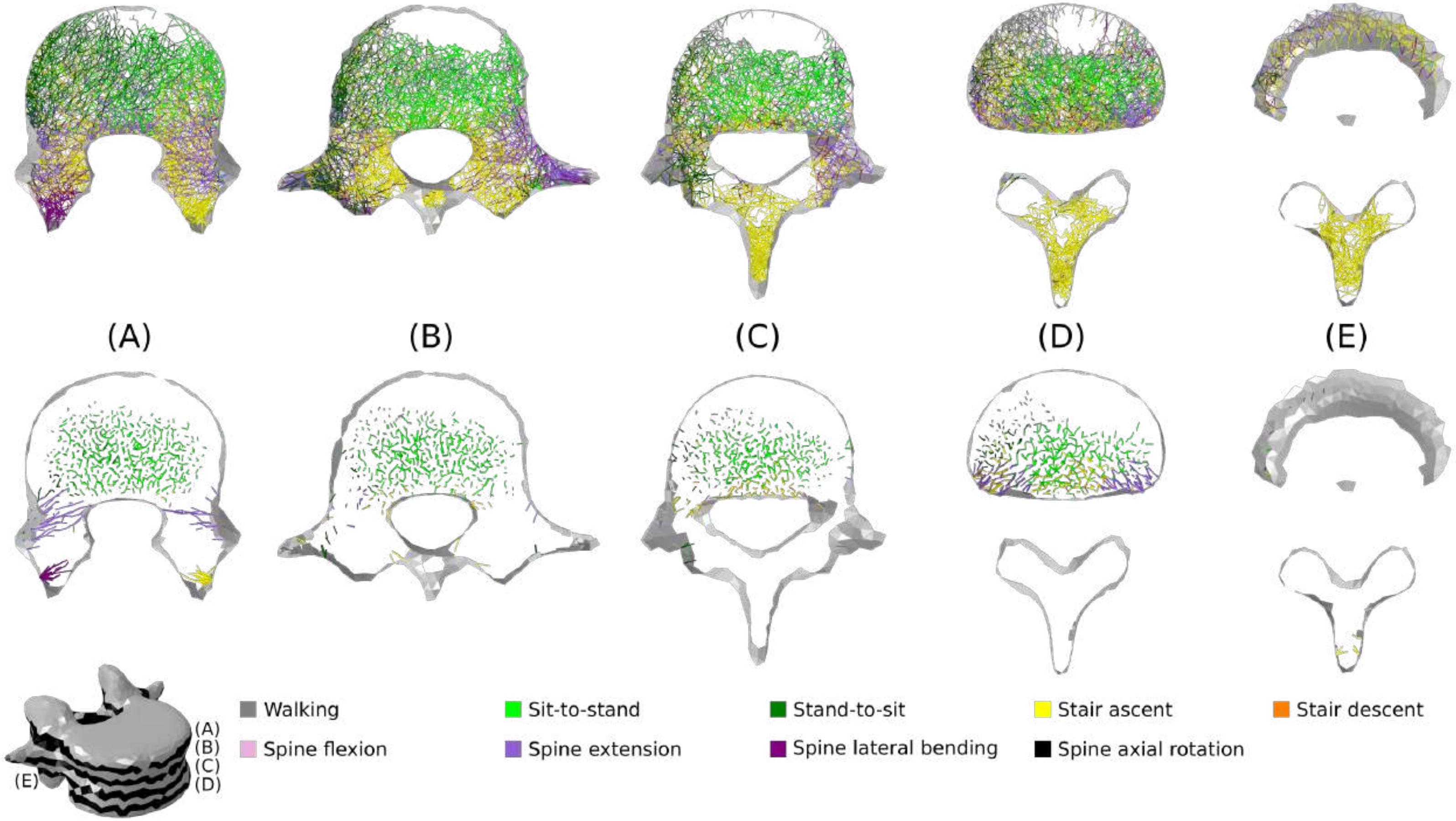

Figure S83: Selection of 3 mm transverse slices of the converged L4 model adapted to the sedentary scenario. Trabecular truss elements are colour mapped based on the activity most influential to their final geometry. On the top row, only trabecular truss elements representing the secondary structure (with a radius of 0.1 mm) are shown. On the bottom row, only trabecular truss elements with a radius superior to 0.1 mm are shown. Cortical shell elements are sown in light grey.

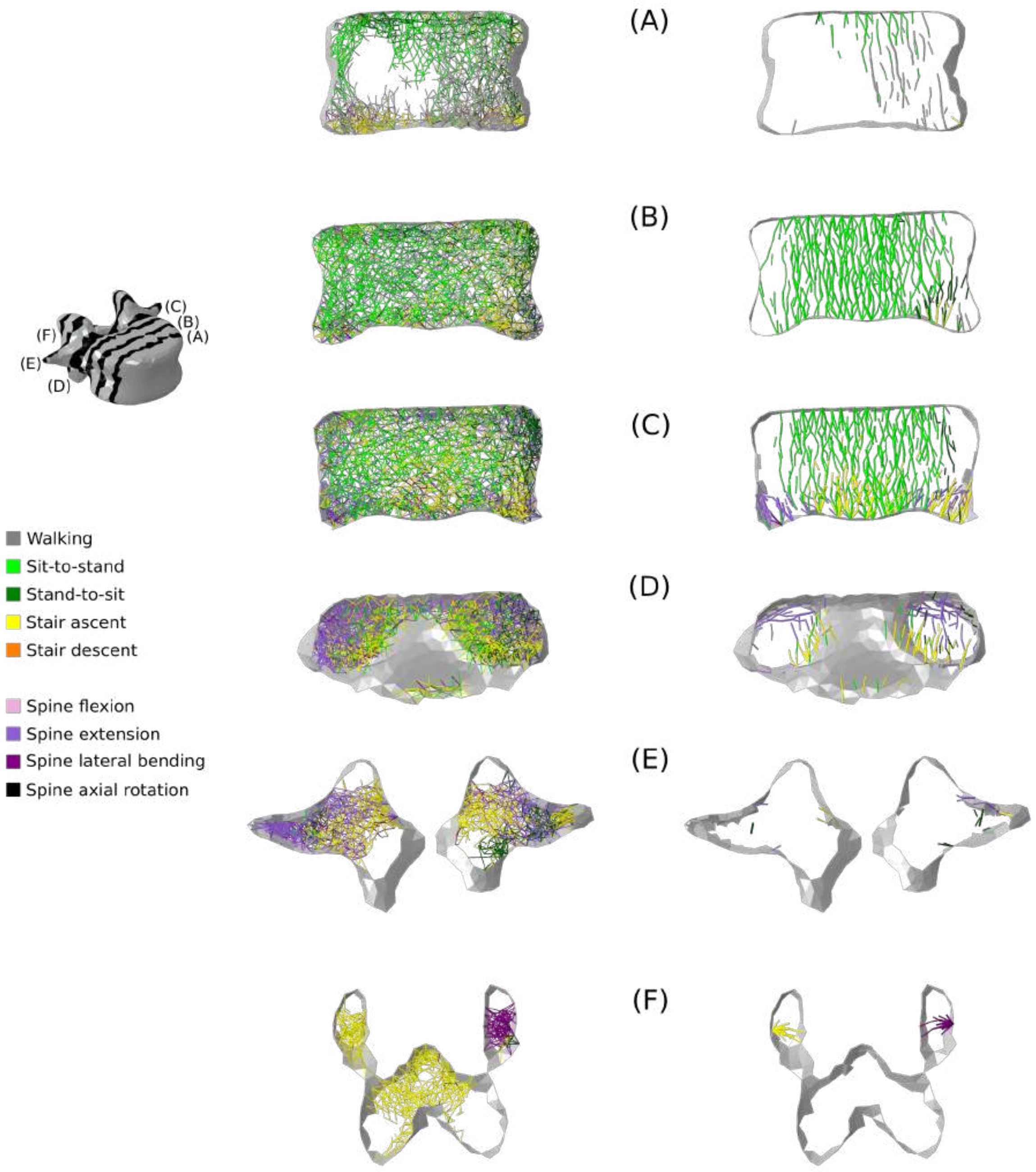

Figure S84: Selection of 3 *mm* coronal slices of the converged L4 model adapted to the sedentary scenario. Trabecular truss elements are colour mapped based on the activity most influential to their final geometry. On the left, only trabecular truss elements representing the secondary structure (with a radius of 0.1 *mm*) are shown. On the right, only trabecular truss elements representing the primary structure (with a radius superior to 0.1 *mm*) are shown. Cortical shell elements are sown in light grey.

6 FIFTH LUMBAR VERTEBRA

6.1 Load cases

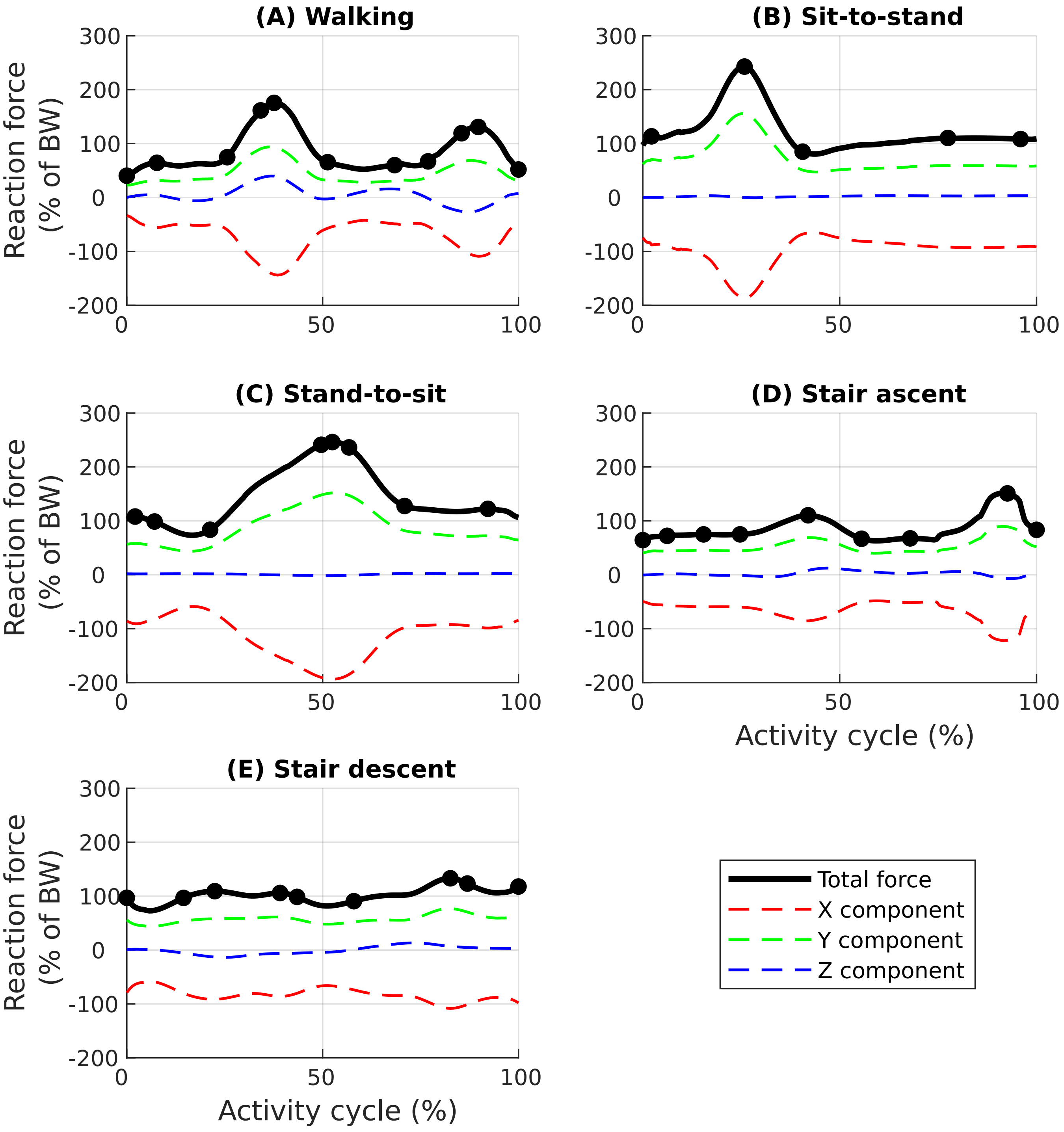

Figure S85: Selection of load cases for L5, for five activities related to locomotion ((A) level walking, (B) standing up from a chair, (C) sitting down on a chair, (D) walking up the stairs, and (E) walking down the stairs). Total reaction force at L5-S1 joint derived from the musculoskeletal model and normalised to body weight (BW) are shown as black lines. Dots indicate the frames selected for the finite element analysis. Red, green and blue dashed lines show X, Y and Z components of the reaction force expressed in the vertebra coordinate system.

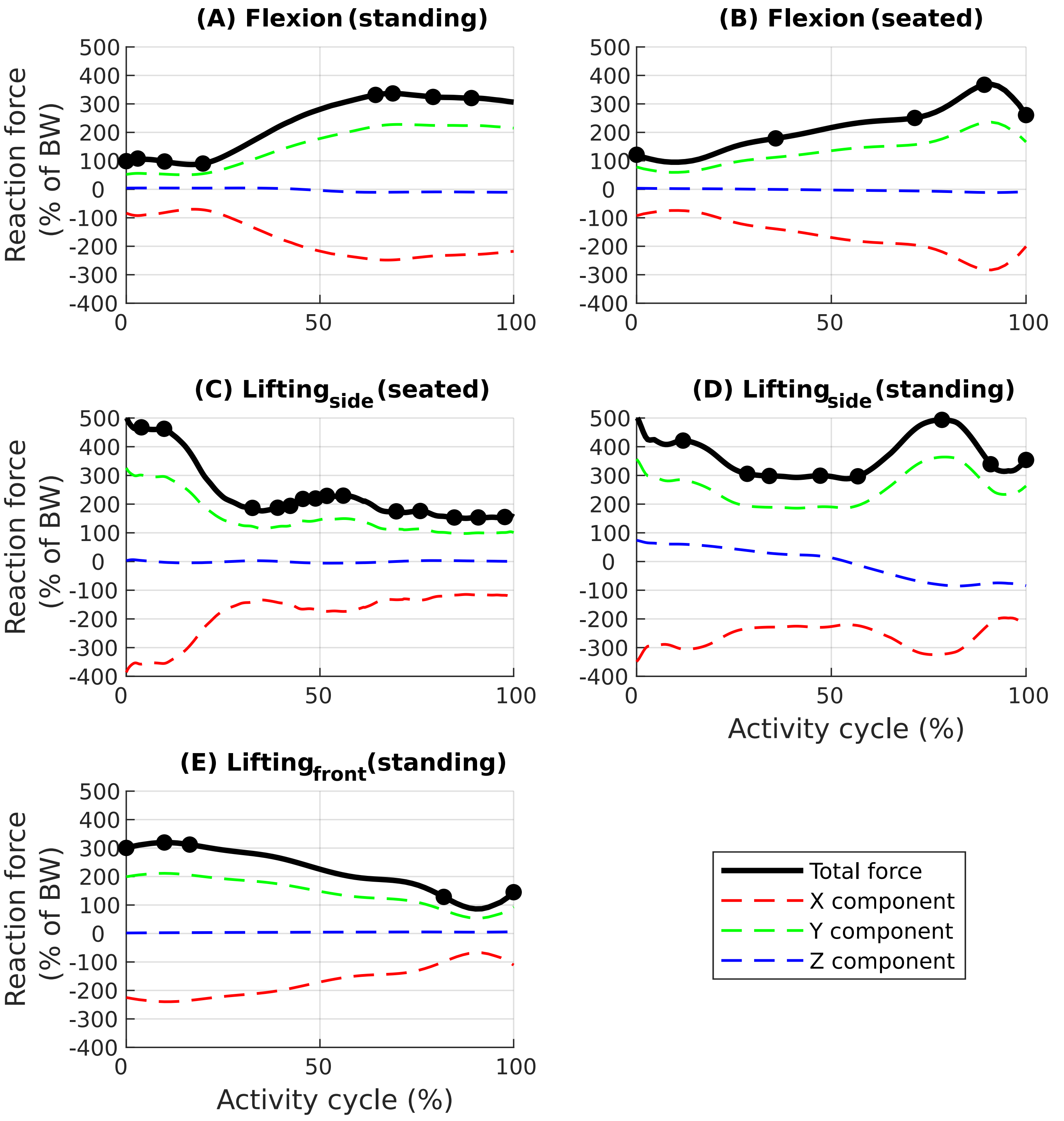

Figure S86: Selection of load cases for L5, for seven activities involving spine movements ((A) forward flexion from upright standing to maximum flexion, (B) forward flexion from upright sitting, (C) in a seated position, twisting and lifting a box from the floor, from the right side to a table in front, (D) in a standing position, twisting and lifting a box from the floor, from the left side to the right side, and (E) in a standing position, lifting a box from the floor in front to the chest). Total reaction force at L5-S1 joint derived from the musculoskeletal model and normalised to body weight (BW) are shown as black lines. Dots indicate the frames selected for the finite element analysis. Load cases obtained with activities (C) and (D) were mirrored through the sagittal plane to obtain loading on the other side. Red, green and blue dashed lines show X, Y and Z components of the reaction force expressed in the vertebra coordinate system.

6.2 Healthy scenario

6.2.1 Cortical thickness

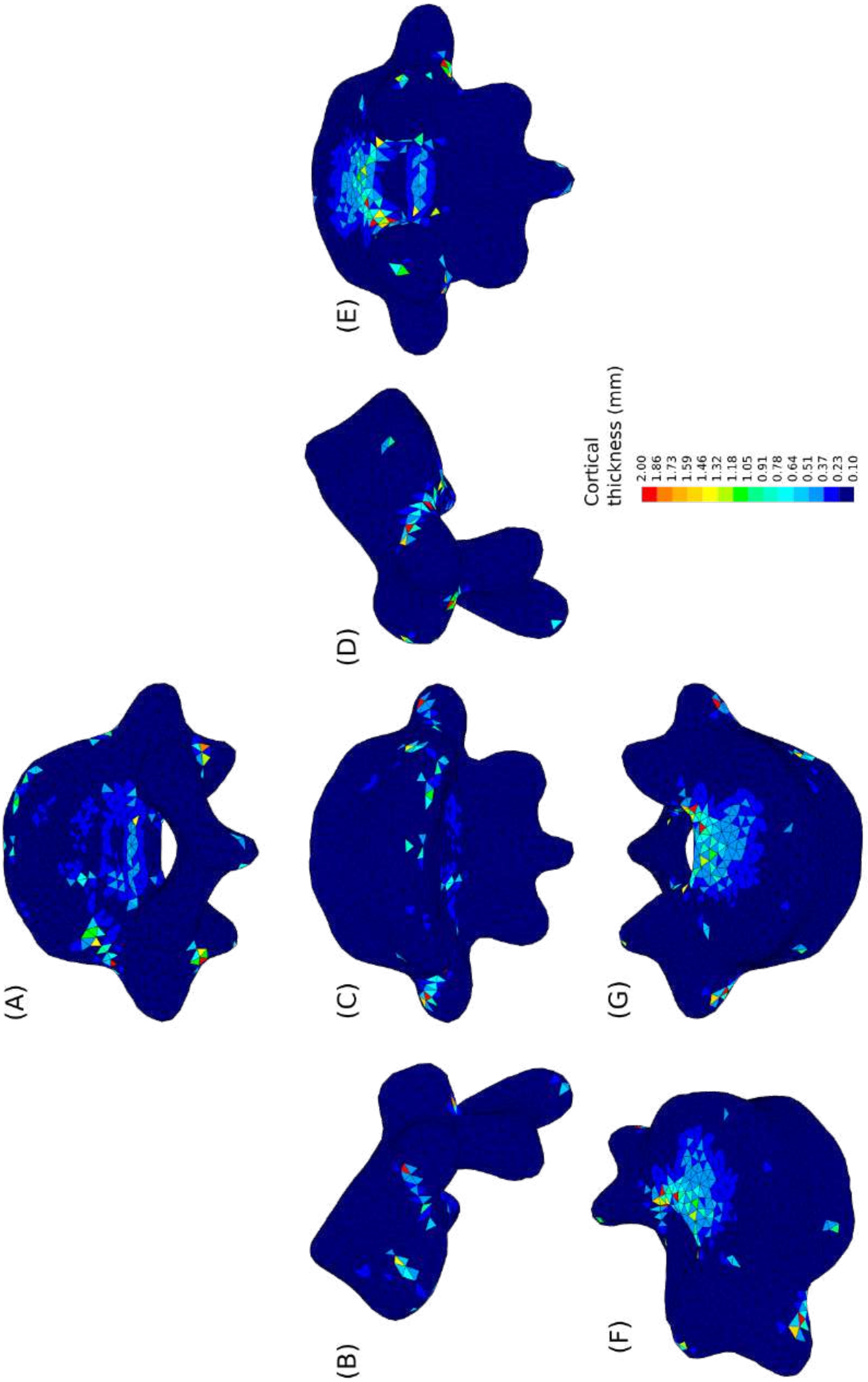

Figure S87: Cortical thickness ranging from 0.1 to 2 *mm* for the converged L5 model adapted to the healthy scenario. (A) caudal view, (B) left lateral view, (C) frontal view, (D) right lateral view, (E) dorsal view, (F) isometric view, (G) cranial view.

6.2.2 Trabecular architecture

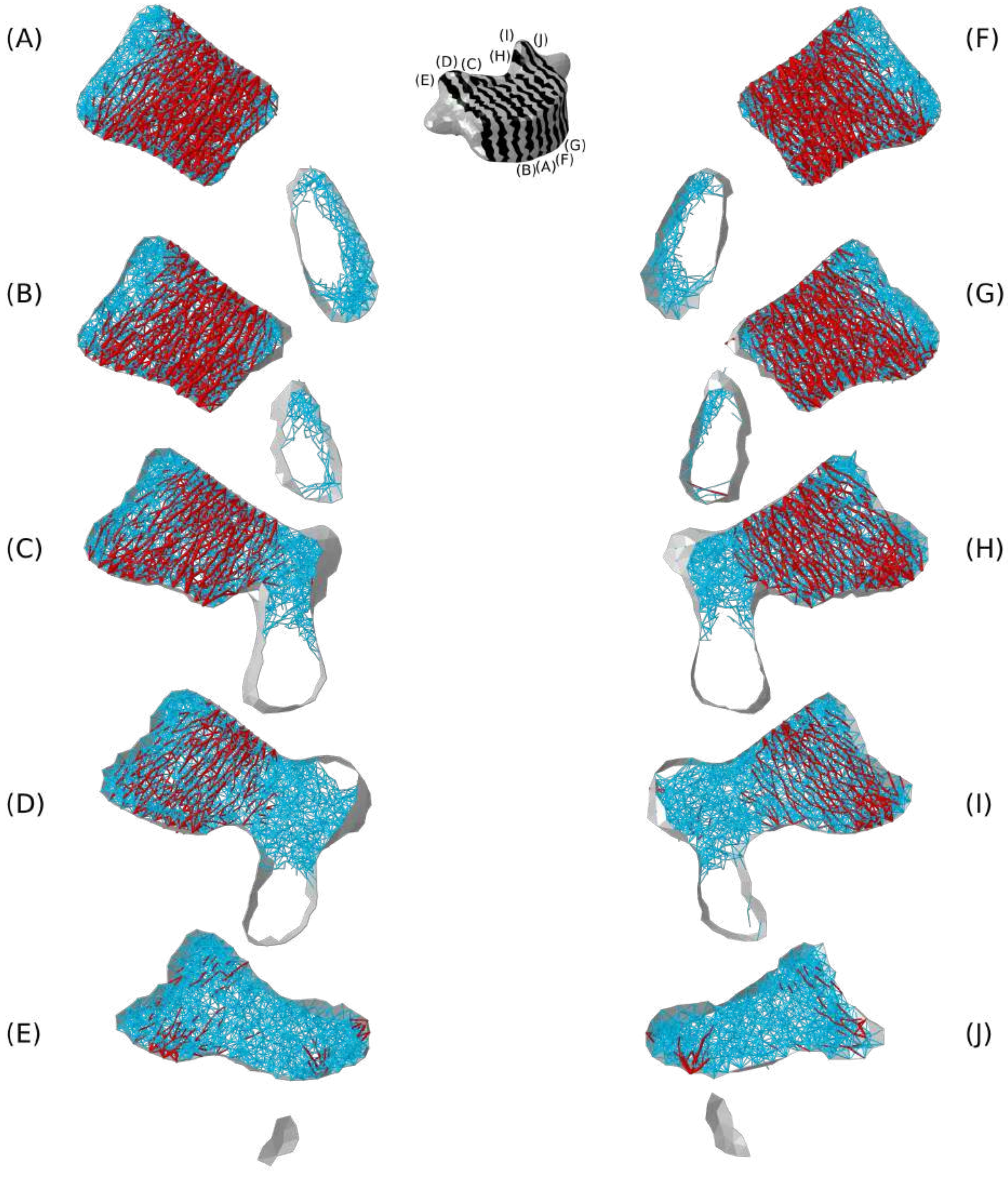

Figure S88: Selected sagittal 3 mm slices for the converged L5 model adapted to the healthy scenario. Cortical shell elements are shown in grey. Trabecular truss elements representing the secondary structure (with a radius of 0.1 mm) are shown in blue. Thicker truss elements representing the primary structure are shown in red. Truss elements in the dead zone (with a radius of 1 μm) are not shown for clarity.

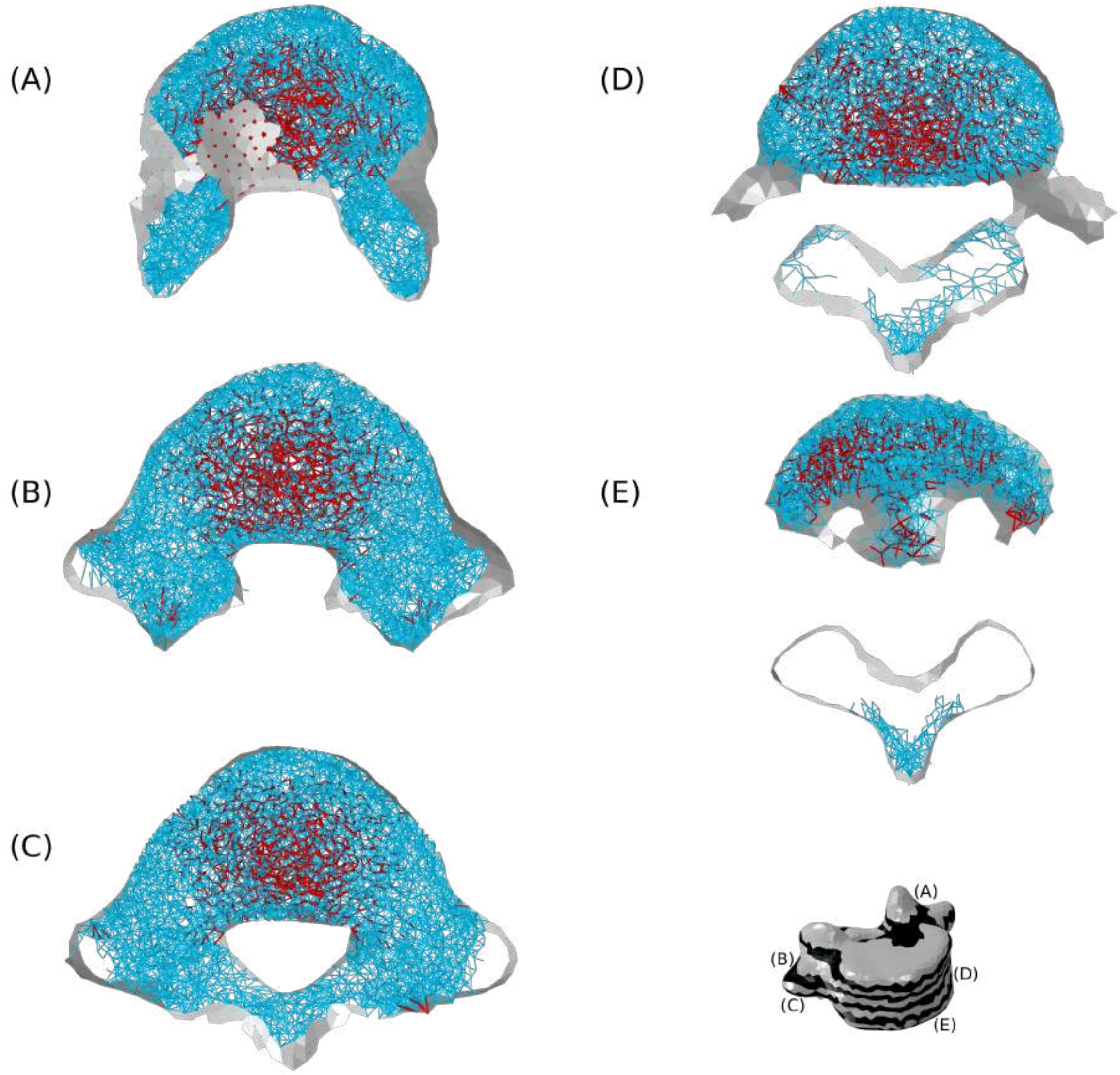

Figure S89: Selected transverse 3 mm slices for the converged L5 model adapted to the healthy scenario. Cortical shell elements are shown in grey. Trabecular truss elements representing the secondary structure (with a radius of 0.1 mm) are shown in blue. Thicker truss elements representing the primary structure are shown in red. Truss elements in the dead zone (with a radius of 1 μm) are not shown for clarity.

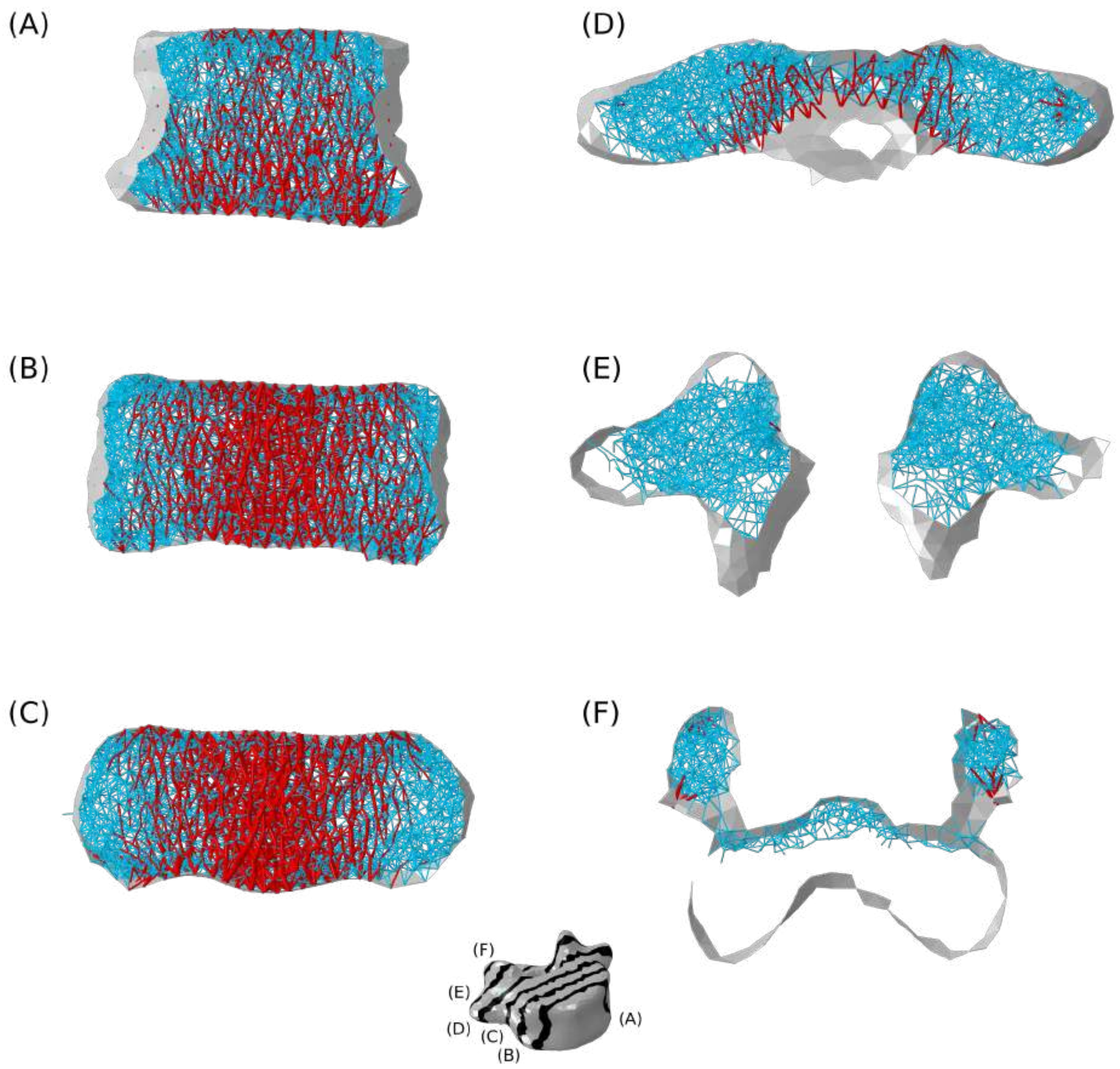

Figure S90: Selected coronal 3 mm slices for the converged L5 model adapted to the healthy scenario. Cortical shell elements are shown in grey. Trabecular truss elements representing the secondary structure (with a radius of 0.1 mm) are shown in blue. Thicker truss elements representing the primary structure are shown in red. Truss elements in the dead zone (with a radius of 1 μm) are not shown for clarity.

6.2.3 Contribution of each activities

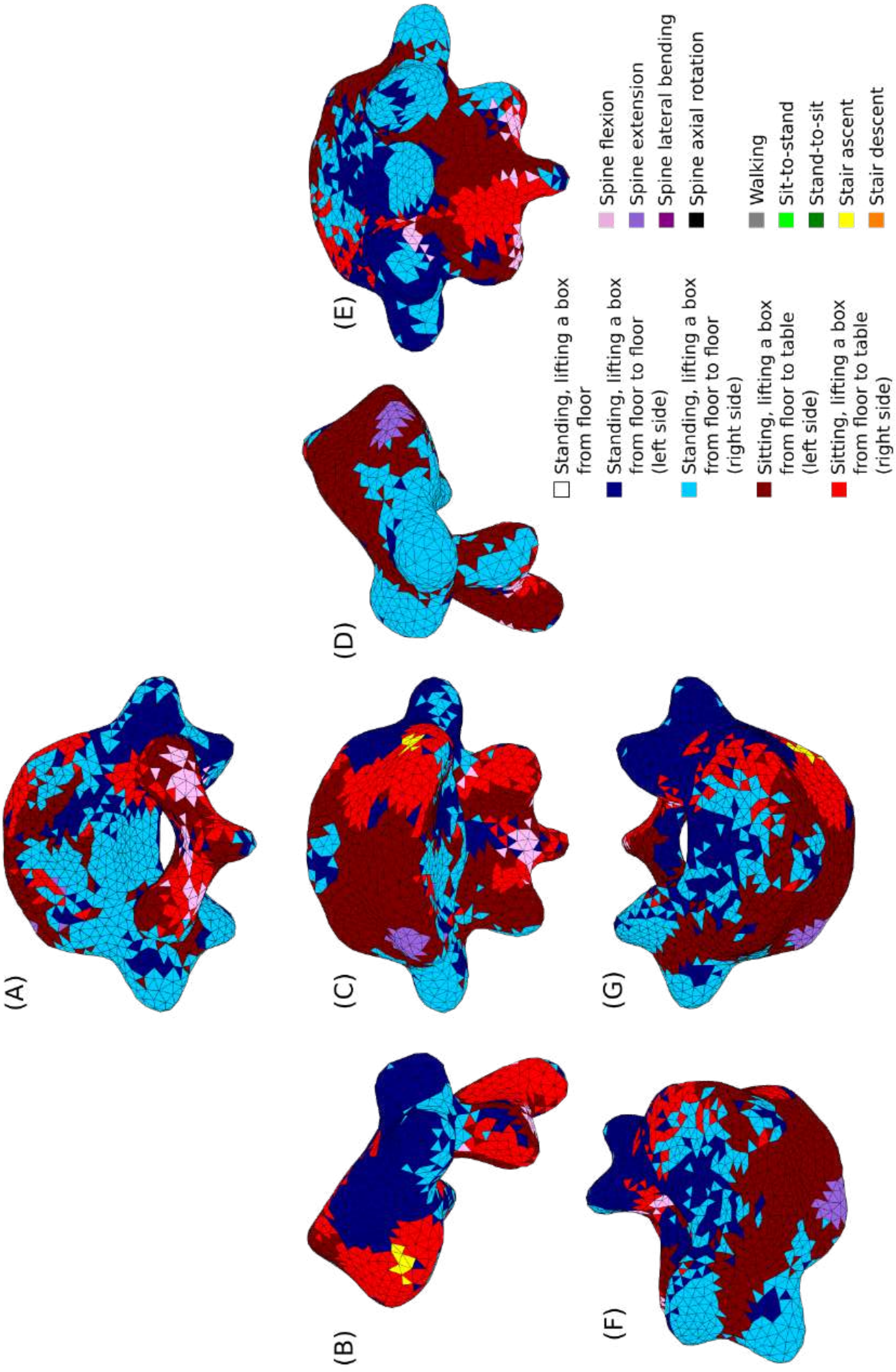

Figure S91: Contribution of each activity to the adaptation of the L5 cortical shells for the healthy scenario. (A) caudal view, (B) left lateral view, (C) frontal view, (D) right lateral view, (E) dorsal view, (F) isometric view, (G) cranial view.

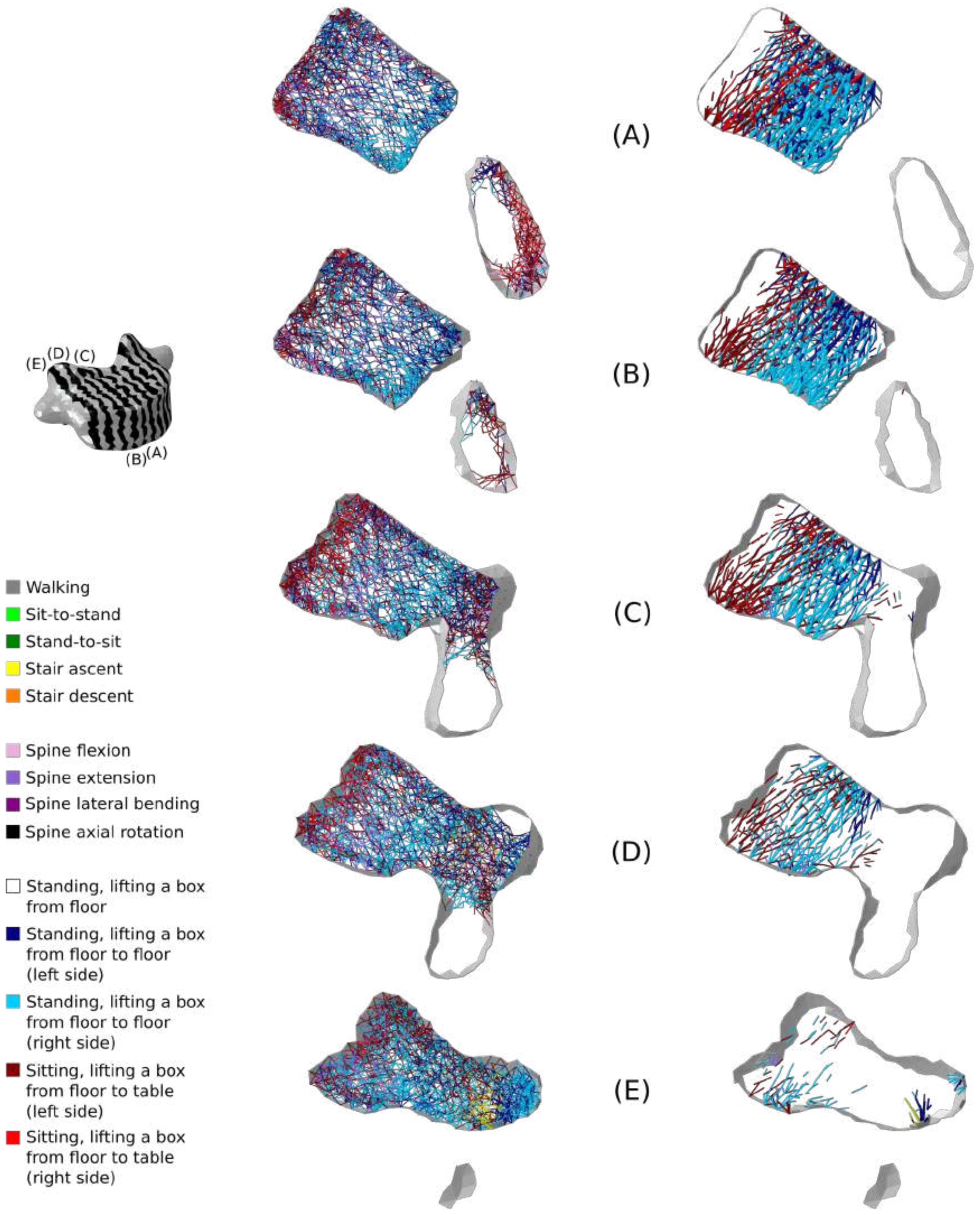

Figure S92: Selection of 3 mm sagittal slices of the right side of the converged L5 model adapted to the healthy scenario. Trabecular truss elements are colour mapped based on the activity most influential to their final geometry. On the left, only trabecular truss elements representing the secondary structure (with a radius of 0.1 mm) are shown. On the right, only trabecular truss elements representing the primary structure (with a radius superior to 0.1 mm) are shown. Cortical shell elements are sown in light grey.

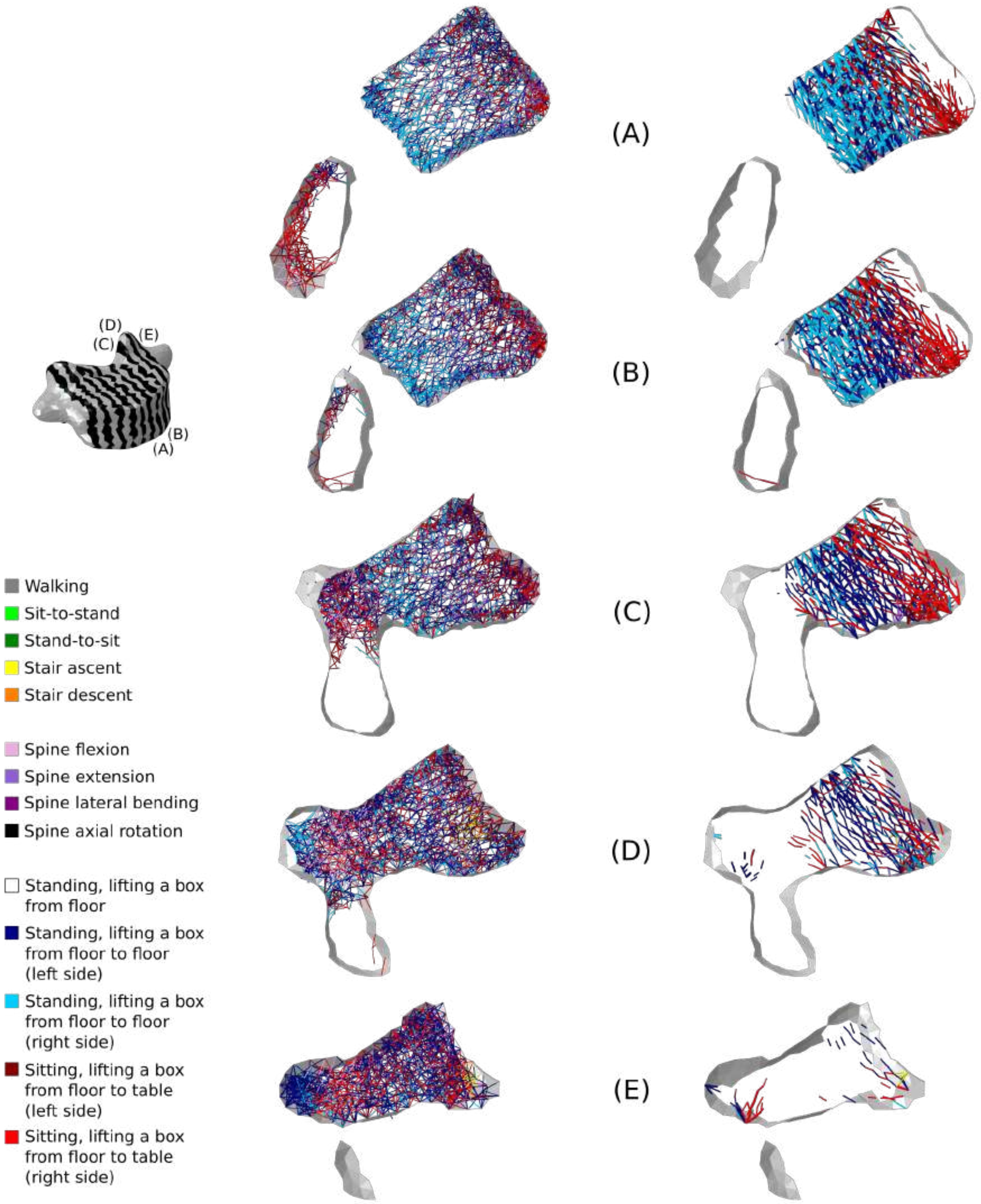

Figure S93: Selection of 3 mm sagittal slices of the left side of the converged L5 model adapted to the healthy scenario. Trabecular truss elements are colour mapped based on the activity most influential to their final geometry. On the left, only trabecular truss elements representing the secondary structure (with a radius of 0.1 mm) are shown. On the right, only trabecular truss elements representing the primary structure (with a radius superior to 0.1 mm) are shown. Cortical shell elements are sown in light grey.

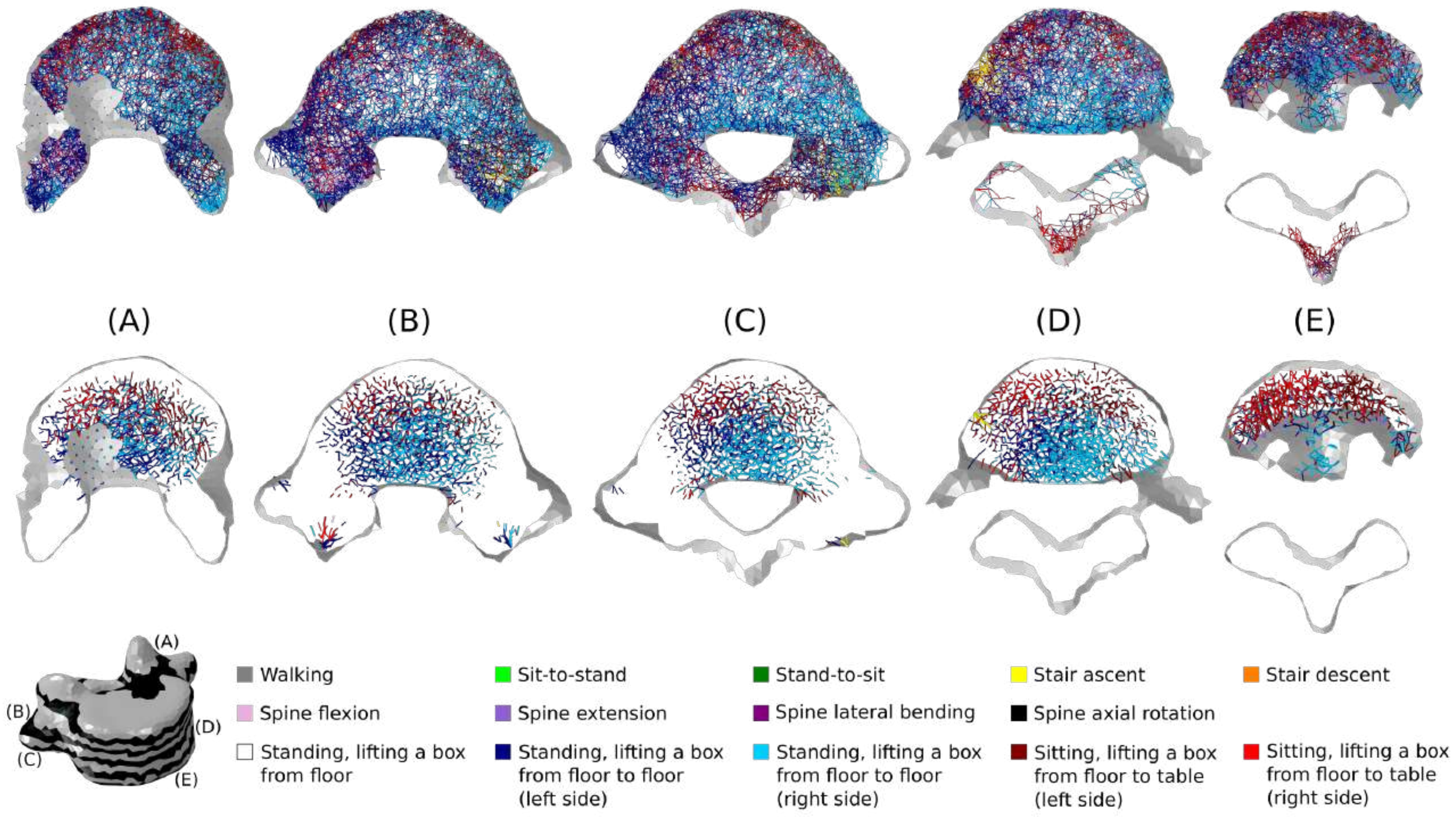

Figure S94: Selection of 3 mm transverse slices of the converged L5 model adapted to the healthy scenario. Trabecular truss elements are colour mapped based on the activity most influential to their final geometry. On the top row, only trabecular truss elements representing the secondary structure (with a radius of 0.1 mm) are shown. On the bottom row, only trabecular truss elements with a radius superior to 0.1 mm are shown. Cortical shell elements are sown in light grey.

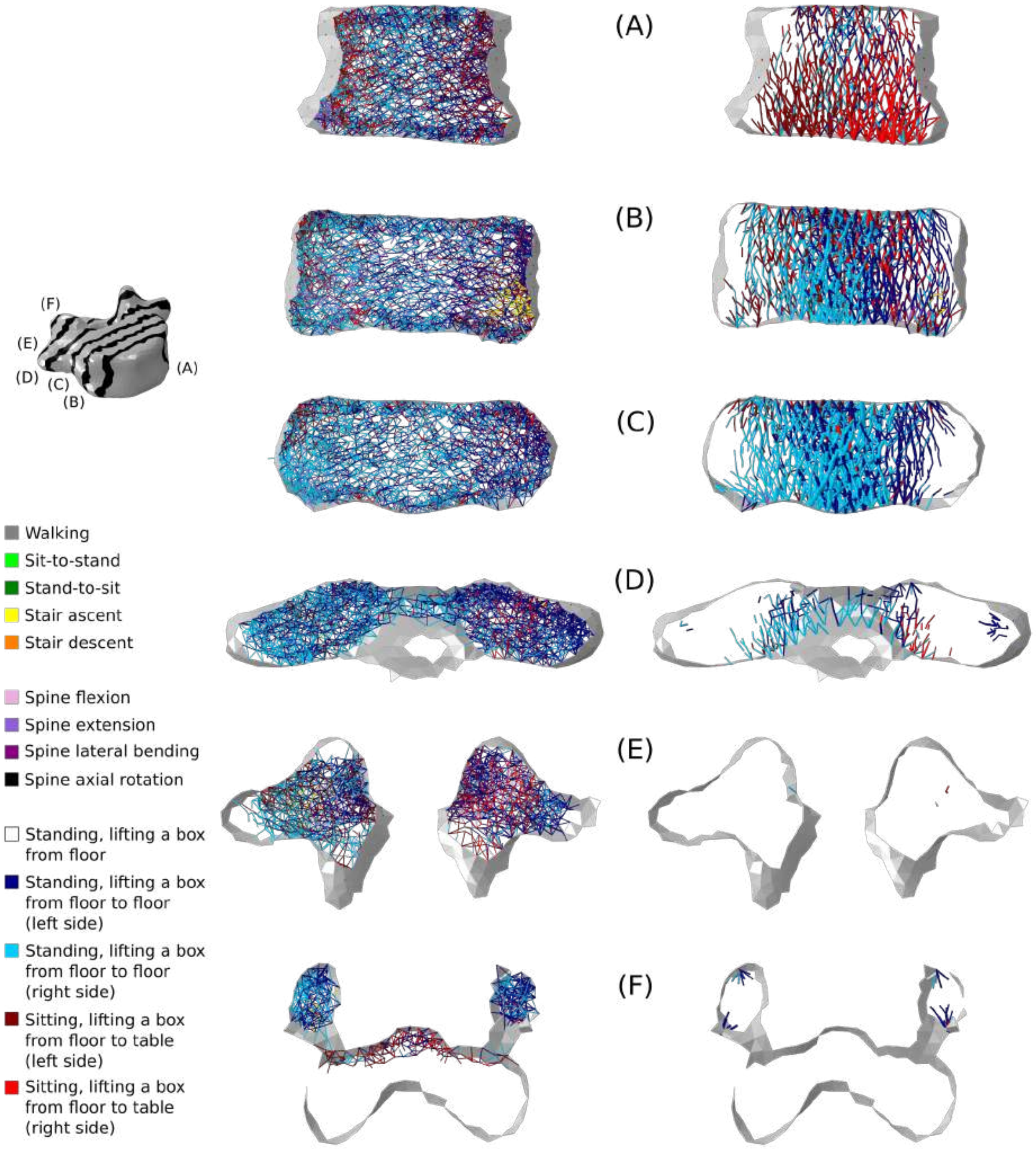

Figure S95: Selection of 3 mm coronal slices of the converged L5 model adapted to the healthy scenario. Trabecular truss elements are colour mapped based on the activity most influential to their final geometry. On the left, only trabecular truss elements representing the secondary structure (with a radius of 0.1 mm) are shown. On the right, only trabecular truss elements representing the primary structure (with a radius superior to 0.1 mm) are shown. Cortical shell elements are sown in light grey.

6.2.4 Characterisation of trabecular anisotropy

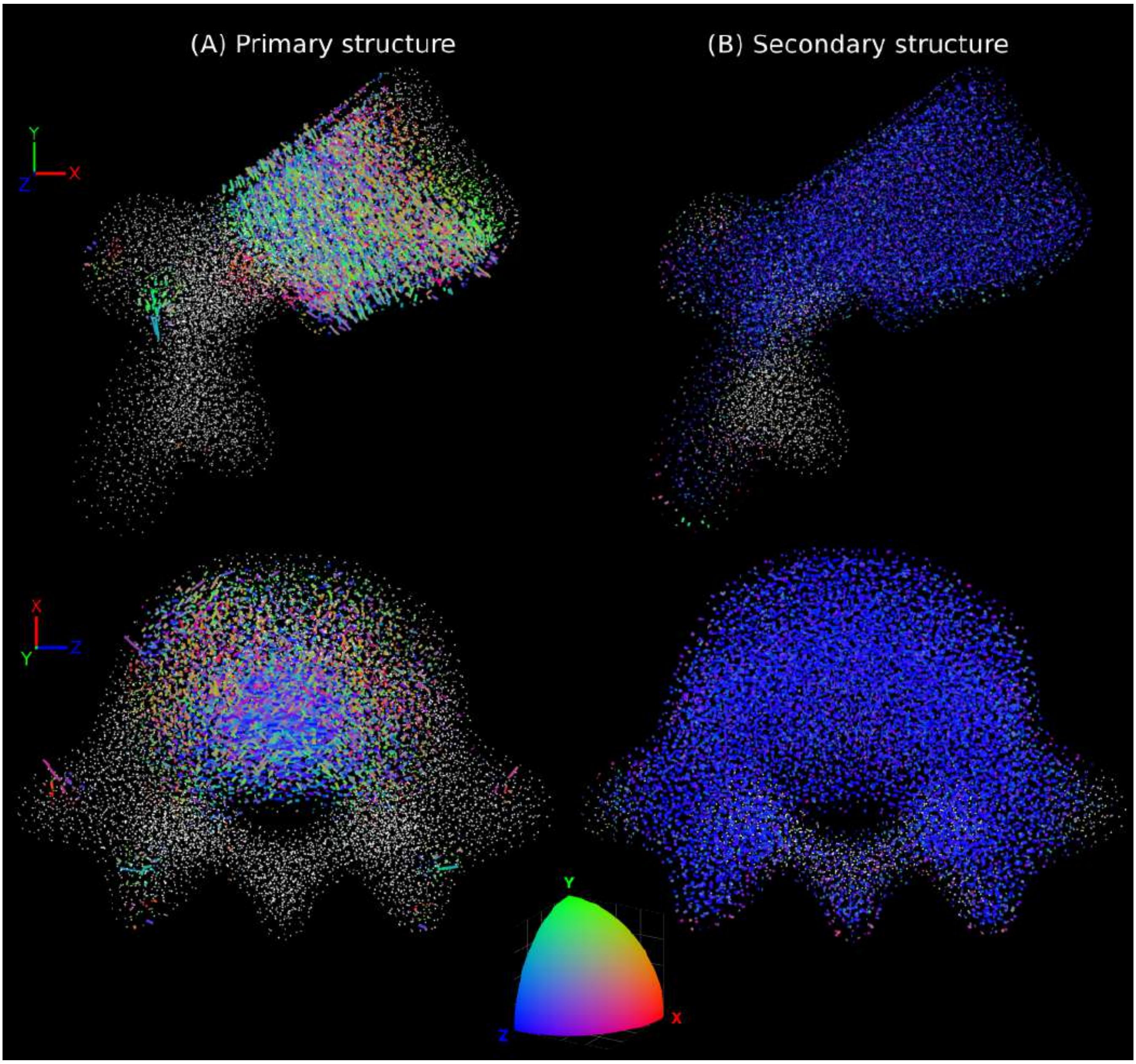

Figure S96: Characterisation of anisotropy in the trabecular bone of L5 adapted to the healthy scenario. (A) shows the orientation of the trabecular trusses of the primary structure (with a radius larger than 0.1 *mm*). (B) shows the orientation of the trabecular trusses of the secondary structure (with a radius of 0.1 *mm*). For (A) and (B), side view (top row) and top view (bottom row) are shown. Lines are attached to each trabecular node, with colour and length varying respectively with the orientations and radii of the truss elements connected to that particular node. The colour scale at the bottom shows how the colour of the lines should be interpreted. Orientation along the X, Y or Z axes are in red, green or blue respectively. Any orientation that is not colinear with these axes shows as a combination of red, green and blue. A white dot indicates a node without elements in the size range being looked at connected to it.

6.3 Sedentary scenario

6.3.1 Cortical thickness

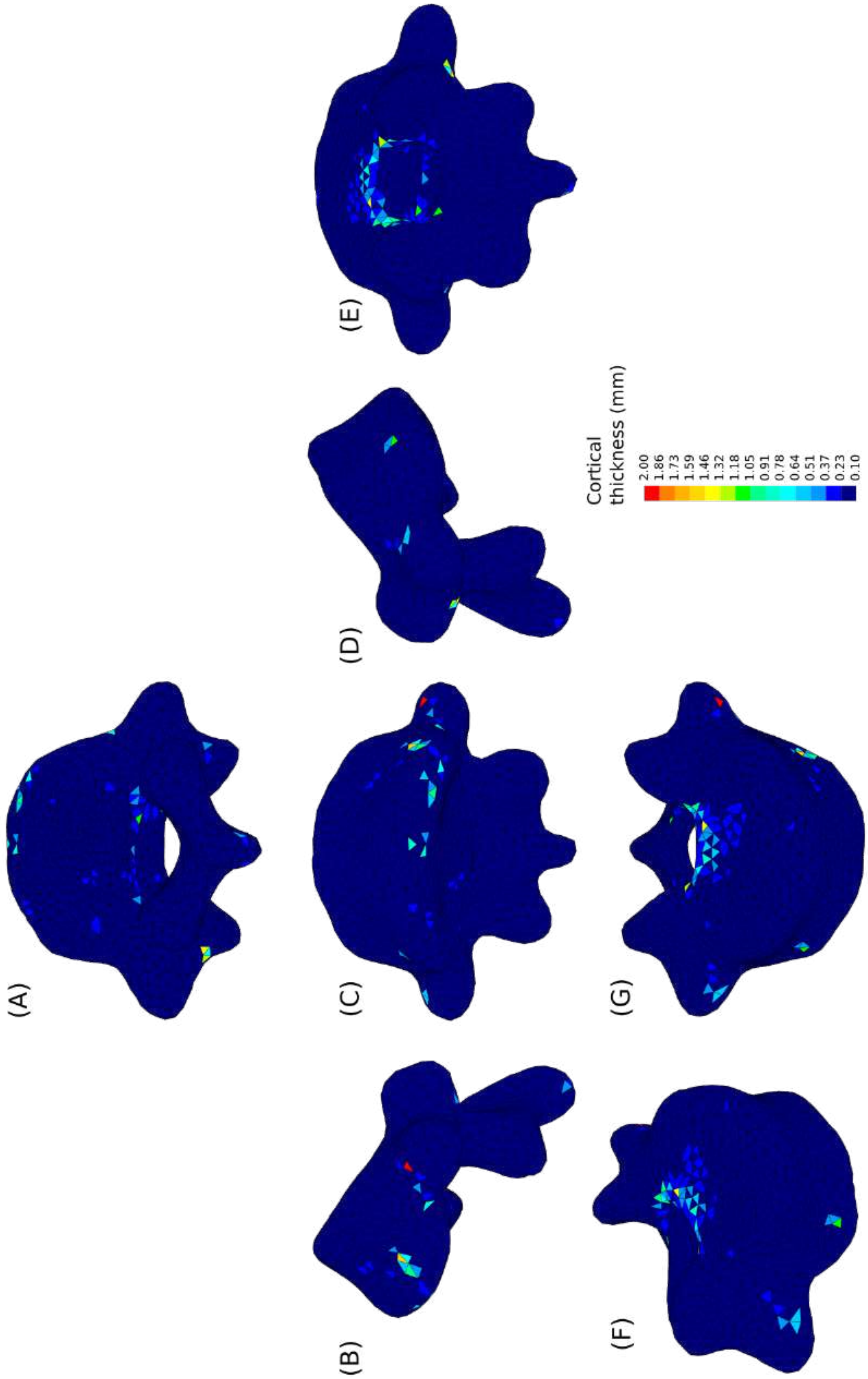

Figure S97: Cortical thickness ranging from 0.1 to 2 *mm* for the converged L5 model adapted to the sedentary scenario. (A) caudal view, (B) left lateral view, (C) frontal view, (D) right lateral view, (E) dorsal view, (F) isometric view, (G) cranial view.

6.3.2 Trabecular architecture

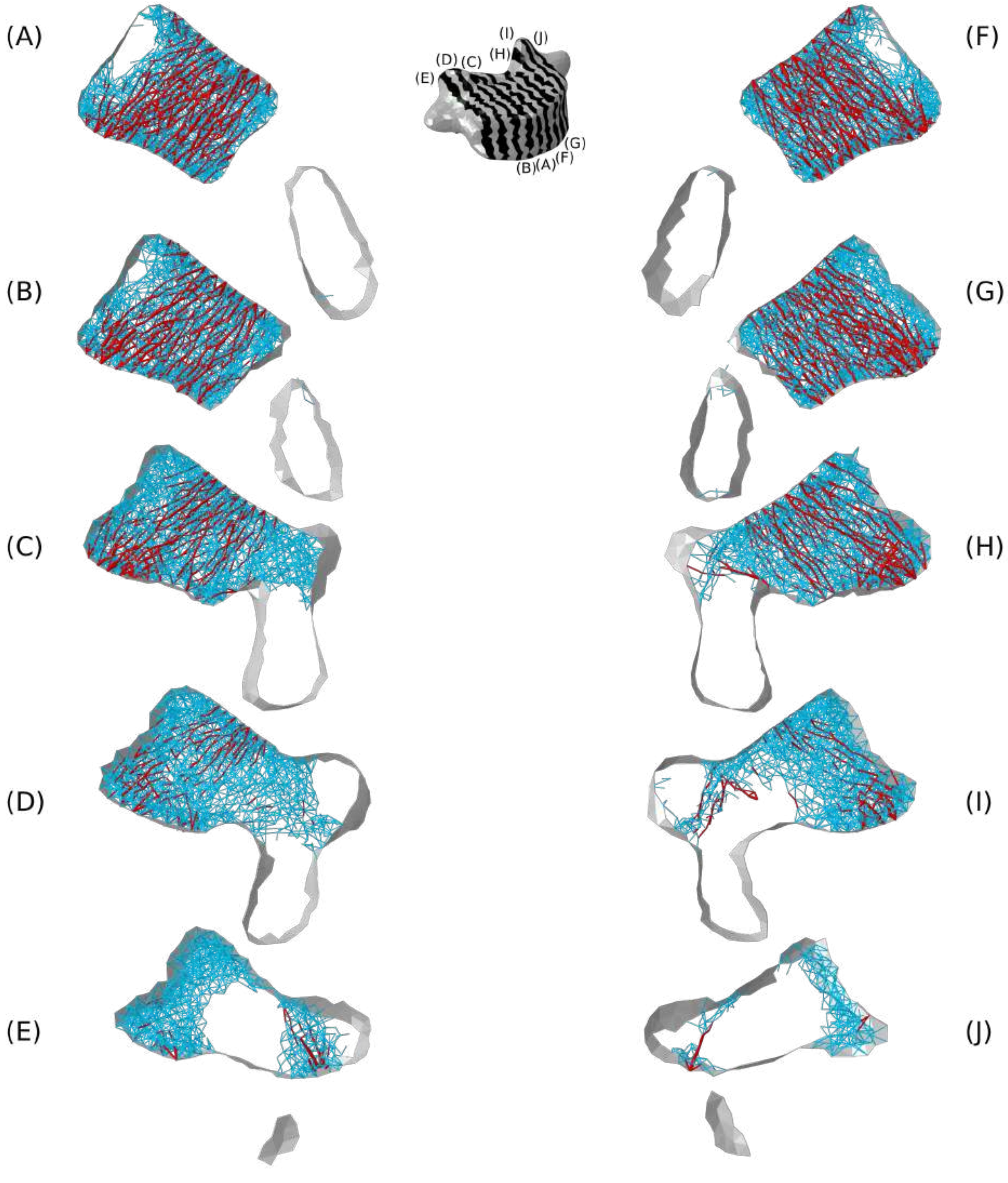

Figure S98: Selected sagittal 3 *mm* slices for the converged L5 model adapted to the sedentary scenario. Cortical shell elements are shown in grey. Trabecular truss elements representing the secondary structure (with a radius of 0.1 *mm*) are shown in blue. Thicker truss elements representing the primary structure are shown in red. Truss elements in the dead zone (with a radius of 1 μm) are not shown for clarity.

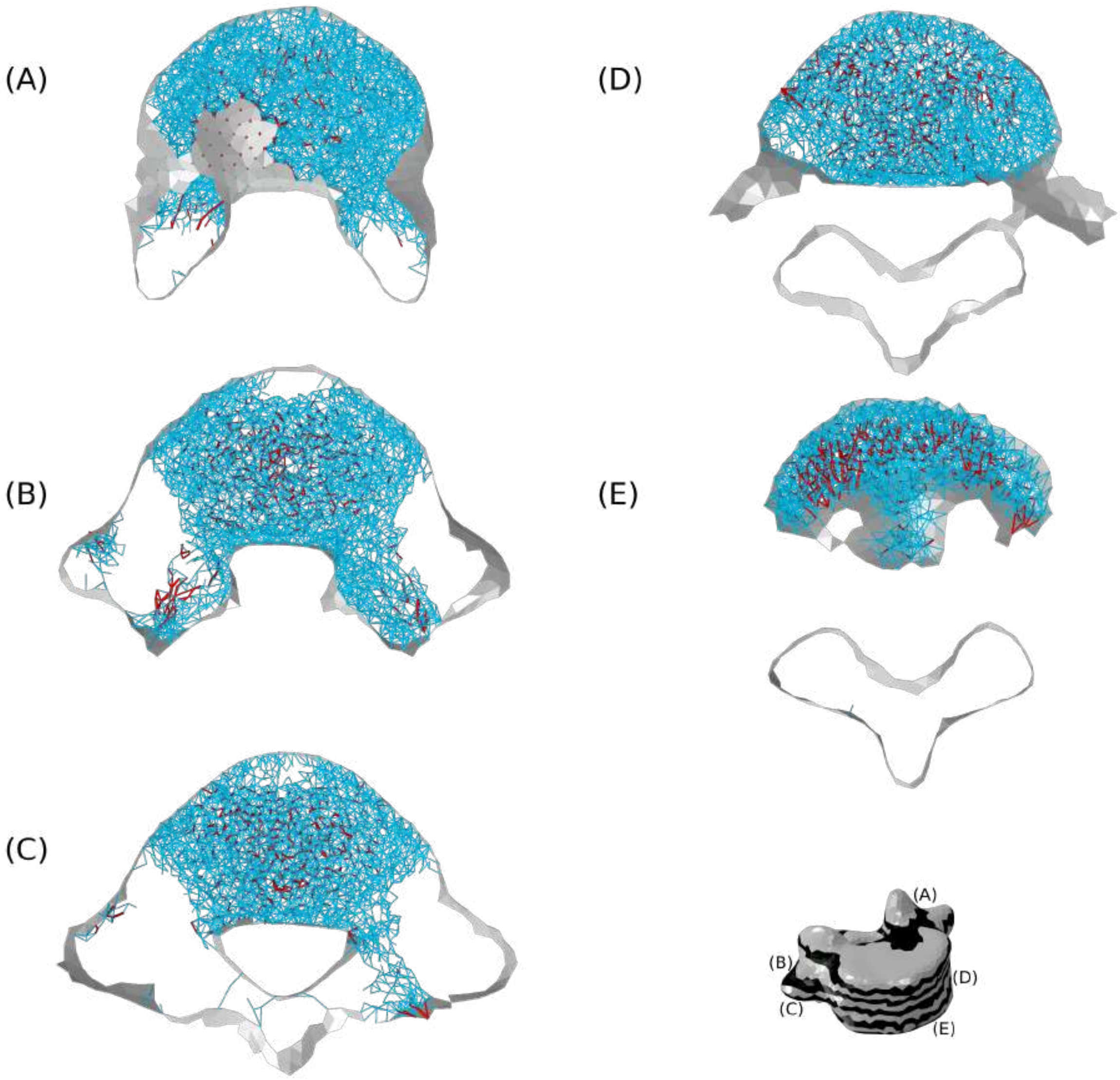

Figure S99: Selected transverse 3 mm slices for the converged L5 model adapted to the sedentary scenario. Cortical shell elements are shown in grey. Trabecular truss elements representing the secondary structure (with a radius of 0.1 mm) are shown in blue. Thicker truss elements representing the primary structure are shown in red. Truss elements in the dead zone (with a radius of 1 μm) are not shown for clarity.

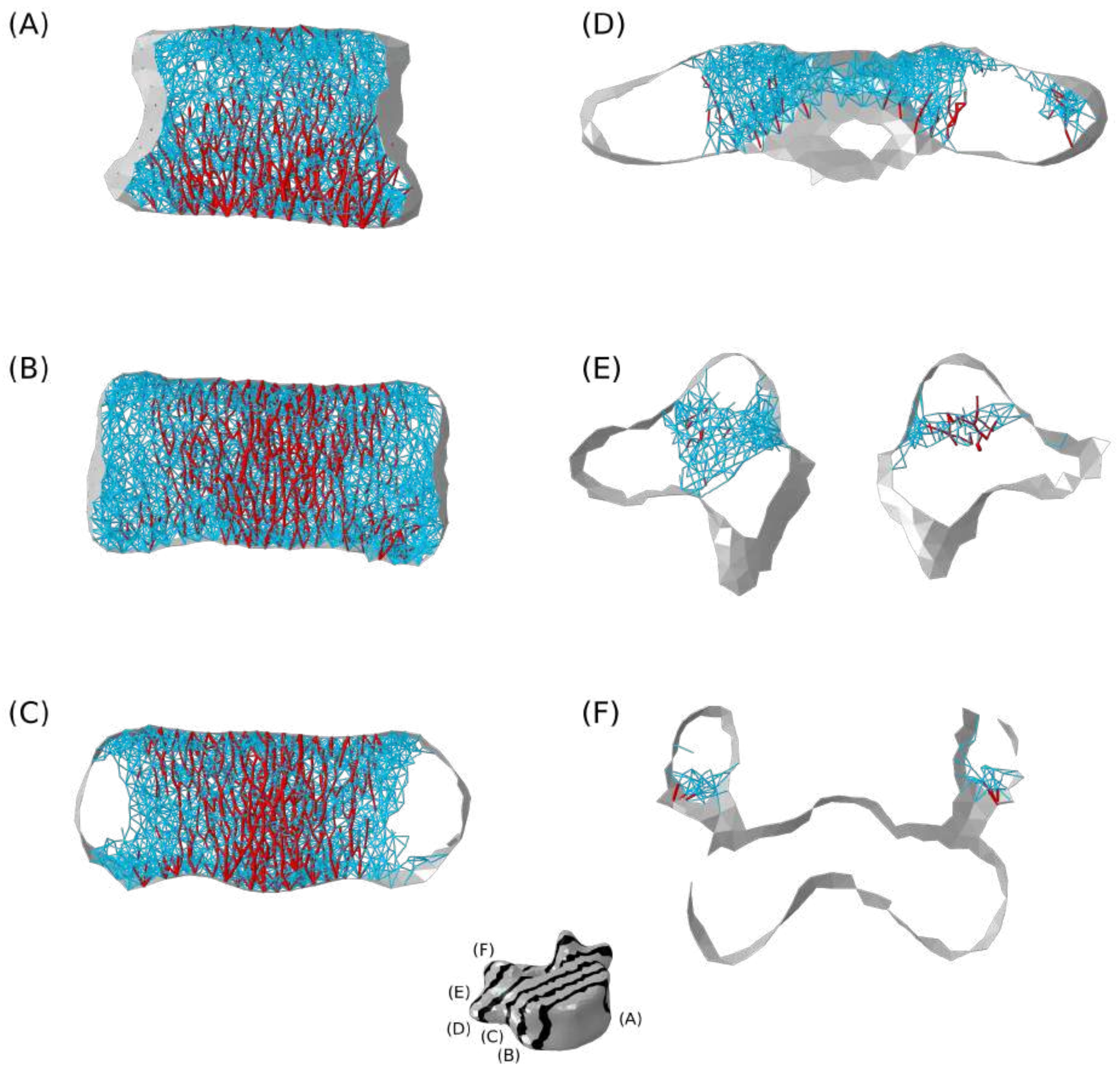

Figure S100: Selected coronal 3 mm slices for the converged L5 model adapted to the sedentary scenario. Cortical shell elements are shown in grey. Trabecular truss elements representing the secondary structure (with a radius of 0.1 mm) are shown in blue. Thicker truss elements representing the primary structure are shown in red. Truss elements in the dead zone (with a radius of 1 μm) are not shown for clarity.

6.3.3 Contribution of each activities

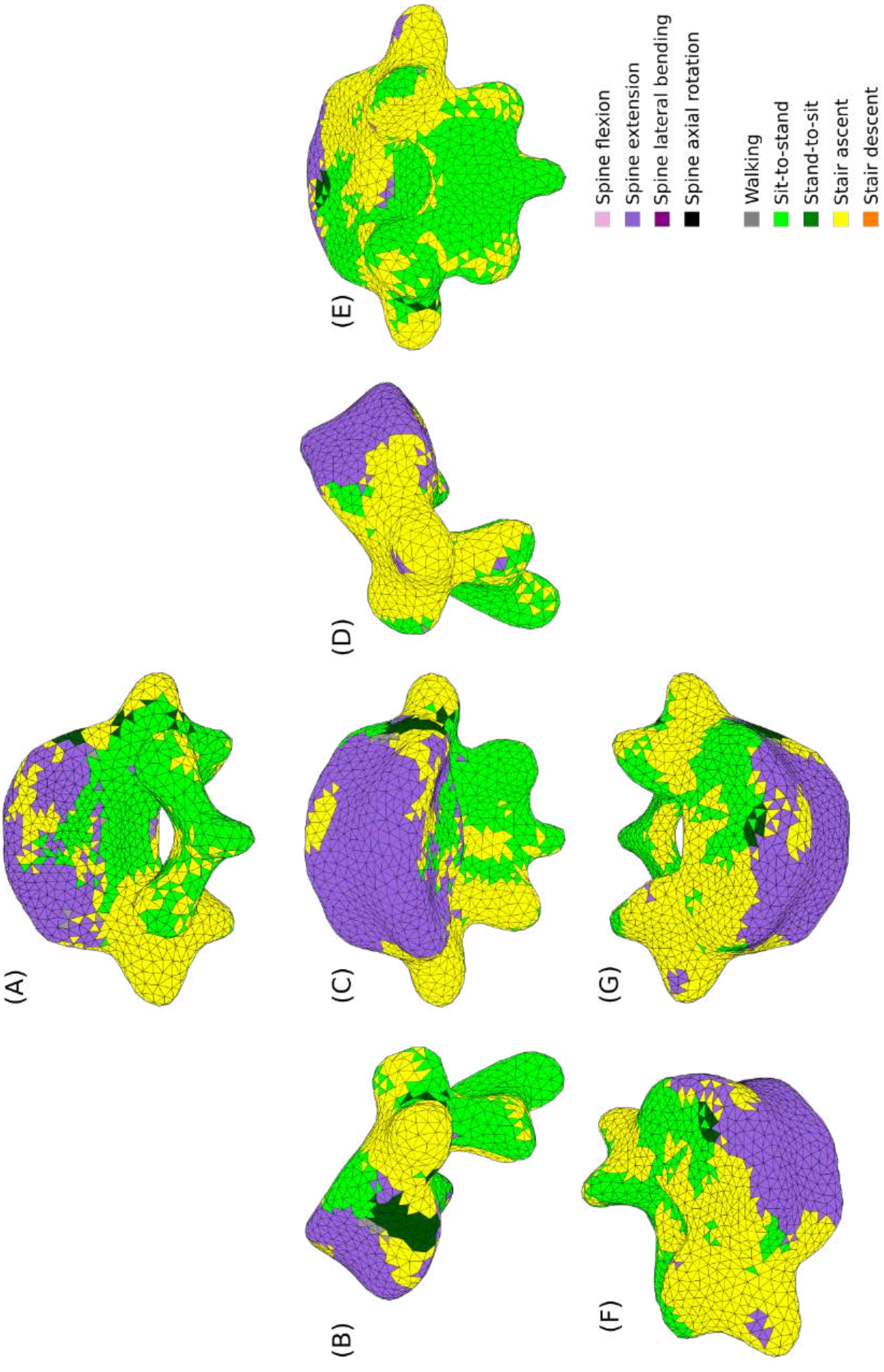

Figure S101: Contribution of each activity to the adaptation of the L5 cortical shells for the sedentary scenario. (A) caudal view, (B) left lateral view, (C) frontal view, (D) right lateral view, (E) dorsal view, (F) isometric view, (G) cranial view.

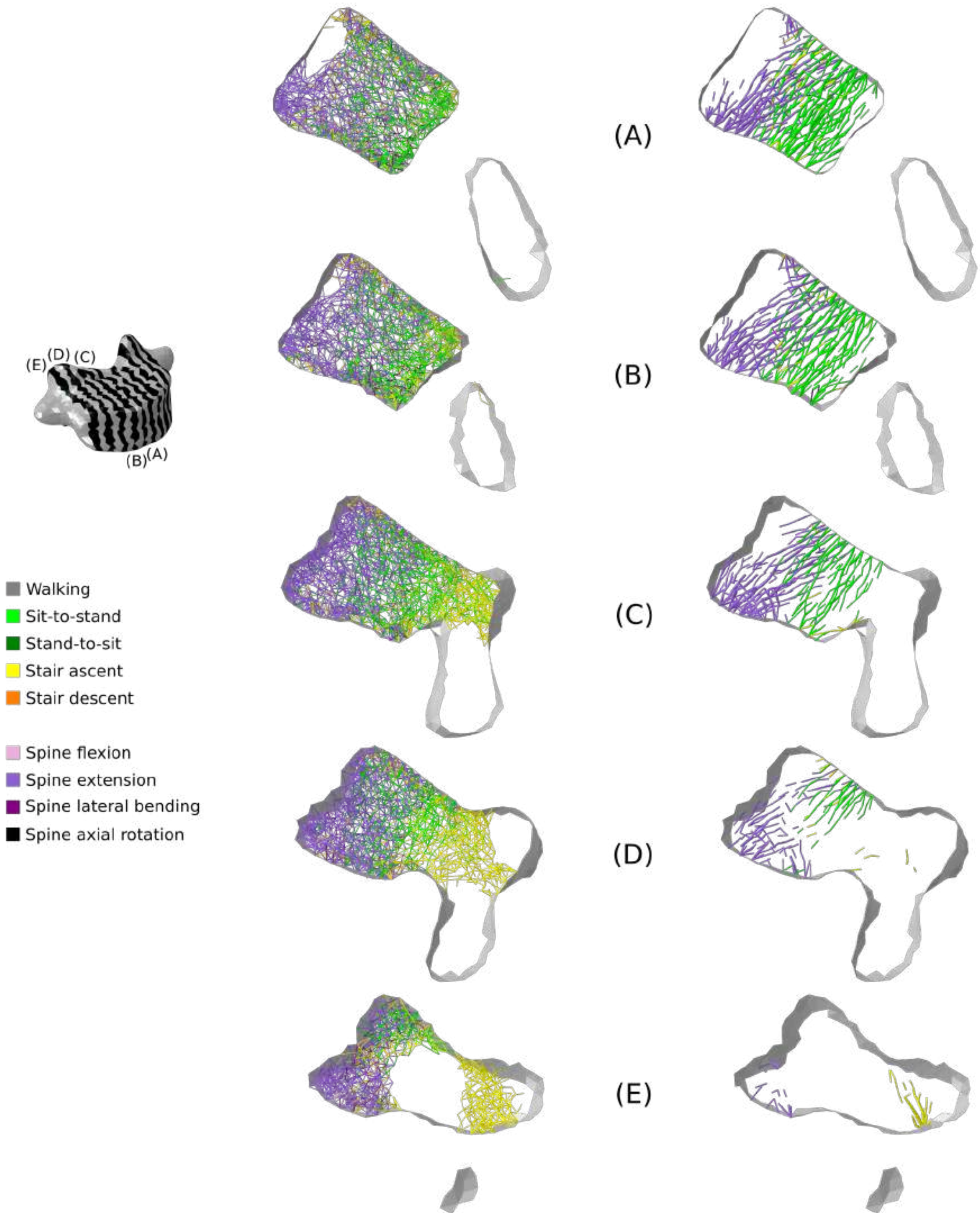

Figure S102: Selection of 3 mm sagittal slices of the right side of the converged L5 model adapted to the sedentary scenario. Trabecular truss elements are colour mapped based on the activity most influential to their final geometry. On the left, only trabecular truss elements representing the secondary structure (with a radius of 0.1 mm) are shown. On the right, only trabecular truss elements representing the primary structure (with a radius superior to 0.1 mm) are shown. Cortical shell elements are sown in light grey.

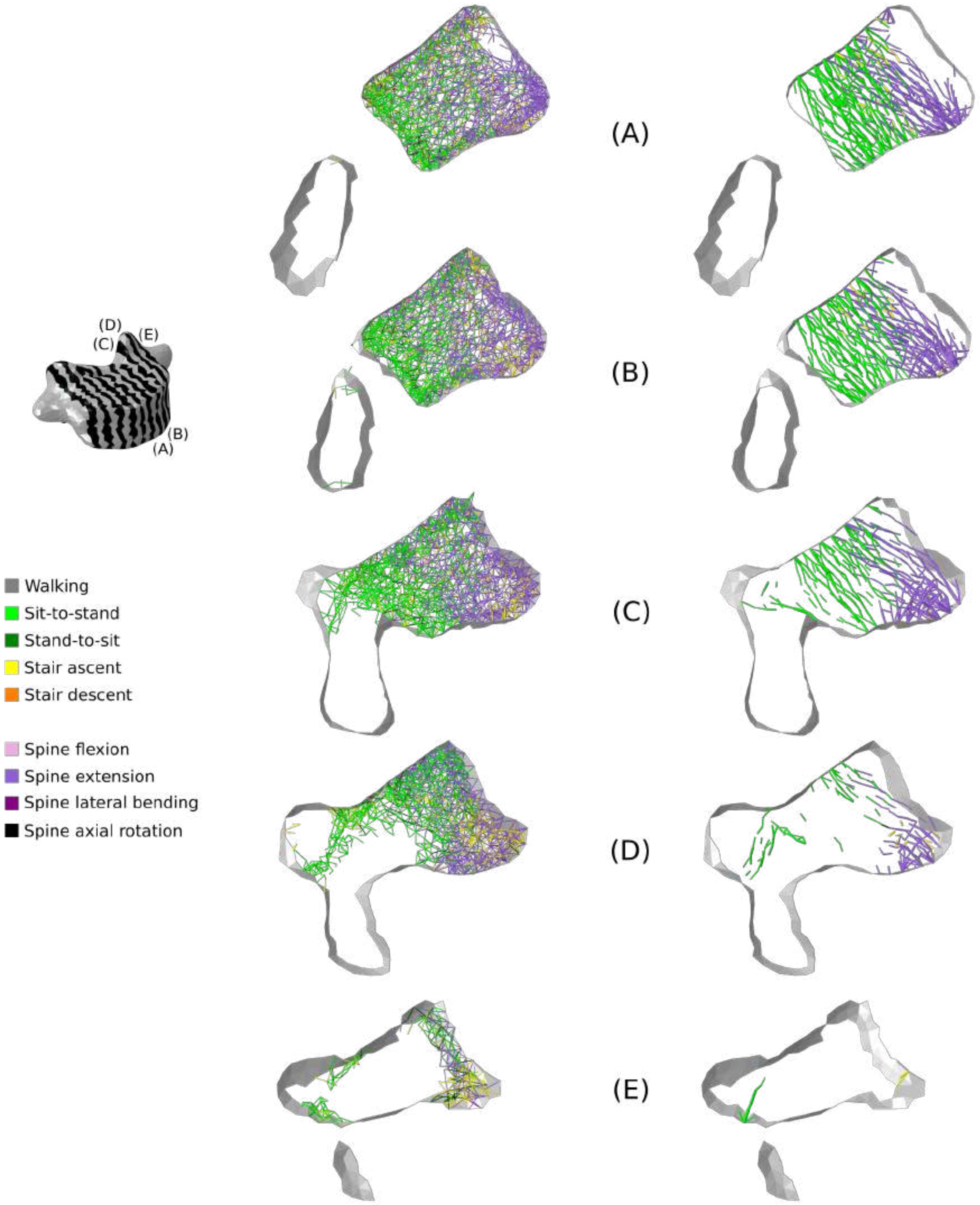

Figure S103: Selection of 3 *mm* sagittal slices of the left side of the converged L5 model adapted to the sedentary scenario. Trabecular truss elements are colour mapped based on the activity most influential to their final geometry. On the left, only trabecular truss elements representing the secondary structure (with a radius of 0.1 *mm*) are shown. On the right, only trabecular truss elements representing the primary structure (with a radius superior to 0.1 *mm*) are shown. Cortical shell elements are sown in light grey.

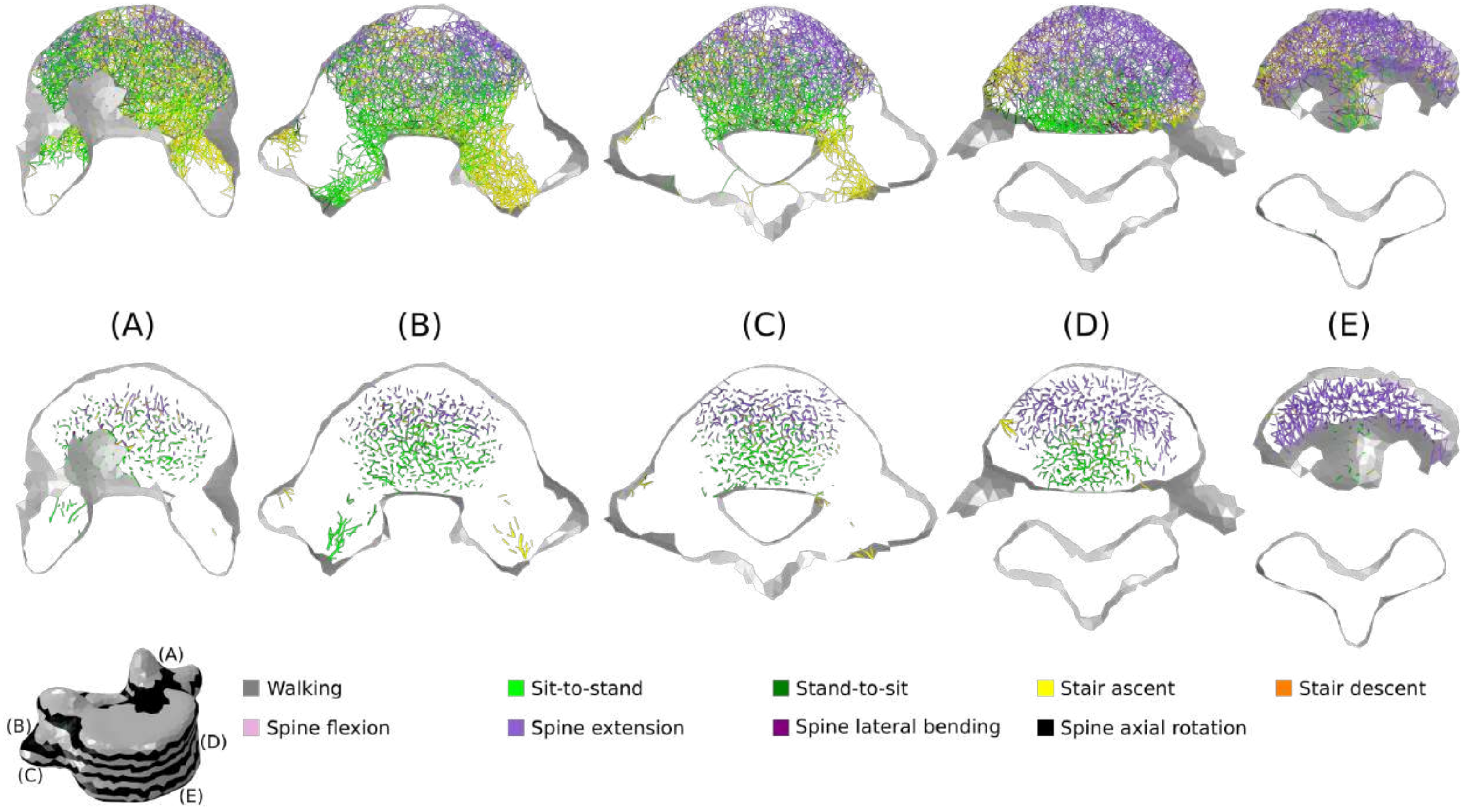

Figure S104: Selection of 3 *mm* transverse slices of the converged L5 model adapted to the sedentary scenario. Trabecular truss elements are colour mapped based on the activity most influential to their final geometry. On the top row, only trabecular truss elements representing the secondary structure (with a radius of 0.1 *mm*) are shown. On the bottom row, only trabecular truss elements with a radius superior to 0.1 *mm* are shown. Cortical shell elements are sown in light grey.

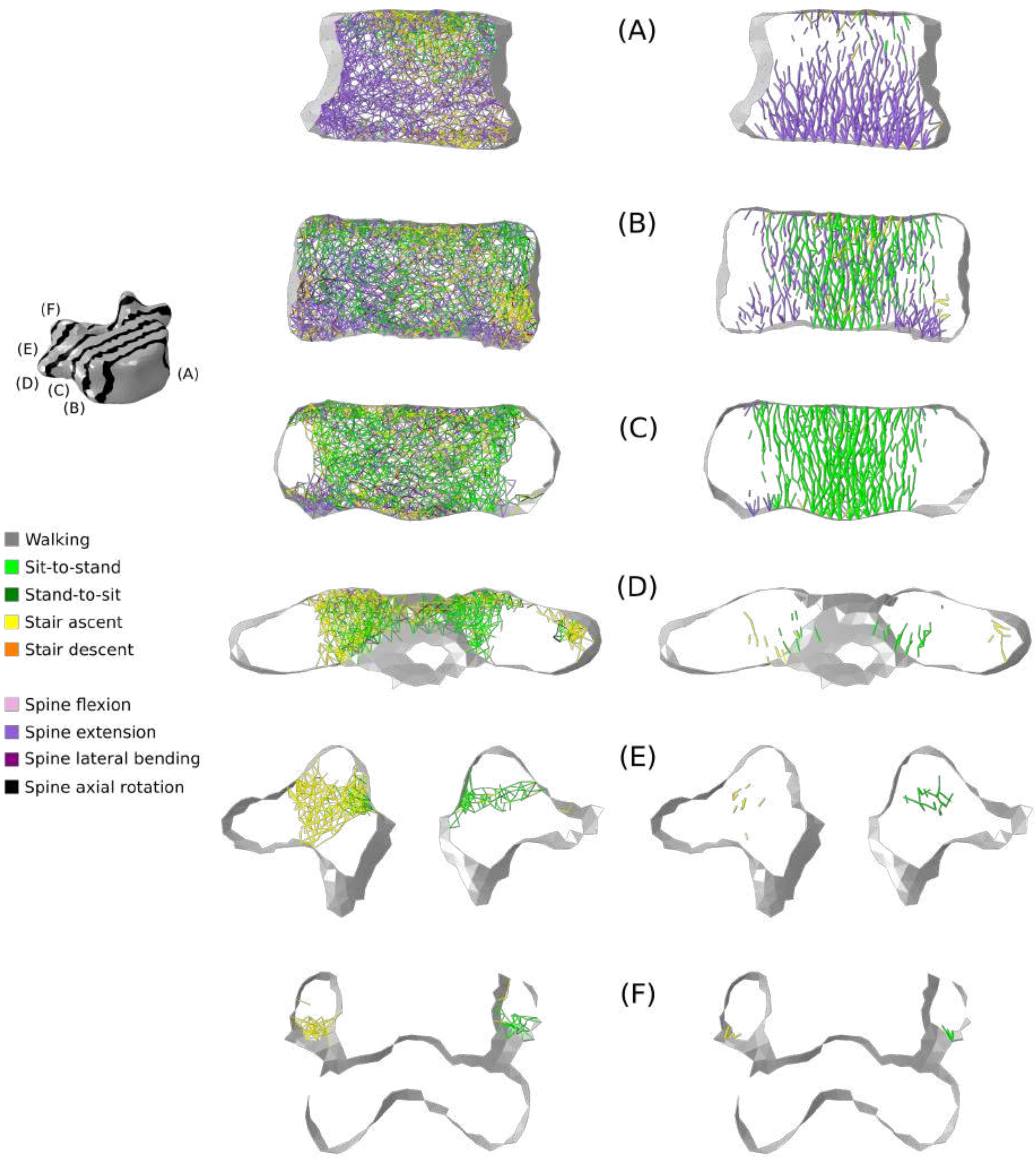

Figure S105: Selection of 3 *mm* coronal slices of the converged L5 model adapted to the sedentary scenario. Trabecular truss elements are colour mapped based on the activity most influential to their final geometry. On the left, only trabecular truss elements representing the secondary structure (with a radius of 0.1 *mm*) are shown. On the right, only trabecular truss elements representing the primary structure (with a radius superior to 0.1 *mm*) are shown. Cortical shell elements are sown in light grey.
